# Quaternary rodents of South Africa: A companion guide for cranio-dental identification

**DOI:** 10.1371/journal.pone.0289812

**Published:** 2023-11-28

**Authors:** Pierre Linchamps, D. Margaret Avery, Raphaël Cornette, Christiane Denys, Thalassa Matthews, Emmanuelle Stoetzel

**Affiliations:** 1 Institut de Systématique, Evolution, Biodiversité (ISYEB) UMR 7205, CNRS, Muséum National d’Histoire Naturelle, UPMC, EPHE, Sorbonne Universités, Paris, France; 2 Histoire Naturelle de l’Homme Préhistorique (HNHP) UMR 7194, CNRS, Muséum National d’Histoire Naturelle, UPVD, Sorbonne Universités, Paris, France; 3 Iziko Museums of South Africa, Cape Town, South Africa; University of Puthisastra, CAMBODIA

## Abstract

Rodentia is the most species-rich order among mammals. The Republic of South Africa harbours a high rodent diversity whose taxonomy and phylogeny have been extensively studied using genetic tools. Such advances have led to the establishment of new faunal lists for the country. Because rodents are frequently recovered from archaeological cave site material and owl pellets, and constitute prime material for studying both past and present environmental conditions, it is necessary to characterize their osteological remains. The skull and teeth are the most useful diagnostic skeletal elements preserved in modern and fossil accumulations. This key provides updated craniodental criteria for identifying rodent genera found in Quaternary deposits, and modern material from the Republic of South Africa, thus facilitating research on past and present rodent diversity.

## 1. Introduction

The Republic of South Africa (RSA) has a rich storehouse of Quaternary archaeological sites, spanning the last 2.6 MYA from the Early Pleistocene to the late Holocene. It possesses one of the world’s richest fossil hominin inventories documenting the origin of modern humans, as well as an infinite amount of animal and plant fossils which have enabled a detailed palaeo-reconstruction of the environments in which we evolved. Small mammals, especially rodents, have been widely used for reconstructing past environments from the Pleistocene to the Holocene [e.g., 1–10]. They have an advantage in that they are frequently abundant in the fossil record, and, because they do not migrate and have small home ranges, generally provide a clearer and more detailed picture of local conditions than the larger mammals.

Rodents are a very diverse group of mammals, with an exceptional taxonomic and phenotypic diversity, and constitute the most species-rich order among mammals, making up over 40% of all living mammal species [11–13]. This variability in body shapes and sizes, together with morphological and physiological adaptations, resulted in the successful colonization of most terrestrial environments [12–14]. Rodents are found in almost every habitat around the world; from open, dry deserts, to thick, wet rainforests, and display a wide array of ecomorphological adaptations including fossorial (burrowing), saltatorial (jumping), arboreal, subaquatic, and gliding forms [15].

Rodents are the preferred prey of many predators, including owls and small and meso-carnivores, which are often the agents responsible for the accumulation of micromammal remains in archaeological and palaeontological cave sites [16–19]. For instance, diurnal and nocturnal raptors regurgitate pellets that contain the undigested parts (mostly hair, bones, and teeth) of their prey at roost and nest sites; those pellets may accumulate over a long period of time, and bones and teeth become incorporated into the sediment as they break down. Analysis of modern owl pellets thus provides useful comparative information for identifying cranial fragments and teeth of fossil Pleistocene microfauna.

Whether the remains are fossil or (sub)contemporaneous, the material used for identification are the mandibles and maxillae (these are seldom complete, and rarely retain all the teeth) and also isolated teeth. The most effective method for identification of specimens at species level is a morpho-anatomical comparison of the fossil material with a modern reference collection. However, these are not always easy to access, and modern, comparative collections in museums, for example, may contain specimens which are misidentified, poorly documented, or which have unresolved taxonomies. A solution to these problems is the use of an identification key. There are several available keys for the identification of southern African rodents [20–22] but they generally rely on external characteristics such as length of the body, proportions of the tail or hindfeet, color and pattern of the pelage or number of nipples, and are therefore of little use for identifying cranio-dental remains. The only exhaustive existing keys based on cranial characters are those of Coetzee [23] and De Graaff [24], which are not up to date in terms of taxonomic research, and some other publications cover only specific families or subfamilies [25–27]. More recently, the systematics of many taxa has been partially resolved, with several new species being described and published in taxonomic reference publications [12, 13, 22], as well as in many systematic studies based upon genetic analyses and field surveys [e.g., 28–31]. Many recently described taxa are missing from earlier keys and this, together with the problems related to obtaining reliable comparative collections, has prevented the correct identification, and effective palaeoenvironmental reconstructions of quaternary rodent assemblages.

Here we propose a new identification key of cranio-dental morphological features which are of generic diagnostic importance, and apply them to material extracted from owl pellets and Quaternary fossil assemblages. The key, which follows the systematics of Wilson et al. [12, 13], covers all genera that occurred in South Africa during the Quaternary, including extinct fossil taxa, as well as more recently introduced taxa. Since cryptic diversity has been showed to occur within several genera, and because many uncertainties remain regarding the validity and/or taxonomic status of some extinct species, we chose not to provide identification guidance beyond the genus level, pending further systematic, taxonomic, and morphometric investigations. To assist in the identification of specimens in the field, and to get an idea of the relative size of each genus, some synthetic plates with full-scale photos of modern specimens are provided as supplementary material (S1 Fig for upper jaws, S2 Fig for lower jaws, S1 Table for references of photographed specimens; the plates S1 and S2 Figs should be printed at actual size to maintain full-scale).

As this key is mostly dedicated to researchers working in palaeontology, archaeology, and taphonomy, diagnostic information is based on features and materials that are most often recovered from fossil or taphonomic sites, i.e., upper and lower toothrows, and mandibles and maxillae with, and without, teeth. Some other cranial features which are often useful for identifying rodents, such as the auditory bullae and width of the nasals, are not dealt with in detail here because they are not generally preserved on the material studied.

## 2. Past and present rodent diversity in South Africa

There are currently 35 genera of rodents living in RSA (see Table 1), with one additional valid fossil genus listed for the Quaternary. These 36 genera belong to nine families:

- The Muridae is the largest family of rodents, including many diverse species such of mice, rats and gerbils.
- Mole-rats of the family Bathyergidae are burrowing rodent with cylindrical bodies and short limbs that have distinct morphological adaptations to subterranean life.
- Dormice of the family Gliridae are small nocturnal rodents, largely arboreal and well adapted to climbing; in sub-Saharan Africa, they are only represented by the genus *Graphiurus*.
- Porcupines of the family Hystricidae are large, nocturnal rodents that have quills (spines) for defense against predators.
- The family Petromuridae is monospecific, it contains the dassie rat (*Petromus typicus*)—a medium size rodent which inhabits rocky outcrops.
- Springhares also belong to a single genus which constitutes the family Pedetidae; they are nocturnal rodents whose method of locomotion is hopping with their strong hind legs.
- The family Nesomyidae is diverse, and contains small to medium size morphologically varied rodents endemic to continental Africa and Madagascar.
- South African squirrels of the family Sciuridae are terrestrial (*Geosciurus*) or semi-arboreal (*Paraxerus*) diurnal rodents with an elongated body and bushy tail.
- Cane rats of the family Thryonomyidae are large, heavily built rodents that live in marshy areas and along riverbanks.

**Table 1:** List of Pleistocene and modern rodent species from Republic of South Africa (RSA) based on Wilson et al. [2016, 2017]. Taxa are listed in alphabetical order. Published extinct taxa are identified by the symbol † in the table.

Family	Subfamily	Genus	Species	Common generic name
Bathyergidae	Bathyerginae	*Bathyergus*	*janetta*, *suillus*	dune mole-rat
		*Cryptomys*	*hottentotus*, *†robertsi*	mole-rat
		*Fukomys*	*damarensis*	mole-rat
		*Georychus*	*capensis*	Cape mole-rat
Gliridae	Graphiurinae	*Graphiurus*	*microtis*, *murinus*, *ocularis*, *platyops*, *rupicola*	dormouse
Hystricidae		*Hystrix*	*africaeaustralis*, *†makapanensis*	crested porcupine
Muridae	Deomyinae	*Acomys*	*selousi*, *subspinosus*	spiny mouse
	Gerbillinae	*Desmodillus*	*auricularis*	Cape sort-tailed gerbil
		*Gerbilliscus*	*afra*, *brantsii*, *leucogaster*, *paeba*, *vallinus*	gerbil & hairy-footed gerbil
	Murinae	*Aethomys*	*chrysophilus*, *ineptus*	veld rat
		*Dasymys*	*capensis*, *incomtus*, *robertsii*	shaggy rat
		*Grammomys*	*cometes*, *dolichurus*	thicket rat
		*Lemniscomys*	*rosalia*	grass mouse
		*Mastomys*	*coucha*, *natalensis*	multimammate mouse
		*Micaelamys*	*granti*, *namaquensis*	lesser veld rat
		*Mus*	*indutus*, *minutoides*, *musculus*, *neavei*	old world & pygmy mouse
		*Myomyscus*	*verreauxii*	meadow mouse
		*Otomys*	*angoniensis*, *auratus*, *†gracilis*, *irroratus*, *karoensis*, *laminatus*, *sloggetti*, *unisulcatus*	vlei rat or laminate-toothed rat
		*Parotomys*	*brantsii*, *littledalei*	whistling rat
		*Rattus*	*rattus*, *norvegicus*, *tanezumi*	rat
		*Rhabdomys*	*bechuanae*, *dilectus*, *intermedius*, *pumilio*	four-striped grass mouse
		*Thallomys*	*†debruyni*, *nigricauda*, *paedulcus*, *shortridgei*	acacia rat or tree rat
		*Zelotomys*	*woosnami*	broad-headed mouse
Nesomyidae	Cricetomyinae	*Cricetomys*	*ansorgei*	giant pouched rat
		*Saccostomus*	*campestris*	pouched mouse
	Dendromurinae	*Dendromus*	*melanotis*, *mesomelas*, *mystacalis*, *nyikae*	African climbing mouse
		*Malacothrix*	*typica*	Long-eared Mouse
		*Steatomys*	*krebsii*, *pratensis*	fat mouse
	Mystromyinae	*Mystromys*	*albicaudatus*, *†hausleitneri*	African white-tailed rat
		*†Proodontomys*	*†cookei*	
	Petromyscinae	*Petromyscus*	*barbouri*, *collinus*, *monticularis*	pygmy rock mouse
Pedetidae		*Pedetes*	*capensis*, *†hagenstadti*	springhare
Petromuridae		*Petromus*	*typicus*	noki or dassie rat
Sciuridae		*Geosciurus*	*inauris*, *princeps*	ground squirrel
		*Paraxerus*	*cepapi*, *palliatus*	bush squirrel
Thryonomyidae		*Thryonomys*	*swinderianus*	cane rat

In the genus accounts of this work, we provide a short synthesis of the most recent research on the phylogeny and geographical distribution of each genus and species, as well as an overview of the fossil record in South Africa during the Quaternary period.

## 3. General anatomy and glossary for the lower and upper jaws

**Fig 1:**
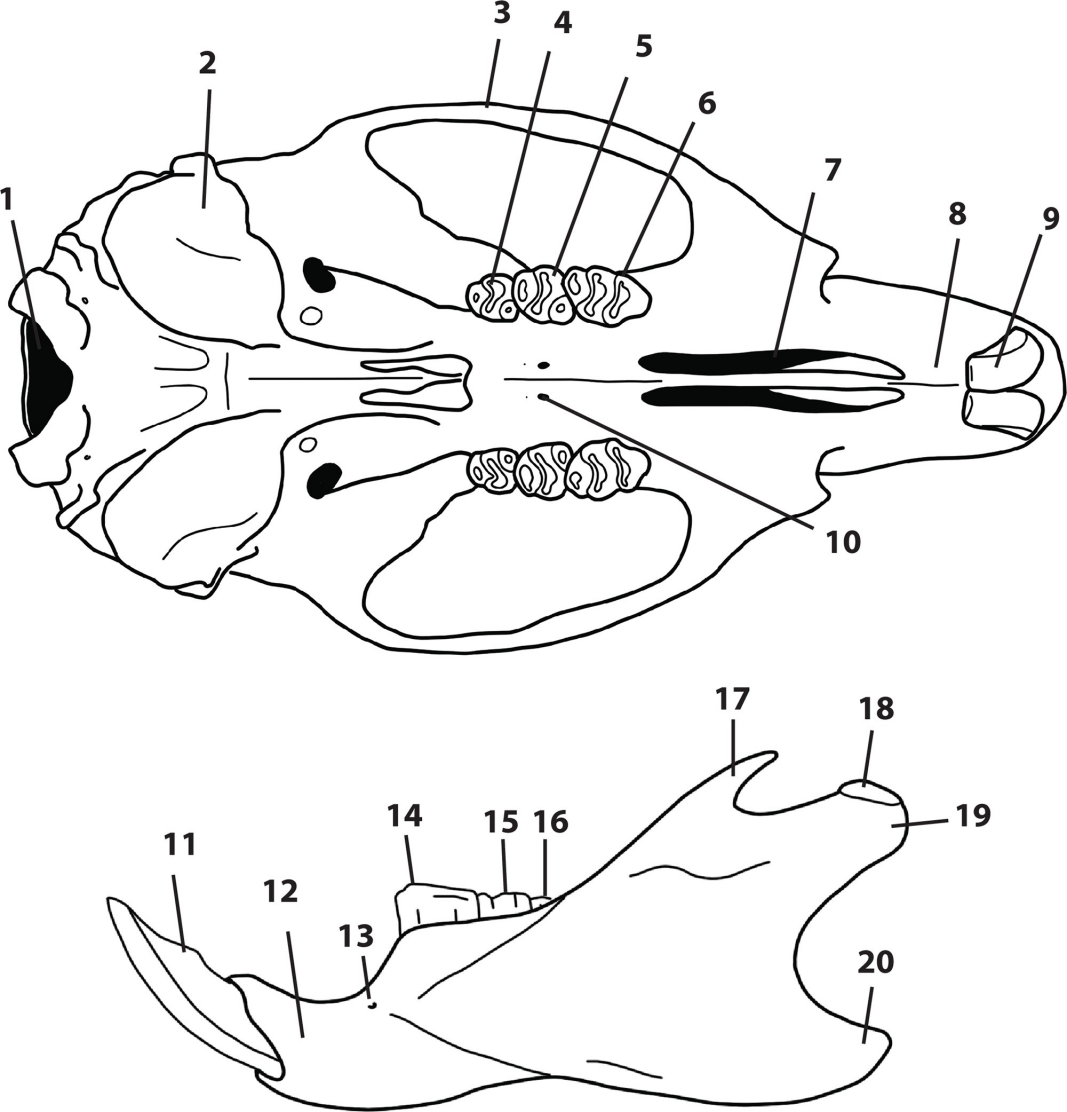
Schematics and nomenclature of rodent cranium (1–10) and mandible (11–20). 1: foramen magnum; 2: tympanic bulla; 3: zygomatic arch; 4: third upper molar (M^3^); 5: second upper molar (M^2^); 6: first upper molar (M^1^); 7: first or anterior palatal foramen; 8: diastema; 9: incisor; 10: second or posterior palatal foramen; 11: incisor; 12: body of mandible; 13: mental foramen; 14: first lower molar (M_1_); 15: second lower molar (M_2_); 16: third lower molar (M_3_); 17: coronoid process; 18: articular surface; 19: condylar process; 20: angular process.

Despite the significant number of species, and a great diversity of morphological and ecological adaptations, rodents are remarkably uniform regarding the general morphology of the skull and dentition. The upper and lower jaws (Fig 1) each support a single pair of large, ever-growing scalpriform incisors. The enamel is mainly limited to the outer surface of the incisors. The presence of iron in the mineral phase of incisor enamel can give the surface of some rodent incisors a yellowish to orange appearance which can be diagnostic for some genera; however, the incisor colour criterion is not always usable in an owl pellet and fossil context due to taphonomic alteration (digestion, diagenesis, staining with sediment, etc.). A large diastema is present between the incisors and the cheekteeth, allowing the lips to fold inwards in order to prevent debris interfering with chewing activity when gnawing. The maximum number of cheekteeth is four for the lower jaw, and five for the upper jaw, with murids having only three molars in each jaw and an incisor in each mandible and premaxilla. The pattern on the occlusal surface of molars is remarkably varied and diverse among rodents, and thus is of great diagnostic taxonomic value.

Below is a glossary of the main terms that will be used in the following anatomical descriptions:

**angular process**. Also called *processus angularis*; process at the posterior lower corner of the mandible that serves for muscular attachment.

**brachyodonty**. Cheekteeth with short crowns.

**bunodonty**. Cheekteeth in which the cusps are high and rounded on the occlusal surface of the crown.

**buno-lophodonty**. Cheekteeth in which the cusps tend to connect transversally but remain partially individualized at low wear.

**condylar process**. Process at the posterior upper corner of the mandible that articulates with the glenoid fossa of the skull, forming the lower hinge of the jaw articulations.

**coronoid process**. Process at the anterior upper corner of the mandible ramus situated anteriorly to the condylar process. It serves as an attachment point for the temporalis muscle and does not participate in the jaw articulation.

**cusp**. Occlusal eminence on the surface of a tooth.

**cusplet**. Small cusp, often located on the edge of a tooth.

**diastema**. Gap between the incisor and the cheekteeth.

**foramen**. Orifice in a bone through which nerves and blood vessels pass.

**hypselodonty**. Ever-growing teeth with very long crown and short roots/rootless.

**hypsodonty**. Teeth with high crowns.

**Fig 2:**
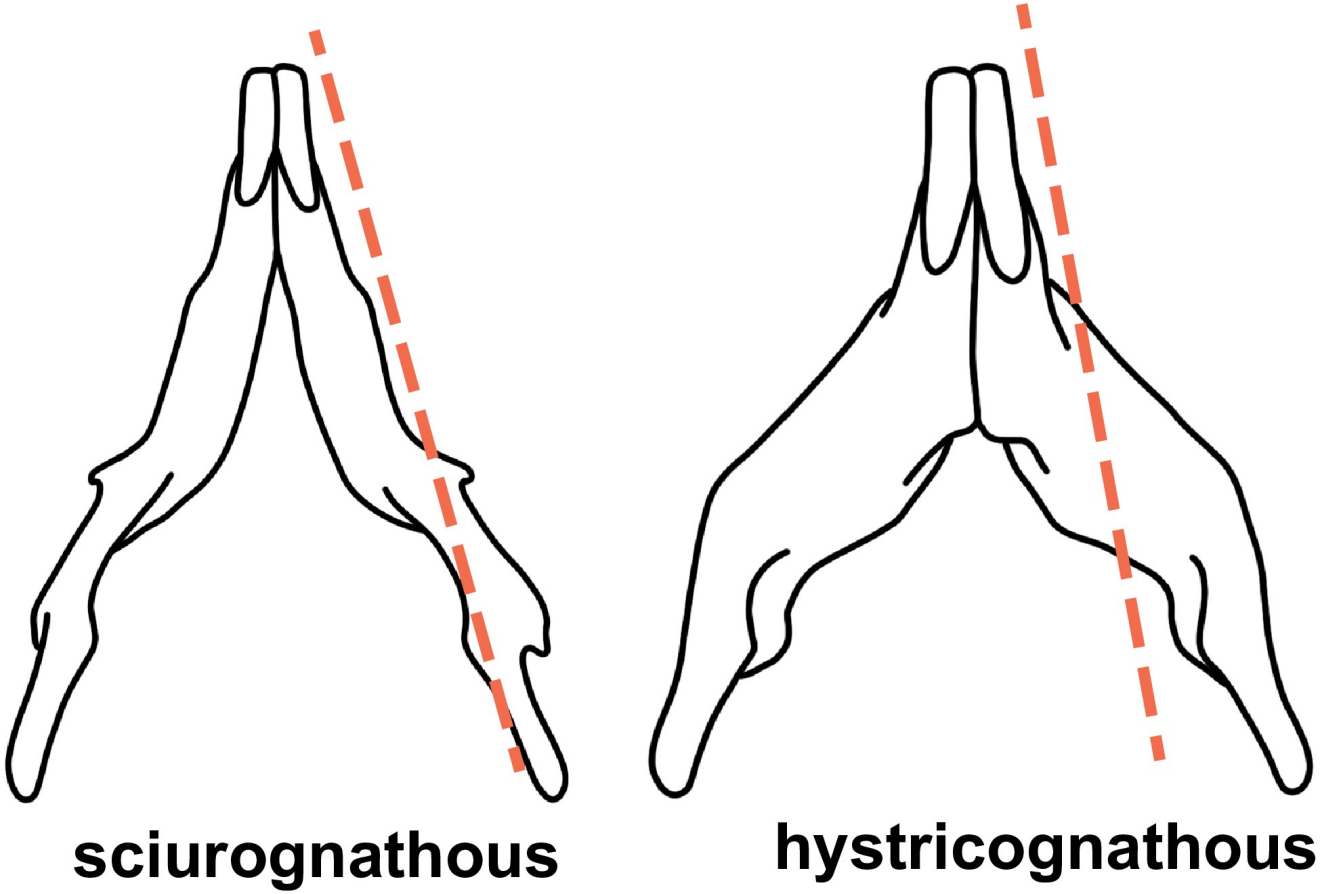
Two mandibles in ventral view showing a sciurognathous condition (left) and hystricognathous condition (right).

**hystricognathous**. Condition of the mandible with the angular process deflected lateral to the plane that includes the alveolus of the incisors (see Fig 2).

**Fig 3:**
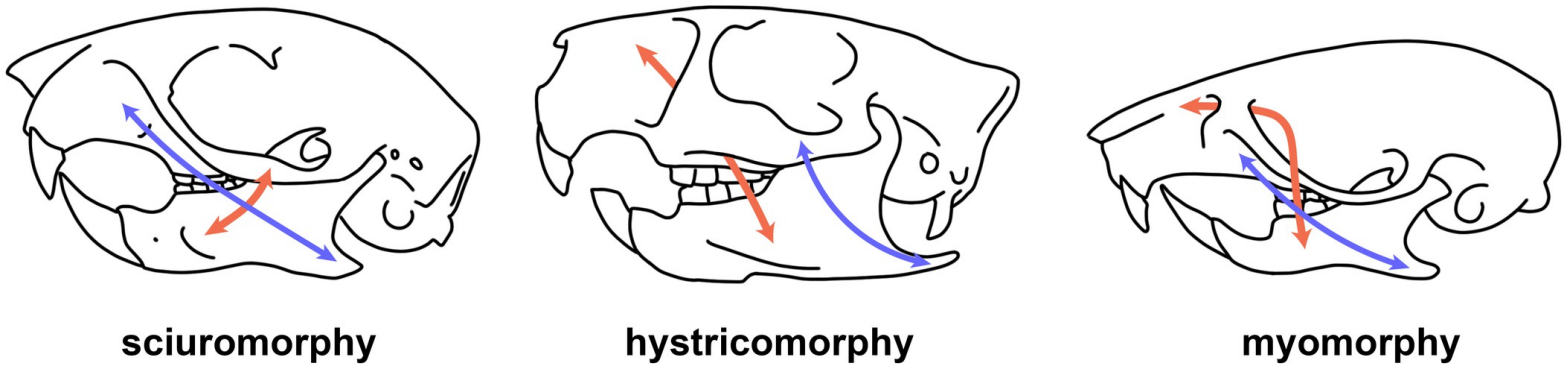
Three types of zygomasseteric architecture of rodent skulls. Arrows show the origin and the insertion of the masseter muscle (medial in red, lateral in blue).

**hystricomorphy**. Type of rodent skull with enlarged infraorbital foramen (see Fig 3).

**incisor groove**. Longitudinal groove running lengthways along the anterior enamel surface of lower and/or upper incisor of several rodent genera. The number of grooves (maximum 3) is often specific to a genus, sometimes to species (e.g., in *Otomys* and *Parotomys*).

**Fig 4:**
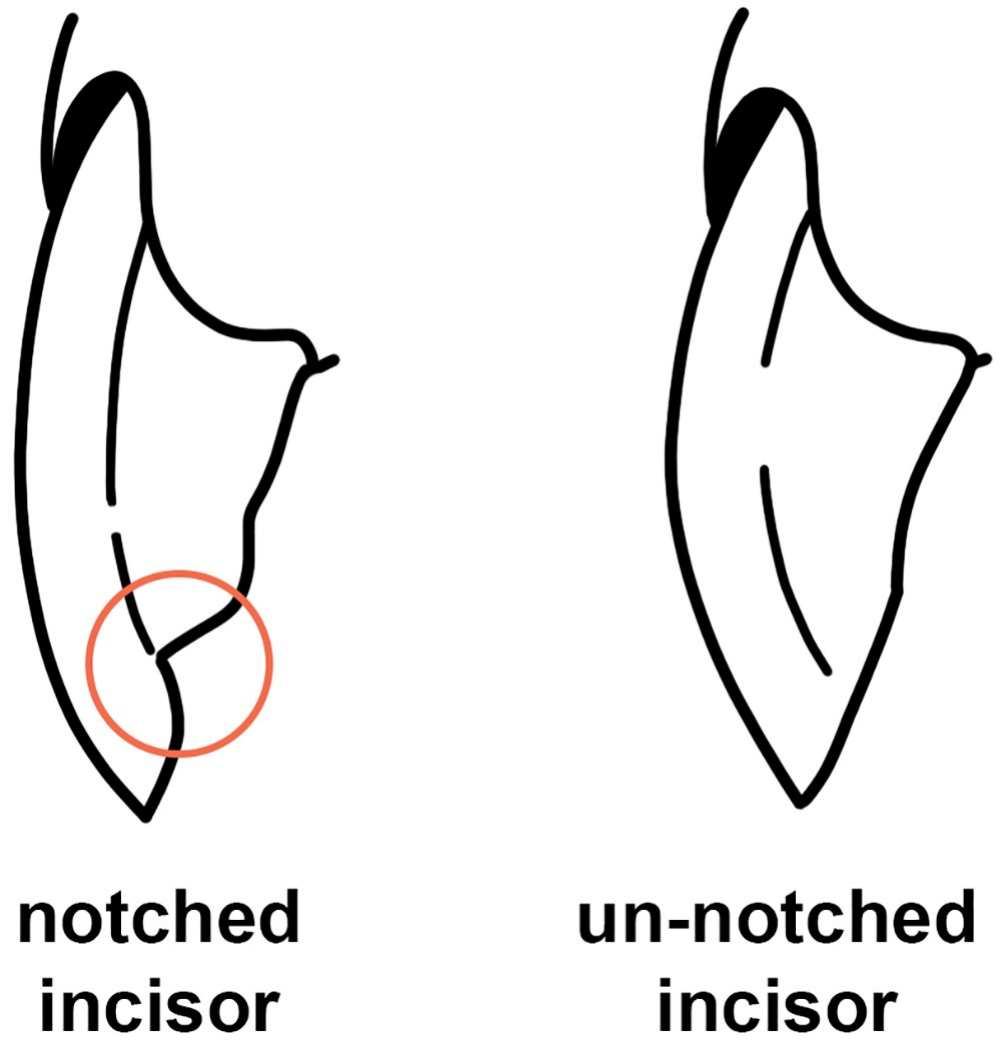
Notched and un-notched left upper incisors in lateral view.

**incisor notch**. Notching on the incisal worn surface of the upper incisors (see Fig 4).

**Fig 5:**
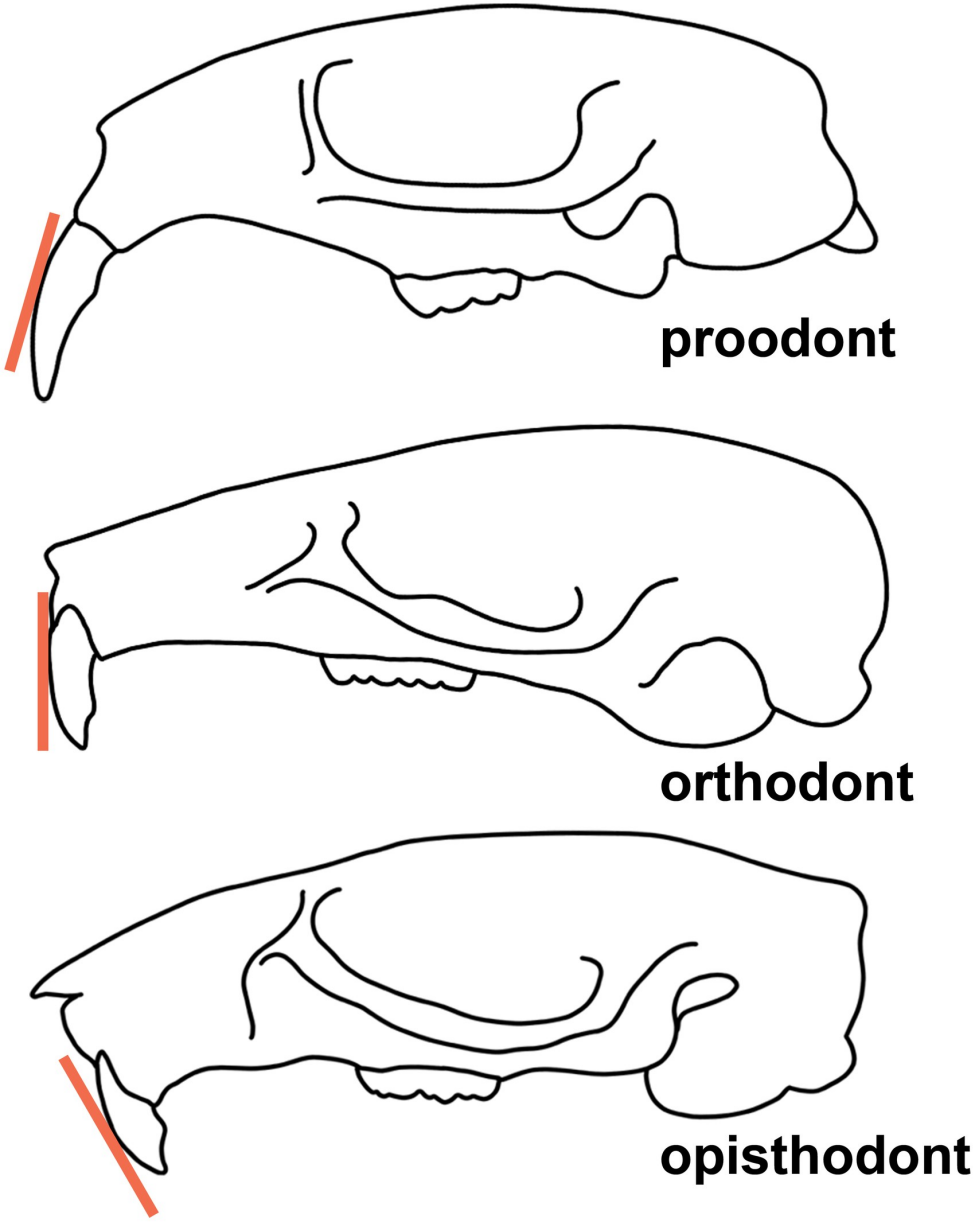
Three different types of orientation of the upper incisor: Proodont (top), orthodont (middle), opisthodont (bottom).

**incisor procumbency**. Orientation of the upper incisors, defined by the position of the cutting edge in relation to the vertical plane of the incisor (see Fig 5).

**lophodonty**. Cheekteeth in which the cusps are fused to form transverse ridges (lophs).

**macrodonty**. Large teeth in proportion to the skull.

**Fig 6:**
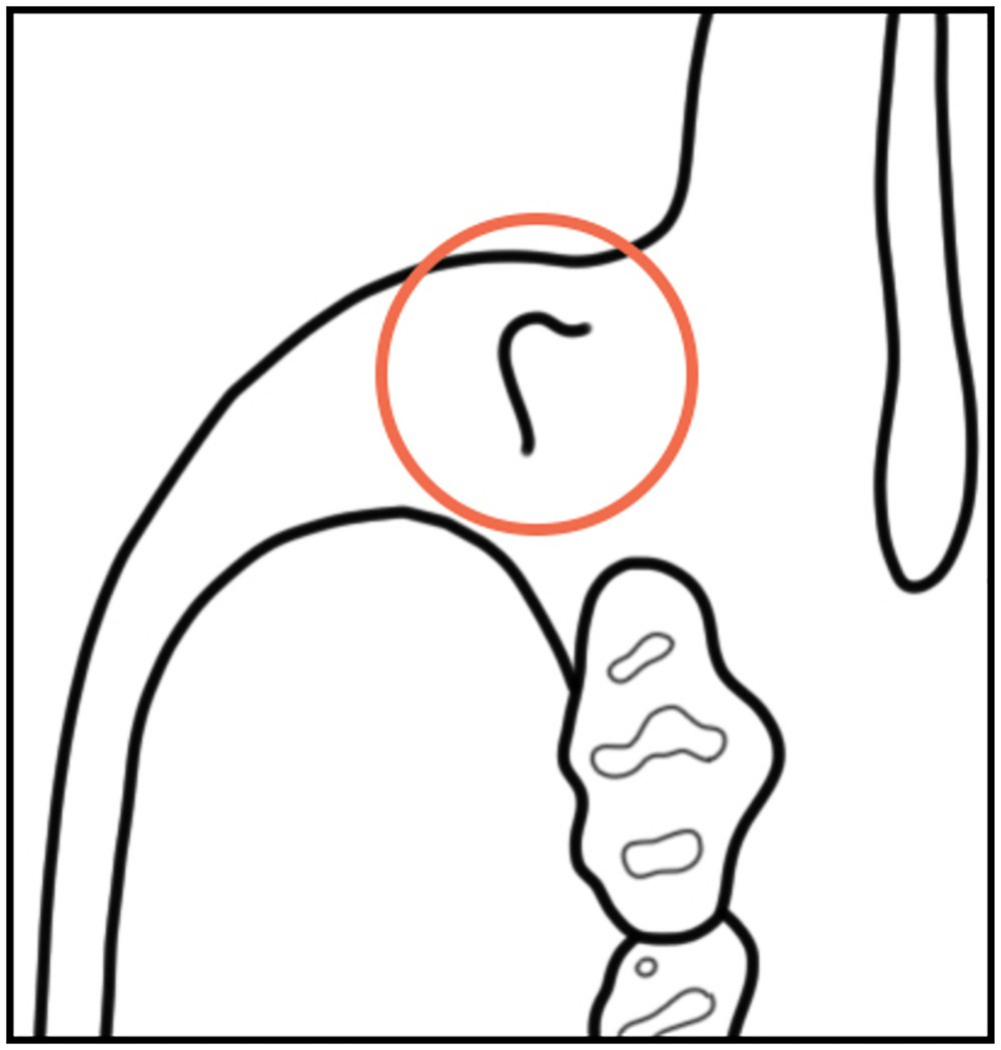
Occlusal view of the upper right toothrow to show the masseter knob, circled in red.

**masseter knob**. Small bony process located close to and below the anterior root of the zygoma (see Fig 6).

**microdonty**. Small teeth in proportion to the skull.

**myomorphy**. Type of rodent skull that combines a large zygomatic plate and a well-developed infraorbital foramen.

**opisthodonty**. Of the incisors, when the cutting extends posterior to the vertical plane (incisors are directed posteriorly; see Fig 5).

**orthodonty**. Of the incisors, when the cutting edge is perpendicular to the plane (incisors are directed more or less vertical plane; see Fig 5).

**palatal foramina**. Orifices in the bony palate for the transmission of palatine vessels and nerves. Anterior palatal foramina (also called incisive foramina) lie between the incisors and the cheekteeth. Posterior palatal foramina are situated between the two rows of cheekteeth.

**posterior cingulum**. Small, rounded structure of enamel on the posterior edge of the molar occlusal surface.

**proodonty**. Of the incisors, when the cutting edge extends anterior to the vertical plane (incisors are directed anteriorly; see Fig 5).

**scalpriform**. Shaped like a chisel.

**sciurognathous**. Condition of the mandible with the angular process in the same plane that the alveolus of incisors (see Fig 2).

**sciuromorphy**. Type of rodent skull with reduced infraorbital foramen and anterior part of the zygomatic arch developed into a large plate (see Fig 3).

**stephanodonty**. Cheekteeth in which ridges are connecting the various cusps in longitudinal rows.

## 4. Material and methods for constructing the key

### 4.1. Material

This key provides a simple step-by-step process for identifying rodents from fossil and owl pellet material in South Africa. For this work we have examined representative specimens from the collections of the Ditsong National Museum of Natural History, Republic of South Africa (DNMNH), Evolutionary Studies Institute of the University of the Witwatersrand, Republic of South Africa (ESI), Muséum national d’Histoire naturelle, France (MNHN), Musée royal de l’Afrique centrale, Belgium (RMCA), and the Institute of Vertebrate Biology, Czech Republic (IVB). List of examined museum specimens with associate catalogue numbers can be found in S1 Checklist. No permits were required for the described study, which complied with all relevant regulations.

This contribution has also relied heavily on information published in other works. For information related to biology, ecology and anatomy of the rodents, the comprehensive works of De Graaff [24], Happold [21], Monadjem et al. [22], and Wilson et al. [12, 13] have been of great value. In addition to these works, a multitude of publications are recommended for supplementing nomenclature and anatomical description of each rodent family; they are explicitly stated in each section through literature citations.

### 4.2. Illustrations

Teeth and skull photographs were taken using Nikon digital camera D 5500 coupled with AF-S Micro NIKKOR 60 mm and macro extension tubes. Photos were stacked using Helicon Focus 8.1.4 and edited in Adobe Photoshop CC 21.1.3. We have tried to show the intrageneric variability in dental anatomy by including photographs of several species in each genus description. Schematics drawings of rodent craniodental morphology, including illustrations of some distinctive features for identification, were realized with Adobe Photoshop CC 21.1.3 and Adobe Illustrator CC 21.0.0. Distribution maps of each species have also been included. These maps are based on distributional data published in Monadjem et al. [22], Wilson et al. [12, 13], data from IUCN (*International Union for Conservation of Nature*) red list database and range maps from various expert sources available on the Map of Life’s website at https://mol.org/ datasets/?dt = range&sg = Mammals [32]. They were designed using R software version 4.1.0.

### 4.3. How to identify families and genera of rodents

The first step of the key selects the name of the family to which a rodent belongs (Tables 2 and 3). For each family, further keys provide readers with a series of statements and two or three choices which will eventually lead to the correct identification of the organism. Some families contain only one genus in South Africa (Gliridae, Hystricidae, Pedetidae, Petromuridae and Thryonomyidae), facilitating easy identification. The keys to the genera (Tables 4 and 5, [32, 33, 43, 44]) are preceded by notes on the habits, preferred habitats, and potential predators, and are followed by a description of each genus and salient morphological characters. It contains dental and alveolar formulas (described below), images of the right upper and right lower molar rows, as well as distribution maps of each species in RSA. The reader may sometimes rely on geographical distributions in distinguishing genera; for example, in the case of *Mastomys* and *Myomyscus*, which are morphologically very close and have a limited overlapping distribution. However, the precision of distribution maps is limited for various reasons, such as species being falsely identified in the field or in collections (also morphologically cryptic species may not be distinguished), lack of knowledge of a taxon’s range, errors introduced in the georeferencing procedure, etc. Furthermore, the range of species and genera have been noted to sometimes change significantly over time [19], so these maps should be used with extreme caution when identifying fossil specimens.

**Table 2:** Key to the rodent families: Upper jaw.

1	3 cheekteeth in adults	2
	4 or 5 cheekteeth in adults	3
2	first lobe of M^1^ with 3 cusps	Muridae*
	first lobe of M^1^ with 1 or 2 cusps	Nesomyidae**
3	occlusal pattern flat and simplified	4
	occlusal surfaces with infolds and/or islands of enamel	5
	occlusal surfaces with cusps and/or transverse ridges	6
4	cheekteeth rooted; incisors markedly proodont; occlusal surfaces simple, either ring or 8-shaped	Bathyergidae
	cheekteeth rootless; incisors opisthodont; teeth bilobed, occlusal surfaces with a single re-entrant fold on the lingual (lower toothrow) or labial (upper toothrow) side of each cheektooth	Pedetidae
5	massive, rounded molars; wavy enamel pattern with multiple crests and islands	Hystricidae
	upper molars have two labial folds and one lingual fold	Thryonomidae
	deep infolds that seem to divide each molar into two separate parts	Petromuridae
6	4 cheekteeth; width of palate about equal to LUTR; toothrow ≤ 4 mm	Gliridae
	4 or 5 cheekteeth; width of palate smaller than LUTR; toothrow > 6 mm	Sciuridae

* Except for the Gerbillinae and Otomyini that have a lophodont dentition

** Except for worn specimens of *Proodontomys* which have a semi-lophodont dentition with flat occlusal surface

**Table 3:** Key to the rodent families: Lower jaw.

1	3 cheekteeth in adults; mandible sciurognath	2
	4 cheekteeth in adults; mandible sciurognath or hystricognath	3
2	first lobe of M_1_ with 2 cusps (with possible presence of an additional tma)	Muridae*
	first lobe of M_1_ with 1 cusp (or two poorly differentiated in some *Saccostomus*)	Nesomyidae**
3	occlusal pattern flat and simplified	4
	occlusal surfaces with infolds and/or islands of enamel	5
	occlusal surfaces with cusps and/or transverse ridges	6
4	cheekteeth rooted; occlusal surfaces simple, either ring or 8-shaped; mandible hystricognath	Bathyergidae
	cheekteeth rootless; teeth bilobed, occlusal surfaces with a single re-entrant fold on the lingual (lower toothrow) or labial (upper toothrow) side of each cheektooth; mandible sciurognath	Pedetidae
5	massive, rounded molars; wavy enamel pattern with multiple crests and islands	Hystricidae
	lower molars have two lingual folds and one labial fold	Thryonomidae
	deep infolds that seem to divide each molar into two separate parts	Petromuridae
6	smaller: toothrow < 4 mm	Gliridae
	larger: toothrow ≥ 7 mm	Sciuridae

* Except for the Gerbillinae and Otomyini that have a lophodont dentition

** Some Saccostomus display two poorly differentiated on first lobe of M_1_

**Table 4:** Key to the murid and nesomyid genera: Upper jaw.

1	tooth are laminate; M^3^ is the largest molar	2 (*Otomys* or *Parotomys*)
	tooth are semi-laminate (lamelliform/buno-lophodont cusps); M^1^ is the largest molar	3
	tooth are not laminate and have well-defined cusps; M^1^ is the largest molar	6
2	bullae enlarged; M^3^ has two or three complete laminae; upper incisors grooved or ungrooved	*Parotomys*
	bullae less inflated; M^3^ has four or more laminae; upper incisors have one or more grooves	*Otomys*
3	lamelliforms cusps in M^1^ and M^2^ lack longitudinal connections	4
	lamelliforms cusps in M^1^ and M^2^ are connected longitudinally in their central region; not found in modern material (last occurrence around 1 MYA)	*†Proodontomys*
4	larger: LUTR ≥ 5.5 mm and WM^1^ ≥ 2 mm	*Gerbilliscus* (*Gerbilliscus*)
	smaller: LUTR ≤ 5 mm and WM^1^ < 2mm	5
5	M^1^ has three roots; M^3^ has one lobe (or two poorly separated when unworn); bullae are very large proportionate to the skull	*Desmodillus*
	M^1^ has four roots; M^3^ has one or two lobes; bullae are smaller	*Gerbilliscus* (*Gerbillurus*)
6	first lobe of M^1^ has one or two cusps	7
	first lobe of M^1^ has three cusps	13
7	skull is large and LUTR > 8 mm	*Cricetomys*
	skull is smaller and LUTR < 8 mm	8
8	cusps of M^1^ are arranged in a zigzag enamel pattern connected by a median longitudinal crest running the length of the tooth	9
	no median longitudinal crest in M^1^	10
9	LUTR < 5 mm	*Petromyscus*
	LUTR > 6 mm	*Mystromys*
10	M^3^ has two lobes; cusps have a bulbous aspect; masseter knob absent	*Saccostomus*
	M^3^ is reduced and has one lobe; molars show typical Dendromurinae pattern; masseter knob present	11
11	palate extends far beyond M^3^; M^1^ is very long, occupying half of the length of the toothrow; masseter knob ridge-shaped	*Malacothrix*
	palate ends close after M^3^; M^1^ is shorter proportionate to the toothrow; masseter knob not ridge-shaped	12
12	molars slightly larger; M^1^ 3-rooted	*Steatomys*
	molars slightly smaller; M^1^ 4-rooted	*Dendromus*
13	small size: LUTR < 4.2 mm	14
	medium or large size: LUTR > 4.2 mm	15
14	M^3^ has a t3; masseter knob absent or reduced; palate extends far backwards	*Acomys*
	M^3^ has no t3; conspicuous masseter knob; palate ends closer to the toothrow	*Mus*
15	pronounced stephanodonty; M^3^ belongs to Group 6 in Fig 12	16
	no or incomplete stephanodonty; M^3^ belongs to Group 3, 4 or 5 in Fig 12	17
16	smaller: mean LUTR ≤ 5 mm; t3 reduced or absent in M^2^, which has four alveoli; large bullae (diameter usually > 6mm)	*Grammomys*
	larger: mean LUTR ≥ 5 mm; t3 present in M^2^, which has five alveoli; smaller bullae (diameter usually < 6mm)	*Thallomys*
17	macrodonty; rows of cusps arranged in transverse rows with tendency to isolate and become laminate with wear	*Dasymys*
	molars of small or average size; rows of cusps showing slight or pronounced distortion	18
18	well-developed t9 projecting outwards, giving the impression that the M^1^ is leaning obliquely	19
	t9 of small or average size, with M^1^ positioned straight in the anteroposterior axis	22
19	M^1^ 3-rooted; shorter toothrow; palatal foramina penetrating between the molars	20
	M^1^ 5-rooted; longer toothrow; palate foramina stop at or just short of the root of M^1^	*Rattus*
20	larger: mean LUTR = 5.3 mm; M^3^ belongs to Group 5 in Fig 12; strong incisors	*Zelotomys*
	smaller: mean LUTR = 4.8 mm; M^3^ belongs to Group 3 in Fig 12; incisors less thick	21
21	posterior palatal foramina generally set between posterior part of M^2^; wide distribution	*Mastomys*
	posterior palatal foramina generally set between anterior part of M^2^; restricted distribution	*Myomyscus*
22	smaller: LUTR usually < 5mm; in the M^1^ the two distal rows of cusps appear to be linked on the labial and lingual sides; in both M^1^ and M^2^ the t9 is reduced to a small ridge	*Rhabdomys*
	larger: LUTR usually > 5mm; in the M^1^ the two distal rows of cusps may be linked or not on the labial and lingual sides; t9 not reduced	23
23	LUTR ≈ length of the upper palatal foramina; M^1^ has four alveoli; distal lobe on M^3^ has one or two poorly differentiated cusps	24
	LUTR < length of the upper palatal foramina; M^1^ has five alveoli; distal lobe on M^3^ has two differentiated cusps	*Lemniscomys*
24	molars slightly smaller; t1 is rather aligned with t2 and t3	*Micaelamys*
	molars slightly larger; t1 is usually behind t2 and t3	*Aethomys*

**Table 5:** Key to the murid and nesomyid genera: Lower jaw.

1	tooth are laminate	2
	tooth are semi-laminate (lamelliform cusps)	3
	tooth have well defined cusps	6
2	lower incisors grooved (one or more grooves)	*Otomys*
	lower incisors ungrooved	*Parotomys*
3	lamelliforms cusps in M_1_ and M_2_ lack longitudinal connections	4
	lamelliforms cusps in M_1_ and M_2_ are connected longitudinally in their central region; not found in modern material (last occurrence around 1 MYA)	*†Proodontomys*
4	cusps in second row of M_1_ fused in a transverse lamina	*Gerbilliscus (Gerbilliscus)*
	cusps in second row of M_1_ unfused	5
5	M_1_ has two alveoli	*Desmodillus*
	M_1_ has four alveoli	*Gerbilliscus (Gerbillurus)*
6	cusps in M_1_ are fused by a median longitudinal crest running the length of the tooth and arranged in a zigzag enamel pattern	7
	cusps show no median longitudinal crest	8
7	small: LLTR < 4 mm; lower incisors smooth	*Petromyscus*
	larger: LLTR > 5 mm; characteristic enamel band on lower incisors	*Mystromys*
8	mandible is very large (length between incisor alveolus and condylar process > 4 cm), with a wide, elongated alveolar region; restricted to the Limpopo province; LLTR > 9,5mm	*Cricetomys*
	mandible is smaller; LLTR < 9,5mm	9
9	first lobe of the M_1_ has a single median anterior cusp; muscle attachment on the mandible is not right next to the mental foramen	10
	first lobe of the M_1_ has a two or three cusps; position of the muscle attachment in relation to the mental foramen variable	13
10	cups in M_1_ not alternated; lower incisors display small raised band of enamel	*Saccostomus*
	typical Dendromurinae pattern with alternated cusps in M_1_; lower incisors smooth	11
11	M_1_ is very long, occupying more than half of the length of the toothrow	*Malacothrix*
	M_1_ is shorter proportionate to the toothrow	12
12	LLTR ≤ 3 mm; hd and ed not fused in M_1_	*Dendromus*
	LLTR ≥ 3 mm; hd and ed fused in M_1_	*Steatomys*
13	broad molars (macrodonty) with WM_1_ ≥ 2 mm; first lobe of M_1_ has three cusps; M_1_ has no clear pc	*Dasymys*
	WM_1_ < 2 m; first lobe of M_1_ has two to three cusps; presence of a pc in M_1_ variable	14
14	LLTR < 3.8 mm	15
	LLTR > 3.8 mm	16
15	M_1_ has a typically enlarged alg; M_3_ has one or two lobes; well-developed coronoid process	*Mus*
	alg and alb roughly the same size; M_3_ has two lobes; poorly developed coronoid process	*Acomys*
16	M_1_ has a well-developed anteromedian cusp	17
	M_1_ has a poorly developed or no anteromedian cusp	18
17	M_1_ has a pc and a stephanodont crest	*Grammomys*
	M_1_ has no pc and no stephanodont crest.	*Micaelamys*
18	conspicuous stephanodont crest in both M_1_ & M_2_	*Thallomys*
	no well-marked stephanodont crest in both M_1_ & M_2_	19
19	M_1_, M_2_ and M_3_ with two main roots	20
	M_1_, M_2_ and M_3_ with three or more roots	22
20	strong incisor; LLTR ± 5.1 mm	*Zelotomys*
	incisor of average size; LLTR ± 4.5 mm	21
21	wide distribution; mandible and teeth length slightly larger on average	*Mastomys*
	restricted distribution; mandible and teeth length slightly smaller on average	*Myomyscus*
22	much smaller: LLTR < 5mm	*Rhabdomys*
	larger: LLTR > 5 mm	*23*
23	M_1_ and M_2_ have a well-developed pc; molars proportionally small in relation to the mandible	*Rattus*
	M_1_ often has no pc; molars of average size in relation to the mandible	24
24	the two most posterior roots of M_1_ are fused; M_2_ has three alveoli; additional cusplets often occur on labial side of M_1_ and M_2_	*Aethomys*
	the two most posterior roots of M_1_ are unfused; M_2_ has six alveoli (presence of two rootlets); lateral cusplets less marked	*Lemniscomys*

We tried to use mainly discriminating criteria, which correspond to diagnostic characters of a genus and are present in all the individuals. As discriminating criteria are not always available in cranio-dental morphology for some taxa, or for broken specimens within the fossil or pellet material, we also propose secondary criteria, *i*.*e*. character states that are not absolute in terms of identifying to genus. Secondary criteria are sometimes absent in taxa that exhibit great intraspecific variation in size and shape, or may be subject to subjective interpretation. The presence of several secondary criteria can lead to the confident identification of a taxon, but identification must be based on as many characters as possible. We specify in the key when a listed character is likely to display variability.

A modified version of the key can be accessed online with Xper^3^, a free collaborative platform designed for computer-aided taxonomic description and identification [33, 34], at: https://rodentsouthafrica.identificationkey.org. The online key will be updated over time and integrate additional photographs for modern and fossil specimens.

We draw attention to the fact that the criteria used in this key are for South African taxa only and may not be applicable extralimitally. Furthermore, in an archaeological and palaeontological context, many specimens have been fragmented or damaged by taphonomic processes. Methods of genetic identification are not always feasible, and taxonomy based on geometric morphometrics is time-consuming and requires a certain amount of expertise. In such situations, the wisest attitude is to limit identification at a higher taxonomic determination level such as the family; it is inevitable that some specimens will be excluded from the fossil record.

#### 4.3.1. Morphological characters

In rodent jaws, the most important morphological character for genus identification is the tooth cusp pattern. Other diagnostic characters for the upper jaw include: number of cheekteeth, incisor morphology, location of the primary and secondary palatal foramina, origination of the zygomatic, presence of a masseter knob, etc., and for the lower jaw: location of the mental foramen, muscle attachment, shape of the mandible, projection of the mental, coronoid and condylar processes, etc. The number of tooth types is written as a dental formula, with the upper and lower teeth shown consecutively. Incisors (I) are indicated first, canines (C) second, premolars (P) third, and molars (M) fourth, giving the formula: I-C-P-M:I-C-P-M.

As loss of teeth is frequent in predator-derived assemblages, alveolar pattern also provides identification information for several taxa. Avery [6] published a useful key (which relies mostly on the size and number of alveoli) to distinguish mandibles of Wonderwerk micromammals in the absence of diagnostic teeth. We use here a similar alveolar-molar root formula to indicate the number of alveoli of the various Muridae and Nesomyidae genera, but here the small, round alveoli resulting from rootlets are counted as independent alveoli. The alveolar formula provides the number of alveoli of each cheektooth and is written in a similar way to dental formula, with upper and lower alveolar patterns consecutively. Thus, the formula 4-3-3:3-2/3-2 means that the upper molars M^1,^ M^2^ and M^3^ have four, three, and three alveoli respectively, and the lower molars M_1_, M_2_ and M_3_ have three, two or three, and two alveoli respectively. There may be variability in the number of roots and rootlets of some taxa, so indication of the alveoli should be used as a guiding, but not absolute, criterion.

#### 4.3.2. Size and measurements

**Fig 7:**
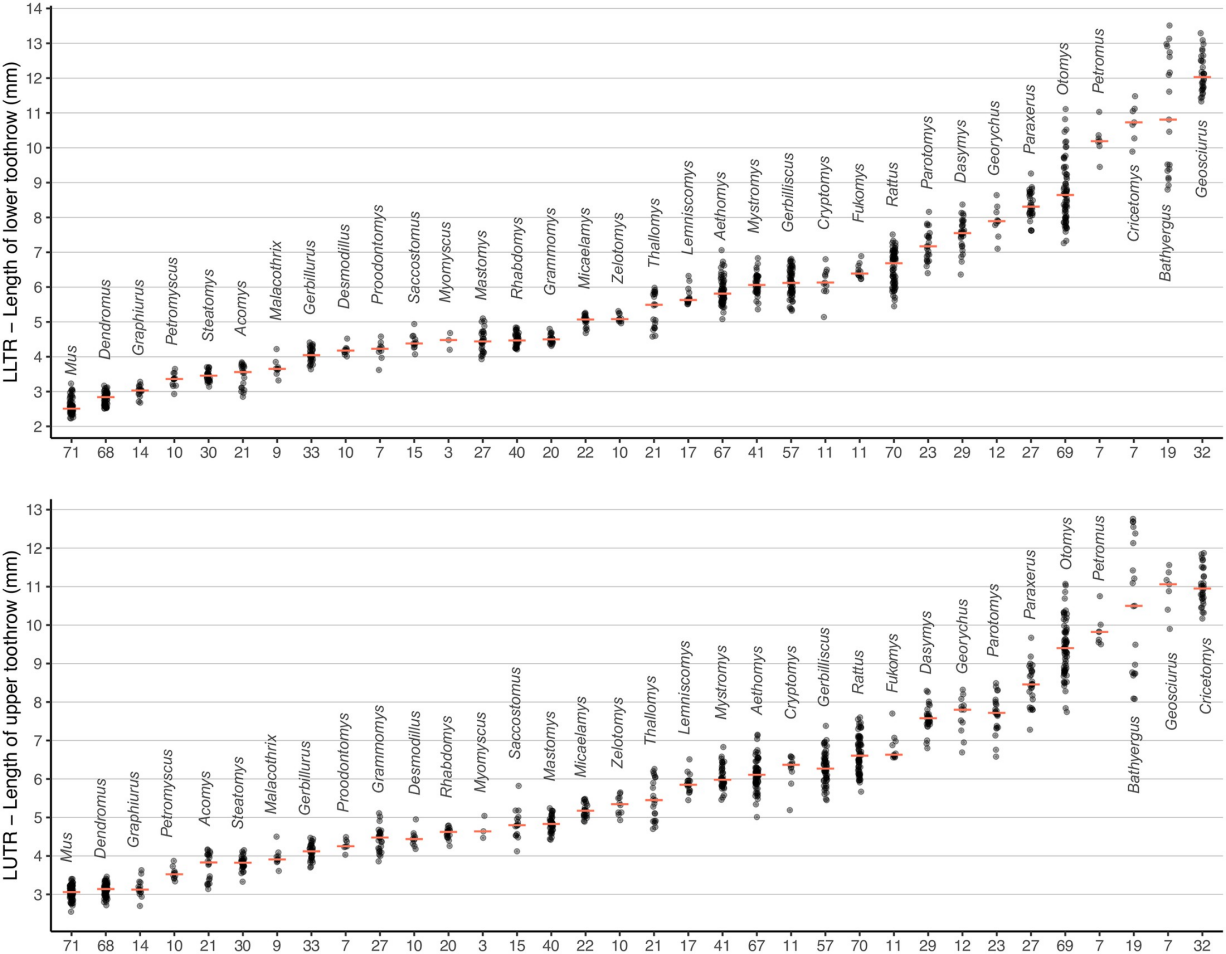
Length of lower toothrow (LLTR) and upper toothrow (LUTR) for rodent genera from RSA. Red dashes represent medians. Numbers under the x-axis represent the number of specimens used for the measurements. The following genera were not included because both LUTR and LLTR are much greater: *Pedetes*, *Thryonomys*, and *Hystrix* (see measurement details in genus accounts).

**Fig 8:**
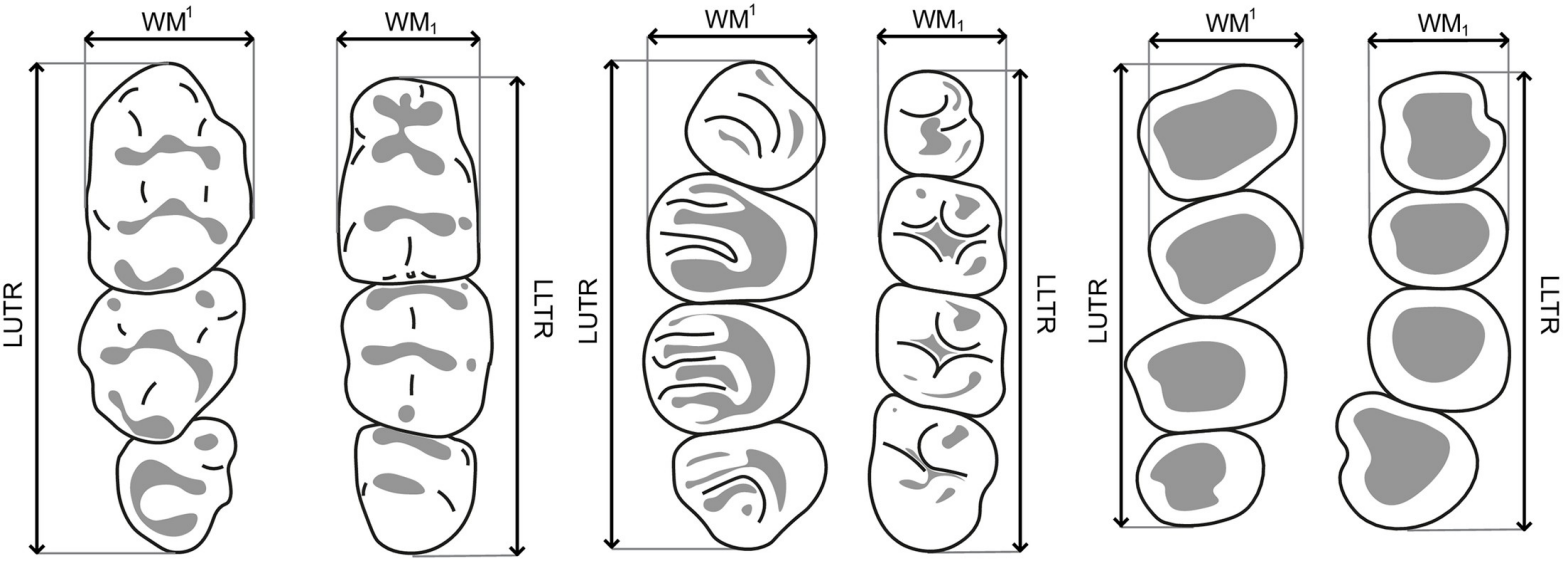
Definition of the measurements used in this key for upper toothrow and lower toothrow of typical Murinae (left), Sciuridae (center) and Bathyergidae (right). LUTR: length of the upper toothrow; WM1: width of the first upper molar; LLTR: length of the lower toothrow; WM1: width of the first lower molar.

There is a great variation in size in South African rodents: the smallest (*Mus indutus*) weighs only 3 to 5 g and the largest (*Hystrix africaeaustralis*) weighs about 20 kg. The same is true for the size of the cheekteeth, and measurements of length and width can assist in identification at genus level. Fig 7 below presents measurements of the length of the upper toothrow (LUTR) and the length of the lower toothrow (LLTR) of each genus from modern museum collections (except for the extinct *Proodontomys* whose measurements were taken on fossils):

We use four types of measurements in this key: LUTR = length of the upper toothrow (including molars and premolar(s) if present); WM^1^ = width of the first upper molar; LLTR = length of the lower toothrow; WM_1_ = width of the first lower molar. All measurements were taken with a digital calliper to the nearest 0.01 mm. The length and width of the teeth correspond to the maximum values along the mesiodistal and labiolingual axes of the teeth on the basis of the crown, as illustrated in Fig 8:

Although these measurements may be useful for preliminary identification, they must not be considered absolute as some taxa may present biogeographic or temporal variations in size [9].

#### 4.3.3. Comments on the age of the specimens

**Fig 9:**
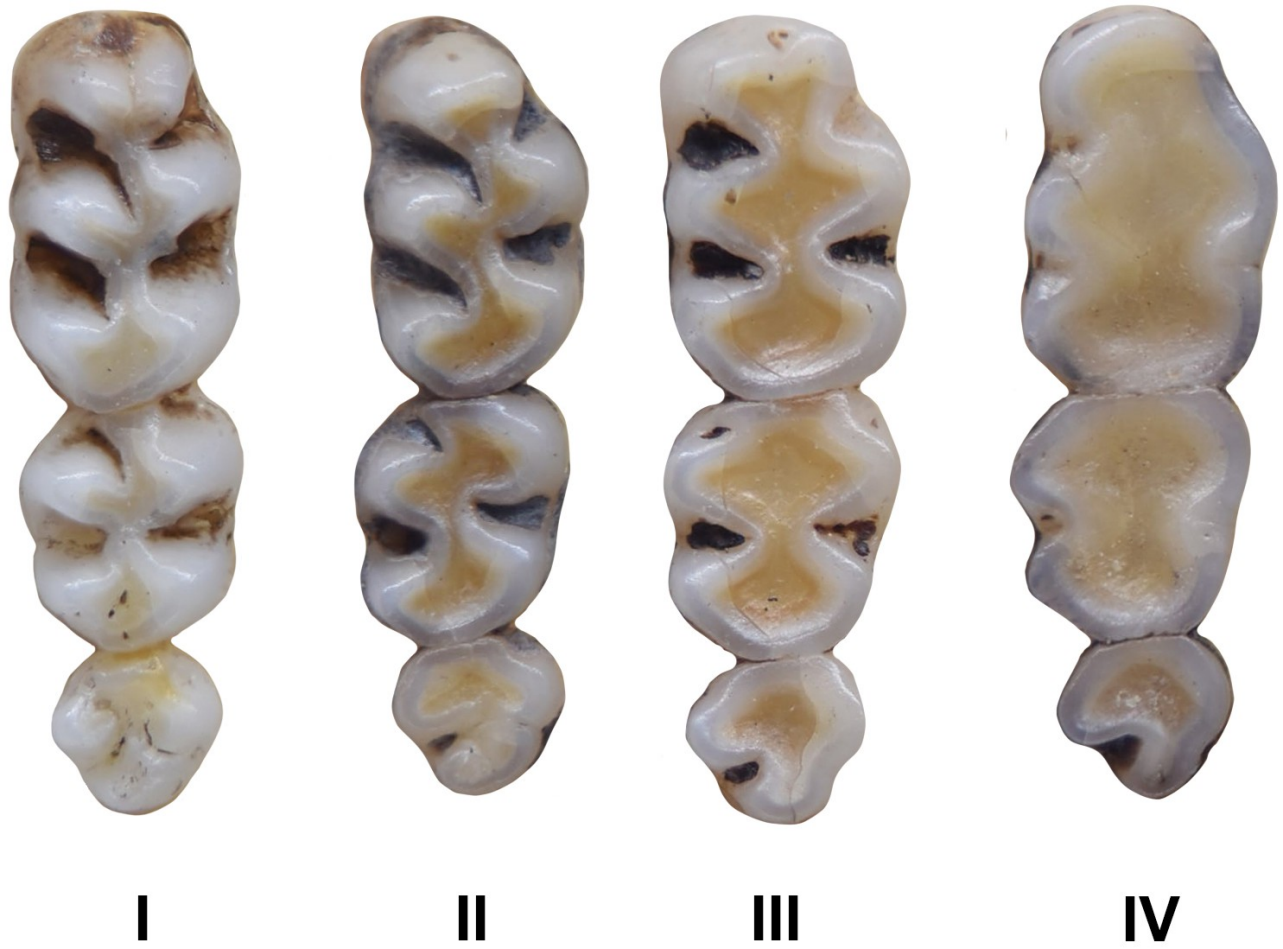
Various dental wear stages of right upper toothrows in the same species *Mystromys albicaudatus*, ranging from no wear (stage 1, juvenile specimen) to advanced wear with dentine exposed (stage 4, old individual).

This key is mostly intended for adult and sub-adult specimens, as juvenile or old specimens may not always key out correctly. In juvenile specimens, the size and eruption of the teeth may differ. This is especially true for larger species, which have a slower growth rate than smaller species and therefore reach maturity after a longer period. For instance, in *Rhabdomys pumilio* (mean weight = 45 g) the M^1^ and M^2^ starts erupting at the age of two weeks, while the M^3^ begins to erupt by four to five weeks of age [35]. In *Hystrix africaeaustralis* (weight up to 20 kg), deciduous premolars begin erupting at about 14 days of age, the M^1^ between 2 to 3 months, the M^2^ between five and six months, and the M^3^ at about 11 months; the permanent premolars are fully erupted at the age of two years [21]. The age at which species reach sexual maturity is also of concern, since most rodents are nidifugous (i.e., they leave the nest shortly after hatching or birth) and will leave the family nest and disperse once they are sexually mature. For instance, the Cairo spiny mice, *Acomys cahirinus*, become sexually mature and leaves the nest at the age of 2–3 months [36]. In *Cryptomys hottentotus*, the pups remain in the maternal burrow system for about 60 days until they are expelled from the burrow by the mother [36]. In *Otomys sloggetti*, males reach sexual maturity in 11 weeks, and females in 16 weeks, before dispersing [37] while subadult males of *Geosciurus inauris* do not disperse until eight months of age [38]. In older specimens, wear on the occlusal surface, which results in the removal of enamel and dentine, may obscure or obliterate the cusp pattern, and previously separate cusps may become fused. At a most advanced stage of wear, molars can be reduced to flattened, ovoid lobes with exposed dentine (Fig 9). At this stage, it is often more prudent to limit identification to the level of the family or subfamily.

#### 4.3.4. Effects of digestion on tooth morphology

**Fig 10:**
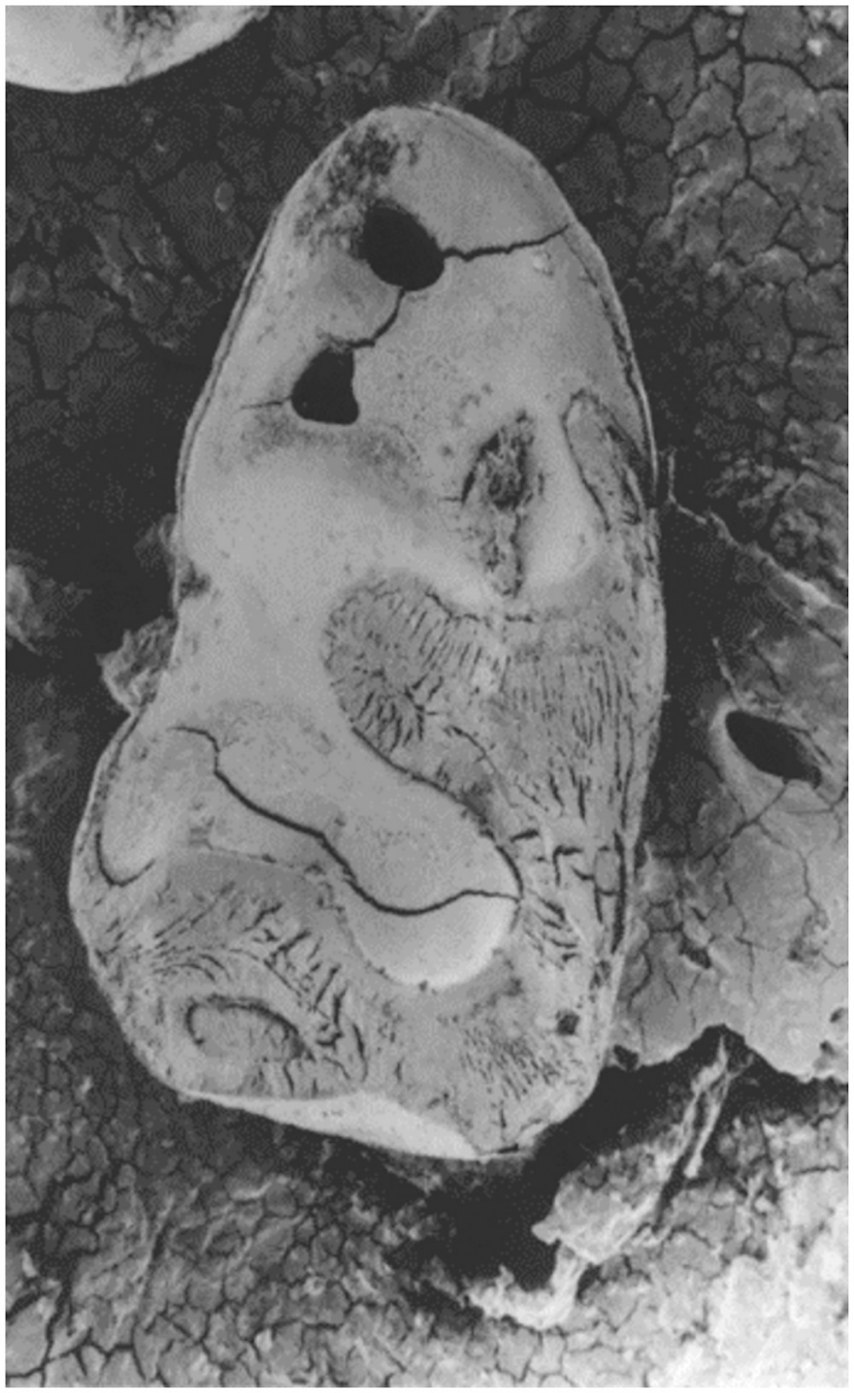
Scanning electron micrograph of a murid first lower molar showing heavy digestion. Enamel is locally removed, dentine is also affected.

The passage through the digestive tract of predators can cause corrosive damage to the teeth, which in turn may sometimes cause difficulties in taxonomic identification. Numerous works have been dedicated to categorizing predators into distinct categories based on their digestion patterns [16, 18, 39, 40]. Predators that cause heavy or extreme digestion modifications, such as diurnal raptors or small carnivores, can alter the enamel outline of molars, resulting in some cusps or other features of diagnostic importance almost eaten away or completely missing (Fig 10).

## 5. Key to the families and genera of rodents in South Africa

The keys presented below for upper and lower jaws provides the main identification criteria to identify specimens to family level.

### Muridae & Nesomyidae

**Fig 11:**
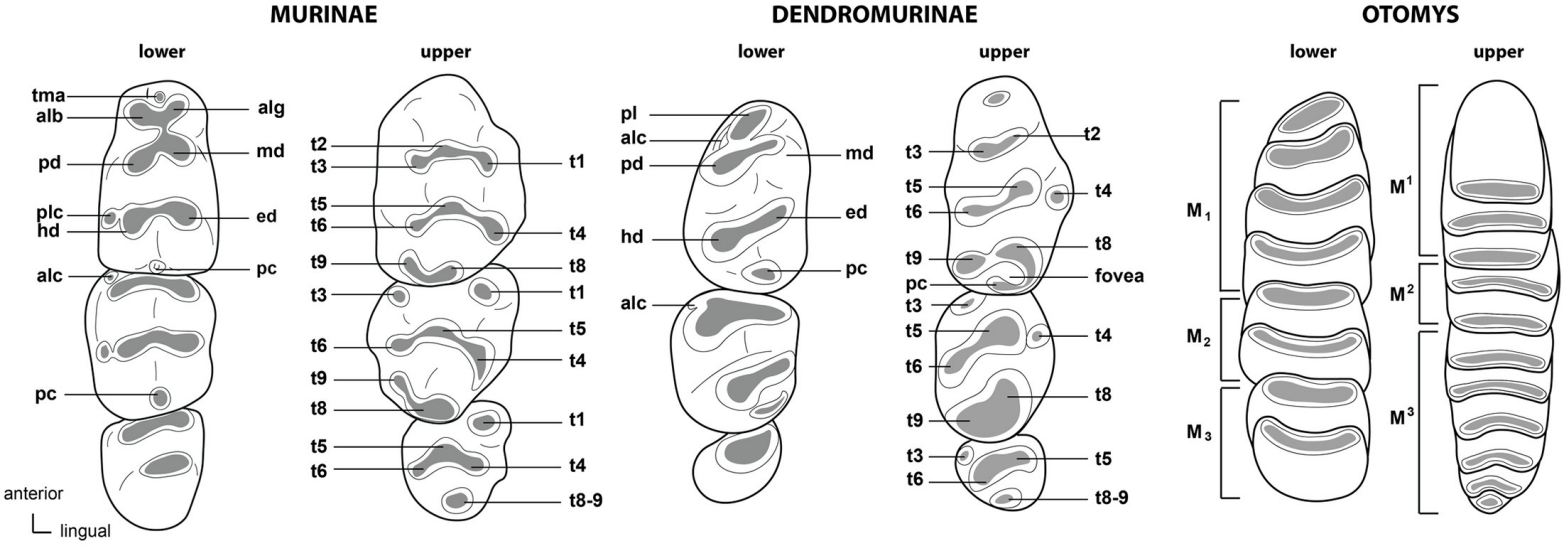
Typical Murinae, Dendromurinae, and Otomys left lower toothrows and right upper toothrows, with nomenclature of the cusps. Lower toothrow: alb: labial anteroconid; alc: anterolabial cingulum; alg: lingual anteroconid; hd: hypoconid; md: metaconid; pd: protoconid; pc: posterior cingulum; pl: prelobe; plc: posterolabial cusplet; tma: anteromedian cusp. Upper toothrow: t1: anterostyle; t2: lingual anterocone; t3: labial anterocone; t4: anterostyle; t5: protocone; t6: paracone; t8: pseudohypocone; t9: metacone (modified from Denys et al., 1992).

**Fig 12:**
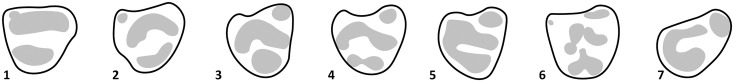
Shape and configuration of the right upper M^3^ of the Muridae and Nesomyidae (after Denys & Michaux [41], reproduced in Monadjem et al. [22]). 1) t3 present, t1 absent, t3 connected to the first row of cusps: *Cricetomys*, *Dendromus*, *Malacothrix*, *Petromyscus*, *Saccostomus*, *Steatomys = Nesomyidae*; 2) t3 present, t1 absent, t3 isolated: *Acomys = Deomyinae*; 3) t1 present, a distal cusp, t3 absent: some *Aethomys*, *Mastomys*, some *Micaelamys*, *Myomyscus*, *Rattus*, *Rhabdomys*; 4) t1 present, t3 absent, second lobe with 2 fused or distinct distal cusps: some *Aethomys*, *Dasymys*, *Lemniscomys*, some *Micaelamys*; 5) rather large t1, labial link between first lobe and second lobe: *Zelotomys*; 6) presence of t1 and tiny t3, trace of median longitudinal link: *Grammomys*, *Thallomys*; 7) small molars with t1 and link between first and second lobe, cusps poorly differentiated: *Mus*.

Following Wilson et al. [12, 13], the family Muridae includes three subfamilies in RSA (Deomyinae, Gerbillinae, Murinae) and the family Nesomyidae includes four subfamilies (Cricetomyinae, Dendromurinae, Mystromyinae, Petromyscinae). Each subfamily of the Nesomyidae is morphologically well characterized, but there are no known morphological dental features that distinguish the family itself from the Muridae. We have therefore grouped the two families together. We use cusp nomenclature from Misonne [26] and Denys et al. [27] for describing the cusps of the lower and upper molars of Murinae and Dendromurinae:

In Muridae and Nesomyidae, the upper molars are usually the most diagnostic teeth. Denys & Michaux [41] grouped some African genera by the structure of the third upper molar; these structures are presented in Fig 11 for genera from South Africa. Unfortunately, skulls are more prone to fragmentation than mandibles, which explains why they are generally proportionately less well-represented in archaeological and paleontological assemblages. In the absence of teeth, the number and structure of alveoli, as well as the length of the anterior palatal foramina, provide useful criteria for identifying Muridae and Nesomyidae taxa (Fig 12).

Family **MURIDAE** Illiger, 1811

Subfamily **DEOMYINAE** Thomas, 1888

Genus ***Acomys*** I. Geoffroy, 1838 (Spiny Mice)

Figs 13–15; Table 6

**Fig 13:**
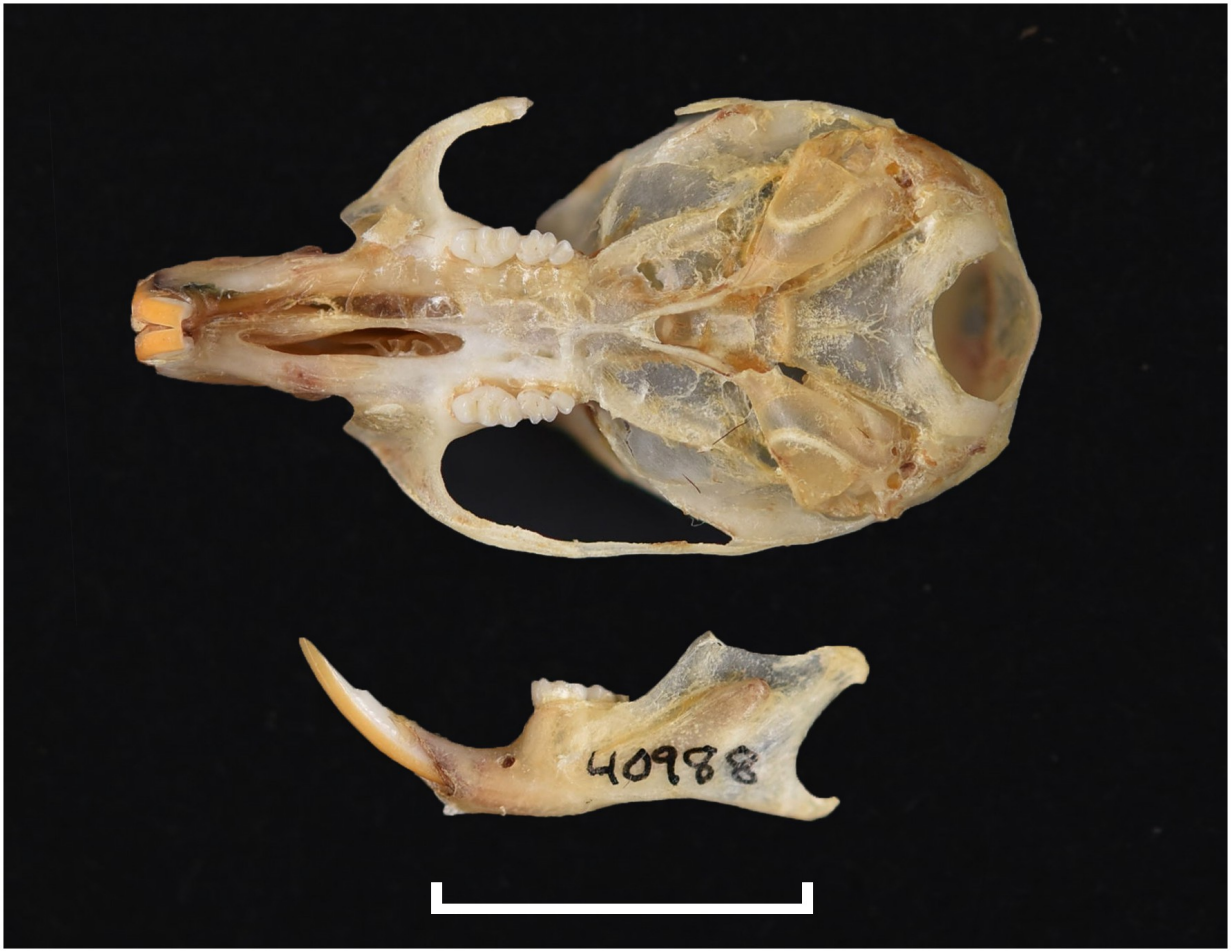
Cranium of *Acomys subspinosus* (DNMN-40988), with a scale bar of 1 cm.

**Fig 14:**
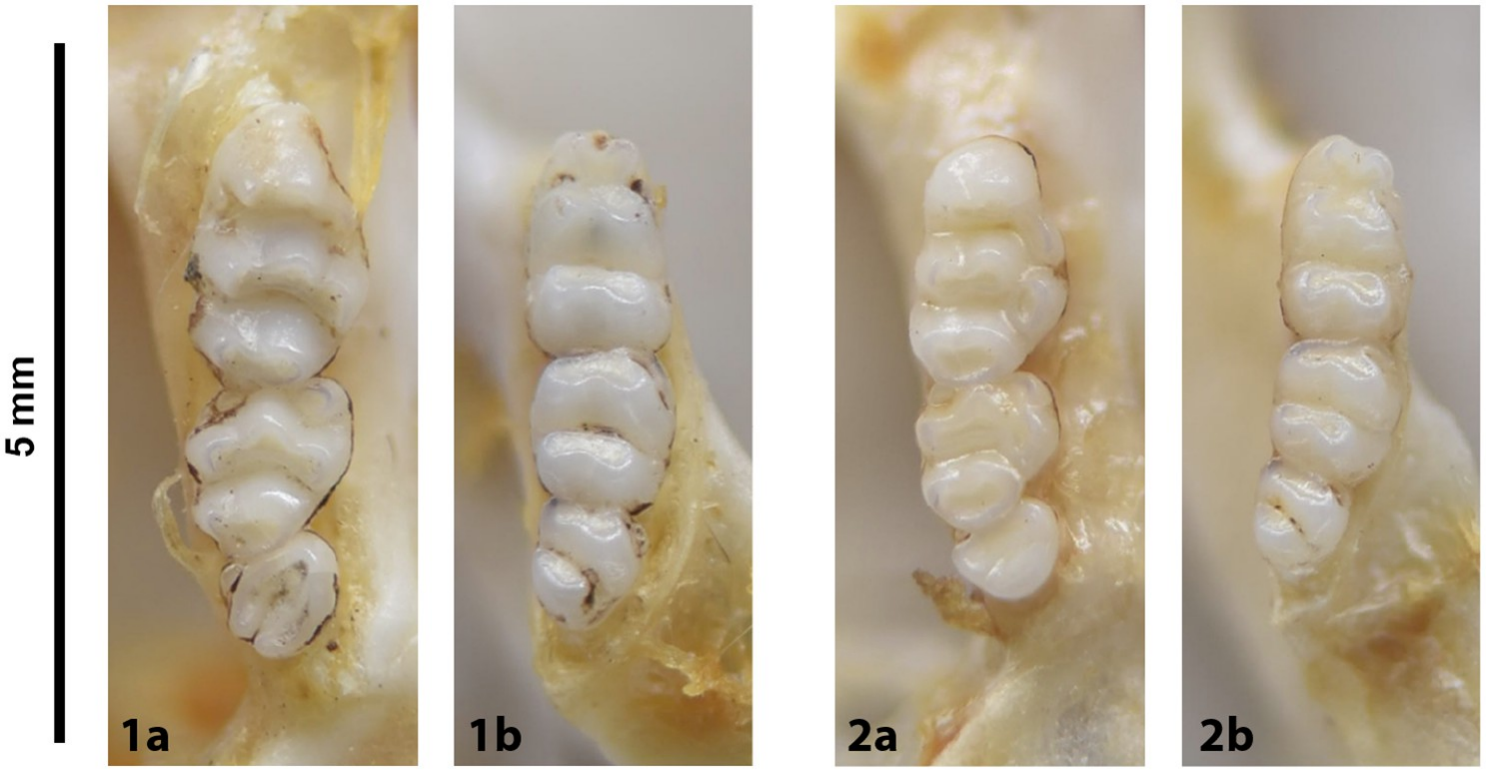
Cheekteeth of *Acomys*. **1)** Upper (a) and lower (b) right toothrow of *A*. *selousi* (DNMNH-2833); **2)** Upper (a) and lower (b) right toothrow of *A*. *subspinosus* (DNMNH-40989).

**Fig 15:**
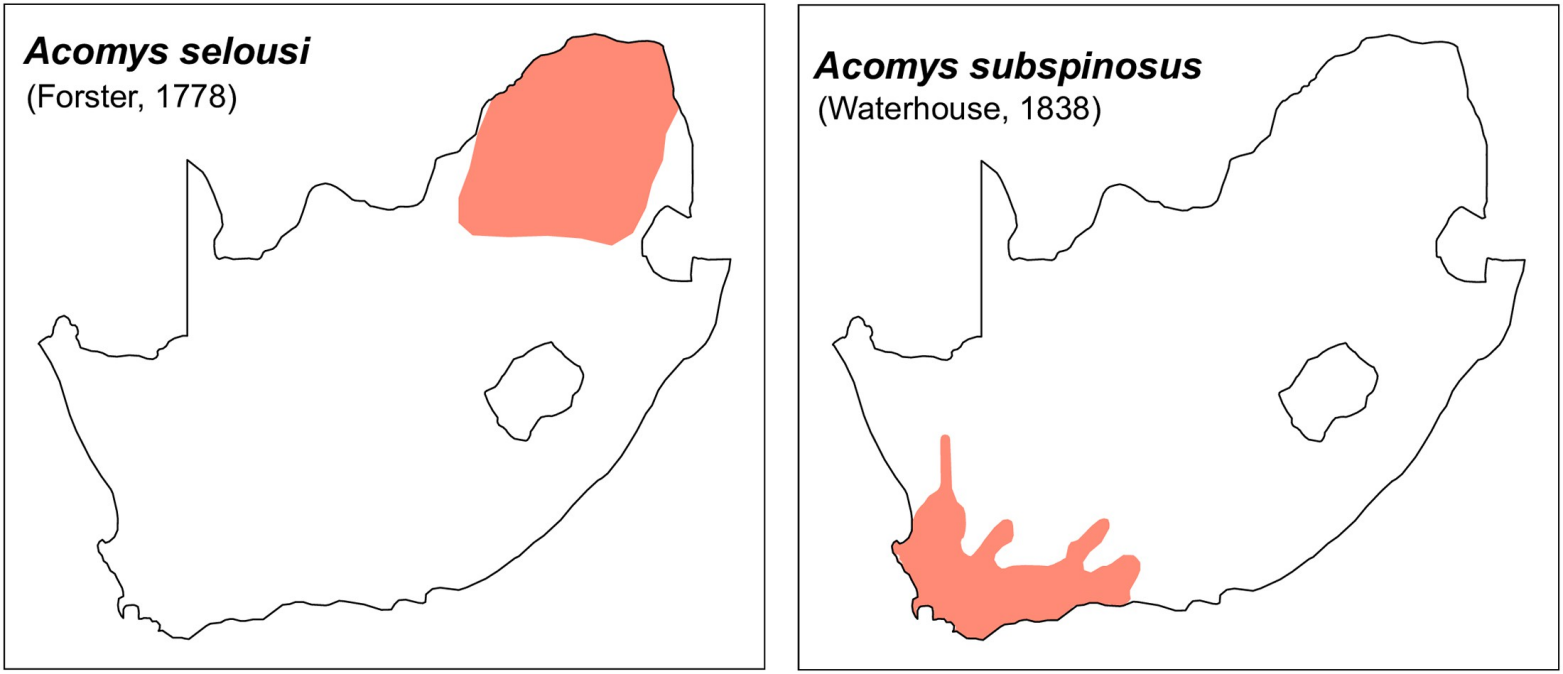
Distribution maps.

**Table 6:** Dental measurements (in mm) for *Acomys* from South Africa, sexes and species combined.

	Mean	Min	Max	n
LLTR	3.5	2.9	3.8	20
WM_1_	1.0	0.8	1.1	20
LUTR	3.8	3.1	4.2	21
WM^1^	1.2	1.0	1.4	21

**Fig 16:**
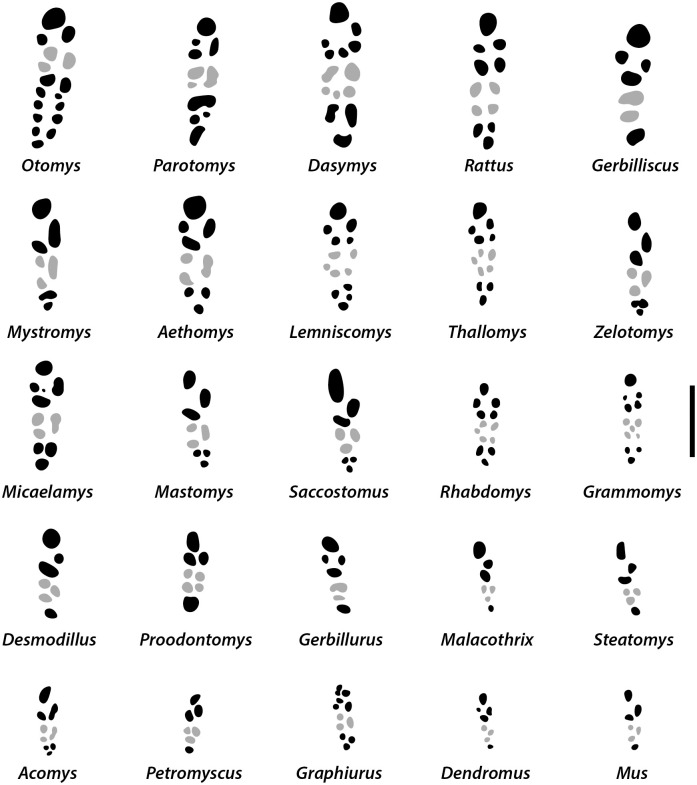
Right maxilla alveolar patterns of modern Muridae and Nesomyidae (with the exception of the larger *Cricetomys*) and *Graphiurus* from South Africa, with a scale bar of 4 mm. Alveoli of the molars M^2^ are indicated in grey. Adapted and modified from [6].

**Fig 17:**
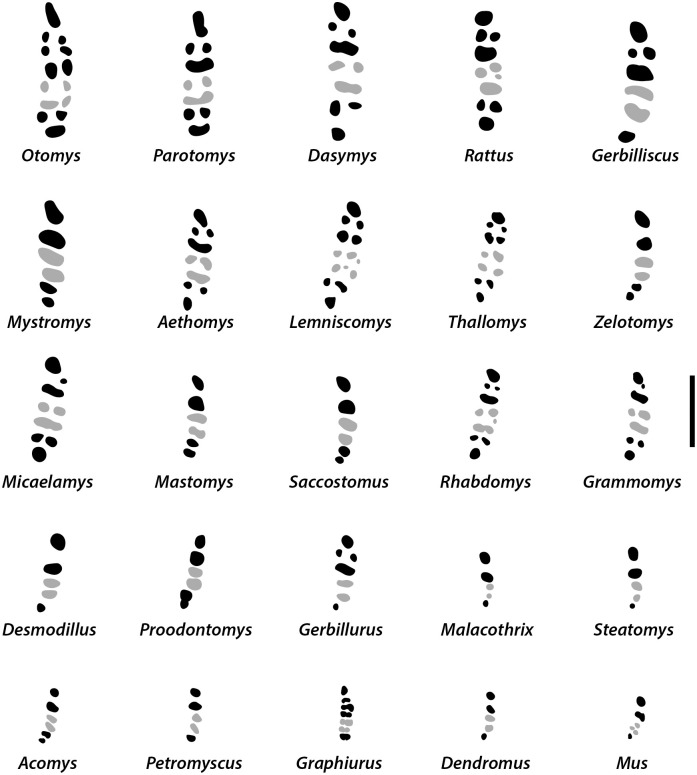
Right mandible alveolar patterns of modern Muridae and Nesomyidae (with the exception of the larger *Cricetomys*) and *Graphiurus* from South Africa, with a scale bar of 4 mm. Alveoli of the molars M_2_ are indicated in grey. Adapted and modified from [6].

Dental formula is 1-0-0-3:1-0-0-3. Alveolar formula is 3-3-3/2-2-2 (Figs 16 and 17).

#### Upper jaw

Upper incisors are opisthodont and ungrooved. The palatal foramina taper to the t4 of the M^1^. Molars are small and show a superficially *Mus-*like cusp configuration, with the t1 displaced backwards (sometimes almost in line with t5 and t6) and the t4 subsequently low. M^3^ is rather small and has a t3 but no t1 (group 2 in Fig 12), in contrast to Dendromurinae and most Murinae, including *Mus*.

#### Lower jaw

The M_1_ is the longest in the lower toothrow. The lingual anteroconid is approximately the same size as or slightly larger than the labial anteroconid. A posterolabial cusplet is sometimes present on the tooth. In South African species, the posterior cingulum is absent or very small in both the M_1_ and the M_2_. The molars M_2_ and M_3_ have two rows of cusps. The M_2_ displays a short anterolophid on its anterolabial zone, while the M_3_ displays a small anterolabial cusplet (more developed in *A*. *selousi* than in *A*. *subspinosus*) which tends to obliterate with wear (this cusplet is absent in *Mus*). The sciurognath mandible has a poorly developed coronoid process. The mandible is small, being of same average size than in *Steatomys*.

#### Systematic notes and South African fossil record

Two species are currently recognized in South Africa:

- *A*. *selousi* (Forster, 1778)
- *A*. *subspinosus* (Waterhouse, 1838)

The species *A*. *selousi* has been described as a split from as *A*. *spinosissimus* Peters, 1852 [22, 42], the latter occurring further north in South-Central Africa. Further taxonomic and biogeographic investigation of these species is required [43]. Two additional fossil species have been described:

- †*Acomys mabele* Denys, 1990 known only from the Pliocene site of Langebaanweg, and that constitutes the first occurrence of the genus in the South African fossil record
- *Acomys spinosissimus* Peters, 1852 described in various Pleistocene sites

Fossils of this genus have been recorded from many Quaternary fossil deposits. Based on the recent split between *A*. *selousi* and *A*. *spinosissimus*, which are morphologically undistinguishable, South African fossil specimens previously attributed to *A*. *spinosissimus* may be more parsimoniously assigned to *A*. *selousi*.

Subfamily **GERBILLINAE** Gray, 1825

Genus ***Desmodillus*** Thomas & Schwann, 1904 (Cape Short-tailed Gerbils)

Figs 18–20; Table 7

**Fig 18:**
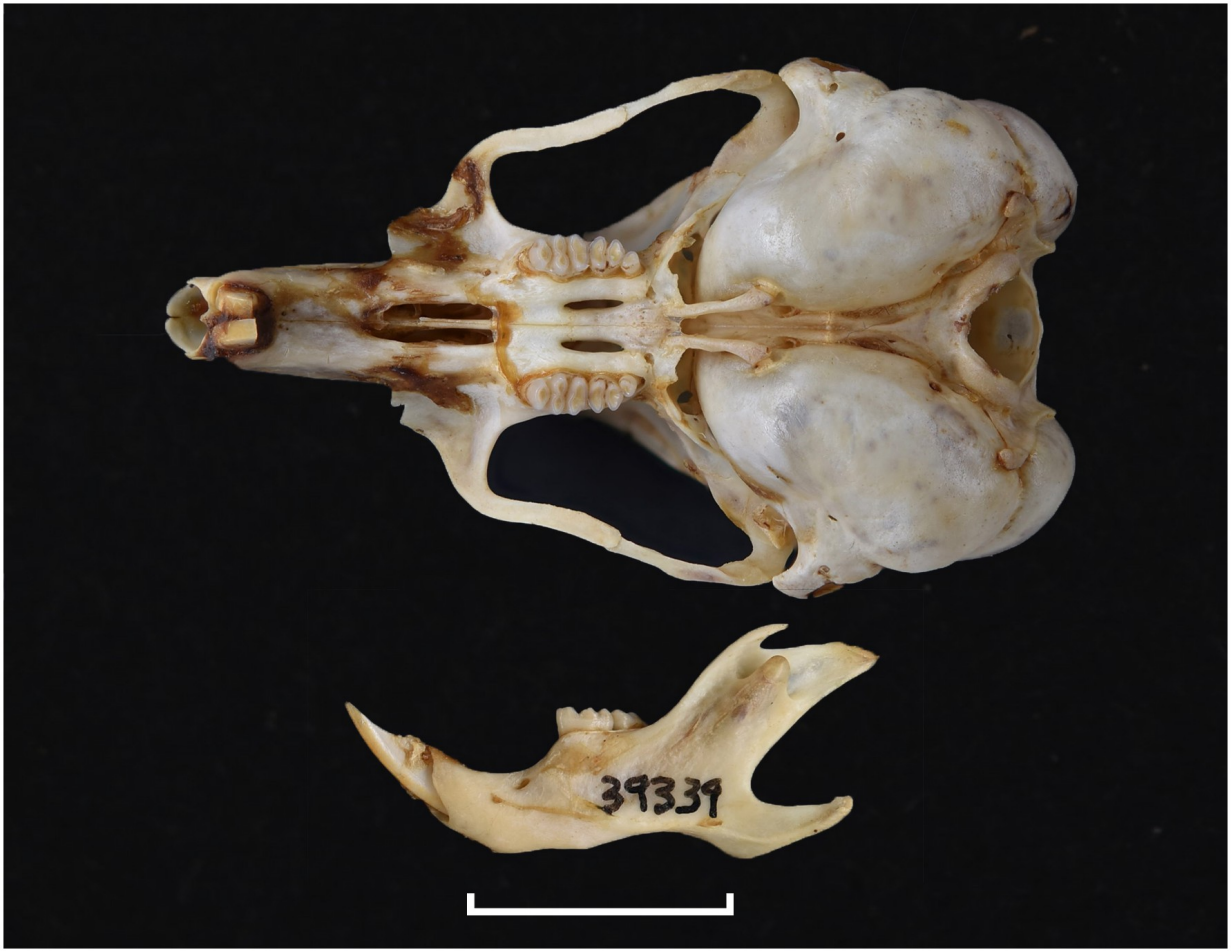
Cranium of *Desmodillus auricularis* (DNMN-39339), with a scale bar of 1 cm.

**Fig 19:**
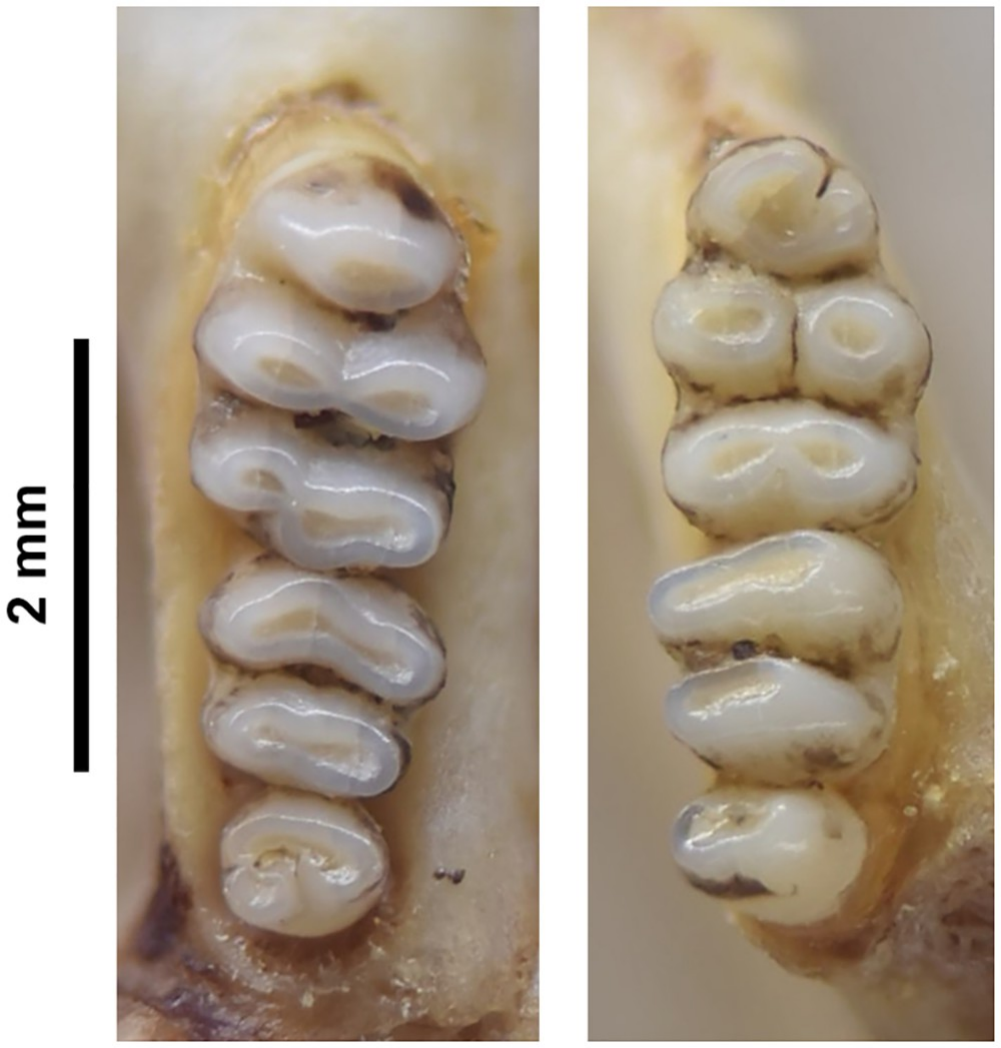
Cheekteeth of *Desmodillus*. Upper (a) and lower (b) right toothrow of *Desmodillus auricularis* (DNMNH-39365).

**Fig 20:**
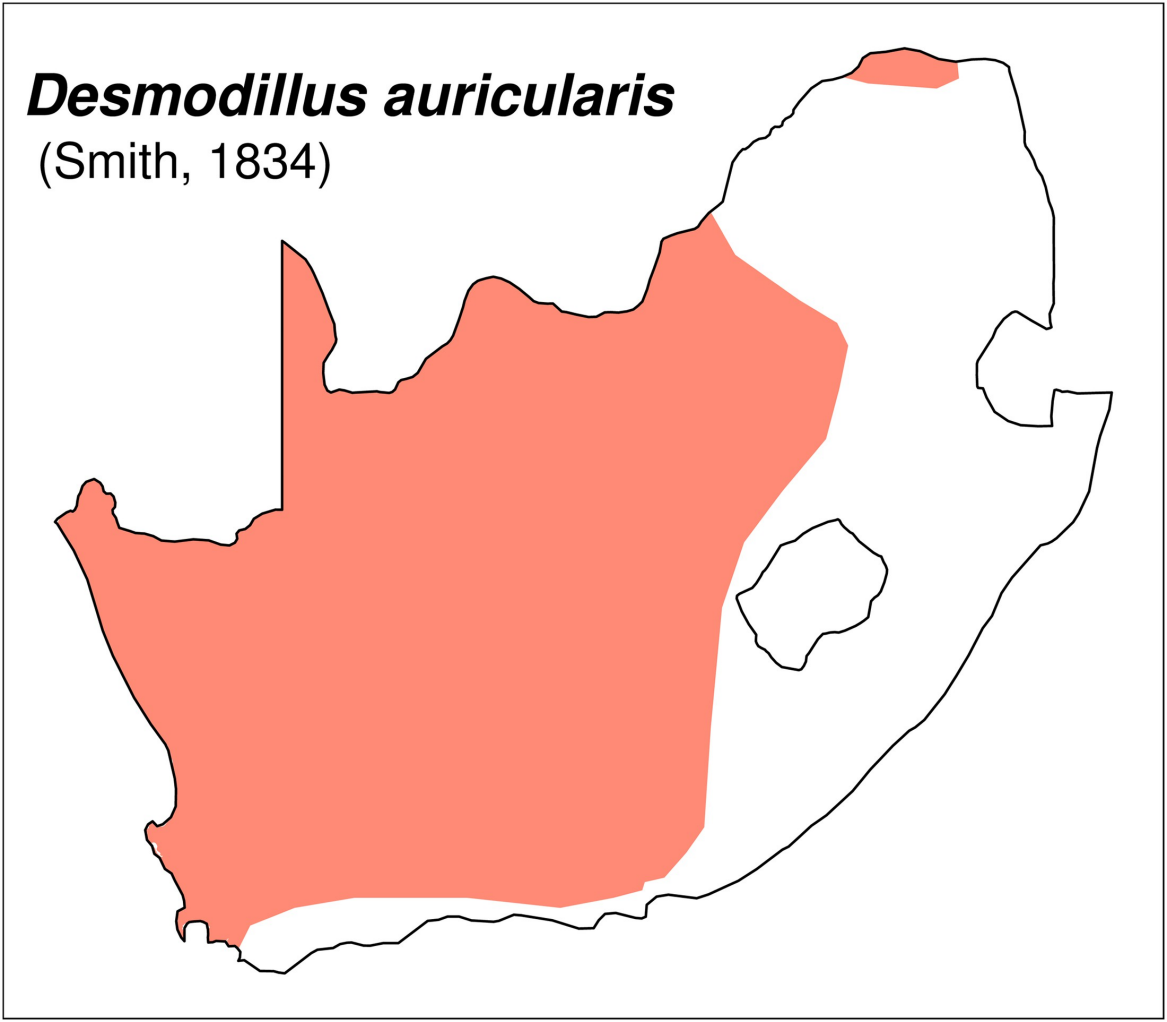
Distribution map.

**Table 7:** Dental measurements (in mm) for *Desmodillus auricularis*, sexes combined.

	Mean	Min	Max	n
LLTR	4.2	4.0	4.5	10
WM_1_	1.4	1.4	1.5	10
LUTR	4.5	4.2	5.0	10
WM^1^	1.6	1.4	2.0	10

Dental formula is 1-0-0-3:1-0-0-3. Alveolar formula is 3-3-1:2-2-1 (Figs 16 and 17).

#### Upper jaw

Upper incisors are long with a shallow groove. The anterior palatal foramina end short of the M^1^, the posterior palatal foramina extend between the second lamina of M^1^ to the beginning of M^3^. Molars show a semi-lophodont (or buno-lophodont) condition. M^1^ has three lobes, M^2^ has two, and M^3^ has one lobe only (species of *Gerbilliscus (Gerbillurus*) have one or two lobes). The central lobe in M^1^ has two circular cusps. Tympanic bullae are greatly inflated; they can even be seen when the skull is viewed from above. Fossil material that preserves only the toothrow is not always easily distinguished from *Gerbillurus*, although the length of the toothrow is greater in *Desmodillus*.

#### Lower jaw

Lower incisors are plain. Young specimens have two cusps in the first lobe of the M_1_ but they fuse with the age to form the typical horseshoe shape of Taterillini; the two cusps of the second lobe of M_1_ remain unfused, while they are fused in a lamina in the third lobe. M_2_ has two lobes. M_3_ is very small and consists of one tiny cusp. The angular process of the mandible is sharp and elongated, and its ventral edge makes an angle with the ventral margin of the mandibular body. The coronoid process is higher than in *Gerbillurus*.

#### Systematic notes and South African fossil record

The genus is monotypic:

- *Desmodillus auricularis* (Smith, 1834)

An additional fossil species has been described:

- *†Desmodillus magnus* Denys and Matthews, 2017 from Langebaanweg

Since the Early Pliocene, until the present, fossils of *Desmodillus* have been recorded from numerous fossil deposits.

Genus ***Gerbilliscus*** Thomas, 1897 (Gerbils & Hairy-footed Gerbils)

Subgenus ***Gerbilliscus* (*Gerbilliscus*)**

Figs 21–23, Table 8

**Fig 21:**
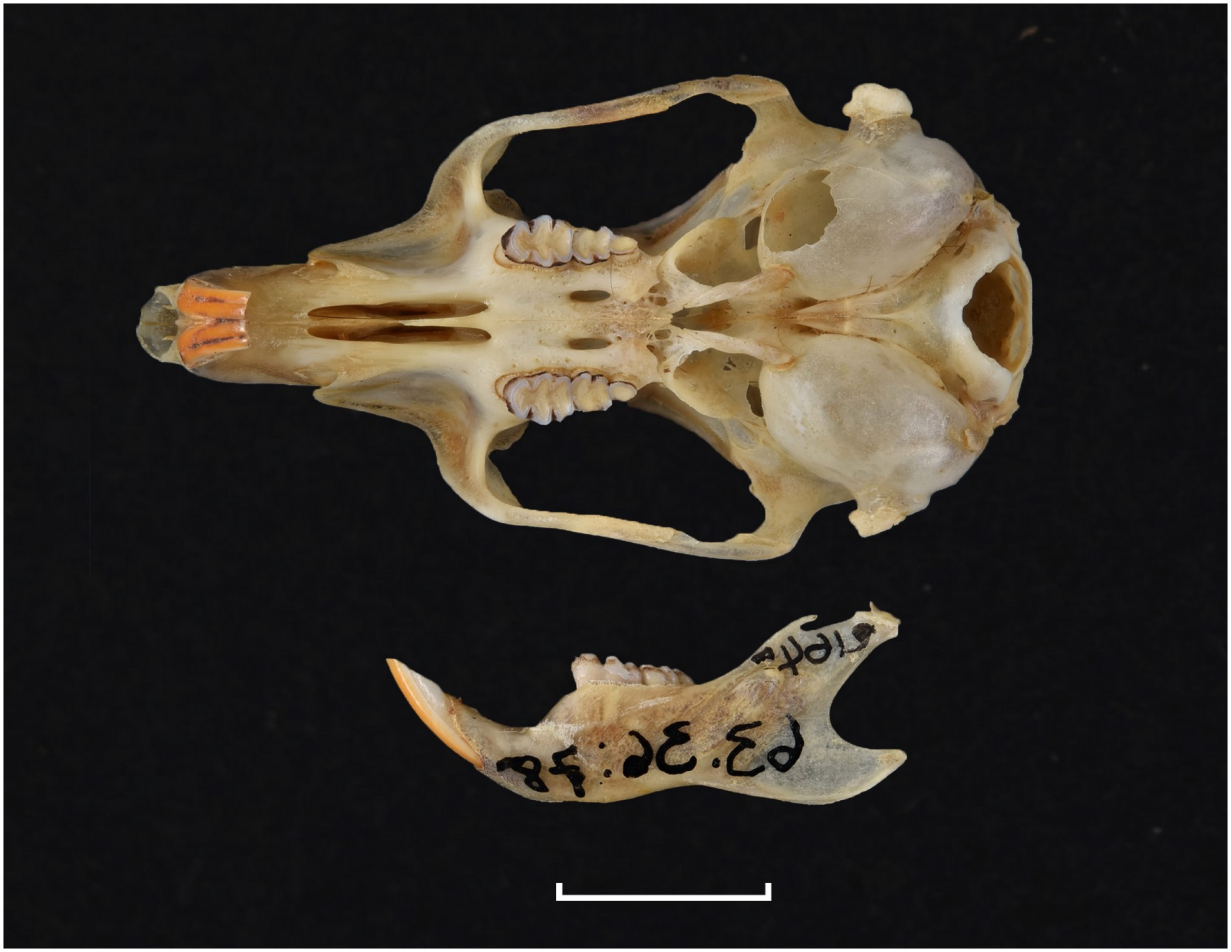
Cranium of *Gerbilliscus afra* (DNMN-21640), with a scale bar of 1 cm.

**Fig 22:**
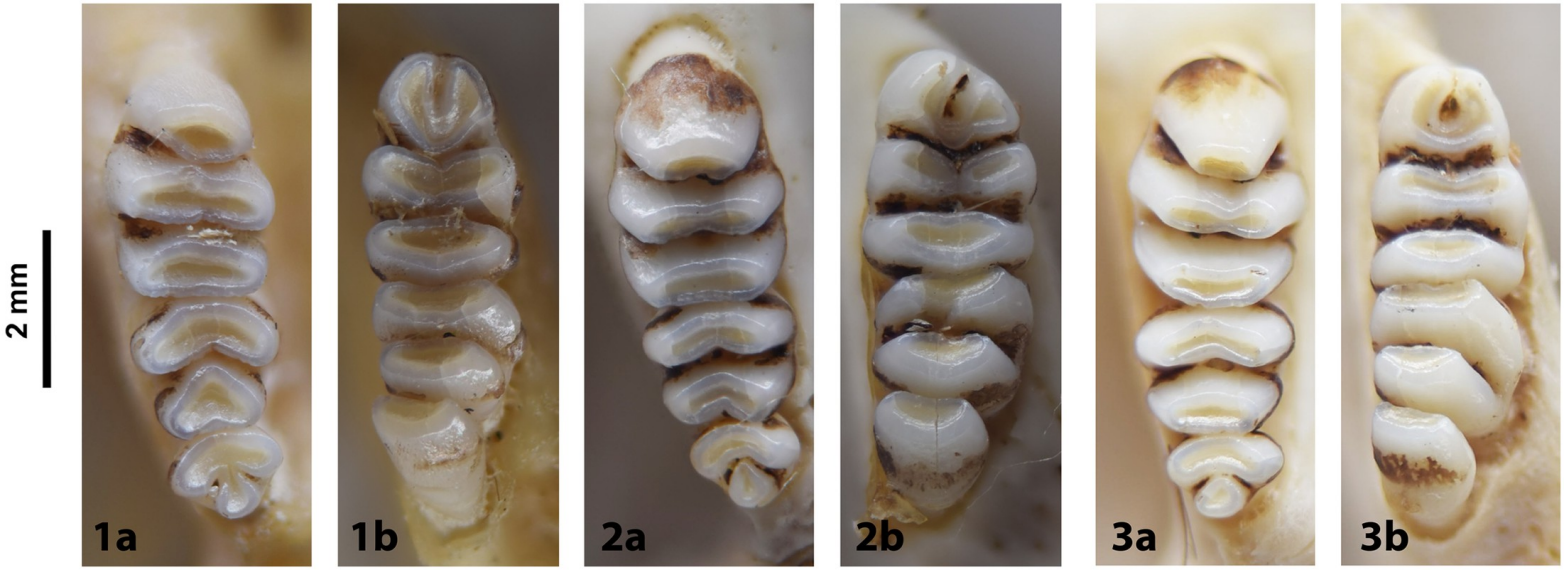
Cheekteeth of *Gerbilliscus (Gerbilliscus)*. **1)** Upper (a) and lower (b) right toothrow of *G*. *afra* (DNMNH-21634); **2)** Upper (a) and lower (b) right toothrow of *G*. *brantsii* (DNMNH-27755); **3)** Upper (a) and lower (b) right toothrow of *G*. *leucogaster* (DNMNH-44280).

**Fig 23:**
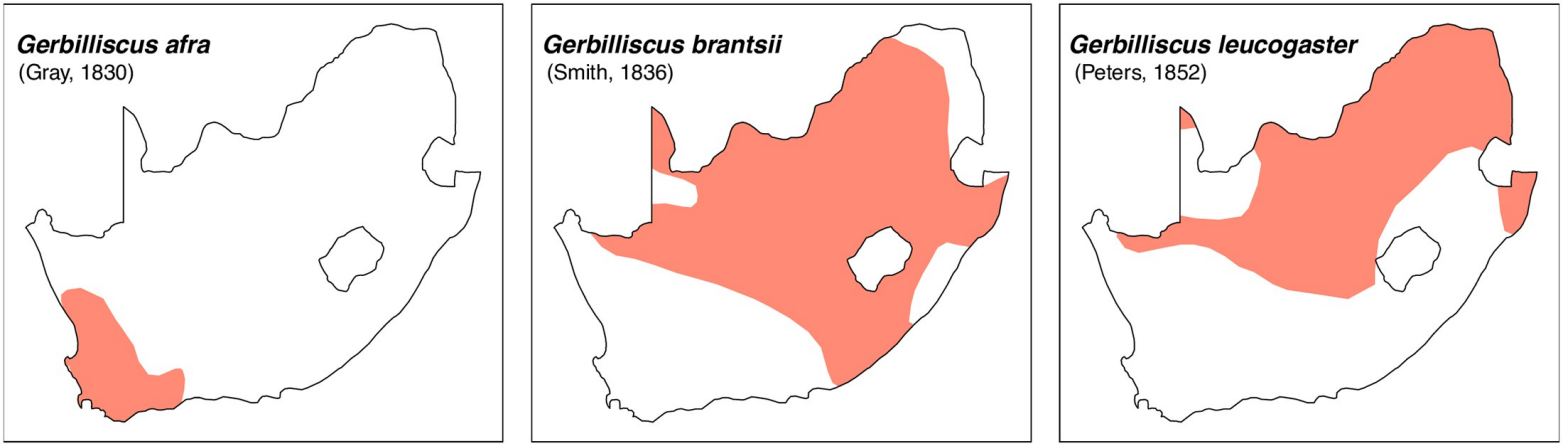
Distribution maps.

**Table 8:** Dental measurements (in mm) for *Gerbilliscus* (*Gerbilliscus*) from South Africa, sexes and species combined.

	Mean	Min	Max	n
LLTR	6.1	5.3	6.8	59
WM_1_	2.2	1.8	2.5	59
LUTR	6.2	5.5	7.4	57
WM^1^	2.3	2.0	2.6	57

Dental formula is 1-0-0-3:1-0-0-3. Alveolar formula is 4-2-1:4-2-1 (Figs 16 and 17).

#### Upper jaw

Upper incisors are slightly opisthodont, yellow to orange in colour, and have a single groove. The anterior palatal foramina barely reach the alveolus of M^1^; the posterior palatal foramina are not as developed as in *Desmodillus* and *Gerbilliscus*. Molars show a lophodont condition, with cusps fused in transverse laminae lacking longitudinal connections. The molar M^1^ has three laminae, the first consisting of a single cusp; molars M^2^ and M^3^ both have two laminae when unworn.

#### Lower jaw

Molars show lophodont condition, with cusps fused in transverse laminae that lack longitudinal connections. The anteroconid of M_1_ has a horseshoe shape whose appearance is highly variable. The M_2_ has two laminae, and the M_3_ has one. The mandible is elongated and seems vertically compressed, with coronoid and condylar processes projecting backwards.

#### Systematic notes and South African fossil record

Species of the genus *Gerbilliscus* were previously included in *Gerbillus*, then in *Tatera*, but are now grouped in their own distinct genus [44, 45]. The genera *Gerbilliscus* and *Gerbillurus* have long been treated as two distinct genera, but recent molecular and chromosomal analysis suggest that they should be combined into one genus, *Gerbilliscus* [46]. In South Africa, three species of *Gerbilliscus* (excluding *Gerbillurus*) are currently recognised:

- *Gerbilliscus afra* (Gray, 1830),
- *Gerbilliscus brantsii* (Smith, 1836)
- *Gerbilliscus leucogaster* (Peters, 1852).

The first occurrences of this genus are from Makapansgat [47, 48] and Taung [1, 47, 49] in the Pliocene. Remains of *Gerbilliscus* are found in many deposits throughout the Quaternary [50].

Subgenus ***Gerbilliscus* (*Gerbillurus*)**

Figs 24–26; Table 9

**Fig 24:**
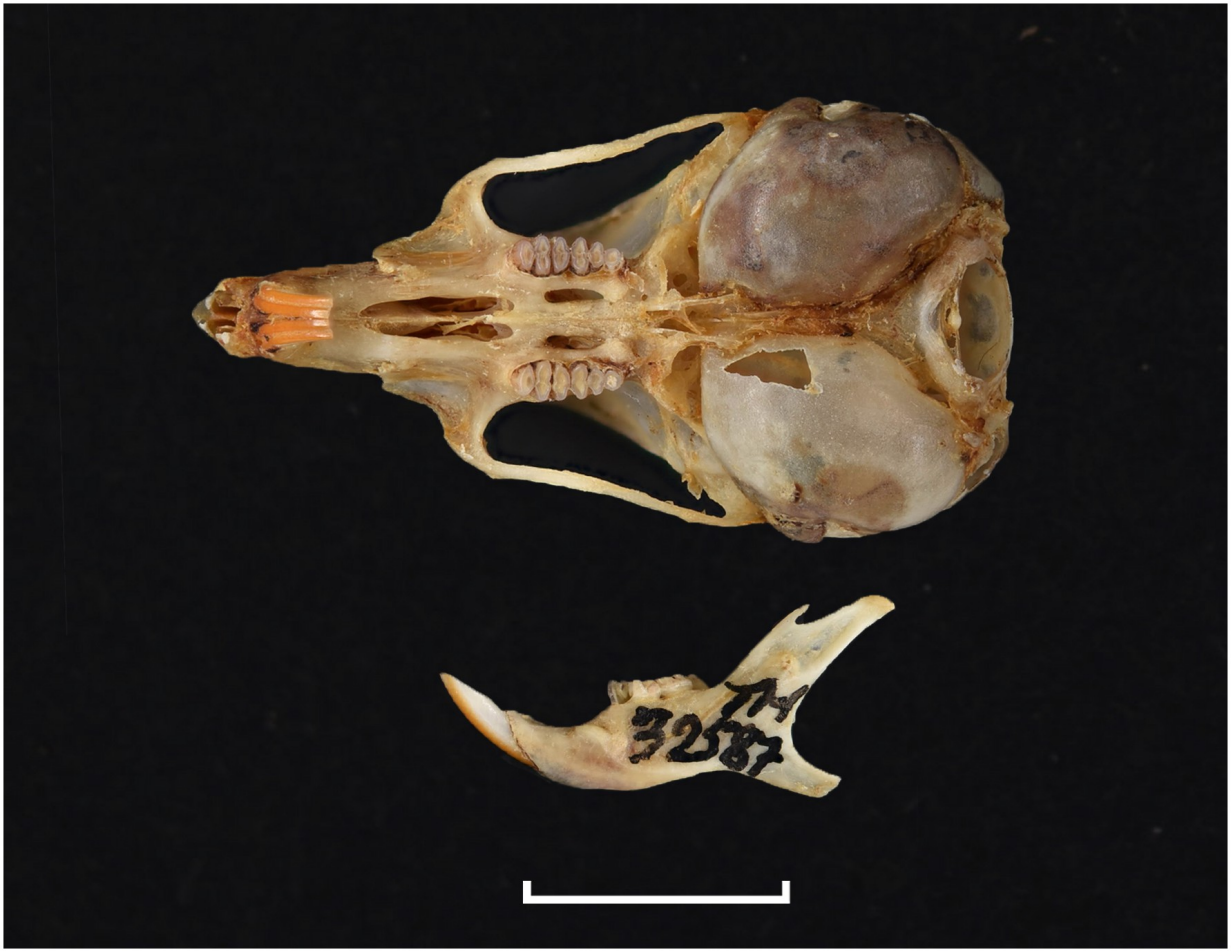
Cranium of *Gerbilliscus (Gerbillurus*) vallinus (DNMN-21640), with a scale bar of 1 cm.

**Fig 25:**
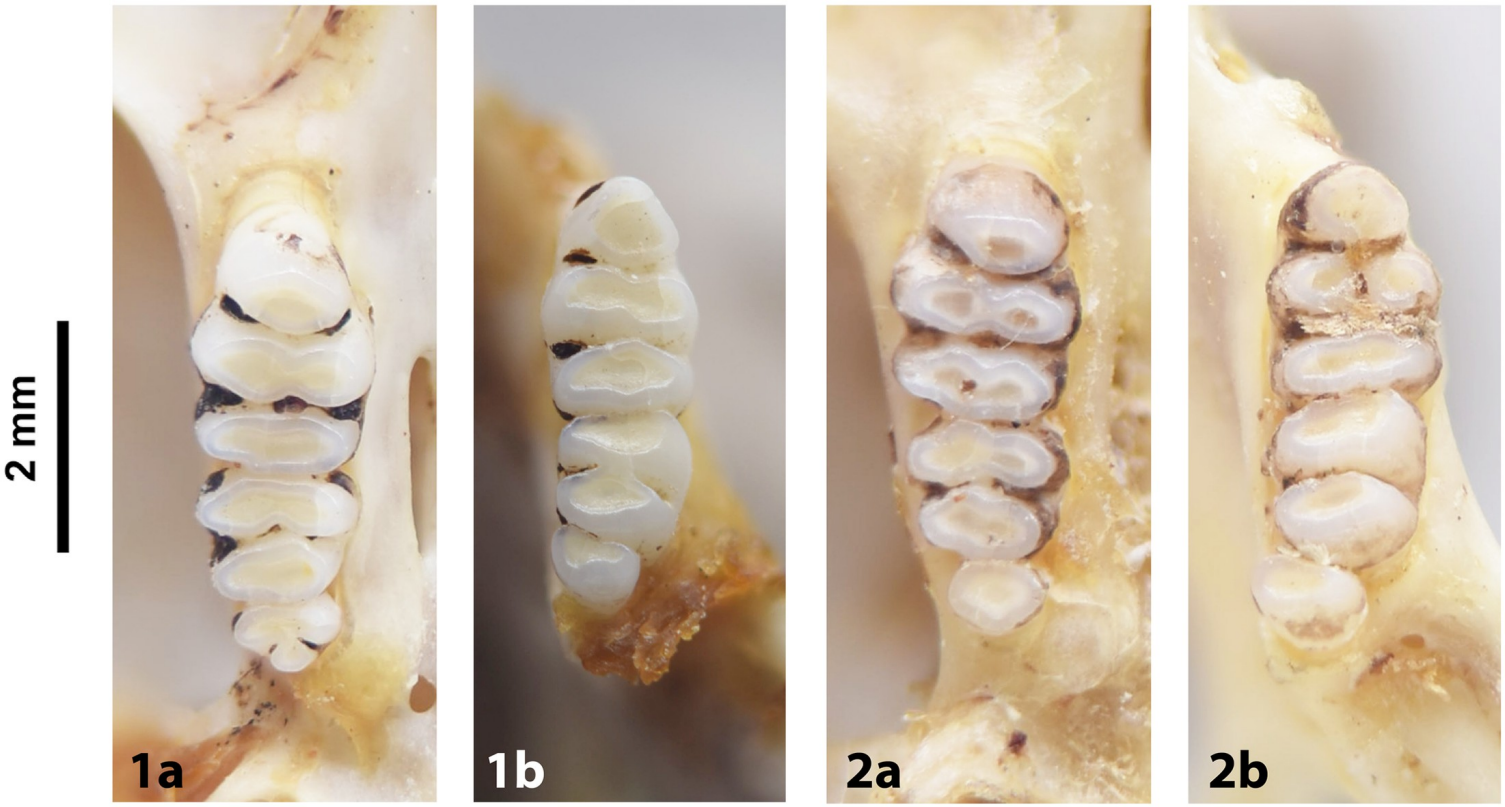
Cheekteeth of *Gerbilliscus* (*Gerbillurus*). **1)** Upper (a) and lower (b) right toothrow of *G*. *paeba* (DNMNH-32636); **2)** Upper (a) and lower (b) right toothrow of *G*. *vallinus* (DNMNH-32580).

**Fig 26:**
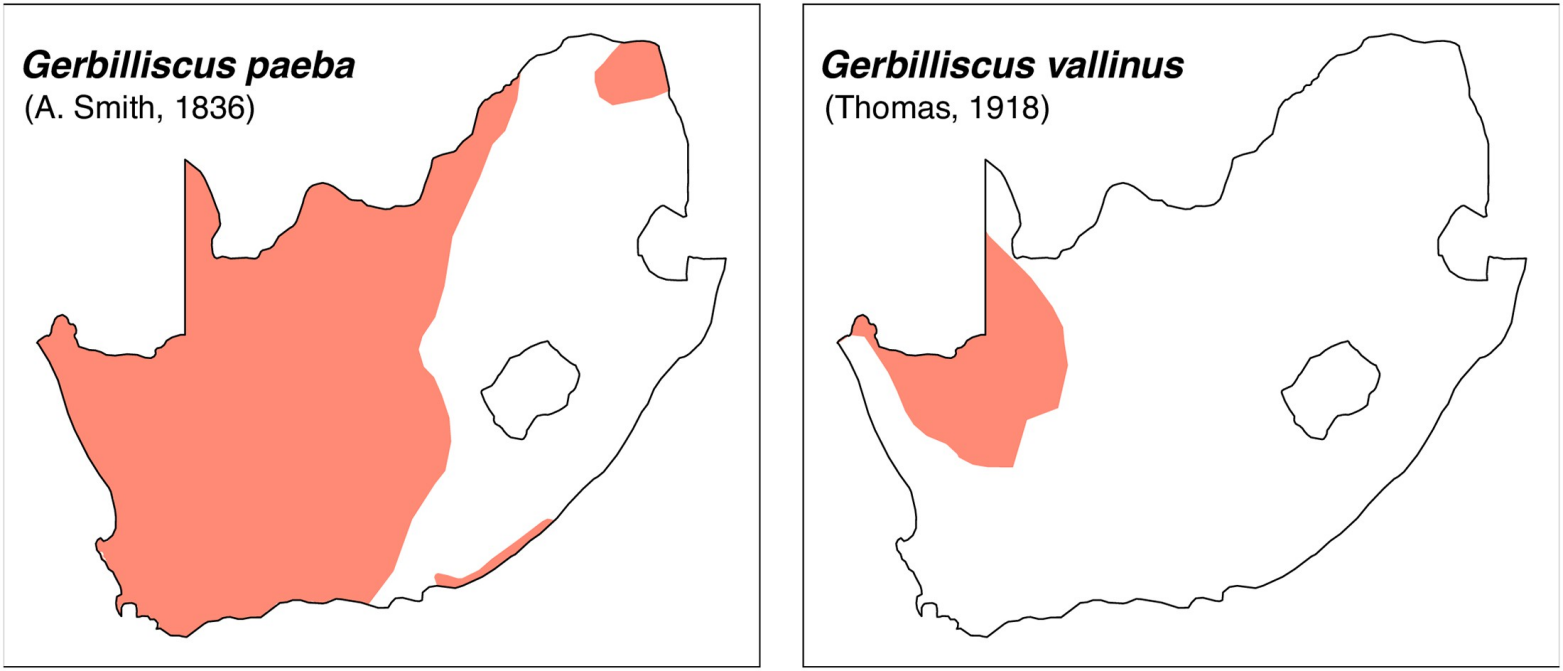
Distribution maps.

**Table 9:** Dental measurements (in mm) for *Gerbilliscus* (*Gerbillurus*) from South Africa, sexes and species combined.

	Mean	Min	Max	n
LLTR	4.1	3.6	4.4	32
WM_1_	1.3	1.2	1.5	32
LUTR	4.1	3.7	4.5	33
WM^1^	1.5	1.3	1.6	33

Dental formula is 1-0-0-3:1-0-0-3. Alveolar formula is 4-2-1:4-2-1 (Figs 16 and 17).

#### Upper jaw

Upper incisors are strongly opisthodont, yellow to orange, and have a single groove. The anterior palatal foramina end short of the M^1^, the posterior palatal foramina extend between the second lamina of M^1^ to the beginning of M^3^. Molars show semi-lophodont condition, with cusps fused in transverse laminae that lack longitudinal connections. M^1^ has three lobes, M^2^ has two lobes, and M^3^ has two lobes, poorly, or completely, fused (in *Desmodillus* the second lobe is always much reduced). Fossil material that preserves only the teeth is not always easily distinguishable from *Desmodillus*, although the length of the toothrow is smaller in *Gerbilliscus*.

#### Lower jaw

As opposed to *Gerbilliscus (Gerbilliscus*), cusps are high and round and do not fuse together in transverse laminae as in other *Gerbilliscus* spp. Lower molars are very similar to those of *Desmodillus*, but the M_1_ has four alveoli and the incisor is thinner. Mandible is small, vertically compressed and not as high as in *Desmodillus*.

#### Systematic notes and South African fossil record

Until recently, specimens of *Gerbillurus* were placed in their own genus based on the marked morphological difference with other *Gerbilliscus*, but recent chromosomal and molecular analyses indicate they should be assigned to *Gerbilliscus*. In South Africa, two species of *Gerbilliscus* (*Gerbillurus*) are currently recognized:

- *Gerbilliscus paeba* (A. Smith, 1836)
- *Gerbilliscus vallinus* (Thomas, 1918)

The oldest remains of *Gerbillurus* in the South African fossil record are found in the Early Pleistocene site of Wonderwerk [6, 51].

Subfamily **MURINAE** Illiger, 1811

Genus ***Aethomys*** Thomas, 1915 (Veld Rats)

**Fig 27:**
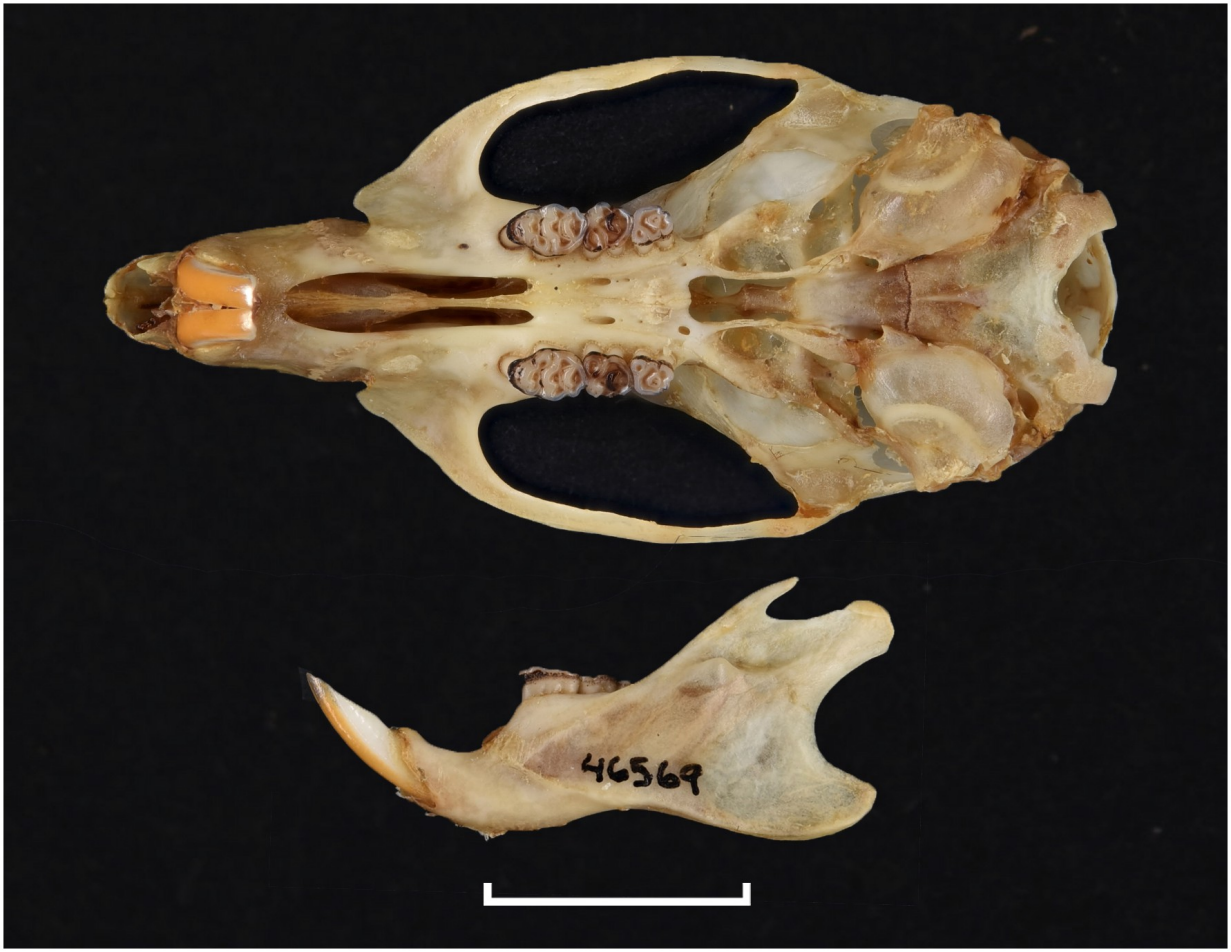
Cranium of *Aethomys ineptus* (DNMNH-4659), with scale bar of 1 cm.

**Fig 28:**
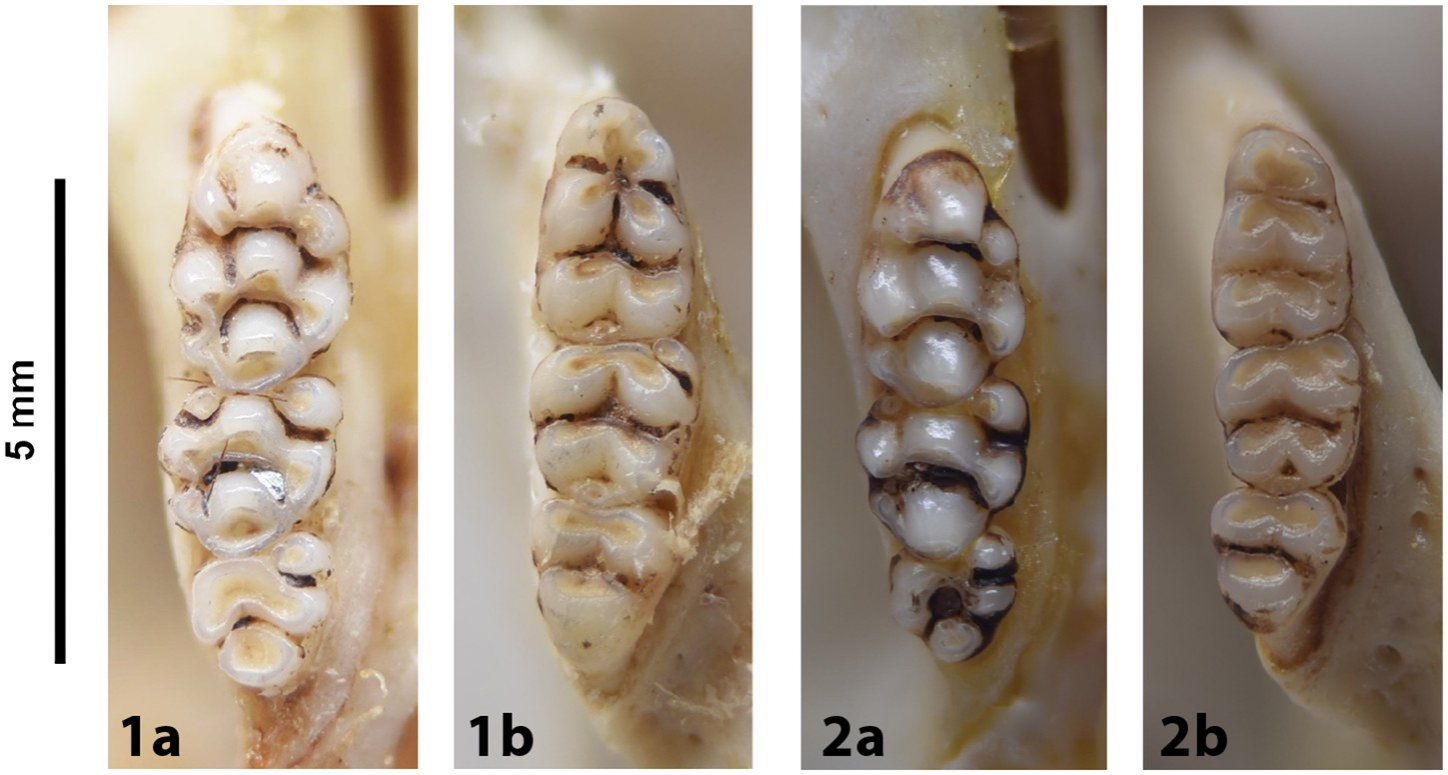
Cheekteeth of *Aethomys*. **1)** Upper (a) and lower (b) right toothrow of *A*. *chrysophilus* (DNMNH-4659); **2)** Upper (a) and lower (b) right toothrow of *A*. *ineptus* (DNMNH-46903).

**Fig 29:**
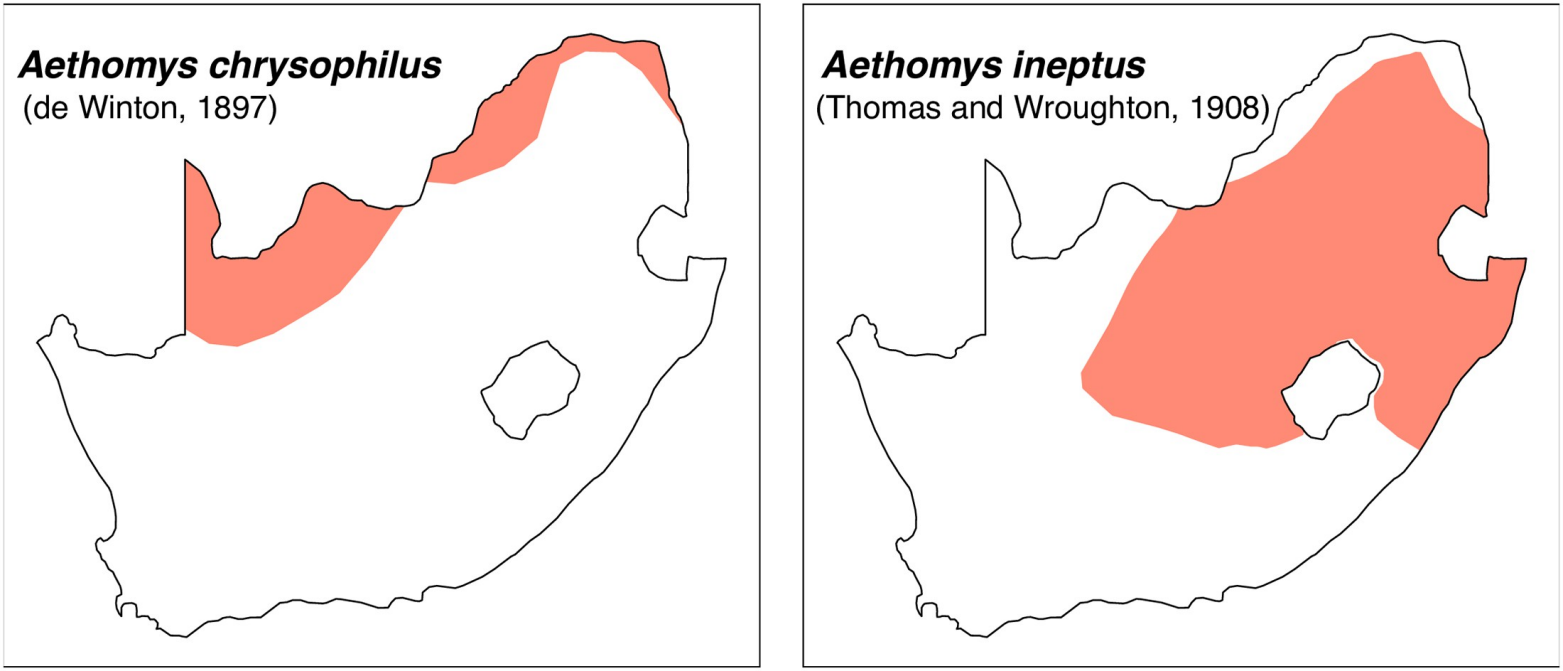
Distribution maps.

Figs 27–29; Table 10

**Table 10:** Dental measurements (in mm) for *Aethomys* from South Africa, sexes and species combined.

	Mean	Min	Max	n
LLTR	5.1	5.1	7.1	65
WM_1_	1.7	1.5	2.3	65
LUTR	6.0	5.0	7.2	67
WM^1^	2.0	1.7	2.4	67

Dental formula is 1-0-0-3:1-0-0-3. Alveolar formula is 4-3-3:4-3-3 (Figs 16 and 17).

#### Upper jaw

Incisors are ungrooved and opisthodont. The anterior palatal foramina are long and extend beyond the level of the M^1^. Molars are relatively broad. In M^1^, the t1 is situated slightly or well behind t2 and t3; there is often a small stephanodont crest uniting t6 and t9 on M^1^ and on M^2^; t7 is absent. The configuration of M^3^ corresponds to group 3 or 4 in Fig 12, with the distal lobe having often one elongated cusp (or two poorly differentiated, as opposed to two well differentiated in *Lemniscomys*) although this criterion shows variability in few specimens.

#### Lower jaw

The M_1_ sometimes display lateral cusplets or small ridges that can form a stephanodont crest; it has no conspicuous posterior cingulum (a condition similar in *Micaelamys* and *Lemniscomys*), although this feature is not constant. In the M_1_ there is no tma (it is present in *Micaelamys*) but some specimens may display a tiny anteromedian cusplet. The M_2_ typically has three alveoli (two isolated and two fused ones).

#### Systematic notes and South African fossil record

Species of the genus *Micaelamys* were previously included in *Aethomys*, but they were later placed in their own genus based on molecular and morphological data [52]. Two species of *Aethomys* are currently recognized in South Africa:

- *Aethomys chrysophilus* (de Winton, 1897)
- *Aethomys ineptus* (Thomas and Wroughton, 1908)

Additional fossil species have been identified:

- *†Aethomys adamanticola* Denys, 1990 from the Early Pliocene locality of Langebaanweg
- *†Aethomys modernis* Denys, 1990 from the Early Pliocene locality of Langebaanweg

Fossils of *Aethomys* are known from many Pleistocene and Holocene sites from South Africa [50]. Many of these specimens were identified as *Aethomys chrysophilus* at a time when *A*. *ineptus* was not recognized yet as a cryptic species distinct from *A*. *chrysophilus*. As these two species appear to be morphologically indistinguishable using the cranio-dental anatomy, caution is recommended when making an osteological identification

Genus ***Dasymys*** Peters, 1875 (Shaggy Rats)

**Fig 30:**
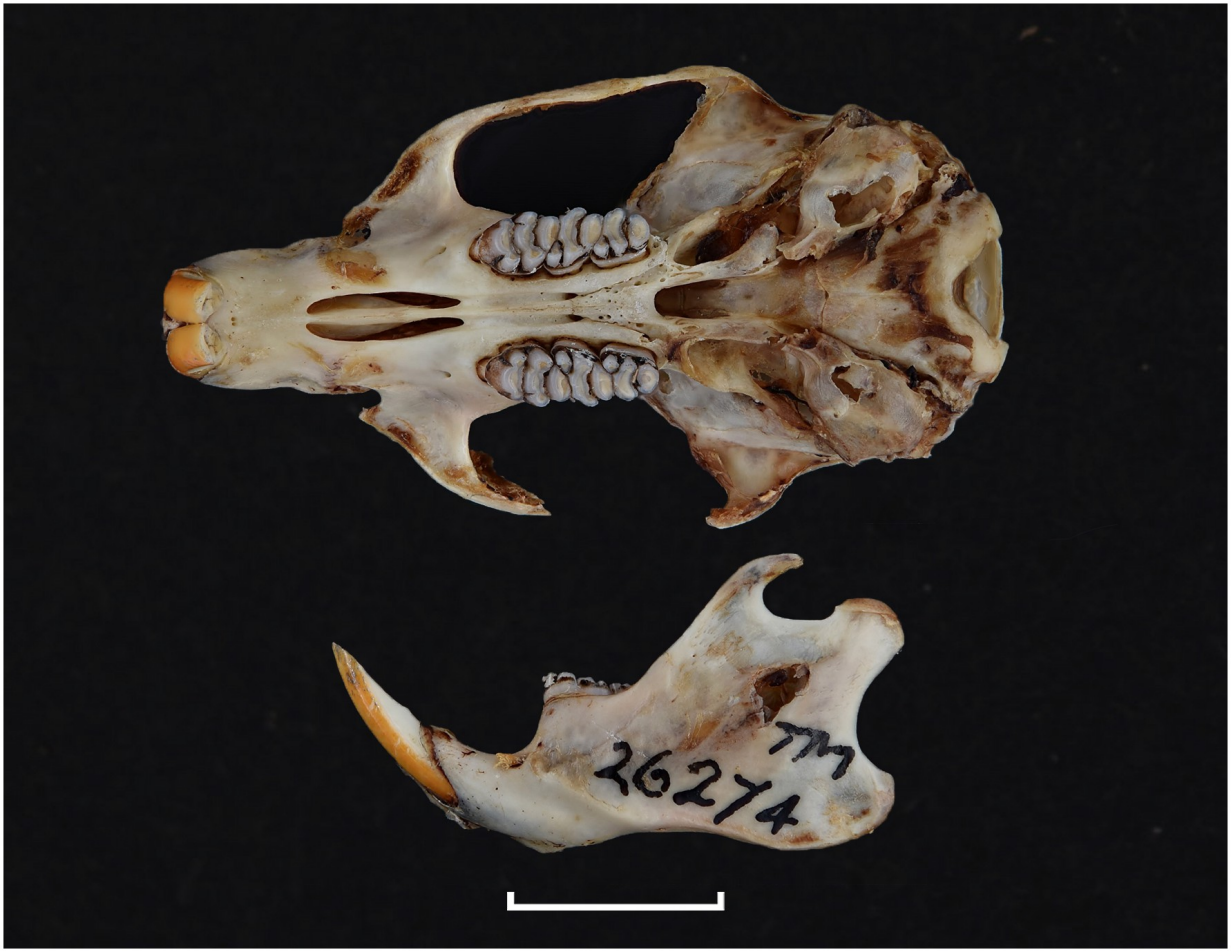
Cranium of *D*. *capensis* (DNMNH-26274), with scale bar of 1 cm.

**Fig 31:**
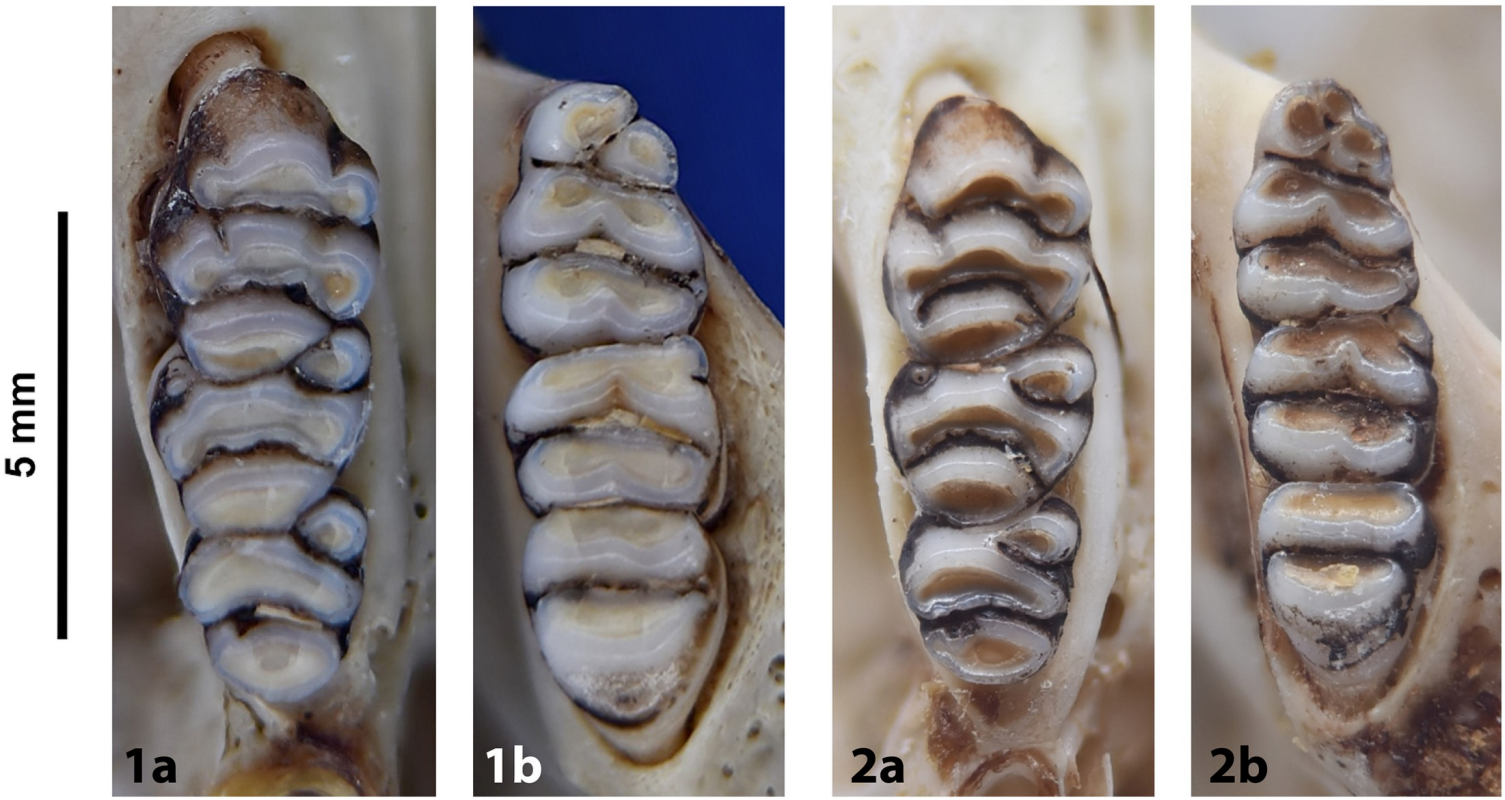
Cheekteeth of *Dasymys*. **1)** Upper (a) and lower (b) right toothrow of *D*. *capensis* (DNMNH-26274); **2)** Upper (a) and lower (b) right toothrow of *D*. *robertsii* (DNMNH-30639).

**Fig 32:**
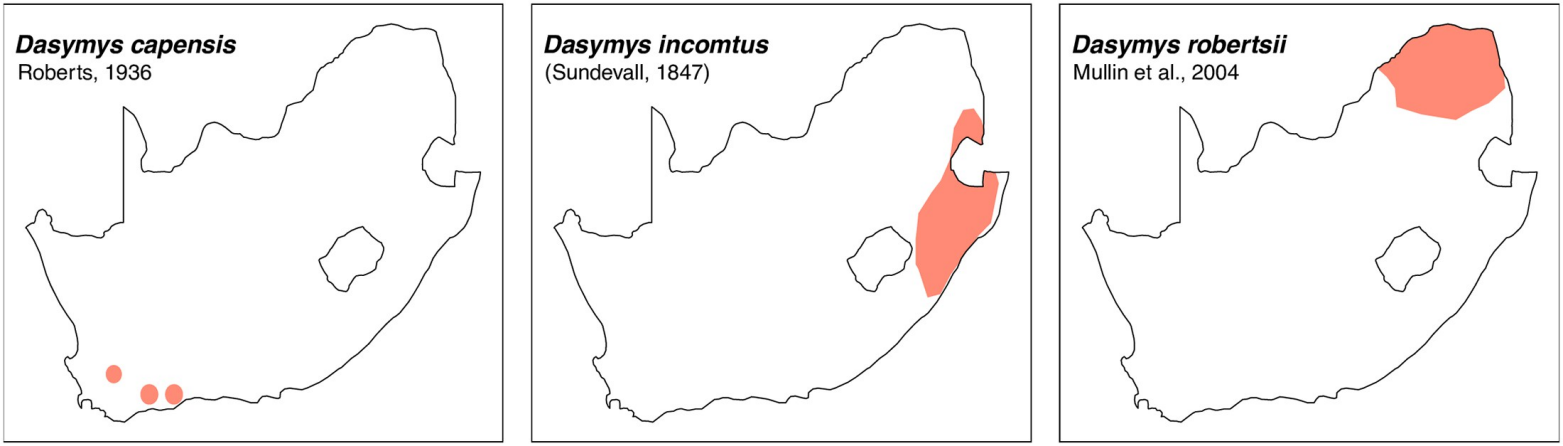
Distribution maps.

Figs 30–32; Table 11

**Table 11:** Dental measurements (in mm) for *Dasymys* from South Africa, sexes and species combined.

	Mean	Min	Max	n
LLTR	7.5	6.4	8.4	31
WM_1_	2.3	1.8	2.6	32
LUTR	7.6	6.8	8.3	29
WM^1^	2.6	2.1	2.8	29

Dental formula is 1-0-0-3:1-0-0-3. Alveolar formula is 6-5-3:4/5-3/4-3 (Figs 16 and 17).

#### Upper jaw

Upper incisors are broad and ungrooved. The palatal foramina end just before the alveolus of the first root of the M^1^. Molars are large and heavily cusped, exhibiting macrodonty. Rows of cusps are arranged in transverse rows, with t1 in line with t2 and t3 in M^1^. With age, rows of tubercles are obliterated, and the spaces between the original rows of cusps may isolate as characteristic enamel islands. The configuration of M^3^ corresponds to Group 4 in Fig 12.

#### Lower jaw

Lower incisors are ungrooved. Molars are large and heavily cusped, showing macrodonty. M_1_ has three cusps on the prelobe, and no posterior cingulum (or a tiny one). Most of the cusps are fused in laminae that tend to isolate as enamel islands with the age. M_2_ and M_3_ have two lobes each and are large. The posterior root of the M_3_ is large. The mandible is rather massive and high.

#### Systematic notes and South African fossil record

The number of species recognised within this genus has changed several times over the past decades, and species limits are still not fully resolved [22]. Currently, three species are described in South Africa:

- *Dasymys capensis* Roberts, 1936
- *Dasymys incomtus* (Sundevall, 1847)
- *Dasymys robertsii* Mullin et al., 2004

Both *D*. *capensis* and *D*. *robertsii* were previously identified as *D*. *incomtus*. Although the three species should be distinguishable on a craniometric basis (if the preservation of the fossils allows it), most of the fossils of *Dasymys* were identified at a time when only *D*. *incomtus* was recognized. This material therefore should be re-evaluated. Additional fossil species from South African deposits have been described:

- *†Dasymys bolti* Broom [Unpublished]
- *†Dasymys broomi* Broom [Unpublished]
- *†Dasymys lavocati* Broom [Unpublished]

*D*. *bolti* was described by Denys [47], but the other two fossil species *D*. *broomi* and *D*. *lavocati* have not been published and are considered as invalid species [4].

Genus ***Grammomys*** Thomas, 1915 (Thicket Rats)

**Fig 33:**
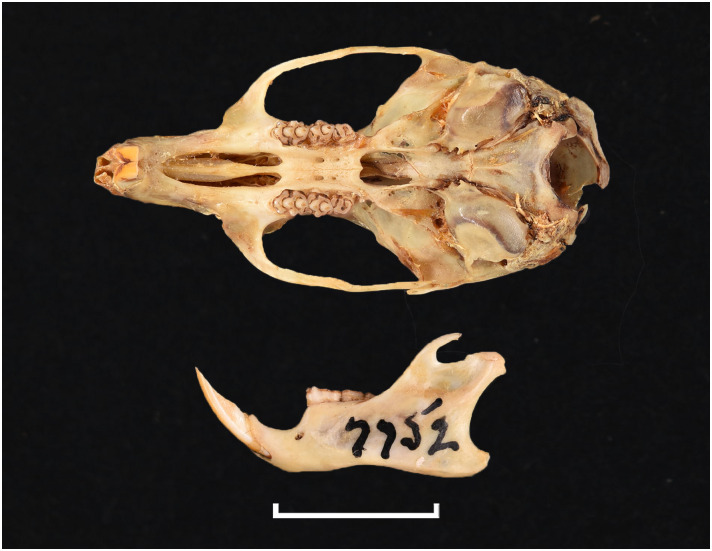
Cranium of *Grammomys cometes* (DNMNH-7752), with scale bar of 1 cm.

**Fig 34:**
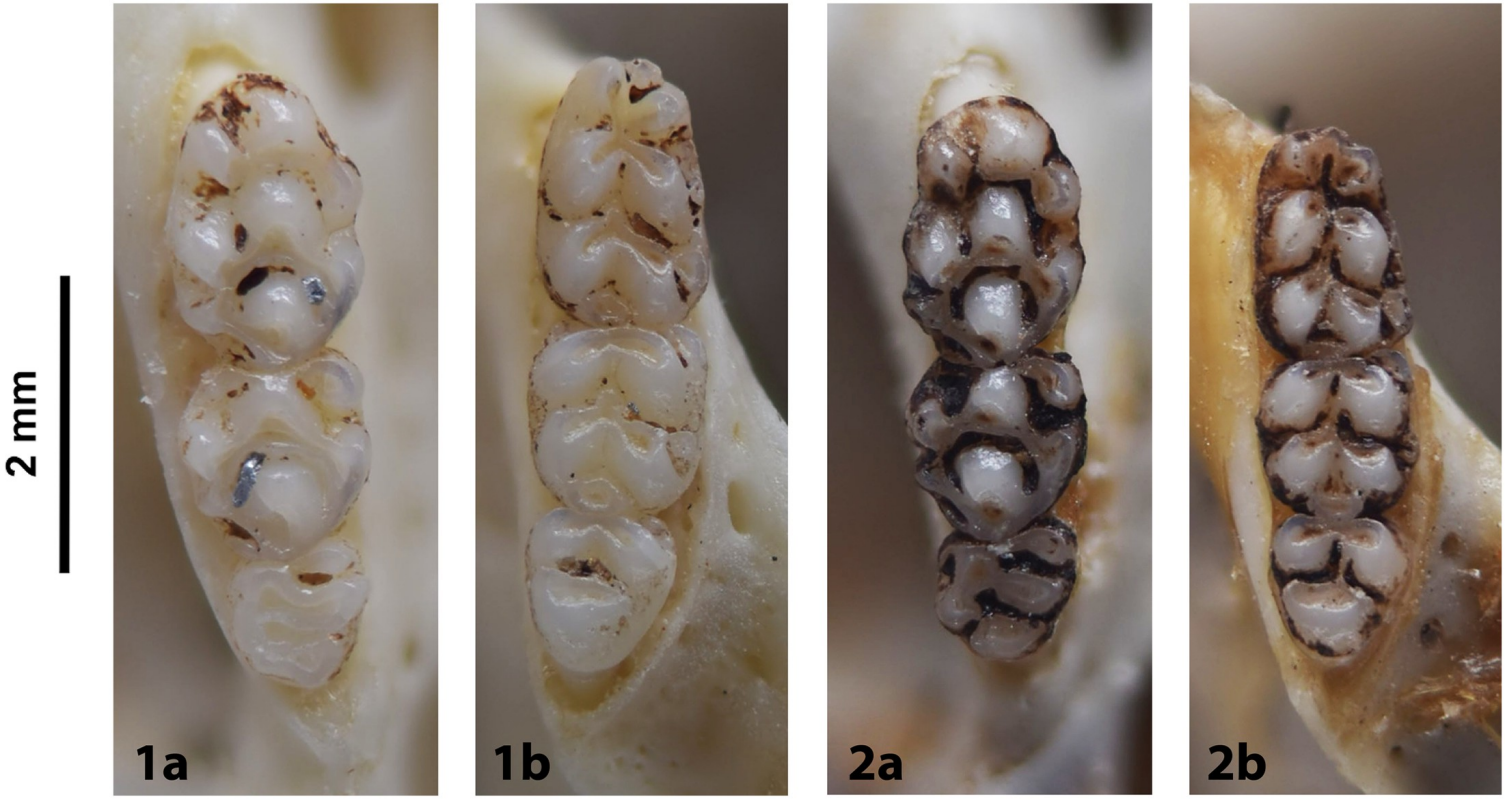
Cheekteeth of *Grammomys*. **1)** Upper (a) and lower (b) right toothrow of *G*. *cometes* (DNMNH-40467); **2)** Upper (a) and lower (b) right toothrow of *G*. *dolichurus* (DNMNH-10403).

**Fig 35:**
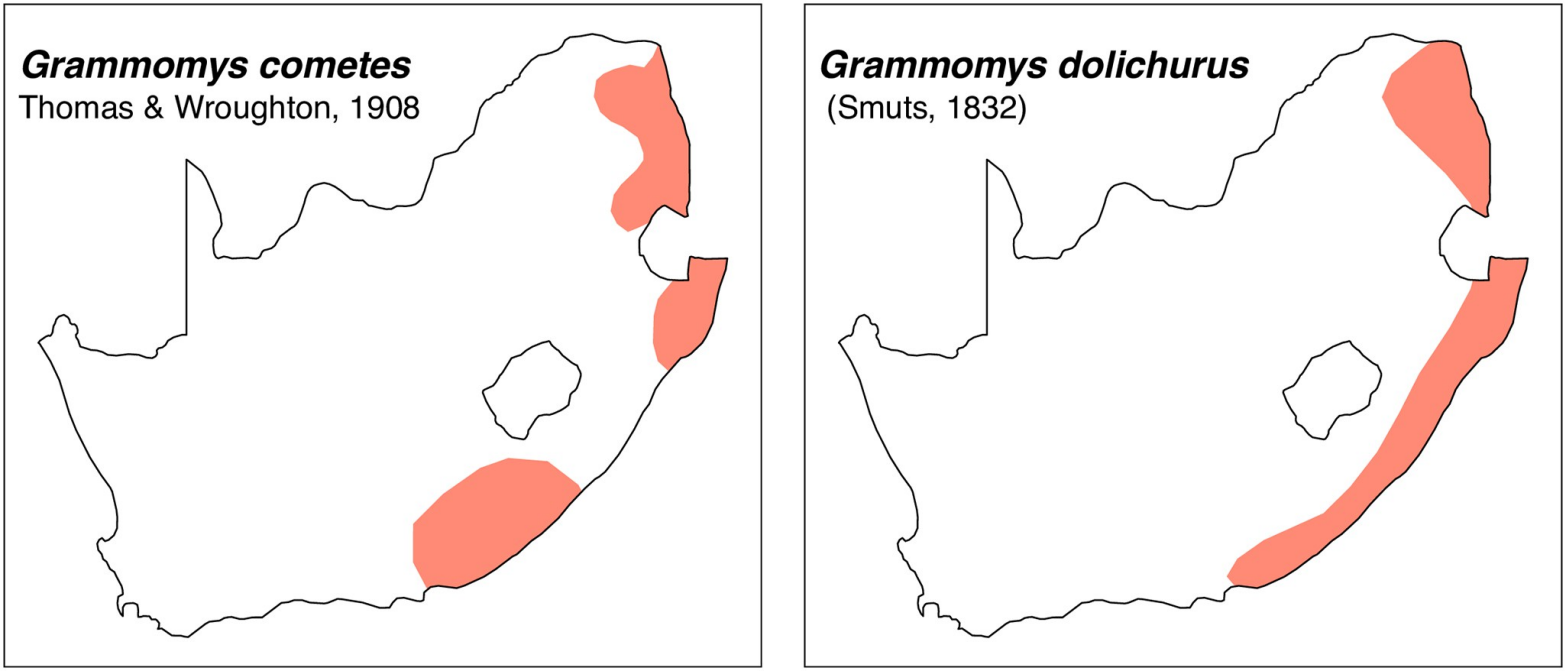
Distribution maps.

Figs 33–35; Table 12

**Table 12:** Dental measurements (in mm) for *Grammomys* from South Africa, sexes and species combined. Note that there is a significant size difference between the two species.

	Mean	Min	Max	n
LLTR	4.5	3.9	5.1	28
WM_1_	1.3	1.0	1.6	28
LUTR	4.4	3.9	5.1	27
WM^1^	1.4	1.3	1.8	27

Dental formula is 1-0-0-3:1-0-0-3. Alveolar formula is 4/5-4/5-3:2/4-3/4-3 (Figs 16 and 17).

#### Upper jaw

Upper incisors are ungrooved and orthodont. The anterior palatal foramina are long and penetrate between the first upper molars. The palate is broad, and the molars show microdonty. The cusps are well separated from each other and linked by stephanodont crests. In the M^1^, t1 is very slightly behind t2 and t3, and t4 is slightly behind t5 and t6. The M^1^ has also a small crestiform t7, and an accessory anterior median cusp is occasionally present. The cusp t3 is reduced or absent in M^2^, and the t9 is well visible and situated close to the t6. On the M^3^, presence of a t1 and a tiny t3, and trace of median longitudinal link (group 6 in Fig 12).

#### Lower jaw

Lower incisors are ungrooved. Molar cusps are high and well separated. There are three cusps on the first lobe of M_1_, a stephanodont crest uniting the first lobe with second and third lobes, and a conspicuous posterior cingulum. The M_2_ has two lobes with two cusps each, a small anterolabial cusp and lateral cusplets or small ridges that can form stephanodont crest, as well as a posterior cingulum. The M_3_ displays a tiny antero-external cusp.

#### Systematic notes and South African fossil record

*Grammomys* was previously considered as subgenus of *Thamnomys* but this genus is now elevated to genus rank based on morphological and phylogenetic analysis [14, 21, 53]. According to Monadjem et al. [22], this genus critically needs revision. Two species are currently recognized in South Africa:

- *Grammomys cometes* Thomas & Wroughton, 1908
- *Grammomys dolichurus* (Smuts, 1832)

Fossils are known from Pliocene to Holocene deposits, with the oldest occurrence in Makapansgat Limeworks [1, 49].

Genus ***Lemniscomys*** Trouessart, 1881 (Grass Mice)

**Fig 36:**
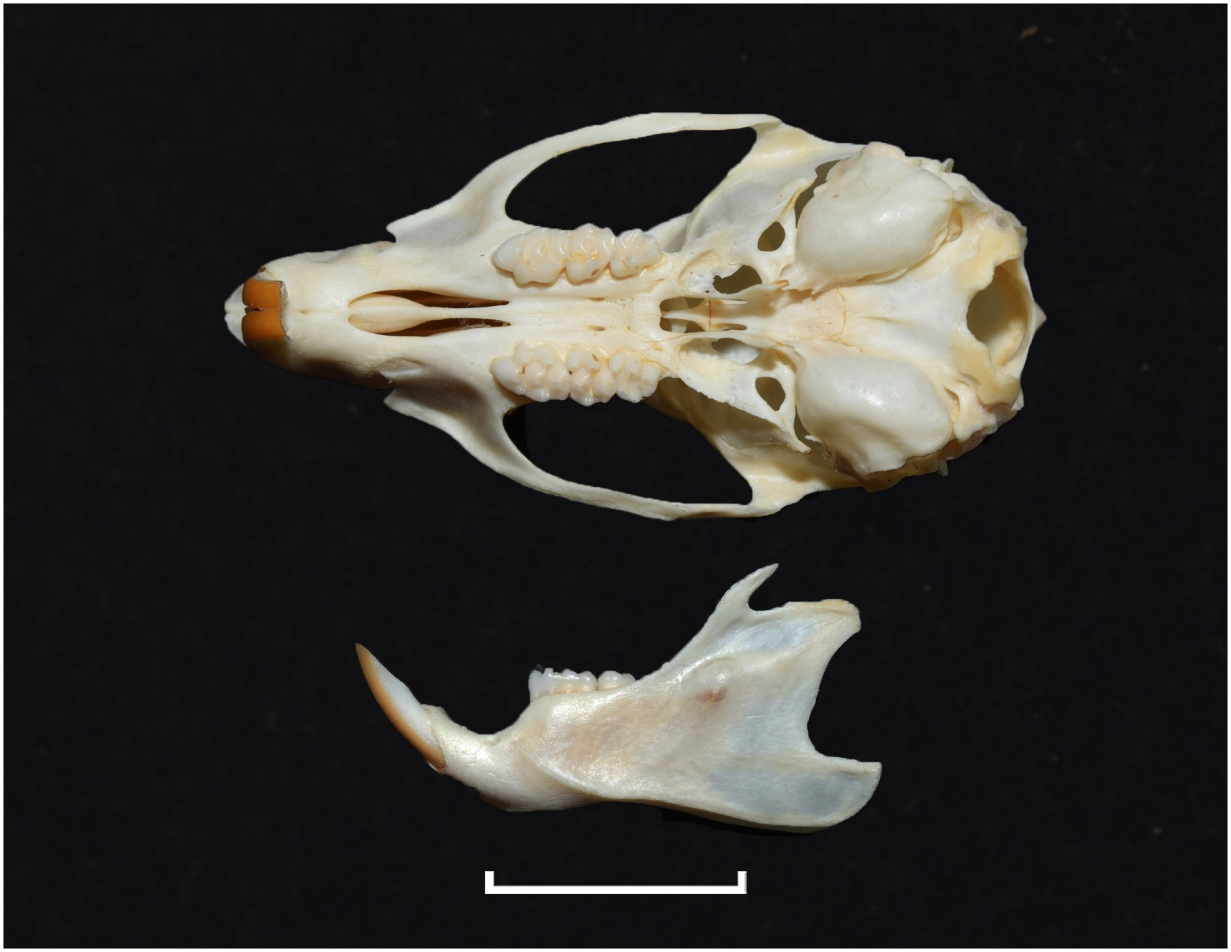
Cranium of *Lemniscomys rosalia* (IVB-RS3796), with scale bar of 1 cm.

**Fig 37:**
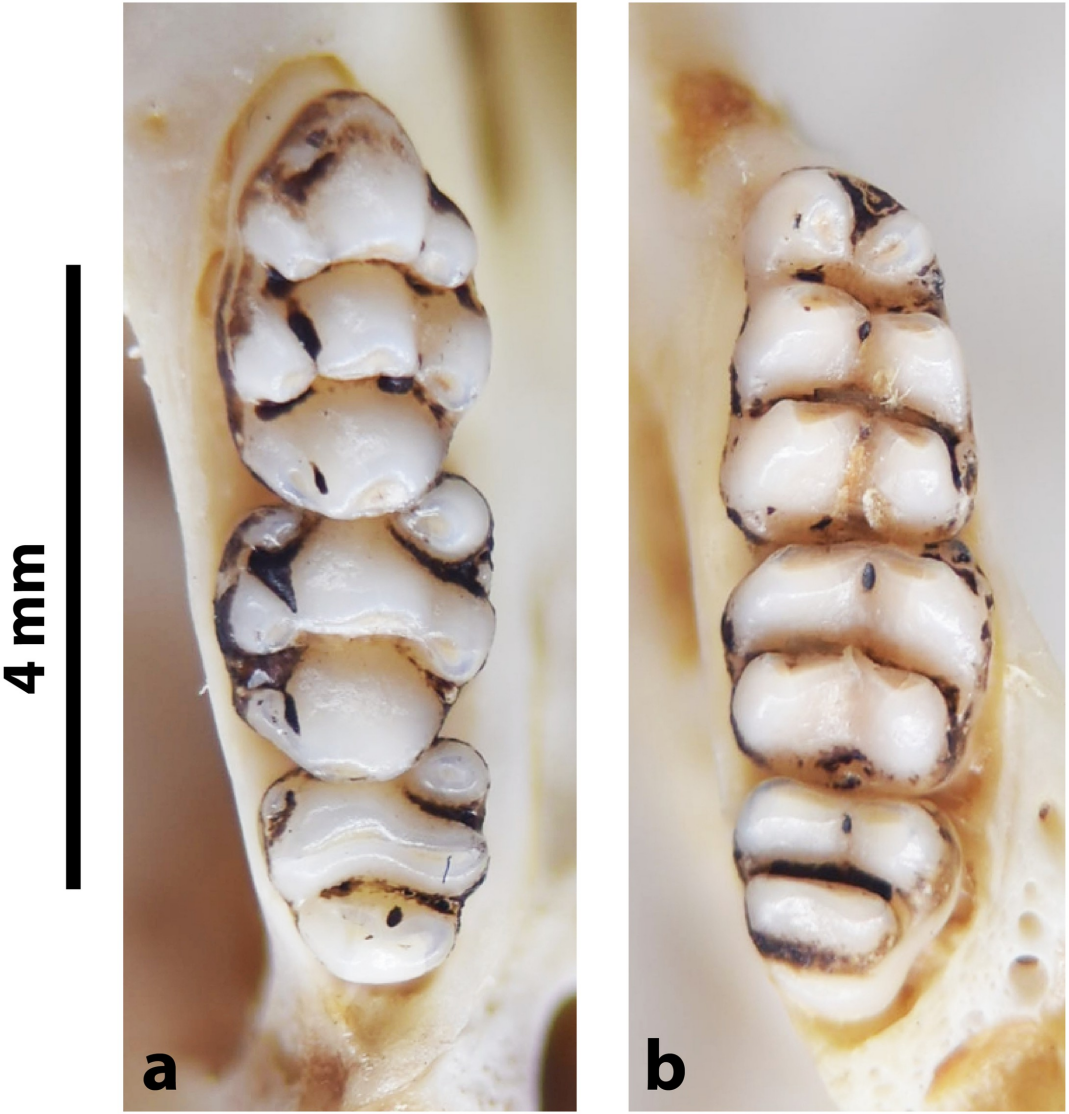
Upper (a) and lower (b) right toothrow of *L*. *rosalia* (DNMNH-29961).

**Fig 38:**
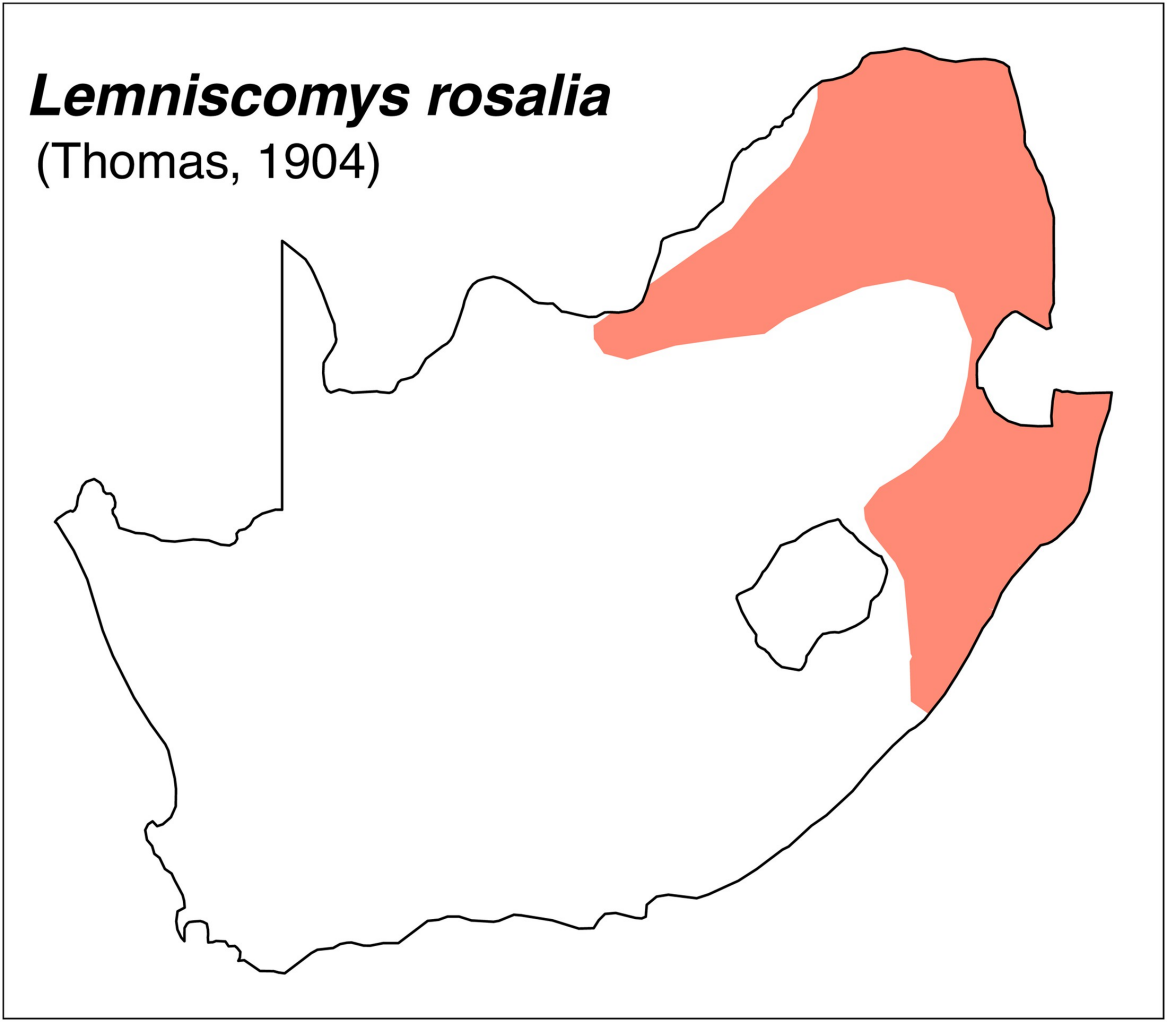
Distribution map.

Figs 36–38; Table 13

**Table 13:** Dental measurements (in mm) for *Lemniscomys rosalia*, sexes and species combined.

	Mean	Min	Max	n
LLTR	5.7	5.5	6.3	17
WM_1_	1.7	1.5	1.9	17
LUTR	5.9	5.5	6.5	17
WM^1^	1.9	1.8	2.2	17

Dental formula is 1-0-0-3:1-0-0-3. Alveolar formula is 5-5-4:5-5/6-3 (Figs 16 and 17).

#### Upper jaw

Incisors are opisthodont. The anterior palatal foramina generally reach the level of the t2 in M^1^. Molars are large, with broad central cusps (t2, t5, t8); this feature is mostly patent in the M^2^. In molar M^1^, the t4 is not connected to the t8 by a small crest as in *Aethomys* and is usually placed higher, sometimes in line with the cusp t5. There can be a small stephanodont crest uniting t6 and t9 on M^1^ and on M^2^. The M^3^ is relatively large, its distal lobe has two well differentiated cusps (as opposed to one or two fused cusps in *Aethomys* or *Micaelamys*); it belongs to Group 4 in Fig 12.

#### Lower jaw

The distinction between *Aethomys* and *Lemniscomys* based on the lower toothrow is sometimes not straightforward. In *Lemniscomys* the lateral cusplets are rarely well marked, while *Aethomys* often displays lateral cusplets or small ridges that can form some stephanodont crest. Molar size is overlapping between the two genera. As with *Micaelamys* and *Aethomys*, the M_1_ has no clear cingulum posterior. The M_2_ has two rootlets, resulting in six alveoli.

#### Systematic notes and South African fossil record

There is only one species recognized in South Africa:

- *Lemniscomys rosalia* (Thomas, 1904)

Fossils are known from the Early Pleistocene in various karst deposits from the Sterkfontein Valley [50].

Genus ***Mastomys*** Thomas, 1915 (Multimammate Mice)

**Fig 39:**
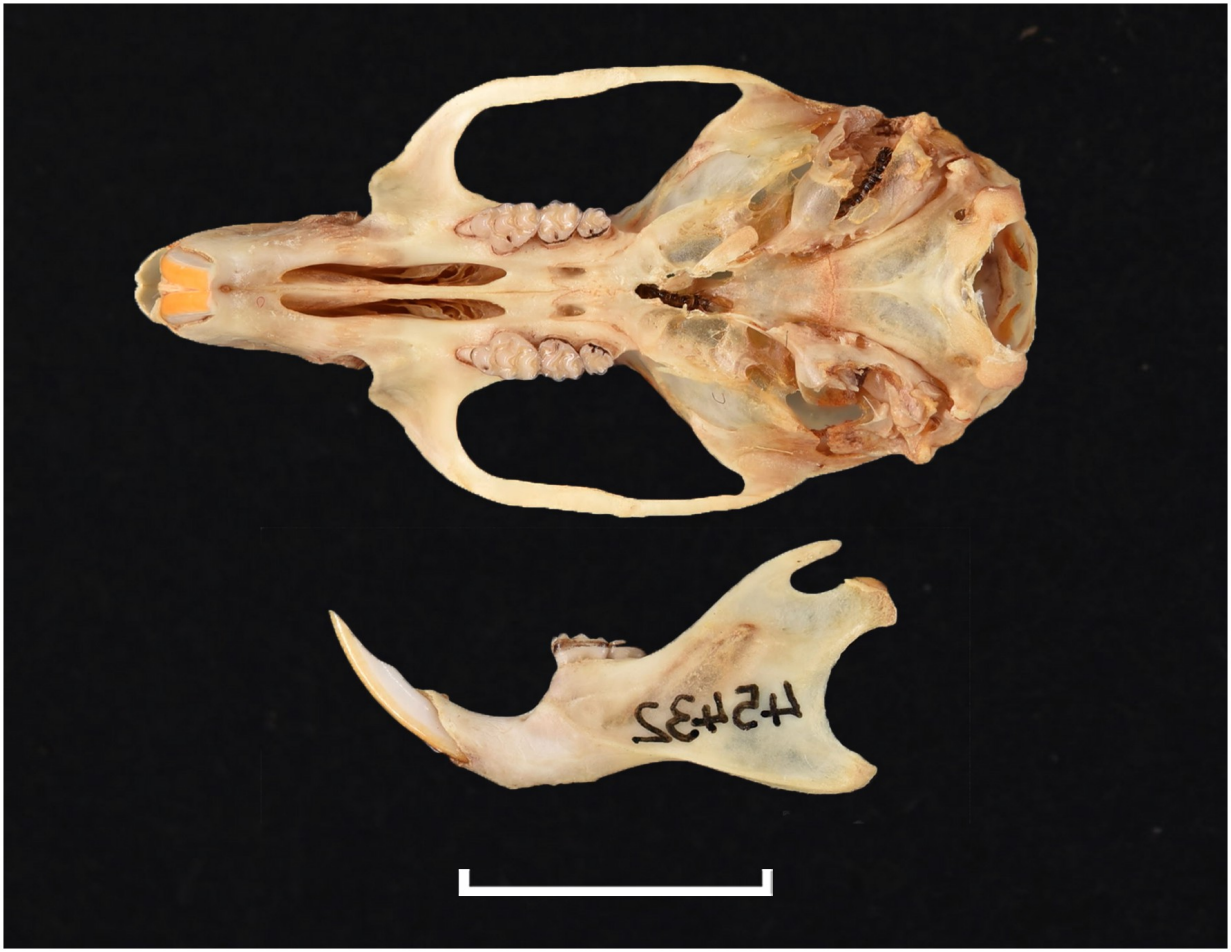
Cranium of *Mastomys coucha* (DNMNH-45432), with scale bar of 1 cm.

**Fig 40:**
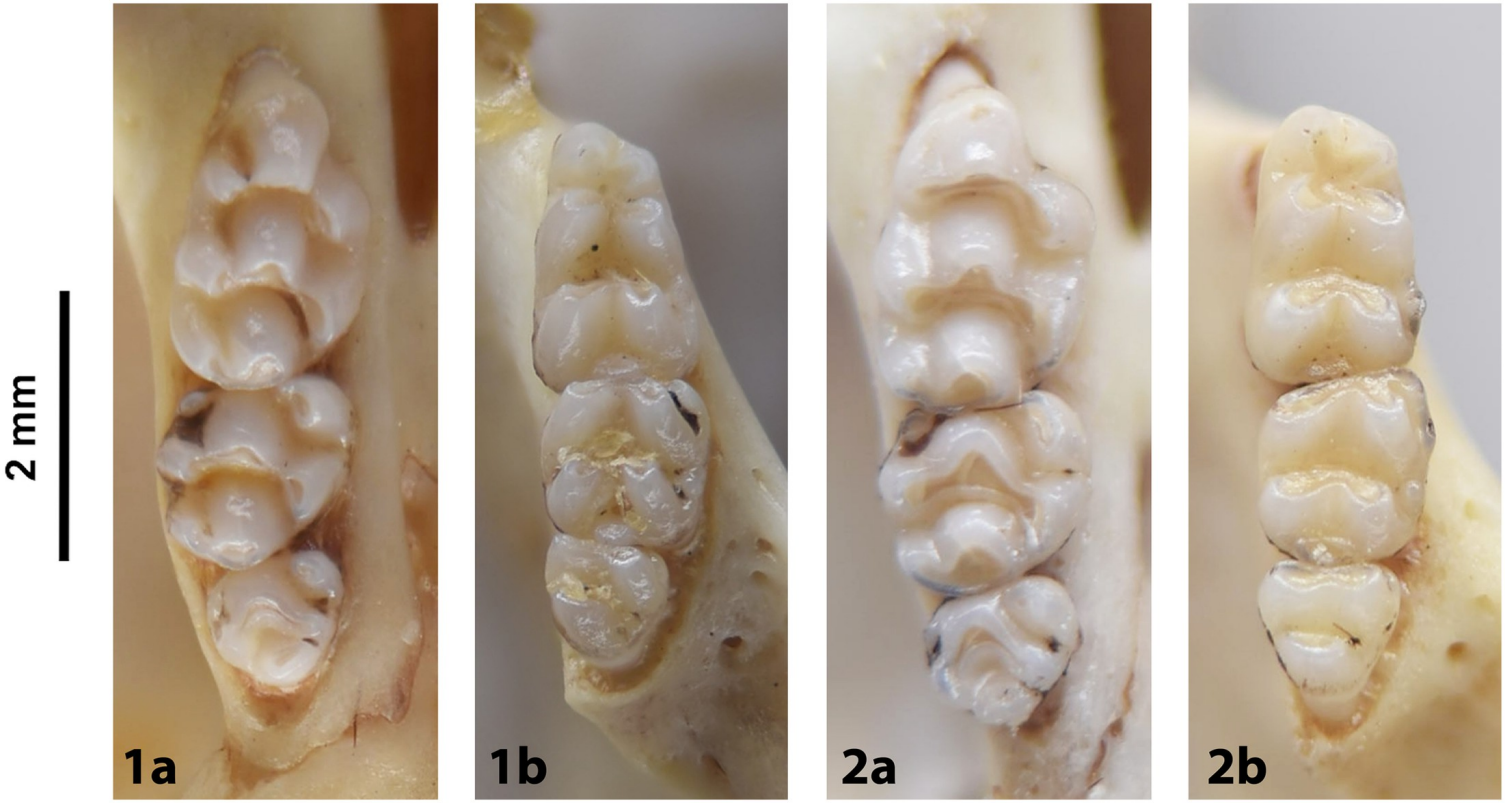
Cheekteeth of *Mastomys*. **1)** Upper (a) and lower (b) right toothrow of *M*. *coucha* (DNMNH-45433); **2)** Upper (a) and lower (b) right toothrow of *M*. *natalensis* (DNMNH-37511).

**Fig 41:**
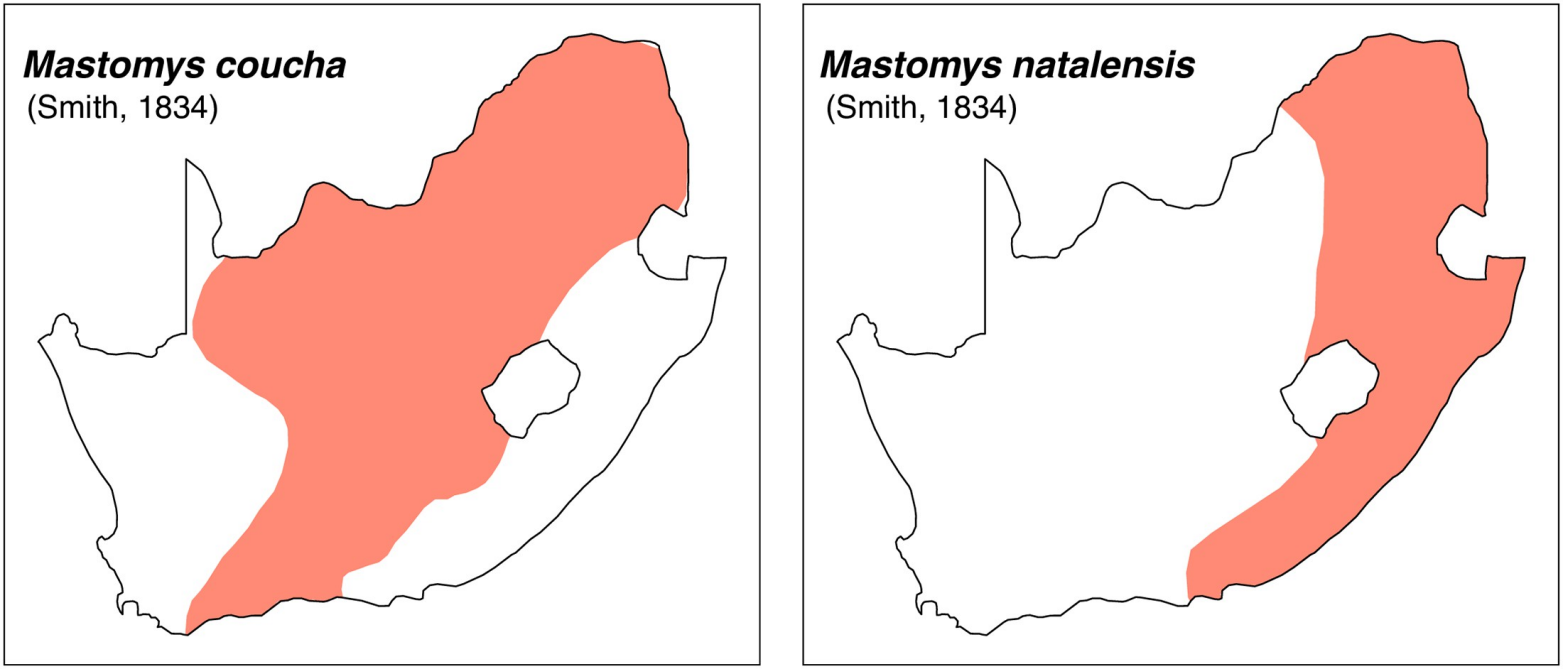
Distribution maps.

Figs 39–41; Table 14

**Table 14:** Dental measurements (in mm) for *Mastomys* from South Africa, sexes and species combined.

	Mean	Min	Max	n
LLTR	4.5	4.2	4.8	40
WM_1_	1.3	1.2	1.5	40
LUTR	4.8	4.4	5.2	40
WM^1^	1.6	1.4	1.8	40

Dental formula is 1-0-0-3:1-0-03. Alveolar formula is 3-3-2:2-2-2 (Figs 16 and 17).

#### Upper jaw

*Mastomys* is morphologically very similar to *Myomyscus* but has a much wider distribution. Upper incisors are smooth and orthodont. The anterior palatal foramina are long and reach the t1 of the M^1^. Rows of cusps are rather distorted in the M^1^, with the t1 located behind t2 and t3, and the t4 also located behind t5 and t6. As in *Myomyscus* and *Zelotomys* (two other members of the Praomyini tribe), the t9 is well separated from the t6, and located far in the labial side. The M^1^ has three roots. The M^2^ has a t1 and a small t3, and the t9 is low and well distinct. The M^3^ has two lobes and corresponds to Group 3 in Fig 12.

#### Lower jaw

Lower incisors are smooth. Molars show a typical Praomyini pattern (as *Myomyscus* and *Zelotomys*). Both M_1_ and M_2_ have a posterior cingulum. The M_1_ displays a small posterior cingulum and a posterolabial cusplet (plc). The M_2_ has both anterolabial and posterolabial cusplets. The mandible is approximately the same size as in *Rhabdomys*, but both genera can be distinguished based on the number of alveoli when teeth are absent (alveolar formula is 2-2-2 in *Mastomys*, 4-5-3 in *Rhabdomys*). As in *Myomyscus* and *Zelotomys*, the alveolar region of the mandible is well-developed in proportion to the rest of the mandible.

#### Systematic notes and South African fossil record

*Mastomys* was previously included in *Praomys*, but is now placed in its own genus [22]. Two species are currently recognized in South Africa:

- *Mastomys coucha* (Smith, 1834)
- *Mastomys natalensis* (Smith, 1834)

Fossils of *Mastomys* have been recovered from many Pleistocene fossil deposits from South Africa. Most of this material is attributed to *M*. *natalensis*, but the remains were identified at a time when *M*. *coucha* was not recognized. These two species appear undistinguishable using cranio-dental features [54], so specimens from the Early Pleistocene should be conservatively attributed to *Mastomys* sp.

Genus ***Micaelamys*** Ellerman, 1941 (Lesser Veld Rats)

**Fig 42:**
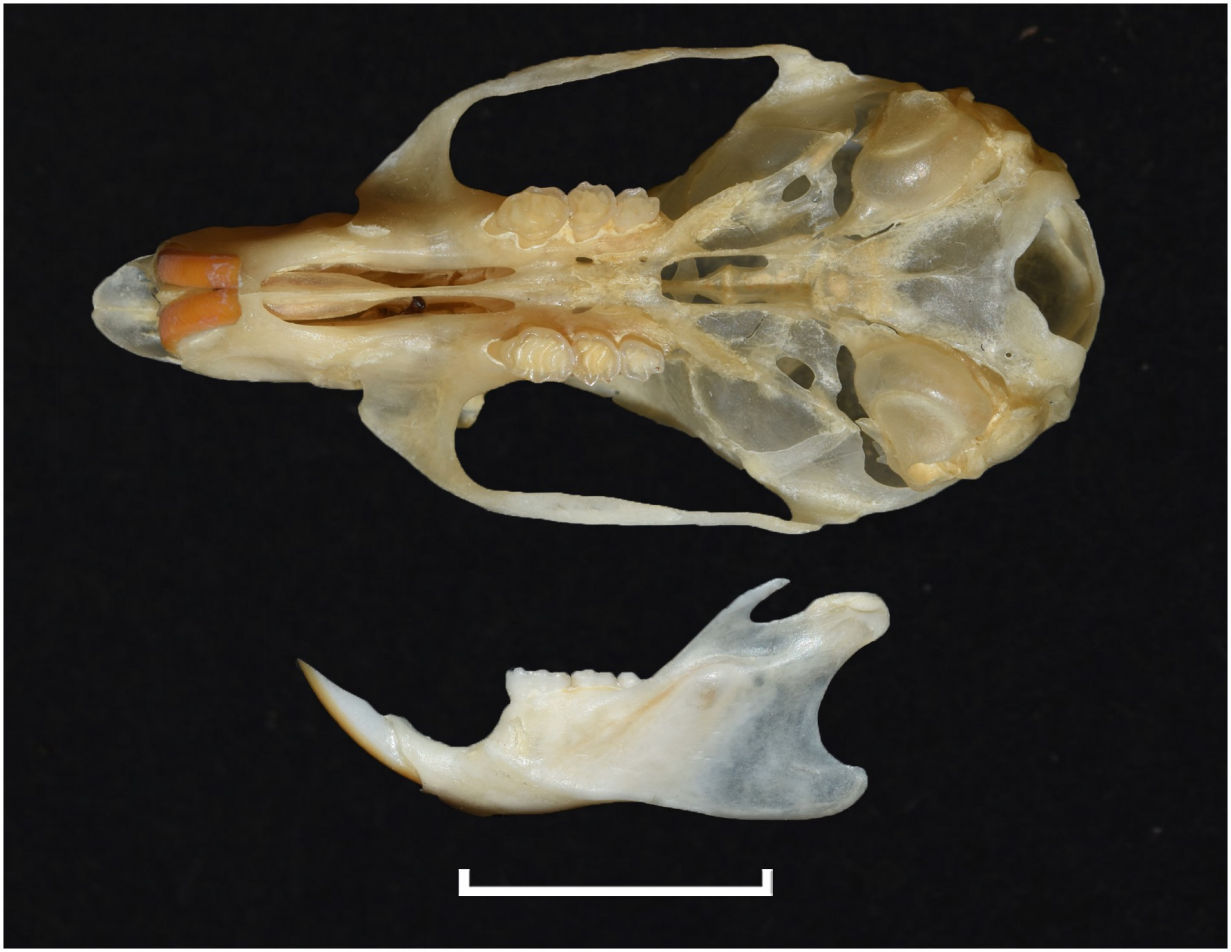
Cranium of *Micaelamys namaquensis* (IVB-M4883), with scale bar of 1 cm.

**Fig 43:**
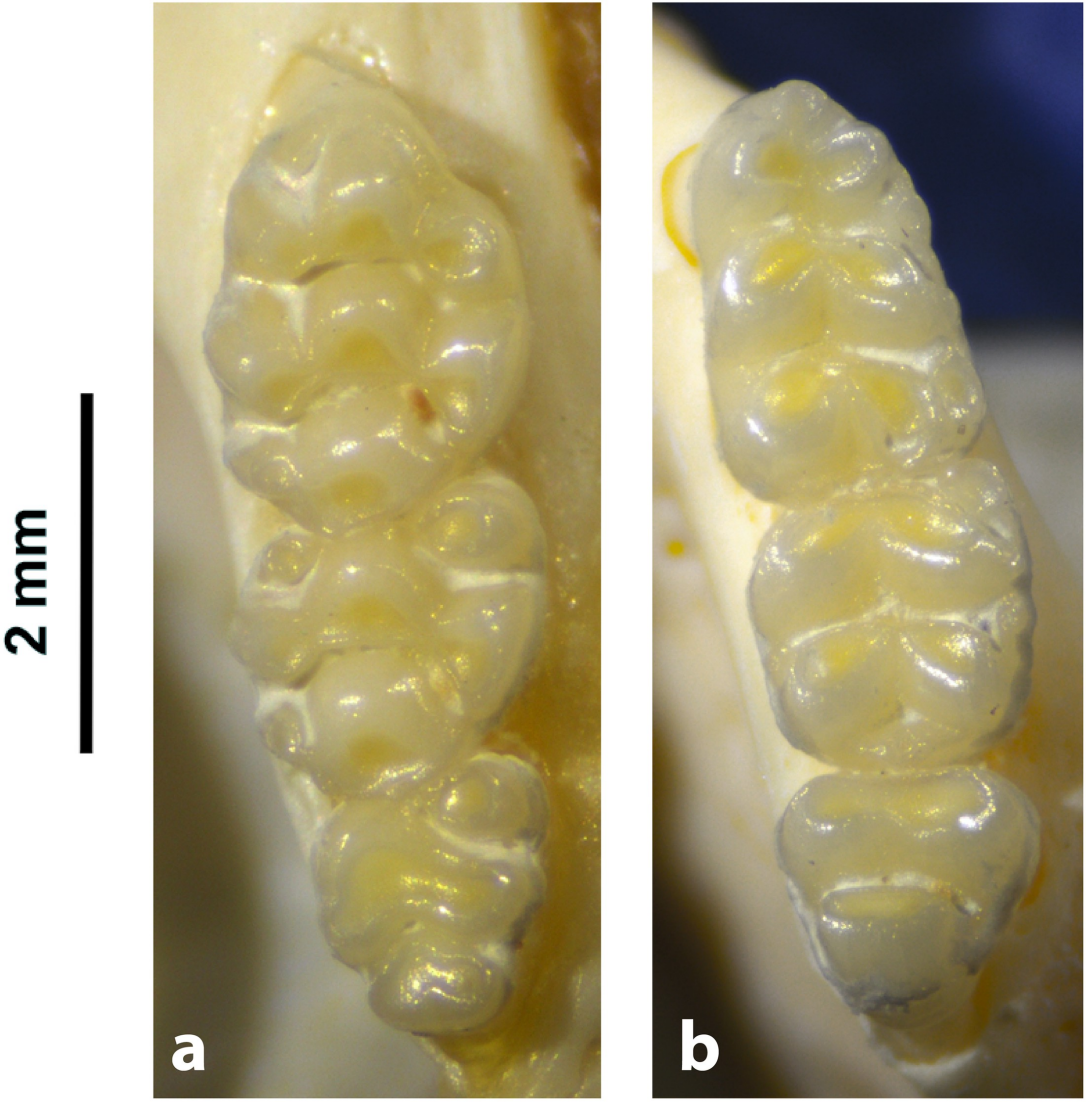
Upper (a) and lower (b) right toothrow of *M*. *namaquensis* (MNHN-ZM-MO-1990-323).

**Fig 44:**
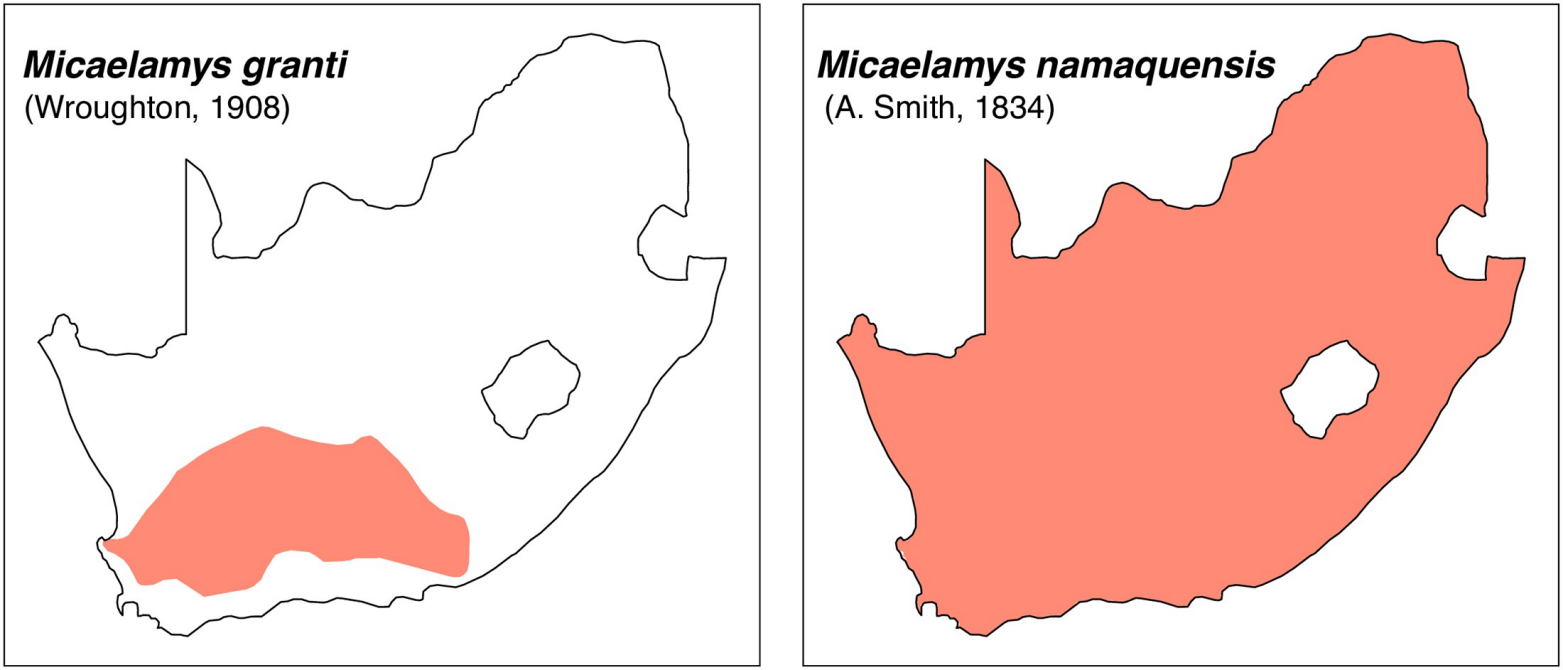
Distribution maps.

Figs 42–44; Table 15

**Table 15:** Dental measurements (in mm) for *Micaelamys namaquensis*, sexes and species combined.

	Mean	Min	Max	n
LLTR	5.0	4.7	5.3	22
WM_1_	1.5	1.3	1.8	22
LUTR	5.2	4.9	5.5	22
WM^1^	1.7	1.6	1.9	22

Dental formula is 1-0-0-3:1-0-0-3. Alveolar formula is 4/5-3/4-3:3-3-3 (Figs 16 and 17).

#### Upper jaw

Incisors are opisthodont. In the M^1^, cusps are few distorted with t1 slightly behind t2 and t3, and t4 behind t5 and t6. There are sometimes traces of stephanodonty. The M^3^ lacks the t3 its distal lobe has one elongated cusp (or 2 very few differentiated, as opposed to two well differentiated in *Lemniscomys*); it corresponds to Group 3 in Fig 12. Molars are smaller than in *Aethomys* and *Lemniscomys*.

#### Lower jaw

M_1_ has always three cusps in the anterior lobe (presence of a well-developed tma), as opposed to two in most specimens of *Aethomys* (some *Aethomys* display a very small anteromedian cusplet). As *Aethomys* and *Lemniscomys*, the M_1_ has no posterior cingulum or a very small one in few specimens. Similarly, the M_2_ has a very small pc contrary to *Aethomys* where there is often a round median pc. A row of additional cusplets often occurs on the labial side of the M_1_ and M_2_.

#### Systematic notes and South African fossil record

Species of this genus were previously included within *Aethomys* but are now placed in their own genus based on morphological and molecular ground [28, 52, 55]. Two species are currently recognized in South Africa:

- *Micaelamys granti* (Wroughton, 1908)
- *Micaelamys namaquensis* (A. Smith, 1834)

The genus *Micaelamys* has been identified in many Pleistocene deposits in South Africa, often referred to as *Aethomys* (for instance, *Aethomys namaquensis*). The oldest *Aethomys* cf. *namaquensis* has been described by Pocock [48] in Makapansgat.

Genus ***Mus*** Linnaeus, 1758 (Old World Mice and Pygmy Mice)

**Fig 45:**
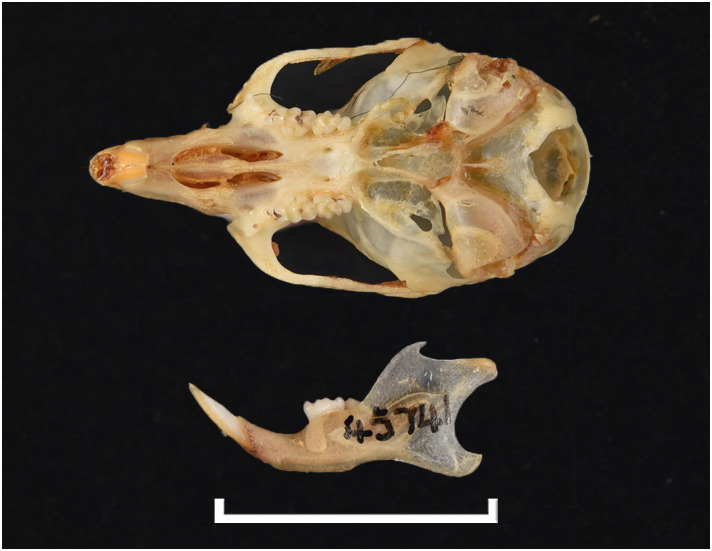
Cranium of *Mus indutus* (DNMNH-45741), with scale bar of 1 cm.

**Fig 46:**
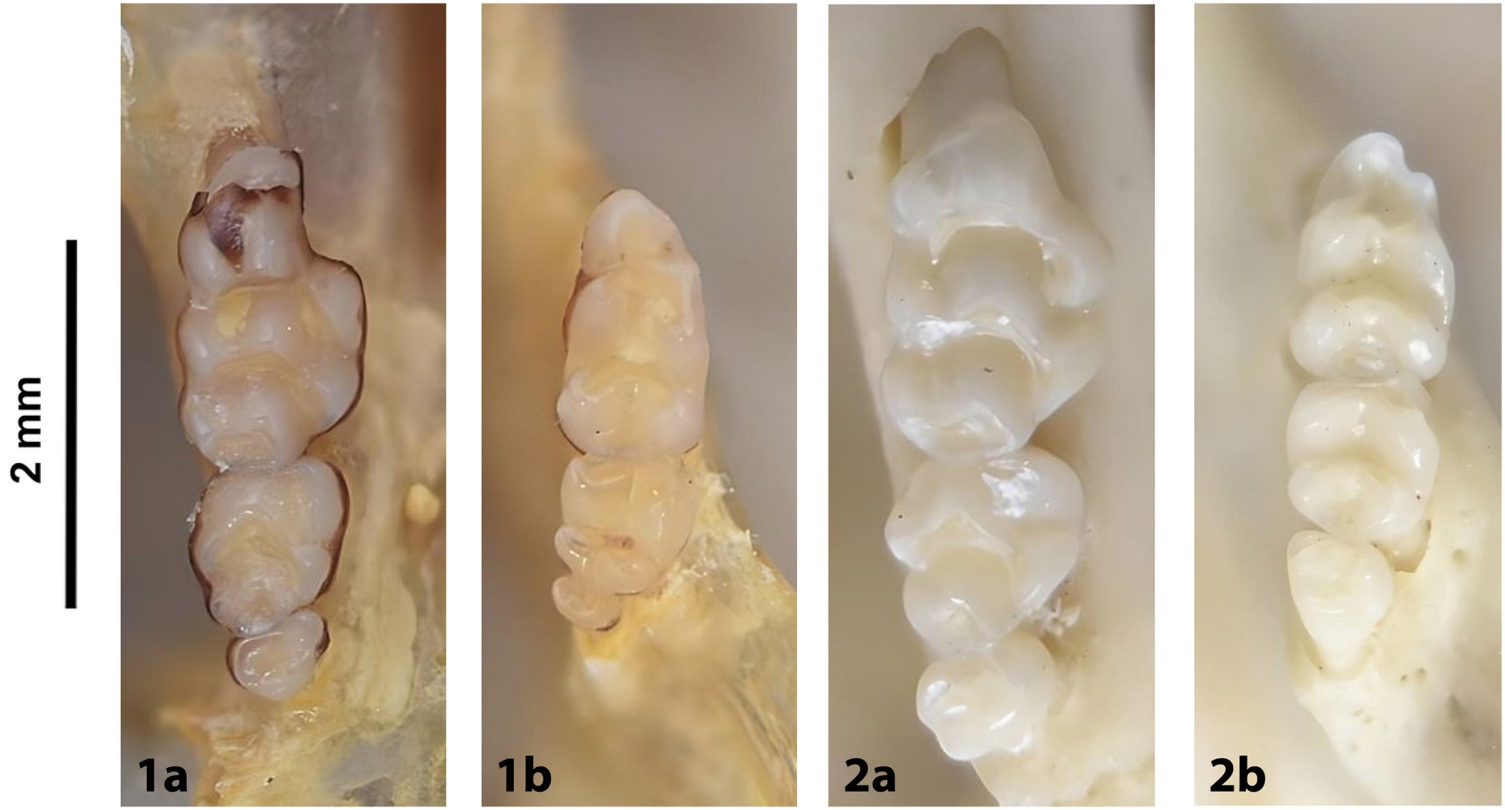
Cheekteeth of *Mus*. **1)** Upper (a) and lower (b) right toothrow of *M*. *indutus* (DNMNH-45737); **2)** Upper (a) and lower (b) right toothrow of *M*. *musculus* (MNHN-ZM-MO-1994-2350).

**Fig 47:**
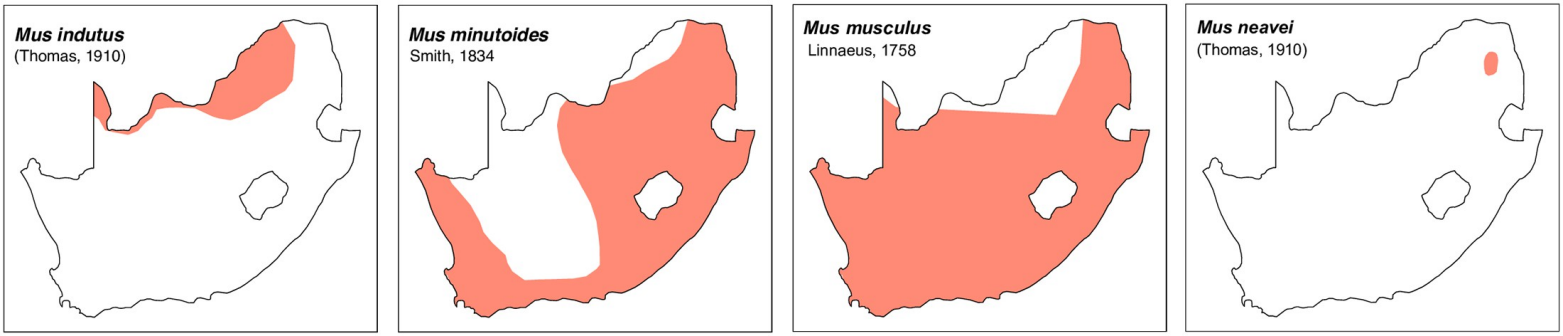
Distribution maps.

Figs 45–47; Table 16

**Table 16:** Dental measurements (in mm) for *Mus* from South Africa, sexes and species combined.

	Mean	Min	Max	n
LLTR	2.6	2.2	3.2	72
WM_1_	0.9	0.7	1.1	72
LUTR	3.1	2.6	3.4	71
WM^1^	1.0	0.9	1.4	71

Dental formula is 1-0-0-3:1-0-0-3. Alveolar formula is 3-3-1/2:2-2/3-1/2 (Figs 16 and 17).

#### Upper jaw

Upper incisors are ungrooved and opisthodont, with the worn posterior surface characteristically notched for the three South African species. The palatal foramina reach the first or the second row of cusps in M^1^. The skull is small, and so are the molars. The cusps on M^1^ are distorted, with t1 situated far posterior of t2 and t3. Molar M^3^ is small with a reduced cusp configuration: it has a t1, but cusps are poorly differentiated and there is a link between first and second lobe (Group 7 in Fig 12). Presence of a masseteric knob.

#### Lower jaw

The M_1_ has a typically enlarged lingual anteroconid that distorts the tooth, while the labial anteroconid is smaller and close to the protoconid. The M_3_ is tiny and has one or two lobes with one or two corresponding alveoli (always two lobes and two alveoli in *Acomys*). The mandible is small, being of the same average size as *Dendromus*; the M_2_ is often 3-rooted in *Mus* whereas it is 2-rooted in *Dendromus*.

#### Systematic notes and South African fossil record

Four species are currently recognized in South Africa:

- *Mus indutus* (Thomas, 1910)
- *Mus minutoides* Smith, 1834
- *Mus musculus* Linnaeus, 1758
- *Mus neavei* (Thomas, 1910)

*M*. *musculus* belongs to the subgenus *Mus*, while *M*. *indutus*, *M*. *minutoides* and *M*. *neavei* belong to the subgenus *Nannomys*, formerly named *Leggada* [e.g., 24, 56]. The genus *Mus* is known from the Pliocene of Makapansgat [48] and has been recovered from many Pleistocene sites. The species *M*. *musculus* was introduced and is thus not found in the Pleistocene deposits. The oldest and single record of *M*. *musculus* in Holocene deposits is from Hope Hill Shelter dated to 4,400±100 B.P. [57]

Genus ***Myomyscus*** Shortridge, 1942 (Meadow Mice)

**Fig 48:**
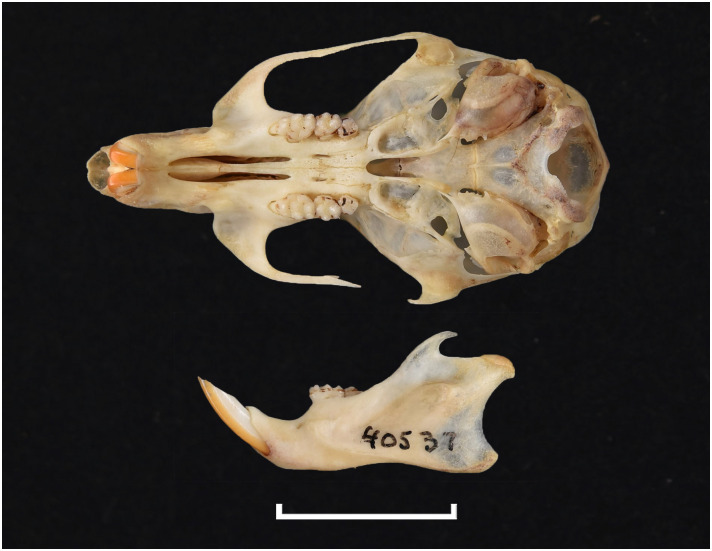
Cranium of *Myomyscus verreauxii* (DNMNH-40537), with scale bar of 1 cm.

**Fig 49:**
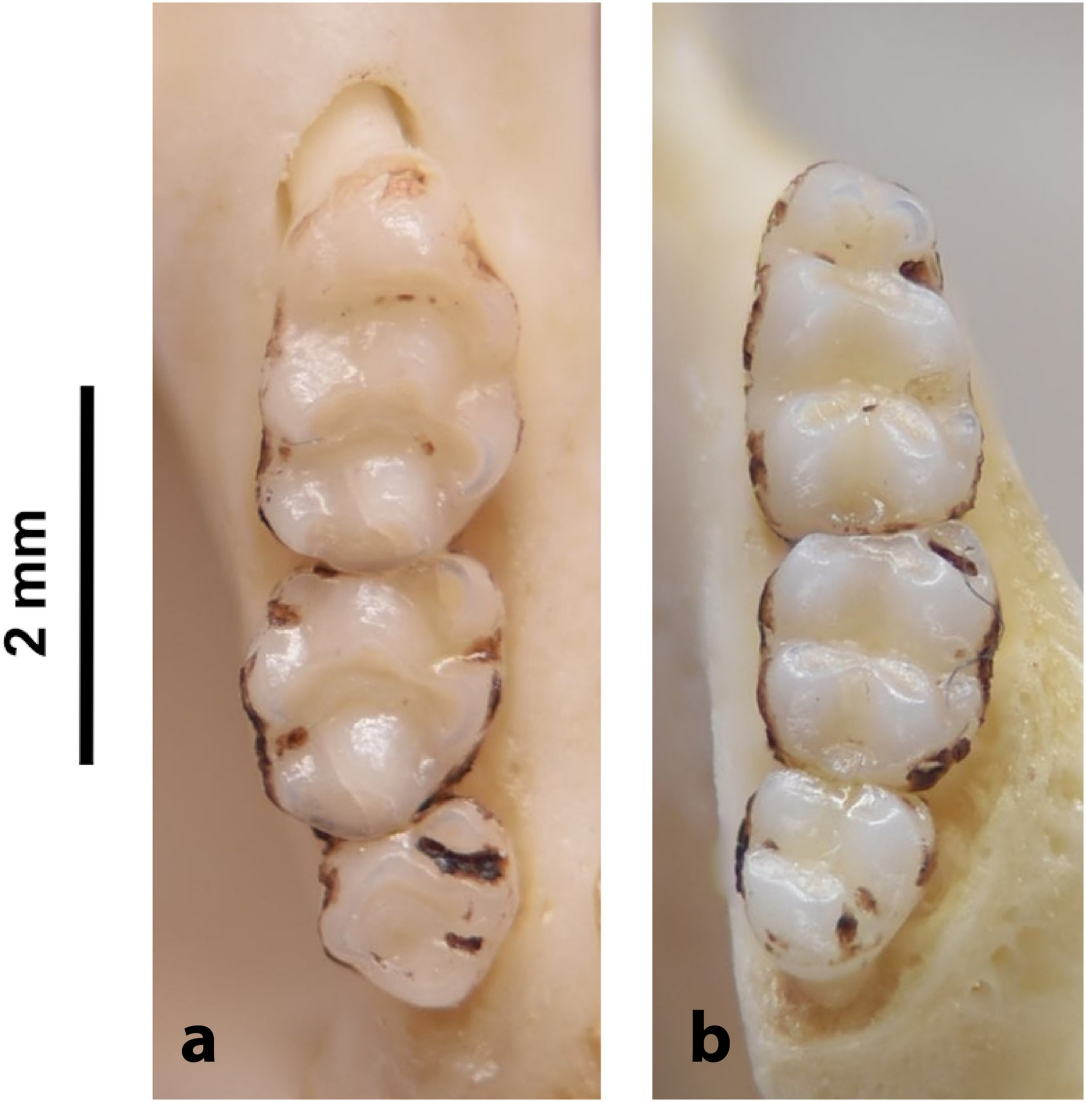
Upper (a) and lower (b) right toothrow of *M*. *verreauxii* (DNMNH-40537).

**Fig 50:**
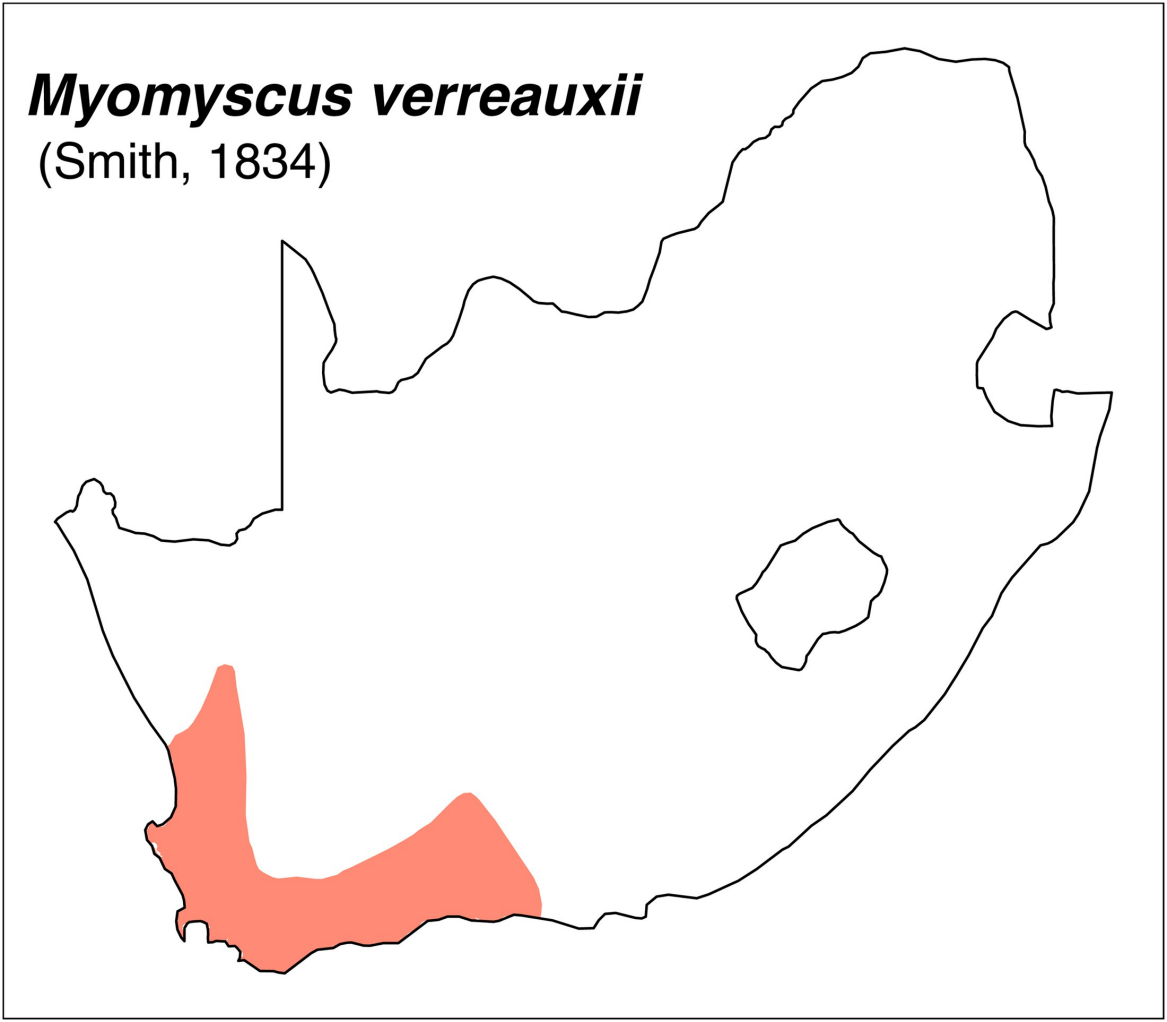
Distribution map.

Figs 48–50; Table 17

**Table 17:** Dental measurements (in mm) for *Myomyscus verreauxii*, sexes combined.

	Mean	Min	Max	n
LLTR	4.5	4.2	4.7	3
WM_1_	1.2	1.2	1.3	3
LUTR	4.7	4.5	5.0	3
WM^1^	2.4	1.4	1.5	3

Dental formula is 1-0-0-3:1-0-0-3. Alveolar formula is 3-3-2:2-2-2 (Figs 16 and 17).

#### Upper jaw

*Myomyscus* is morphologically very similar to *Mastomys* and is mostly diagnosable by its restricted distribution to the Fynbos biome. The anterior palatal foramina reach the t1 of the M^1^. The rows of cusps are slightly distorted in the M^1^, with the t1 located behind t2 and t3, and the t4 also located behind t5 and t6. As with other members of the Praomyini tribe, the t9 is well separated from the t6, and located far in the labial side. The M^1^ has three roots. The M^2^ has a t1 and a small t3, and the t9 is low and well distinct. Like in *Mastomys*, the M^3^ is large with a t1 and a t3 and two lobes and corresponds to Group 3 in Fig 11.

#### Lower jaw

As in *Mastomys*, molars show a typical Praomyini pattern. Both M_1_ and M_2_ have a posterior cingulum. The M_1_ displays a small posterior cingulum and a posterolabial cusplet (plc). The M_2_ has both anterolabial and posterolabial cusplets. The mandible is of average size.

#### Systematic notes and South African fossil record

A single species is recognized in South Africa:

- *Myomyscus verreauxii* (Smith, 1834)

In the past, this species had been placed in the genera *Praomys* and *Myomys* [22]. In South Africa, remains of *Myomyscus* are known since the Late Pliocene [50, 58].

Genus ***Otomys*** Cuvier, 1824 (Vlei Rats or Laminate-toothed Rats)

**Fig 51:**
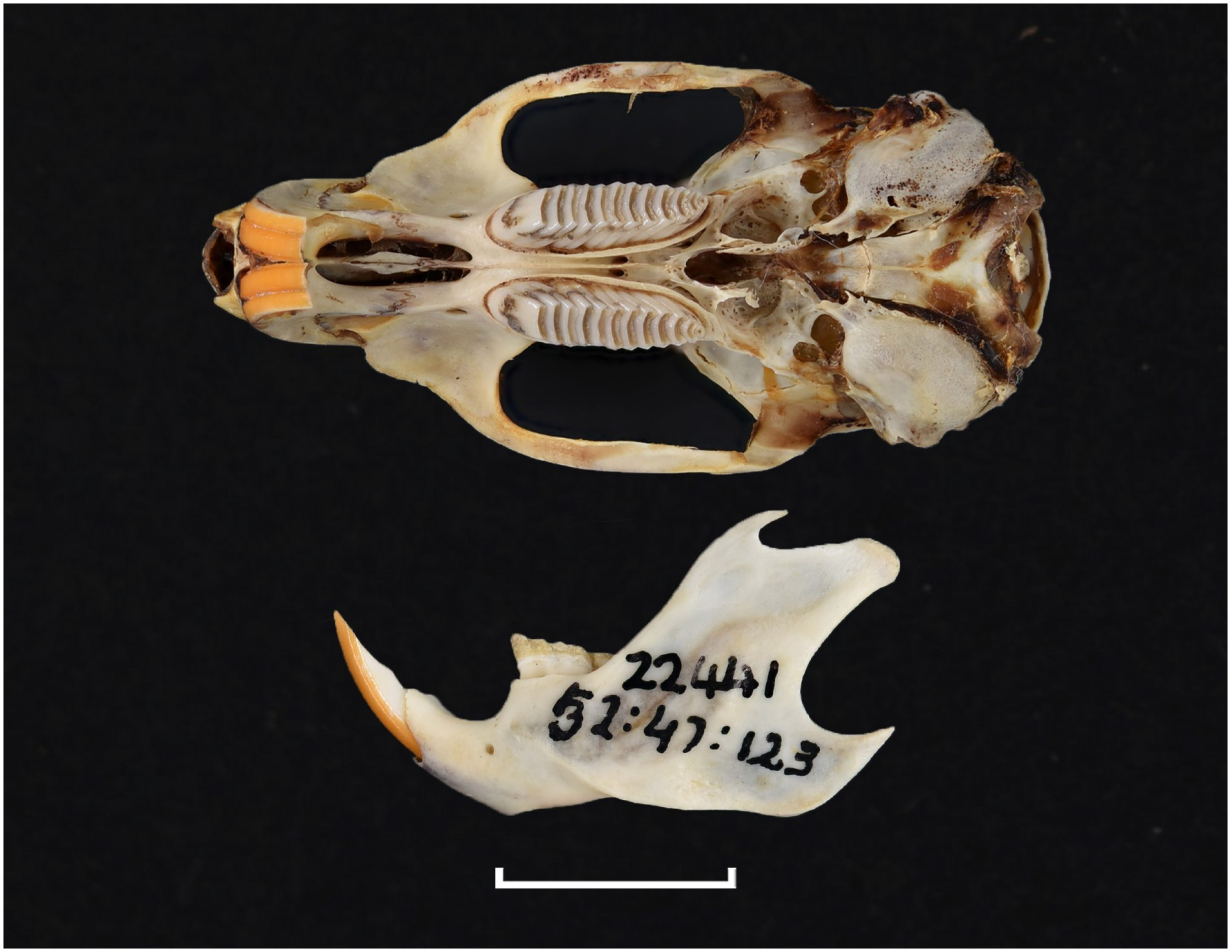
Cranium of *Otomys angoniensis* (DNMNH-27526), with scale bar of 1 cm.

**Fig 52:**
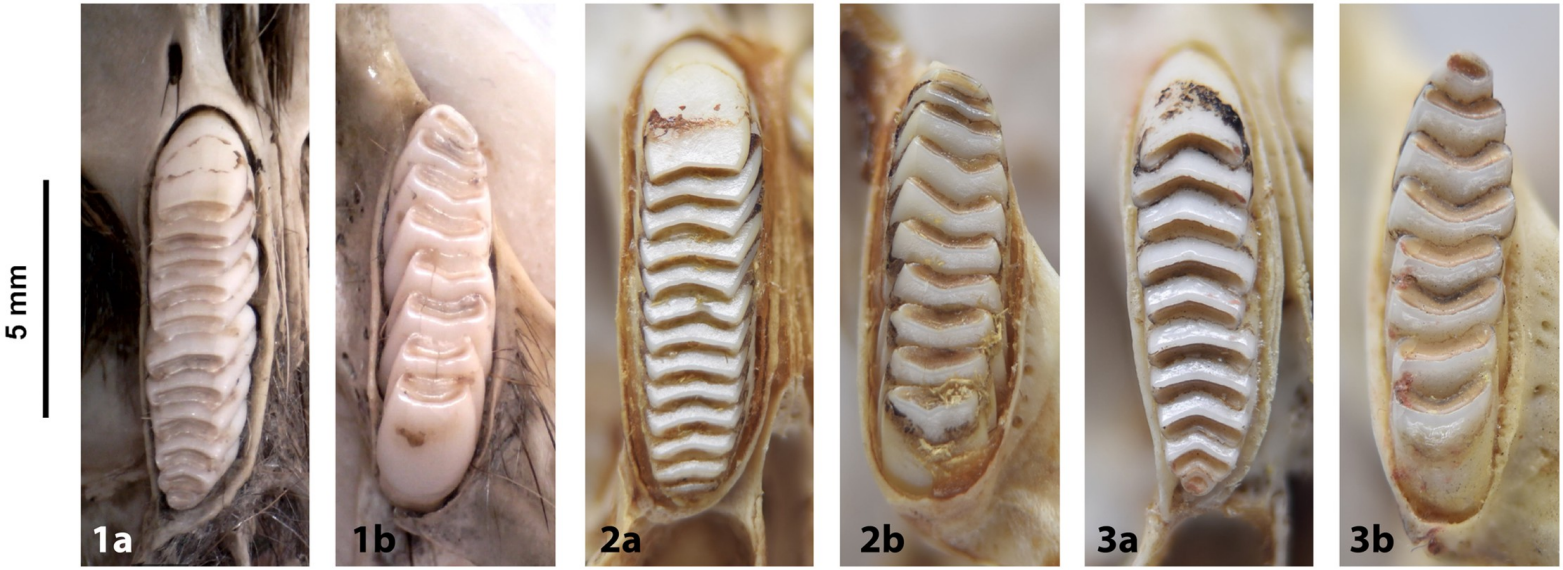
Cheekteeth of *Otomys*. **1)** Upper (a) and lower (b) right toothrow of *O*. *angoniensis* (MNHN- ZM-2020-574 BO24); **2)** Upper (a) and lower (b) right toothrow of *O*. *laminatus* (DNMNH-4647); **3)** Upper (a) and lower (b) right toothrow of *O*. *sloggetti* (DNMNH-7781).

**Fig 53:**
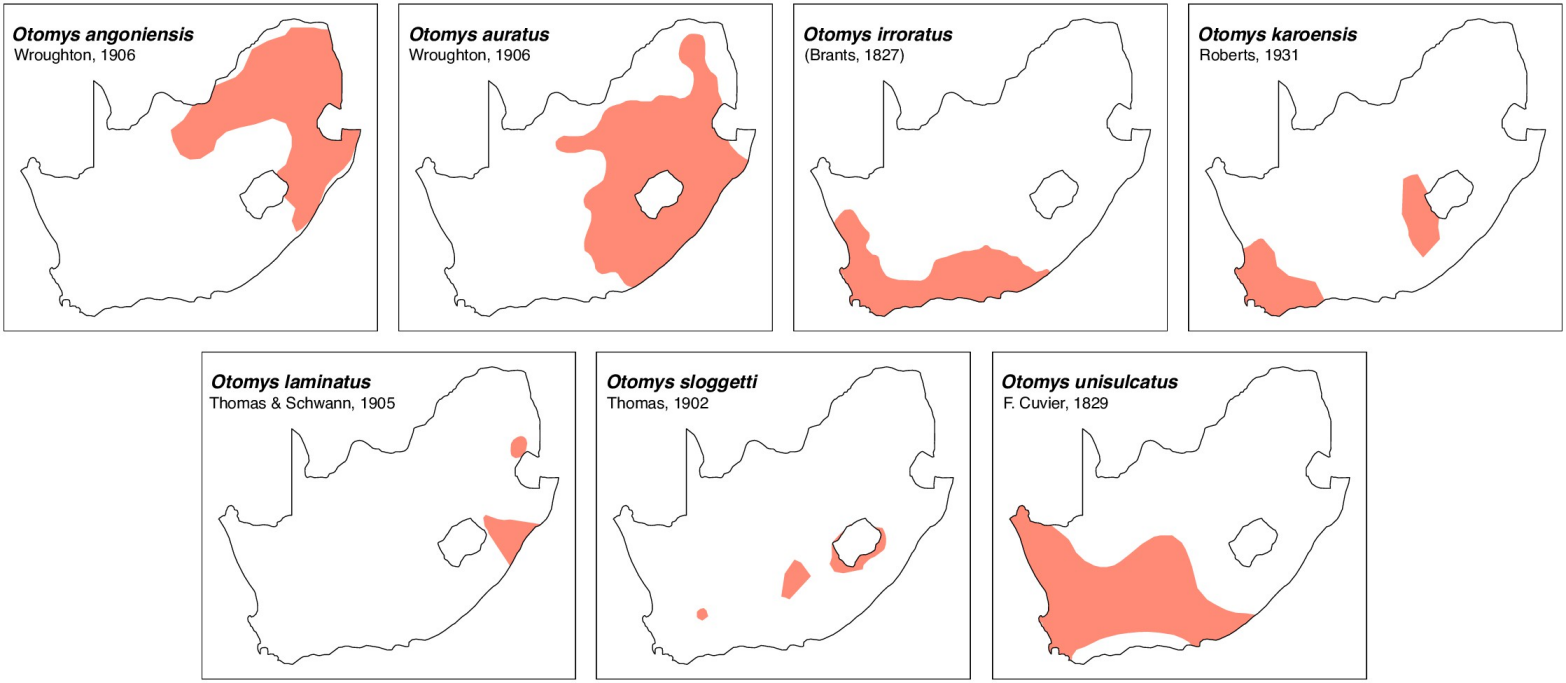
Distribution maps.

Figs 51–53; Table 18

**Table 18:** Dental measurements (in mm) for *Otomys* from South Africa, sexes and species combined.

	Mean	Min	Max	n
LLTR	8.7	7.6	11.1	70
WM_1_	2.4	1.9	3.0	70
LUTR	9.4	7.7	11.1	69
WM^1^	2.5	2.1	3.8	69

Dental formula is 1-0-0-3:1-0-0-3. Alveolar formula is variable, for instance 3-3-11:7-4-3 (Figs 16 and 17).

#### Upper jaw

Incisors are opisthodont and always grooved (the number of grooves varies by species). The anterior palatal foramina rarely reach the M^1^. The palate is very narrow with broad region of contact between the two halves. The cusps of the cheekteeth are fused into several transverse laminae: M^1^ has three laminae; M^2^ has two; M^3^ is the longest cheekteeth, having four or more laminae. The auditory bullae are hardly, or not, inflated.

#### Lower jaw

Lower incisors have one deep or faint groove, or no groove at all. The M_1_ is the largest molar of the toothrow, having between three (*O*. *unisulcatus*) and seven (*O*. *laminatus*) laminae. The M_2_ and the M_3_ have two laminae each. The mandible is stocky with a broad ascending ramus.

#### Systematic notes and South African fossil record

There has been debate about the position of *Otomys* within its own subfamily Otomyinae, but most recent phylogenetic analyses nest it within the Murinae [28, 55]. There has also been debate about the genus boundaries of *Otomys*, *Parotomys*, and *Myotomys*. The latter genus, which comprised *M*. *unisulcatus* and *M*. *sloggetti*, has been proved paraphyletic [59]. For this reason, only the two genera, *Otomys* and *Paratomys*, are currently recognized [22]. Recent systematic analyses have revealed multiple cryptic species, which resulted in a considerable increase in the total number of species currently recognized in South Africa:

- *Otomys angoniensis* Wroughton, 1906
- *Otomys auratus* Wroughton, 1906
- *Otomys irroratus* (Brants, 1827)
- *Otomys karoensis* Roberts, 1931
- *Otomys laminatus* Thomas & Schwann, 1905
- *Otomys sloggetti* Thomas, 1902
- *Otomys unisulcatus* F. Cuvier, 1829

Further taxonomic investigation is required for this genus. Additional fossil species have been described from the fossil deposits of South Africa:

- *†Myotomys campbelli* Broom and Schepers, 1946 from the Pliocene deposit of Taung
- *†Otomys gracilis* Broom, 1937 identified in many Pleistocene karstic deposits from the Sterkfontein Valley

The fossil species *†O*. *gracilis* is regarded by Avery [4] as a synonym of modern *O*. *saundersiae*. The Otomys fossils need to be revised in light of recent advancements in systematic research.

Genus ***Parotomys*** Thomas, 1918 (Whistling Rats)

**Fig 54:**
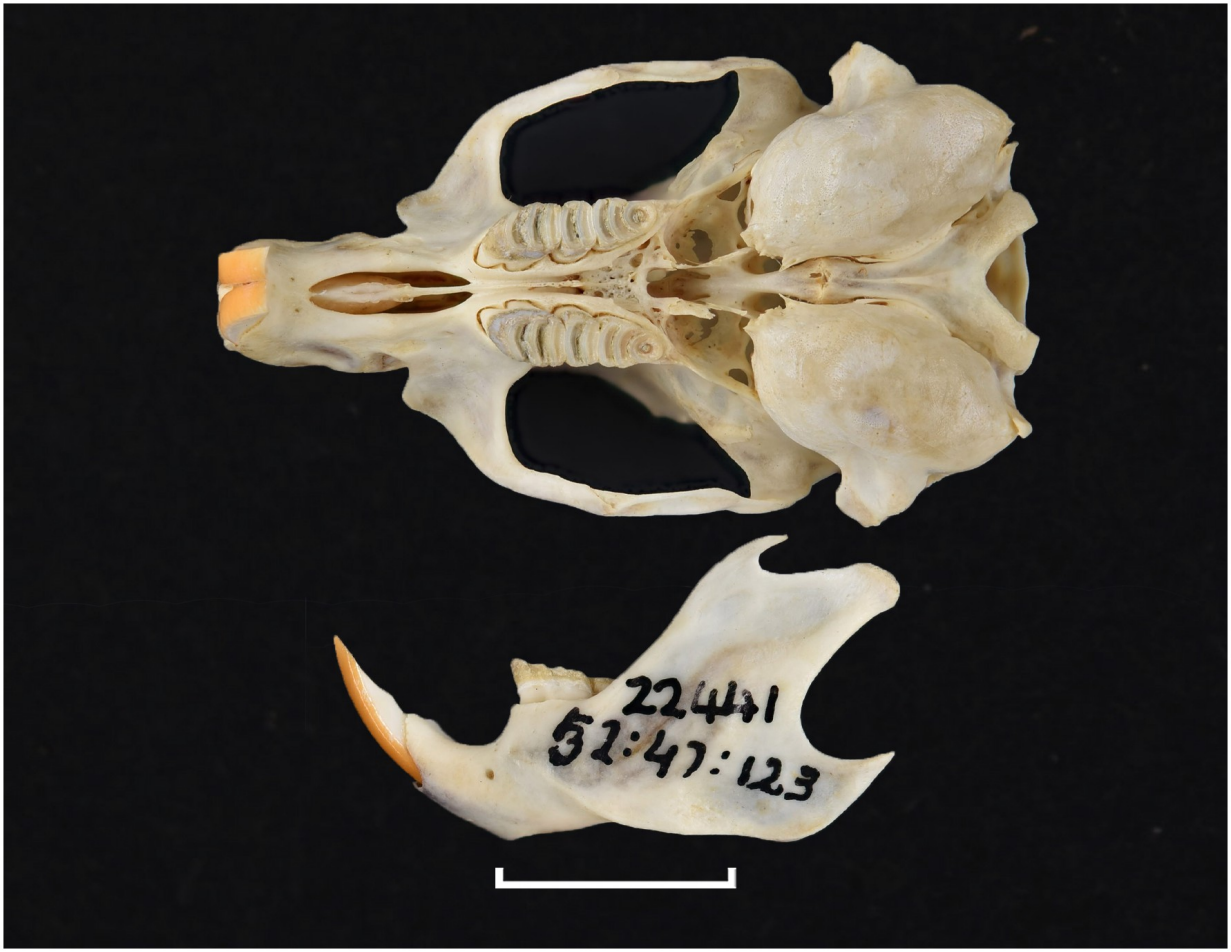
Cranium of *Parotomys littledalei* (DNMNH-22441), with scale bar of 1 cm.

**Fig 55:**
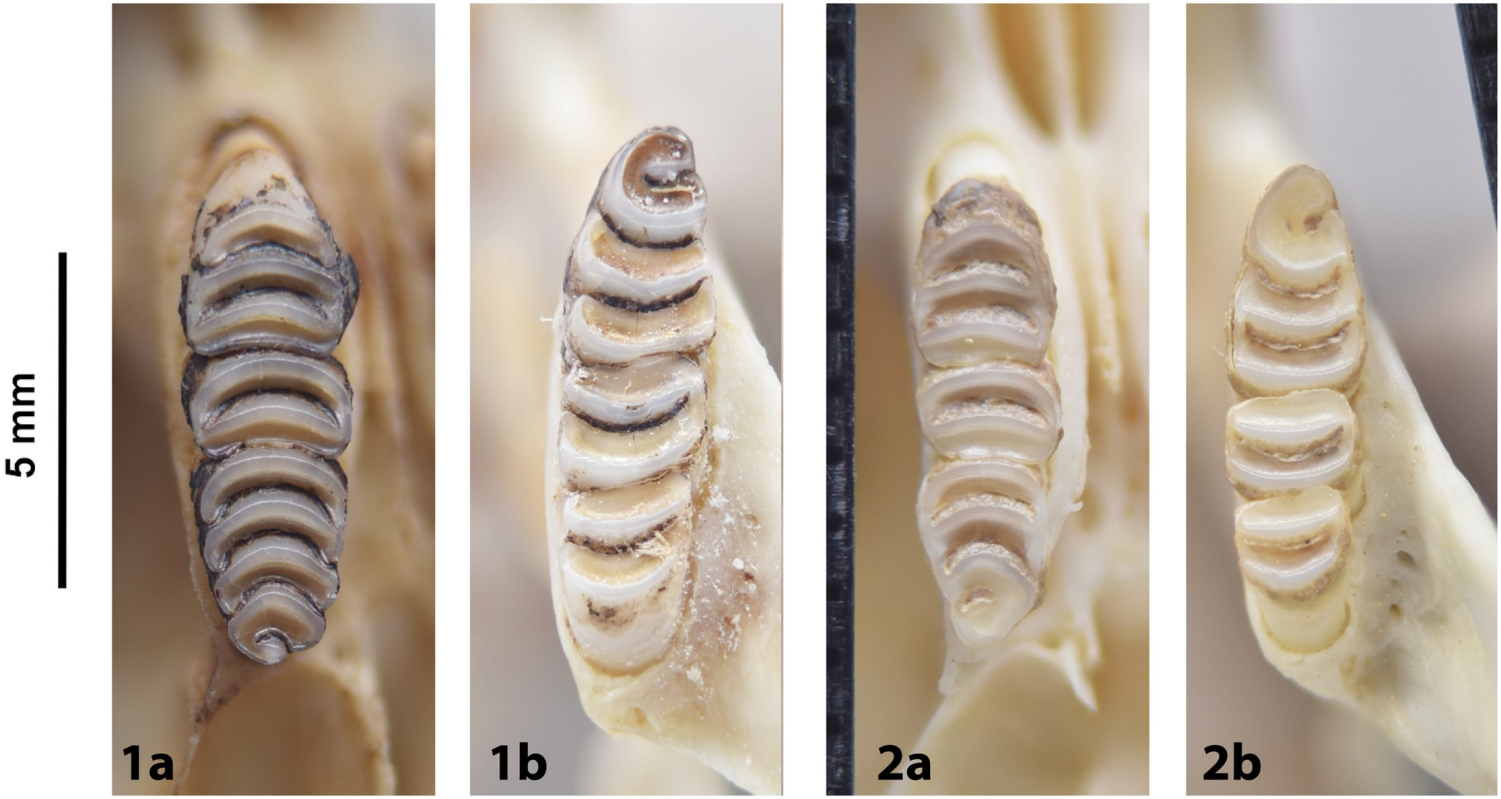
Cheekteeth of *Parotomys*. **1)** Upper (a) and lower (b) right toothrow of *P*. *brantsii* (DNMNH-22612); **2)** Upper (a) and lower (b) right toothrow of *P*. *littledalei* (DNMNH-22446).

**Fig 56:**
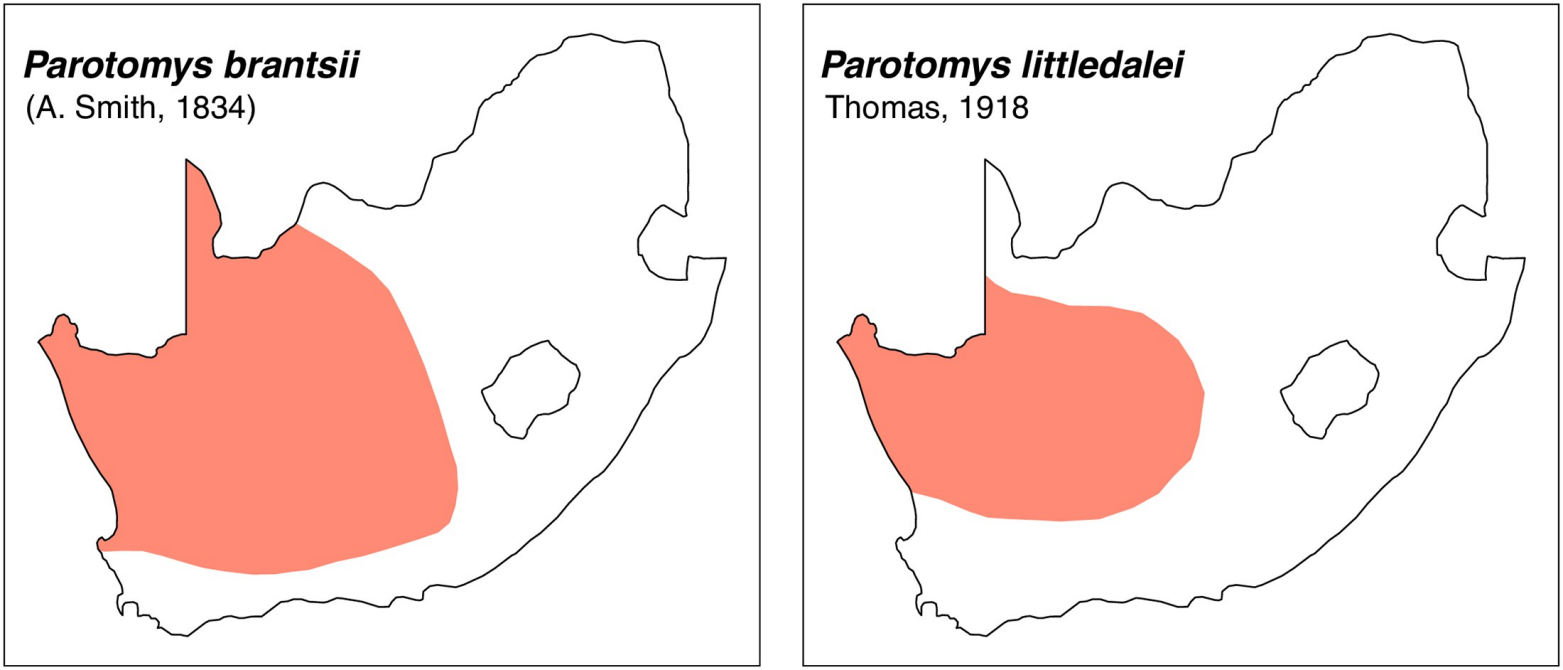
Distribution maps.

Figs 54–56; Table 19

**Table 19:** Dental measurements (in mm) for *Parotomys* from South Africa, sexes and species combined.

	Mean	Min	Max	n
LLTR	7.2	6.4	8.2	23
WM_1_	2.1	1.9	2.4	23
LUTR	7.7	6.6	8.5	23
WM^1^	2.2	2.0	2.4	23

Dental formula is 1-0-0-3:1-0-0-3. Alveolar formula is 4/5-3/4-3/4:4/5-3/4-3/4 (Figs 16 and 17).

#### Upper jaw

Incisors are opisthodont, ungrooved in *P*. *littledalei* and grooved (a single groove) in *P*. *brantsii*. The anterior palatal foramina do not reach the M^1^. Cusps of the cheekteeth are fused into a number of transverse laminae: M^1^ has three laminae; M^2^ has two; M^3^ is the longest cheekteeth, having three (*P*. *brantsii*) or two *(P*. *littledalei*) differentiated laminae and two distal laminae fused by enamel ridge (while they remain separate in *Otomys*). Auditory bullae are distinctively inflated (hardly or not inflated in *Otomys*).

#### Lower jaw

Lower incisors are ungrooved. The M_1_, which is the longest tooth of the toothrow, has four laminae, the first two most anterior of which are joined by an enamel ridge. The M_2_ and the M_3_ have two laminae each.

#### Systematic notes and South African fossil record

Currently, there are two species recognized in South Africa:

- *Parotomys brantsii* (A. Smith, 1834)
- *Parotomys littledalei* (Thomas, 1918)

Fossils of *Parotomys* have been recorded in numerous South African deposits since the Middle Pleistocene [50, 58].

Genus ***Rattus*** Waldheim, 1803

**Fig 57:**
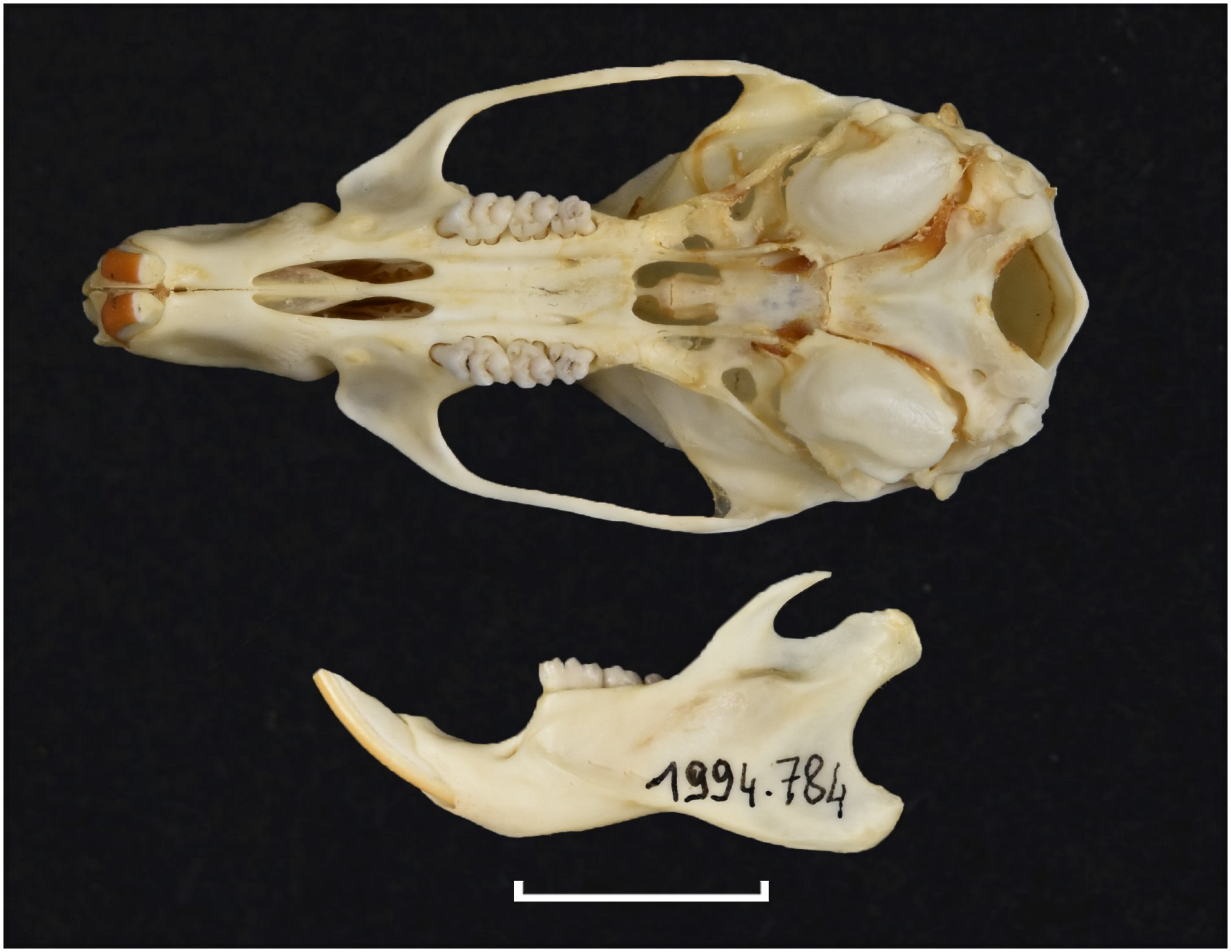
Cranium of *Rattus rattus* (MNHZM-MO-1994-784), with scale bar of 1 cm.

**Fig 58:**
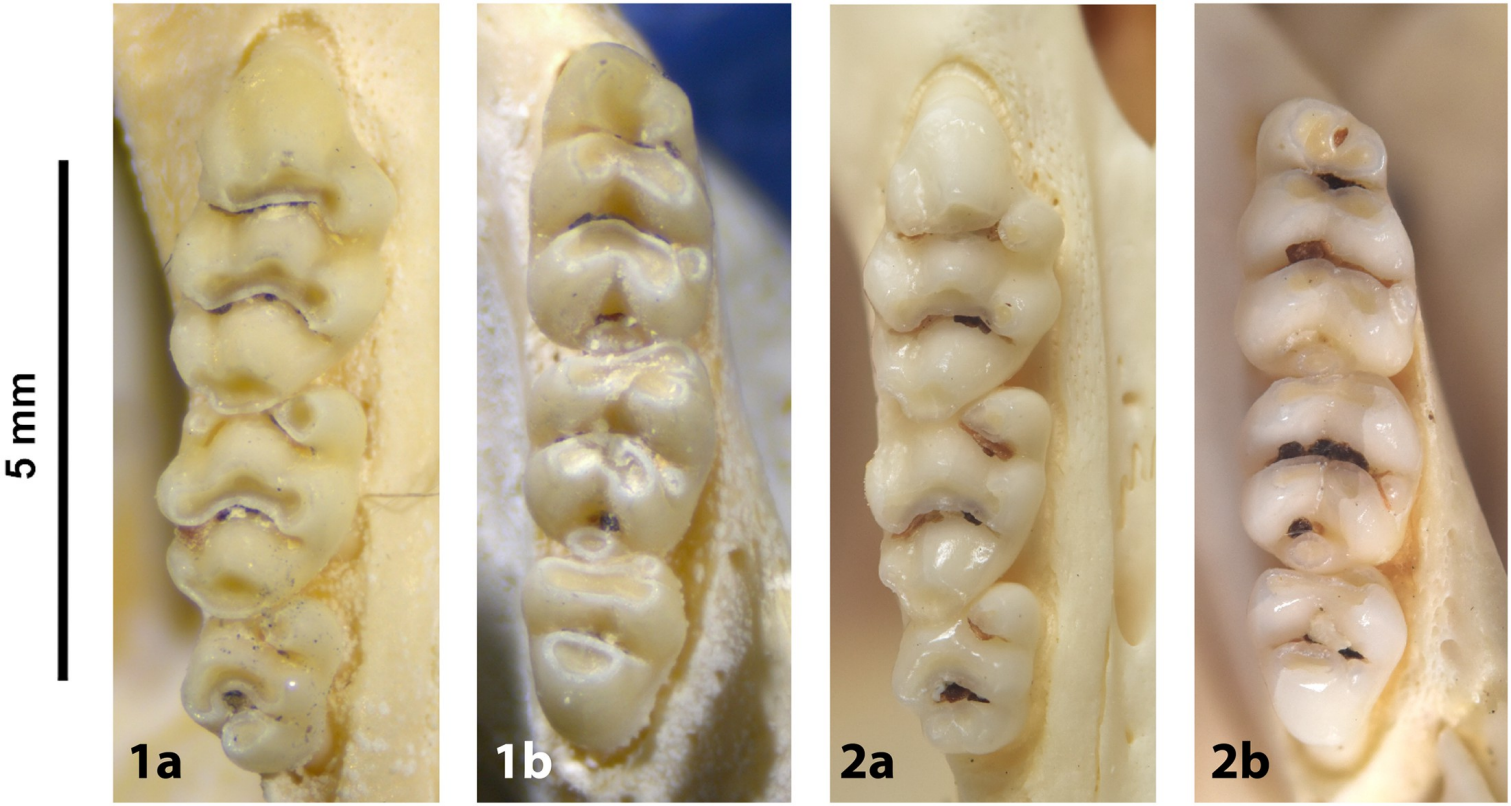
Cheekteeth of *Rattus*. **1)** Upper (a) and lower (b) right toothrow of *R*. *norvegicus* (MNHN-ZM-MO-1888-382); **2)** Upper (a) and lower (b) right toothrow of *R*. *rattus* (MNHN-ZM-MO-1981-352).

**Fig 59:**
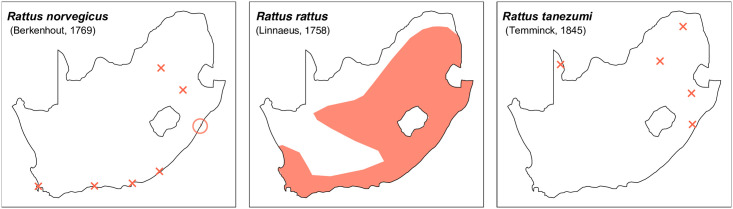
Distribution maps.

Figs 57–59; Table 20

**Table 20:** Dental measurements (in mm) for *Rattus* from South Africa, sexes and species combined.

	Mean	Min	Max	n
LLTR	6.5	5.5	7.5	68
WM_1_	1.8	1.5	2.0	67
LUTR	6.6	5.7	7.6	70
WM^1^	2.0	1.7	2.3	69

Dental formula is 1-0-0-3:1-0-0-3. Alveolar formula is 5-4-3/4:4-3/4-3 (Figs 16 and 17).

#### Upper jaw

Upper incisors are smooth and orthodont. The anterior palatal foramina are rather short, barely reaching short of the root of M^1^. The palate is broad, extending well beyond the M^3^. The dentition is proportionally small with relatively narrow teeth. The central cusps of the upper molars are pronounced. In the M^1^, the t1 is displaced backwards to the t2 and connected to it by a ridge, and cusp t7 is absent. In both M^1^ and M^2^, t9 is reduced and very close to the t8 (forming a single lamina when worn). In M^2^, the t3 is small or absent. The M^3^ displays a prominent t1 but lacks t3, corresponding to Group 3 in Fig 12; it is not markedly smaller than the M^2^.

#### Lower jaw

The alveolar region of the mandible is well-developed in relation to the whole mandible. Both the M_1_ and the M_2_ have a posterior cingulum (often oval-shaped); they lack anterior cusps but up to three small posterolabial cusps may be present.

#### Systematic notes and South African fossil record

This genus was unintentionally introduced to South Africa. Three species are currently recognized:

- *Rattus norvegicus* (Berkenhout, 1769)
- *Rattus rattus* (Linnaeus, 1758)
- *Rattus tanezumi* Temminck, 1844

*R*. *tanezumi* was first discovered in South Africa by Bastos et al. [60] from two sites in Limpopo Province, and has been identified since then from further localities. There is debate about the species status of this taxon, and some authors classify it rather as belonging to a lineage of *R*. *rattus* [61]. The three species *R*. *norvegicus*, *R*. *rattus* and *R*. *tanezumi* are commensal to humans. According to archaeological data, it seems that *R*. *rattus* followed human migrations into southern Africa during Iron Age [62] and that *R*. *norvegicus* may have arrived from European ships during the 19^th^ century [63].

Genus ***Rhabdomys*** Thomas, 1916 (Four-striped Grass Mice)

**Fig 60:**
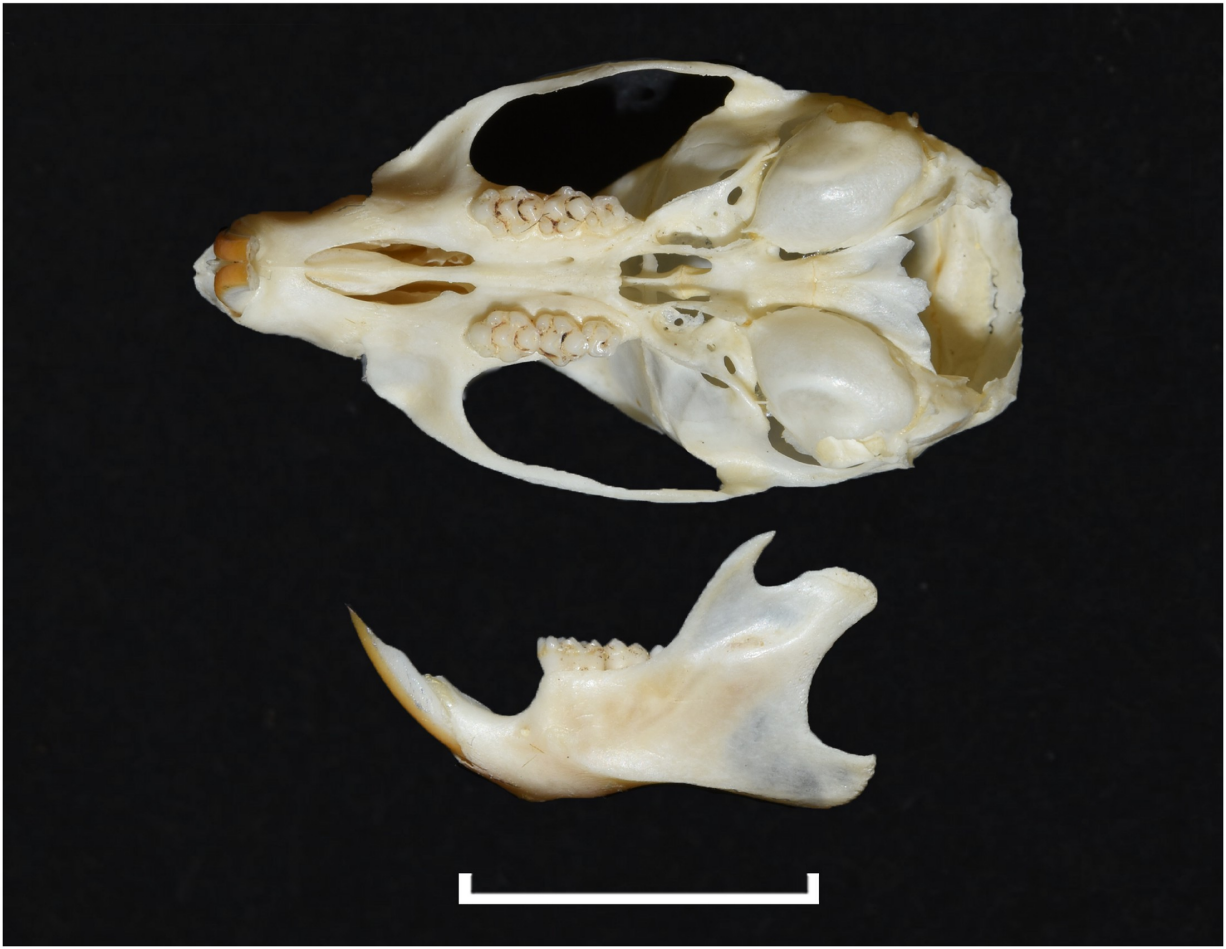
Cranium of *Rhabdomys dilectus* (IVB-M-T8x353), with scale bar of 1 cm.

**Fig 61:**
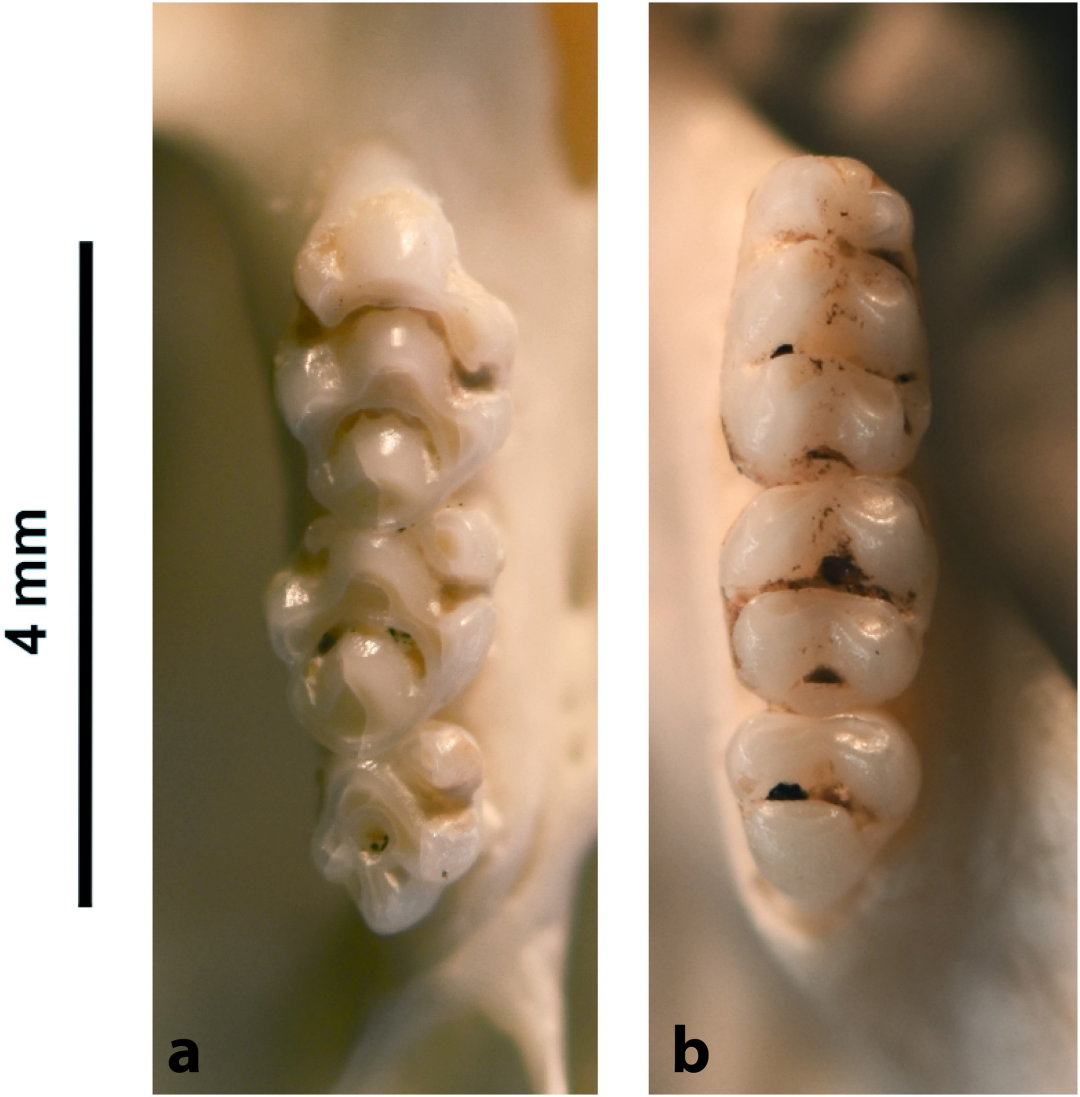
Upper (a) and lower (b) right toothrow of *R*. *dilectus* (upper IVB-T8x336; lower IVB-T8x353).

**Fig 62:**
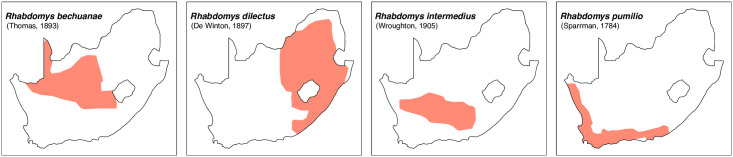
Distribution maps.

Figs 60–62; Table 21

**Table 21:** Dental measurements (in mm) for *Rhabdomys* from South Africa, sexes and species combined.

	Mean	Min	Max	n
LLTR	4.5	4.3	4.8	20
WM_1_	1.2	1.1	1.3	20
LUTR	4.6	4.3	4.8	20
WM^1^	1.5	1.3	1.7	20

Dental formula is 1-0-0-3:1-0-0-3. Alveolar formula is 5-5-3:4-4/5-3 (Figs 16 and 17).

#### Upper jaw

Upper incisors are ungrooved and orthodont to opisthodont. The anterior palatal foramina reach just to the anterior root alveolus of the M^1^. Upper cheekteeth display large central cusps. In the M^1^, the t1 is located behind t2 and t3, and t4 behind t5; the second and third rows of cusps appear to be linked on the lingual and labial sides. In both the M^1^ and M^2^, the t9 is small and often reduced to a small ridge that connects with the t6. The M^3^ lacks the t3 and has one distal cusp. Length of upper cheekteeth is similar to *Mastomys* and *Myomyscus*, but the number of roots and alveoli is different (M^1^ is 5-rooted in *Rhabdomys*, 3-rooted in *Mastomys* and *Myomyscus*).

#### Lower jaw

The M_1_ has two anterior cusps and an additional small tma is often present. Both M_1_ and M_2_ have labial cusplets and reduced to absent posterior cingulum. The lower dentition is of similar size than *Mastomys* but both genera can be distinguished almost unambiguously on the basis of the number of roots and corresponding alveoli: the M_1_ of *Rhabdomys* is 4-rooted, with the central pair of roots usually visible on the sides of the tooth *in situ*, while the M_1_ of *Mastomys* is 2-rooted.

#### Systematic notes and South African fossil record

Until recently, the genus was considered as monotypic [21] with *R*. *pumilio* being the only known species, but new karyotypic and genotypic analyses revealed a more complex diversity [64, 65]. Today, four species are recognized in South Africa:

- *Rhabdomys bechuanae* (Thomas, 1893)
- *Rhabdomys dilectus* (De Winton, 1897)
- *Rhabdomys intermedius* (Wroughton, 1905)
- *Rhabdomys pumilio* (Sparman, 1784)

The oldest known *Rhabdomys* have been discovered around 5 MYA in Langebaanweg [66] and 3.3 MYA in Makapansgat [48]. Fossils are known from a variety of Pleistocene localities. This material was identified as *R*. *pumilio* at a time when the specific diversity of *Rhabdomys* had not been recognized and is likely to include material attributable to other species.

Genus ***Thallomys*** Thomas, 1920 (Acacia Rats or Tree Rats)

**Fig 63:**
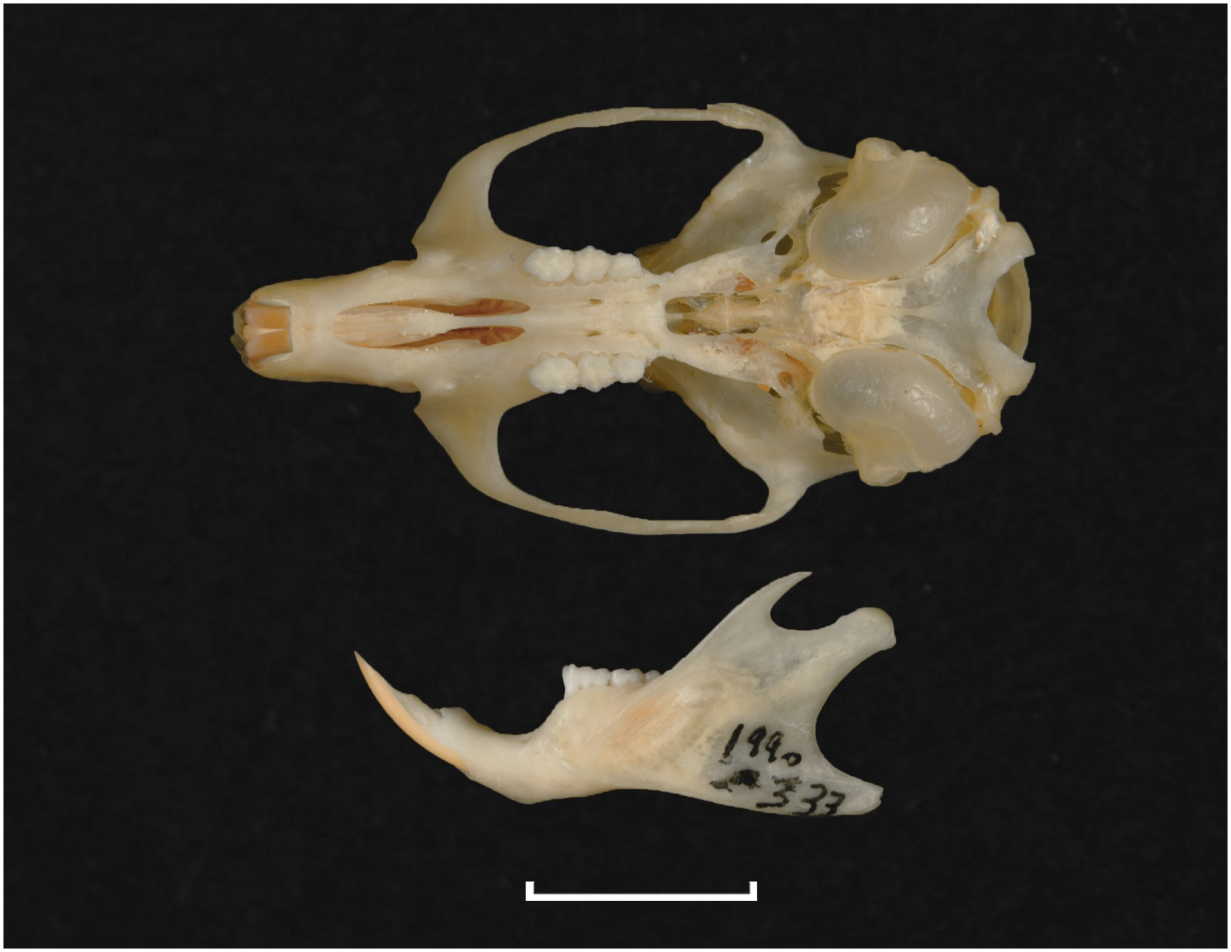
Cranium of *Thallomys paedulcus* (MNHN-ZM-MO-1990-333), with scale bar of 1 cm.

**Fig 64:**
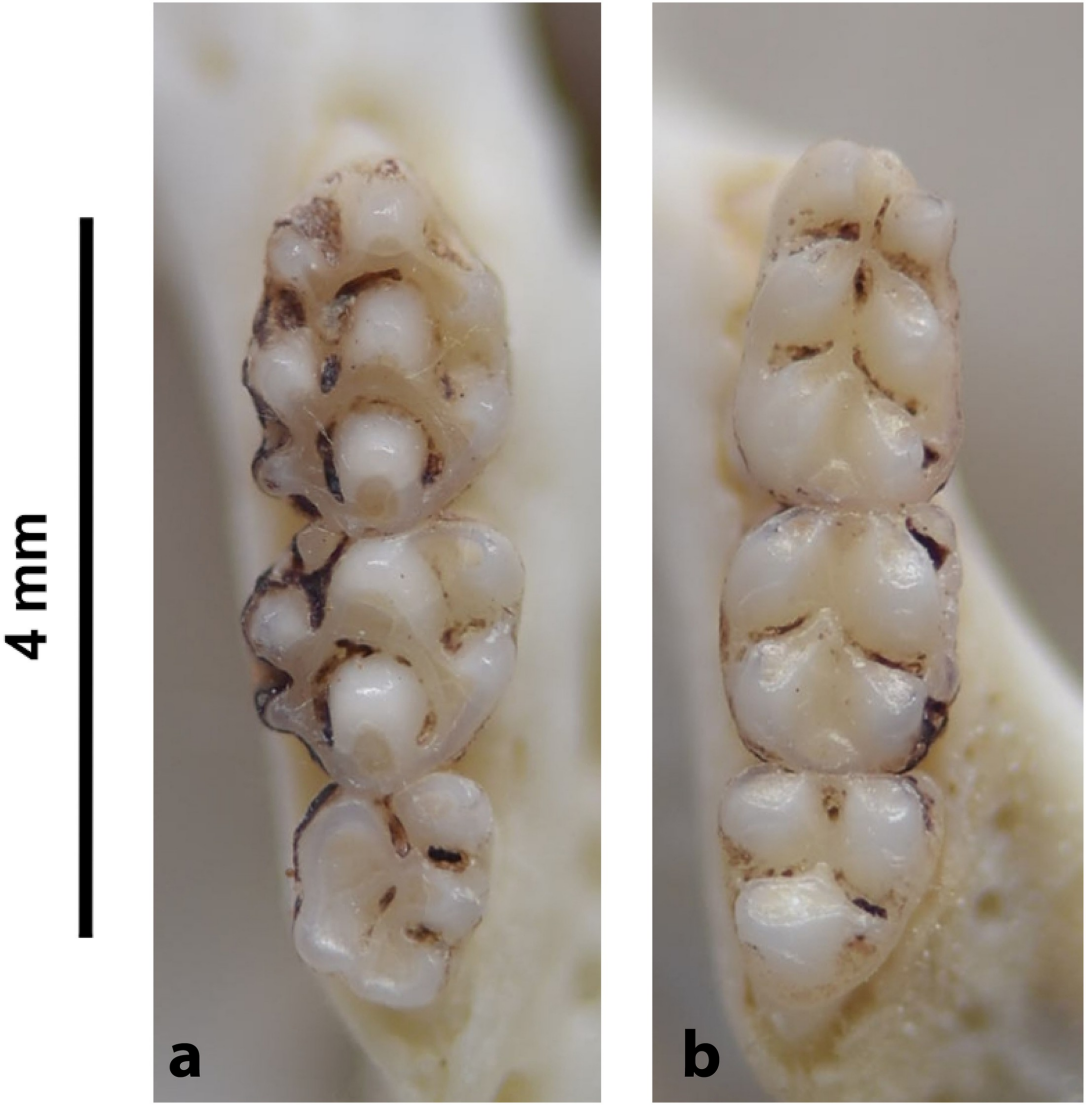
Upper (a) and lower (b) right toothrow of *T*. *paedulcus* (DNMNH-30229).

**Fig 65:**
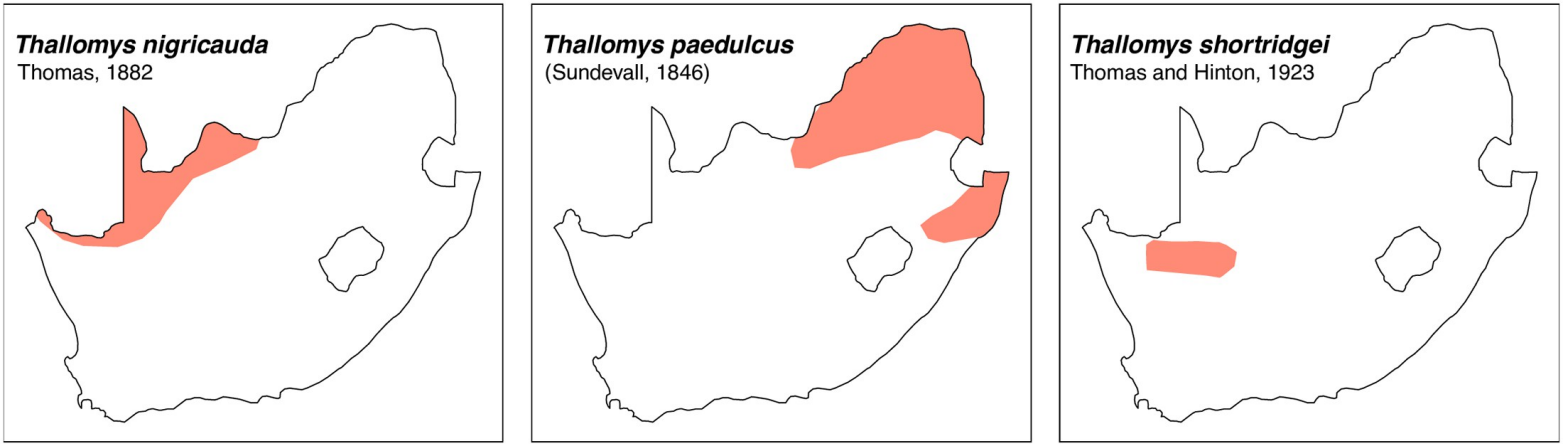
Distribution maps.

Figs 63–65; Table 22

**Table 22:** Dental measurements (in mm) for *Thallomys* from South Africa, sexes and species combined.

	Mean	Min	Max	n
LLTR	5.4	4.6	6.0	21
WM_1_	1.4	1.2	1.6	21
LUTR	5.5	4.7	6.3	21
WM^1^	1.6	1.4	1.8	21

Dental formula is 1-0-0-3:1-0-0-3. Alveolar formula is 5-4/5-3/4:4/5-4-3 (Figs 16 and 17).

#### Upper jaw

Incisors are orthodont and ungrooved. The palatal foramina are large and penetrate between the second rows of cusps on M^1^. Molars have an embossed, angular appearance, and show microdonty. In the M^1^, the t7 is absent or much reduced, and there are small stephanodont crests on t1 and t3 and joining t6 with t9. The central cusps (t2, t5 and t8) are prominent. In the M^2^ the cusps t2 and t7 are absent. The molar M^3^ is the smallest of the toothrow, but not greatly reduced; it corresponds to Group 6 in Fig 12.

#### Lower jaw

Cusps of lower molars are prominent and sharply defined. The M_1_ and M_2_ have equal-sized posterior cingula, and a longitudinal crest that connects the lobes on the labial side. The M_1_ often displays small labial extra cusplets or ridges, and sometimes a small anteromedian cusplet. Both the M_2_ and M_3_ have antero-external cusplets. The toothrow is relatively small compared to the size of the mandible (microdonty).

#### Systematic notes and South African fossil record

According to Monadjem et al. [22], this genus requires urgent revision. Currently, three species are listed in South Africa:

- *Thallomys nigricauda* Thomas, 1882
- *Thallomys paedulcus* (Sundevall, 1846)
- *Thallomys shortridgei* Thomas and Hinton, 1923

An additional fossil species has been described in South Africa:

- *† Thallomys debruyni* Broom, 1948

The oldest known fossil of *Thallomys* was found in Langebaanweg around 5MYA [66]. Fossils of this genus are known from various Pleistocene localities in South Africa.

Genus ***Zelotomys*** Osgood, 1910 (Broad-headed Mice)

**Fig 66:**
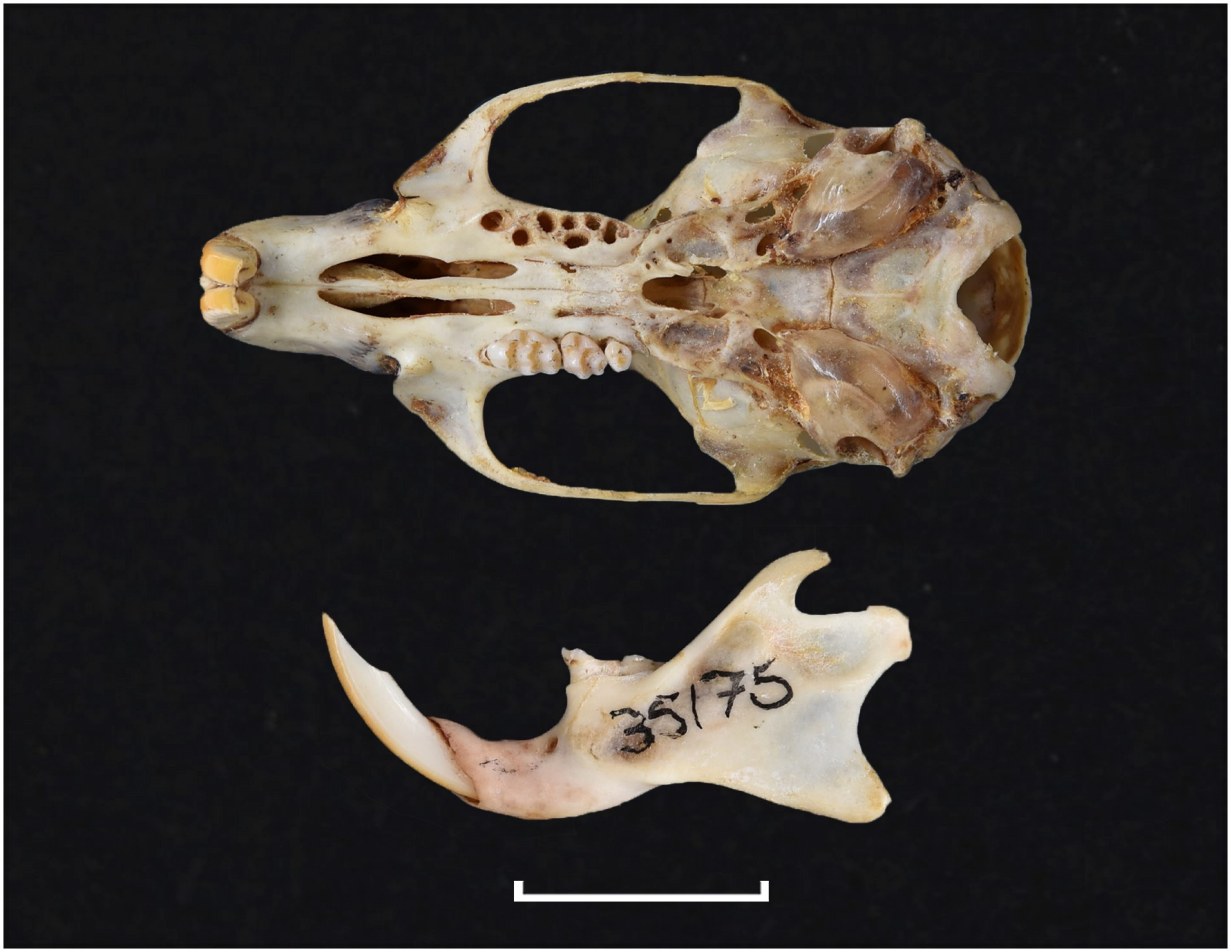
Cranium *of Zelotomys woosnami* (DNMNH-30229), with scale bar of 1 cm.

**Fig 67:**
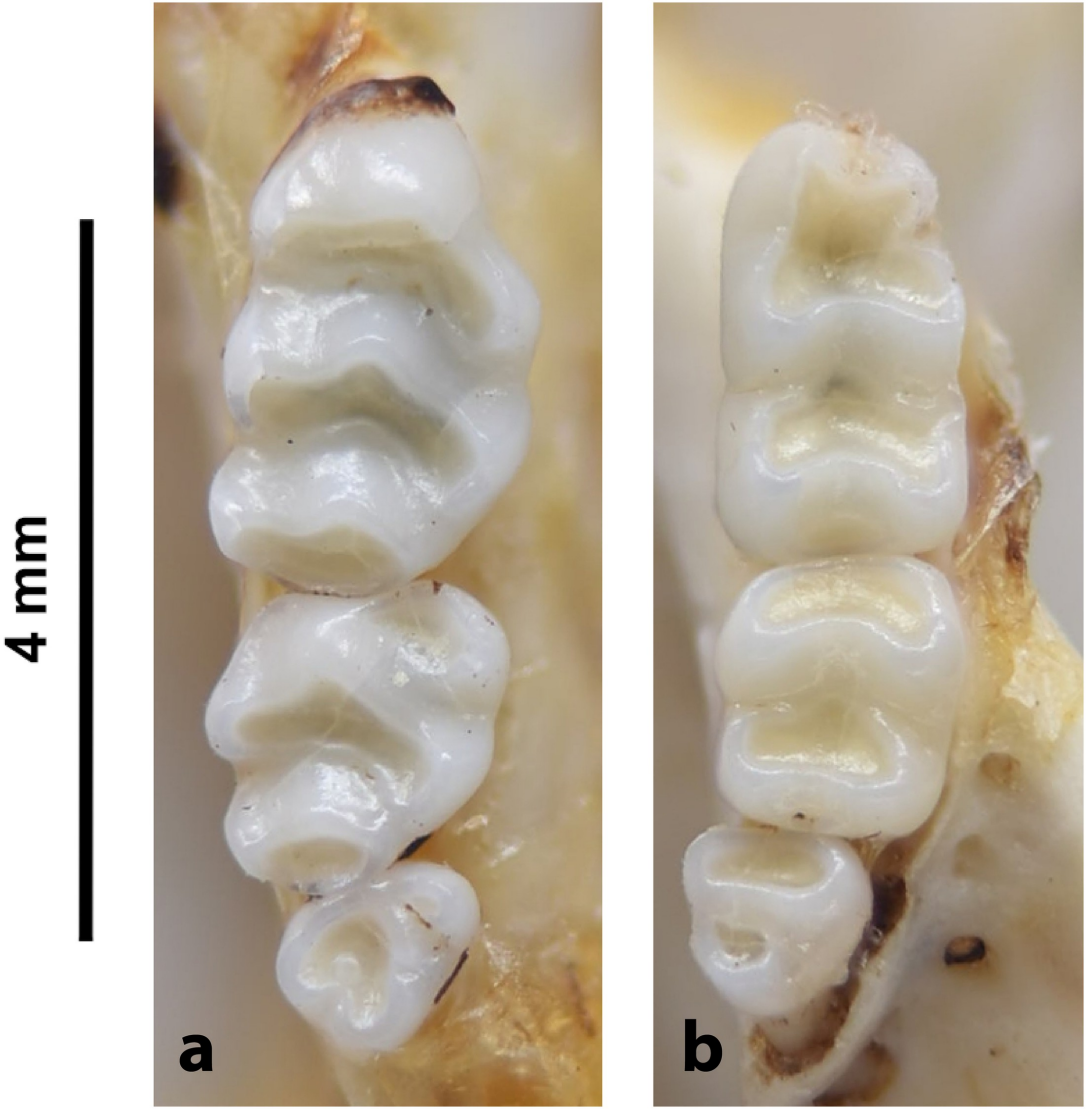
Upper (a) and lower (b) right toothrow of *Z*. *woosnami* (upper DNMNH-6413; lower DNMNH-35175).

**Fig 68:**
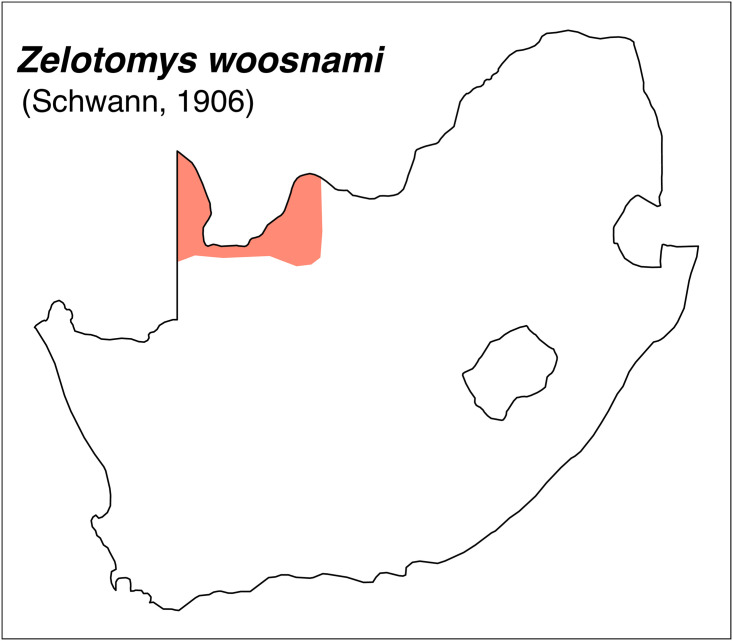
Distribution map.

Figs 66–68; Table 23

**Table 23:** Dental measurements (in mm) for *Zelotomys woosnami*, sexes combined.

	Mean	Min	Max	n
LLTR	5.1	5.0	5.3	10
WM_1_	1.4	1.3	1.5	10
LUTR	5.3	4.9	5.7	10
WM^1^	1.8	1.7	1.9	10

Dental formula is 1-0-0-3:1-0-0-3. Alveolar formula is 3-3-2/3:2-2-2 (Figs 16 and 17).

#### Upper jaw

Upper incisors are ungrooved and opisthodont. The palatal foramina reach the second root of the three-rooted M_1_. The labial row of cusps is well developed and tend to project outwards in a characteristic way. In the M^1^, t1 and t2 tend to fuse with wear. As with other members of the Praomyiny tribe (*Mastomys* and *Myomyscus*), the t9 is well separated from the t6, and located far in the labial side. The M^2^ is as broad as or broader than long, with a low t9 and a t3 very small, often reduced to a tiny process. The M^3^ is markedly reduced, with traces of two laminae (group 5 in Fig 12) and a big t1.

#### Lower jaw

Lower incisors are plain and ungrooved; they are relatively strong, with the alveolar region of the mandible well-developed, and generally extend far beyond the alveolus. The prelobe of the M_1_ has two nearly longitudinal, poorly differentiated cusps that are connected to those of the second lobe, together forming a characteristic compact “trefoil” like pattern, while the third lobe is located further below. There is a small cingulum posterior on M_1_ and M_2_. The M_3_ is reduced.

#### Systematic notes and South African fossil record

Currently, a single species is recognised in South Africa:

- *Zelotomys woosnami* (Schwann, 1906)

The oldest known fossil of *Zelotomys* was found in Langebaanweg around 5MYA [66]. Fossils of this genus are known from various Pleistocene localities in South Africa.

Family **NESOMYIDAE** Major, 1897

Subfamily **CRICETOMYINAE** Roberts, 1951

Genus ***Cricetomys*** Waterhouse, 1840 (Giant Pouched Rats)

**Fig 69:**
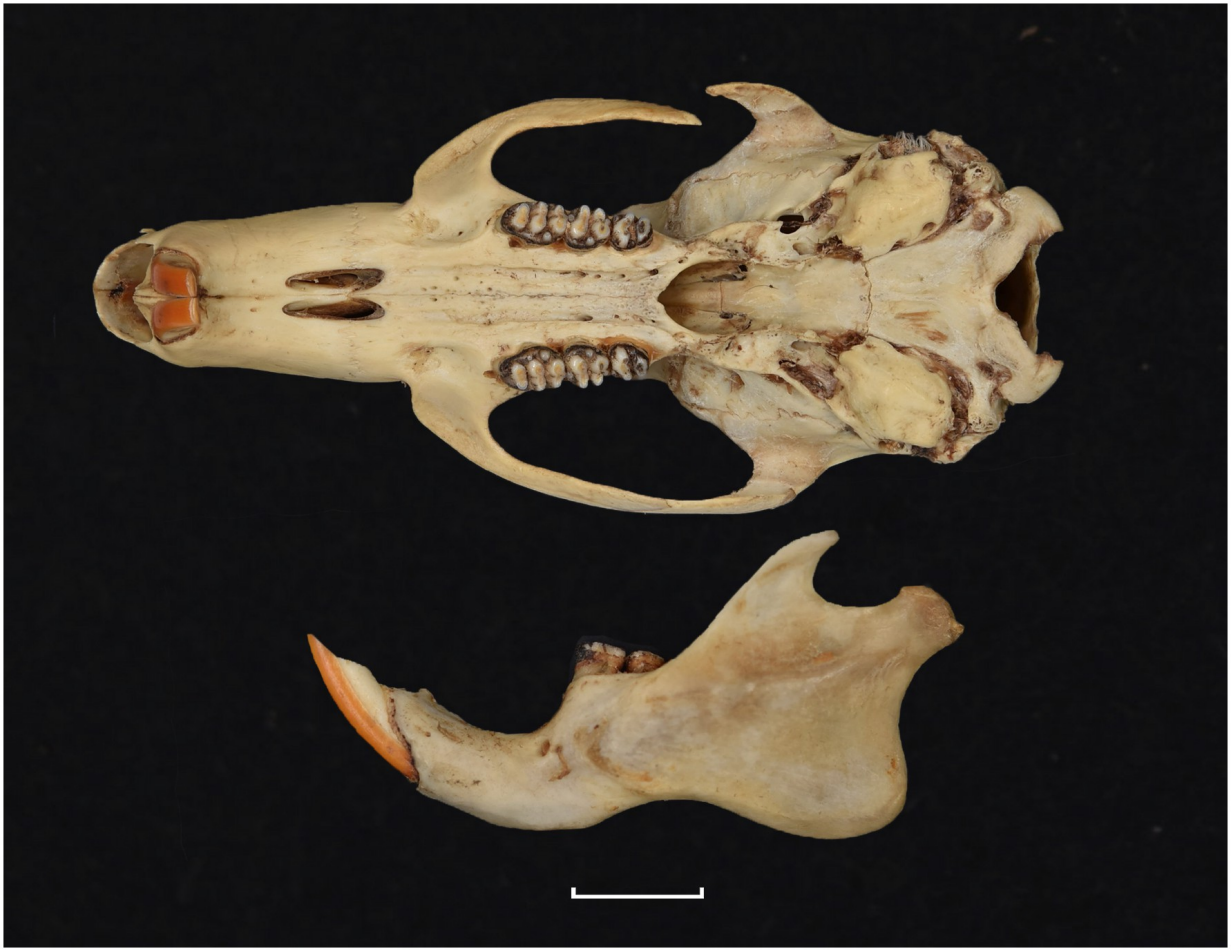
Cranium of *Cricetomys ansorgei* (DNMNH-13954), with scale bar of 1 cm.

**Fig 70:**
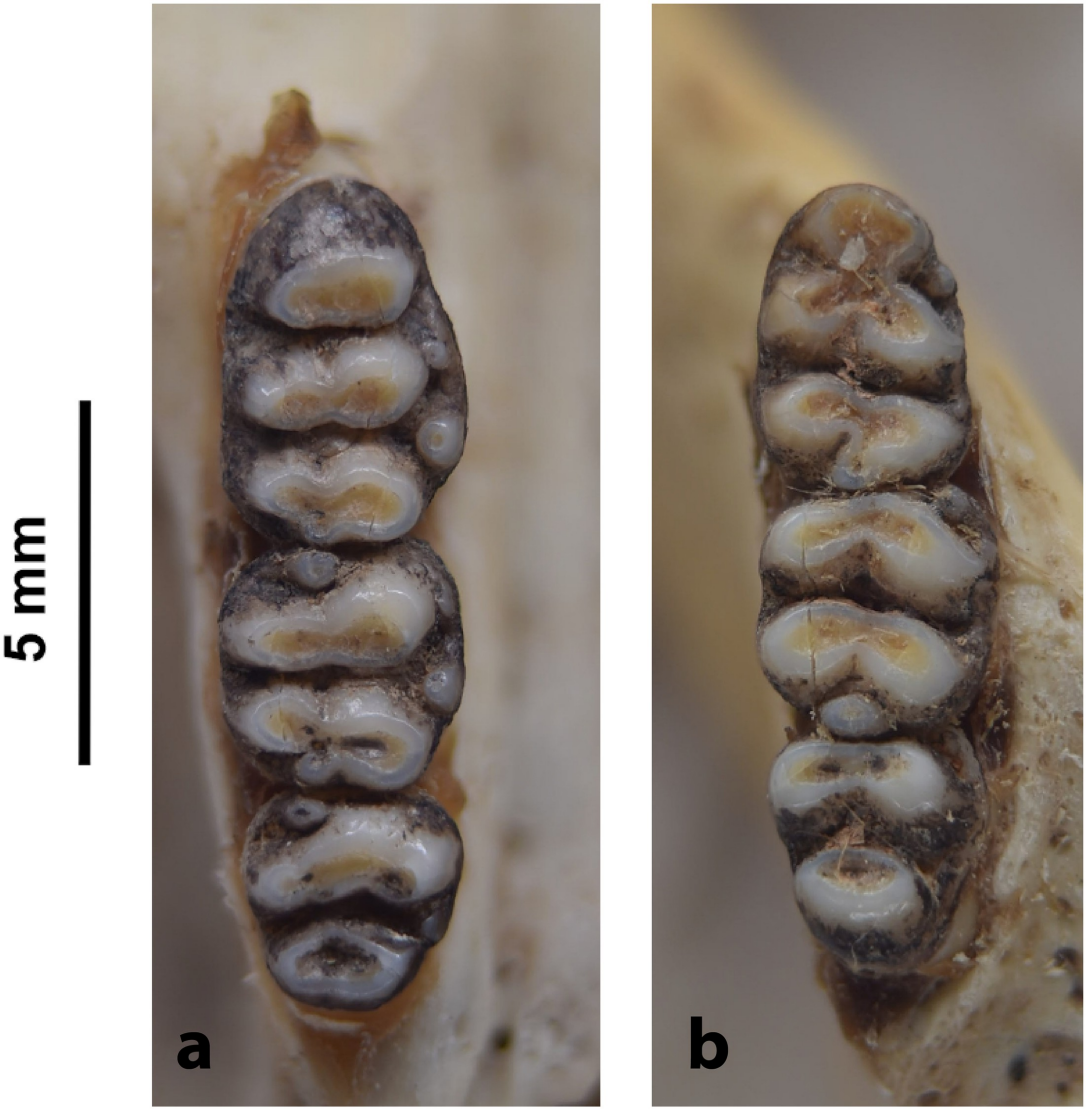
Upper (a) and lower (b) right toothrow of *C*. *ansorgei* (DNMNH-30736).

**Fig 71:**
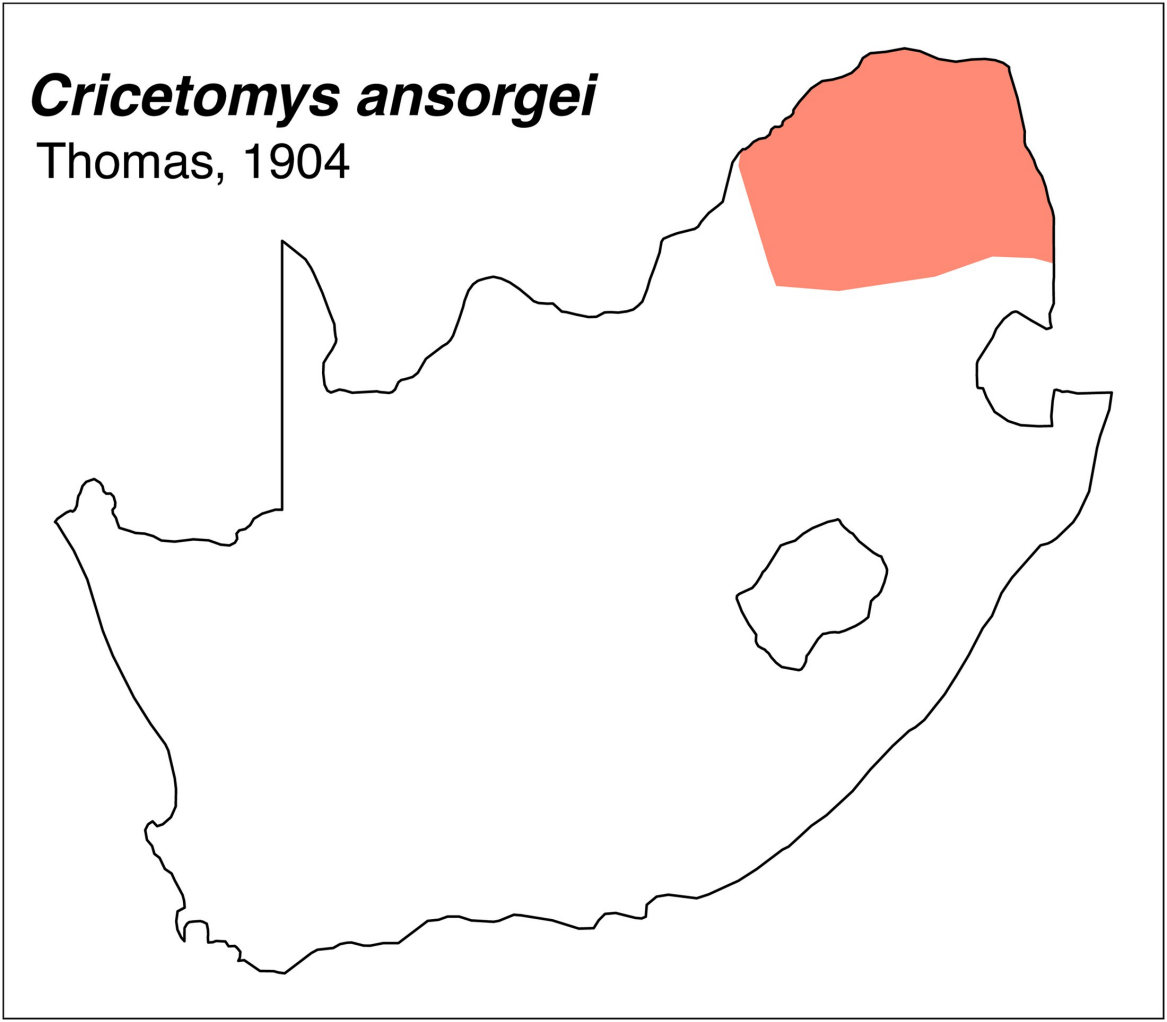
Distribution map.

Figs 69–71; Table 24

**Table 24:** Dental measurements (in mm) for *Cricetomys ansorgei*, sexes combined.

	Mean	Min	Max	n
LLTR	10.7	10.3	11.1	3
WM_1_	3.0	2.9	3.1	3
LUTR	11.3	10.9	11.6	3
WM^1^	3.3	3.2	3.6	3

Dental formula is 1-0-0-3:1-0-0-3. Alveolar formula is 3-3-3:2-2/3-2/3 (Figs 16 and 17).

#### Upper jaw

Upper incisors are opisthodont and ungrooved but display characteristic striations on their anterior enamel surface, forming a slightly raised band (this feature is also present in *Saccostomus* and *Mystromys*). The anterior palatal foramina are short, located before the basis of the zygomatic arches. Molars are large, with high, well-separated cusps. The M^1^ has three lobes, The M^2^ and M^3^ two lobes each. In the first lobe of M^1^, the t2 and t3 are large and connected, being well separated from the t1 which is smaller and isolated below; similarly in the second lobe, the t5 and t6 are large and separated from the t4; the third lobe has no t7, but a large posterior cingulum. The M^2^ has two lobes, with t4 separated from t5 and t6, and a conspicuous posterior cingulum. The M^3^ is almost as large as the M^2^ and corresponds to Group 1 in Fig 12.

#### Lower jaw

Lower incisors are ungrooved but display the same ridged band as the upper incisors. The molar M_1_ has three lobes: the first lobe has one elongated cusp, the second and third lobes have two cusps each that are aligned transversally. It has a well-developed posterior cingulum and a variable number (generally 2) of supplementary cusps on the labial side. As in *Saccostomus* (another member of the subfamily Cricetomyinae), the coronoid process is well-developed while the angular process is not projected backwards.

#### Systematic notes and South African fossil record

South African specimens of *Cricetomys* were previously attributed to *C*. *gambianus* Waterhouse, 1840, but recent morphological and molecular analyses revealed the existence of multiple species [67]. Today, the specimens recorded in Southern Africa are attributed to a single species:

- *Cricetomys ansorgei* Thomas, 1904

Fossils of *Cricetomys* have been identified from very few Quaternary deposits in South Africa. Its oldest record is from the Late Pleistocene from Sibudu [68] and Rose Cottage Cave [69].

Genus ***Saccostomus*** Peters, 1846 (Pouched Mice)

**Fig 72:**
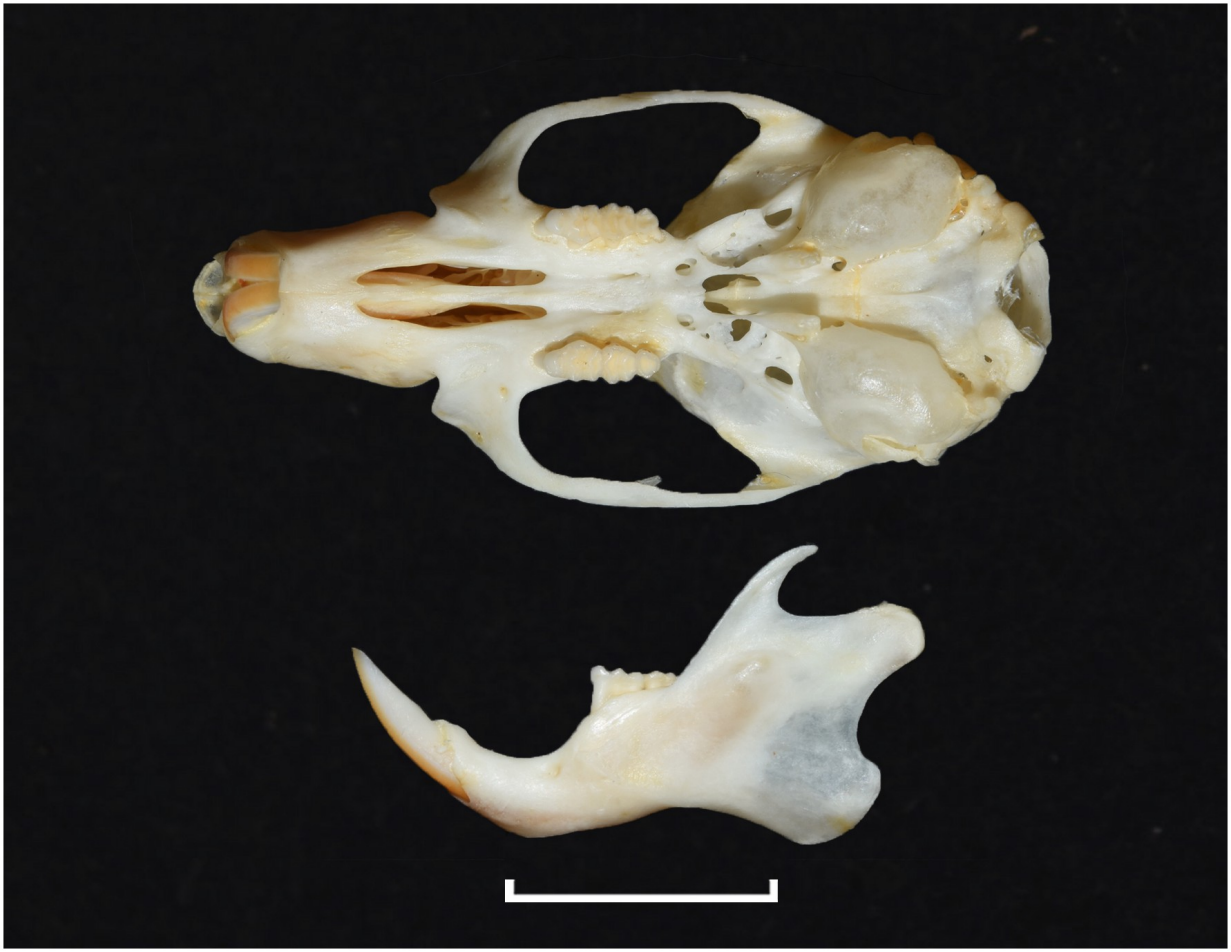
Cranium of *Saccostomus campestris* (DNMNH-4203), with scale bar of 1 cm.

**Fig 73:**
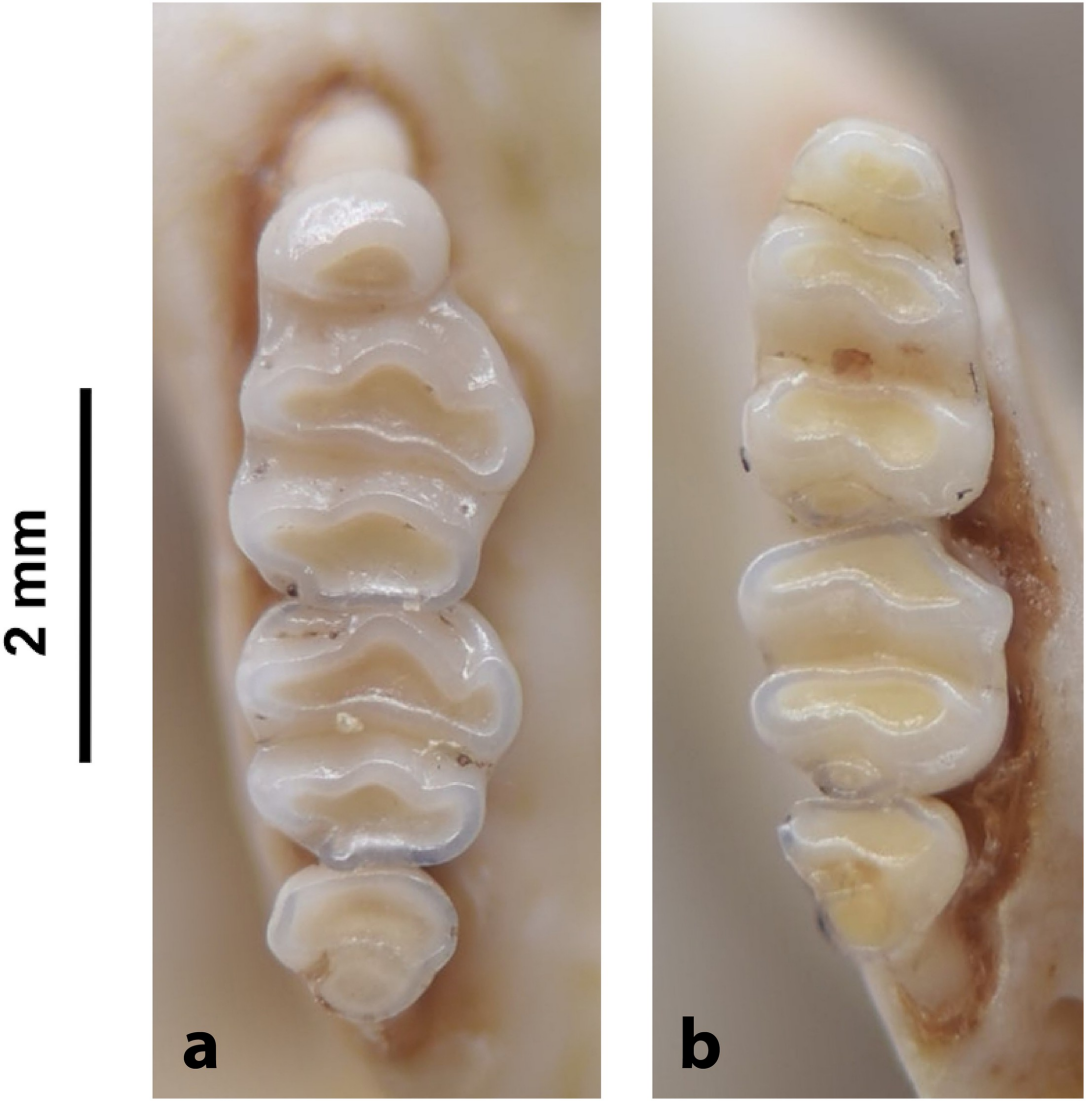
Upper (a) and lower (b) right toothrow of *S*. *campestris* (DNMNH-4203).

**Fig 74:**
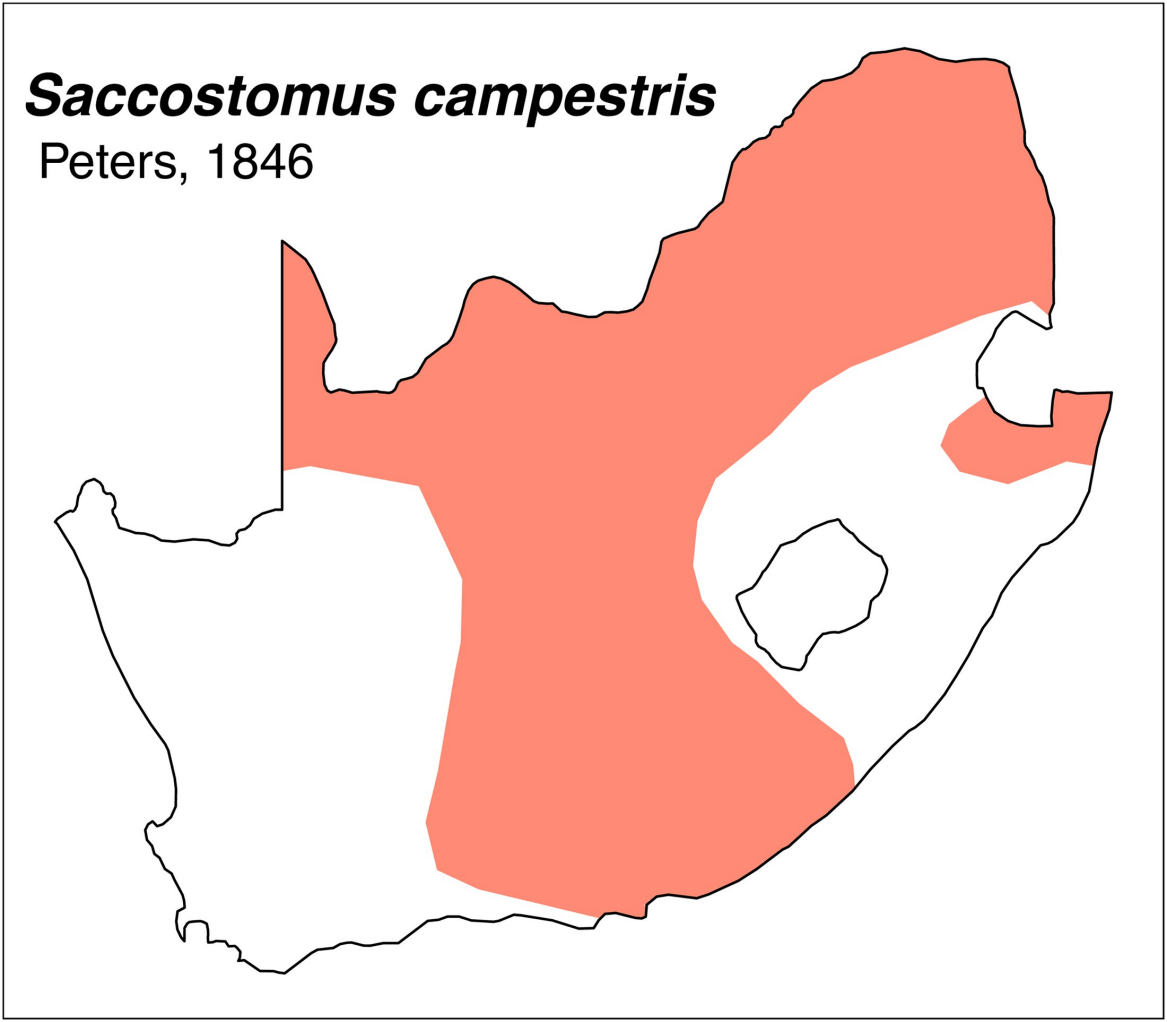
Distribution map.

Figs 72–74; Table 25

**Table 25:** Dental measurements (in mm) for *Saccostomus campestris*, sexes combined.

	Mean	Min	Max	n
LLTR	4.4	4.1	4.9	11
WM_1_	1.3	1.2	1.4	11
LUTR	4.8	4.1	5.9	15
WM^1^	1.5	1.3	1.6	15

Dental formula is 1-0-0-3:1-0-0-3. Alveolar formula is 3-3-3:2-2-2 (Figs 16 and 17).

#### Upper jaw

Upper incisors are opisthodont and ungrooved, but they display characteristic striations on their anterior enamel surface, forming a slightly raised band (this feature is also present in *Cricetomys* and *Mystromys*). The anterior palatal foramina reach the anterior root of the first upper molar. Molars have bulbous cusps that are connected transversally. The M^1^ has three lobes with only the outer two cusps in the first row (it lacks the t1), and three cusps on the second and third rows. Both the M^1^ and the M^2^ have a relatively well-developed posterior cingulum. The M^2^ and M^3^ have two lobes each. The M^3^ is not greatly reduced, as in the Dendromurinae; it corresponds to Group 1 in Fig 12.

#### Lower jaw

Lower incisors are ungrooved but display the same ridged band than in the upper incisors. The M_1_ has three lobes and a small posterior cingulum. The first lobe consists of a single elongated cusp (sometimes two poorly differentiated cusps are visible), which may be connected to the second row of cusps. The second and third lobes have two fused cusps each. The M_2_ has two lobes, with an antero-external cusps of variable size and a posterior cingulum. The M_3_ has two lobes with a tiny antero-external cingulum.

#### Systematic notes and South African fossil record

One species is currently recognized in South Africa:

- *Saccotomus campestris* Peters, 1846

This genus seems absent from South Africa until the late Pleistocene, with the oldest remains discovered in Border Cave around 0.2 MYA [70], in Sterkfontein StP6 around 0.1 MYA [5], in younger levels of Makapansgat Cave of Hearths [1] and in Gladysvale Pink Breccia, S18.E6, and S19.6 of uncertain age [71].

Subfamily **DENDROMURINAE** G.M. Allen, 1939

Genus ***Dendromus*** Smith, 1829 (African Climbing Mice)

**Fig 75:**
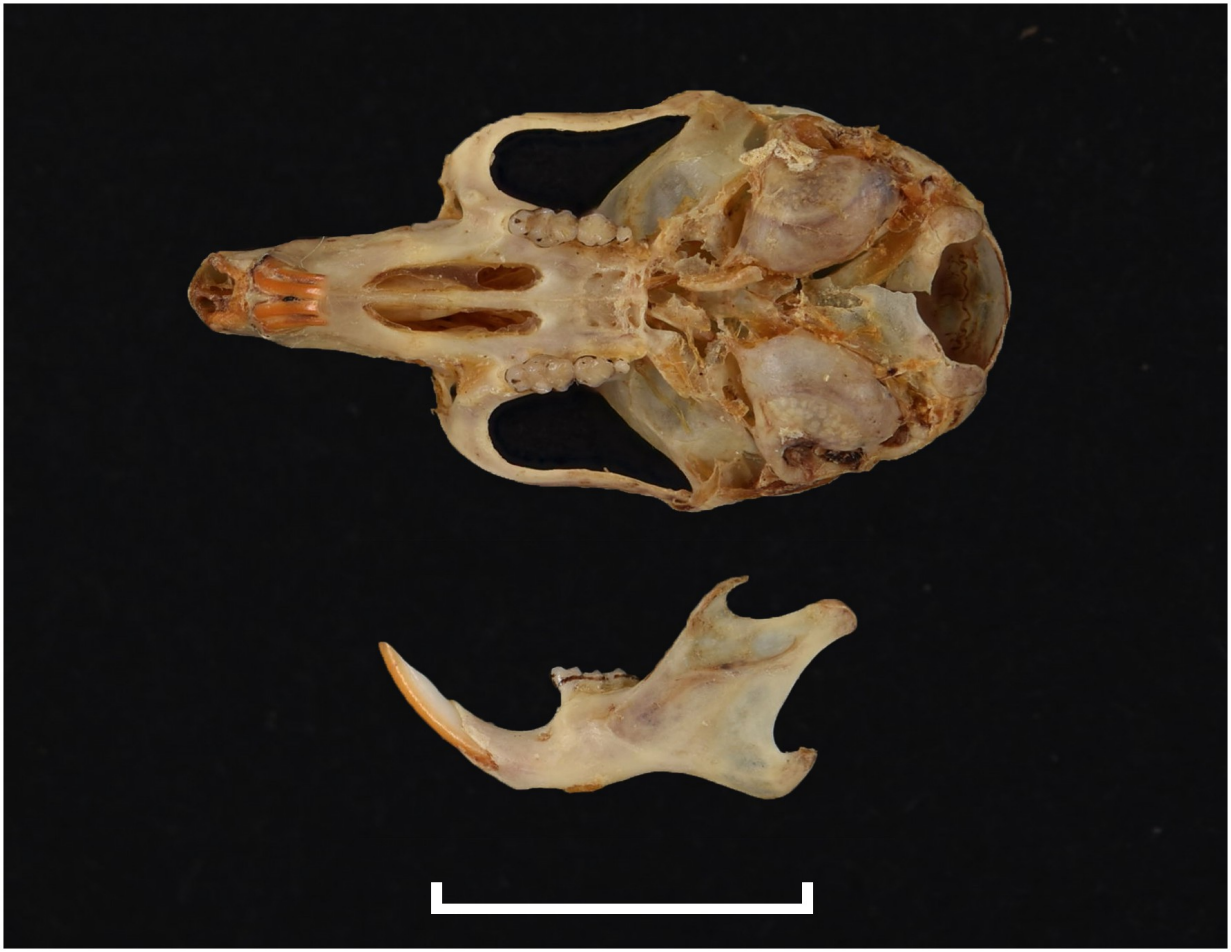
Cranium of *Dendromus (Poemys) nyikae* (DNMNH-34625), with scale bar of 1 cm.

**Fig 76:**
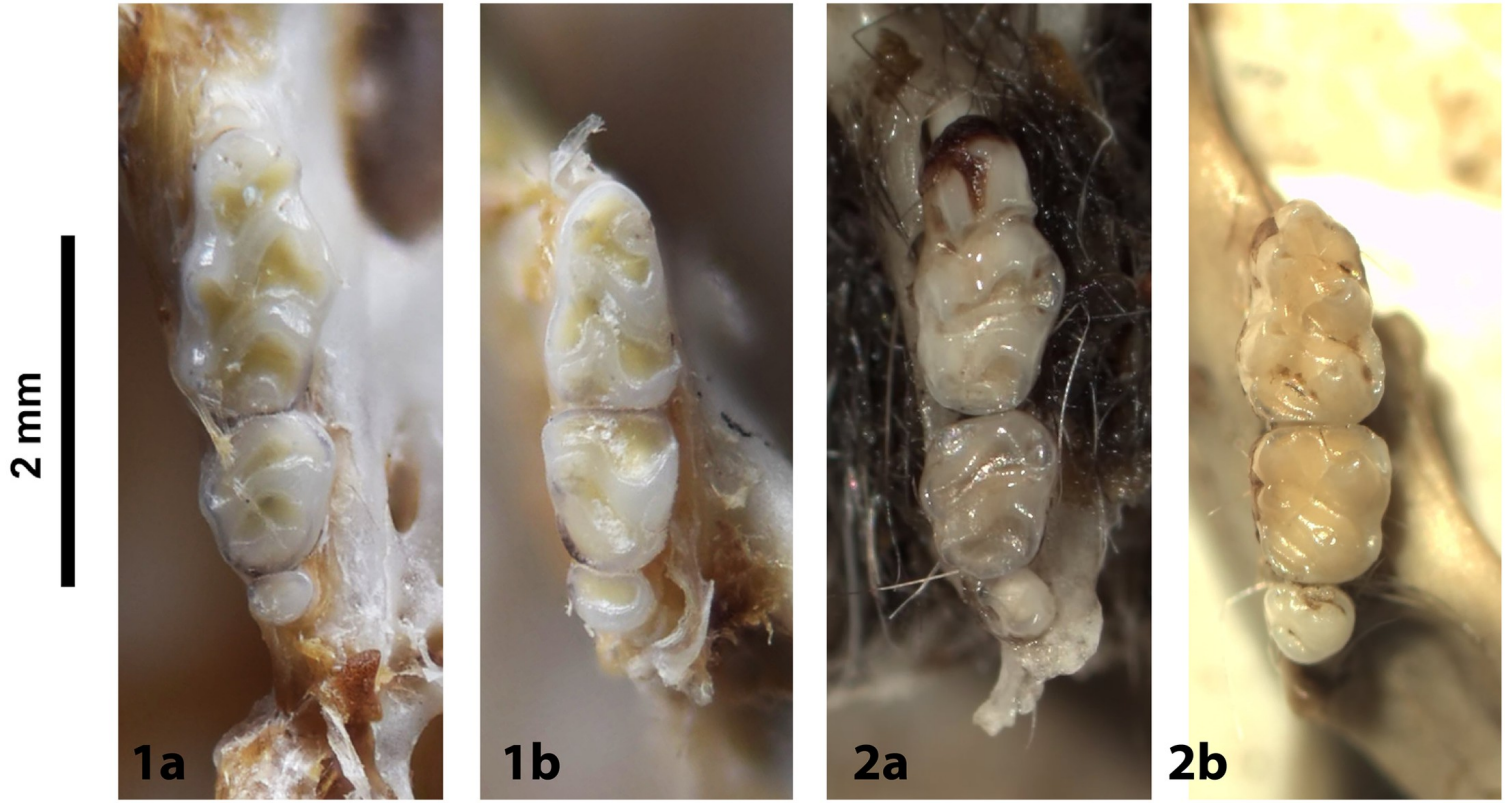
Cheekteeth of *Dendromus*. **1)** Upper (a) and lower (b) right toothrow of *D*. *mesomelas* (DNMNH-2445); **2)** Upper (a) and lower (b) right toothrow of *Dendromus* sp. (ESI modern owl pellet collection).

**Fig 77:**
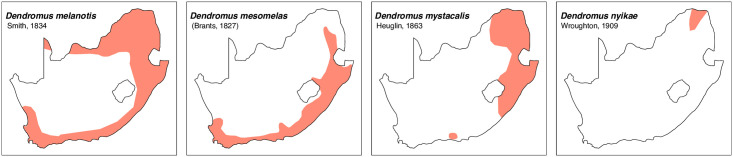
Distribution map.

Figs 75–77; Table 26

**Table 26:** Dental measurements (in mm) for *Dendromus* from South Africa, sexes and species combined.

	Mean	Min	Max	n
LLTR	2.8	2.5	3.2	67
WM_1_	0.8	0.6	0.9	67
LUTR	3.1	2.7	3.5	68
WM^1^	0.9	0.8	1.1	68

Dental formula is 1-0-0-3:1-0-0-3. Alveolar formula is 4-3-1:2-2-1 (Figs 16 and 17).

#### Upper jaw

Upper incisors are opisthodont and have one pronounced groove, located closer to the outer margin than the inner (a character shared with other Dendromurinae). There is a masseteric knob at the lower anterior corner of the zygomatic plate. Anterior palatal foramina extend beyond the first row of cusps of M^1^. Molars have a typical Dendromurinae dental pattern, but cusps are more individualized than in *Steatomys*. The M^1^ is the longest of the toothrow, being twice as large as the M^2^. It lacks the cusp t1 but has a rather large t4 connected to the t5. There is a groove separating the central (t2, t5 and t8) and labial (t3, t6, t9) cusps (not marked in *Steatomys*). The M^2^ lacks the t1 and has a small to tiny t3; the t4 is well-developed and connected to the t5. Molar M^3^ is very small and corresponds to Group 1 in Fig 12.

#### Lower jaw

Lower incisors are ungrooved (one groove in upper incisors). The prelobe of M_1_ consists of a single median cusp. It is connected to the first row of cusps, which is oblique and constituted by a lingual metaconid connected to the labial protoconid located further back. The second row of cusps is equally oblique, with a well-developed hypoconid and a very large posterior cingulum located in the postero-lingual angle of the tooth. The M_2_ has two oblique rows of cusps, with conspicuous anterolabial and posterior cingula. The M_3_ is very small. As in other Dendromurinae, muscle attachment on the mandible is situated rather far from the mental foramen, which can help to distinguish *Dendromus* from *Mus*.

#### Systematic notes and South African fossil record

The classification and species delimitation of the genus *Dendromus* has changed considerably over the last decades. Four species of *Dendromus* are listed in Monadjem et al. [22] for South Africa:

- *Dendromus melanotis* Smith, 1834
- *Dendromus mesomelas* (Brain, 1827)
- *Dendromus mystacalis* Heuglin, 1863
- *Dendromus nyikae* Wroughton, 1909

Recent molecular systematic assessment published by Voelker et al. [72] led to the resurrection of the genus *Poemys*. According to this, *D*. *nyikae* and *D*. *melanotis* would rather be identified as *P*. *nyikae and P*. *melanotis*, with the latter species probably comprising at least four species (*P*. *arenarius*, *P*. *basuticus*, *P*. *melanotis*, *P*. *vulturnus*). The two species *D*. *mesomelas* and *D*. *mystacalis* remain attributed to *Dendromus* and include many lineages and cryptic species not yet described. Consequently we prefer to keep the traditional taxonomy pending further revisions of South African *Dendromus* species. Additional fossil species have been described in South Africa:

- *†Dendromus antiquus* Broom, 1946 from the Late Pliocene of Taung, considered by Avery [4] as *nomen nudum*
- *†Dendromus averyi* Denys, 1994 from the Early Pliocene of Langebaanweg [73]
- *†Dendromus darti* Denys, 1994 from the Early Pliocene of Langebaanweg [73]

Material assigned to this genus has been recovered from many Quaternary fossil deposits in South Africa [50].

Genus ***Malacothrix*** Wagner, 1843 (Long-eared Mouse)

**Fig 78:**
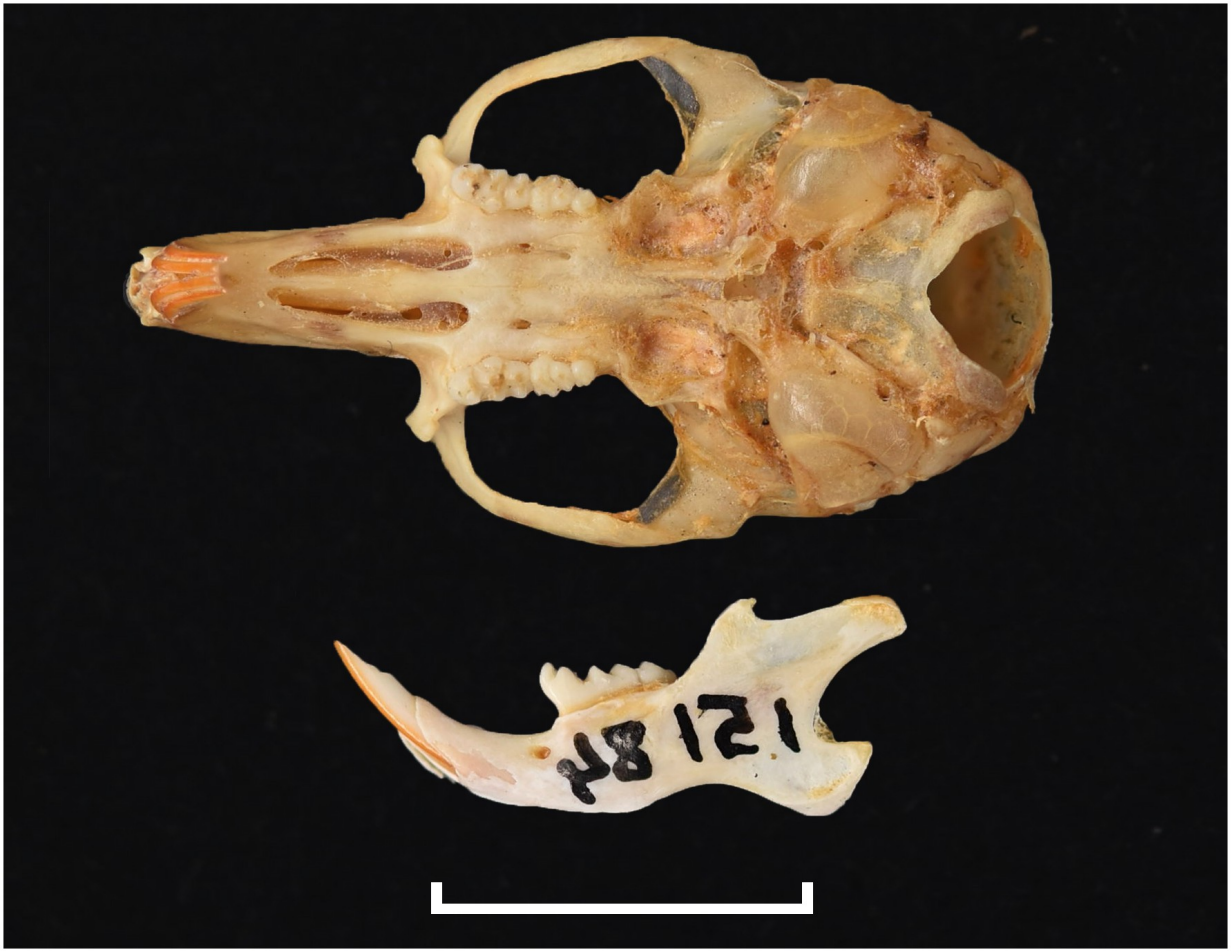
Cranium of *Malacothrix typica* (skull-DNMNH 4965; mandible DNMNH-15184), with scale bar of 1 cm.

**Fig 79:**
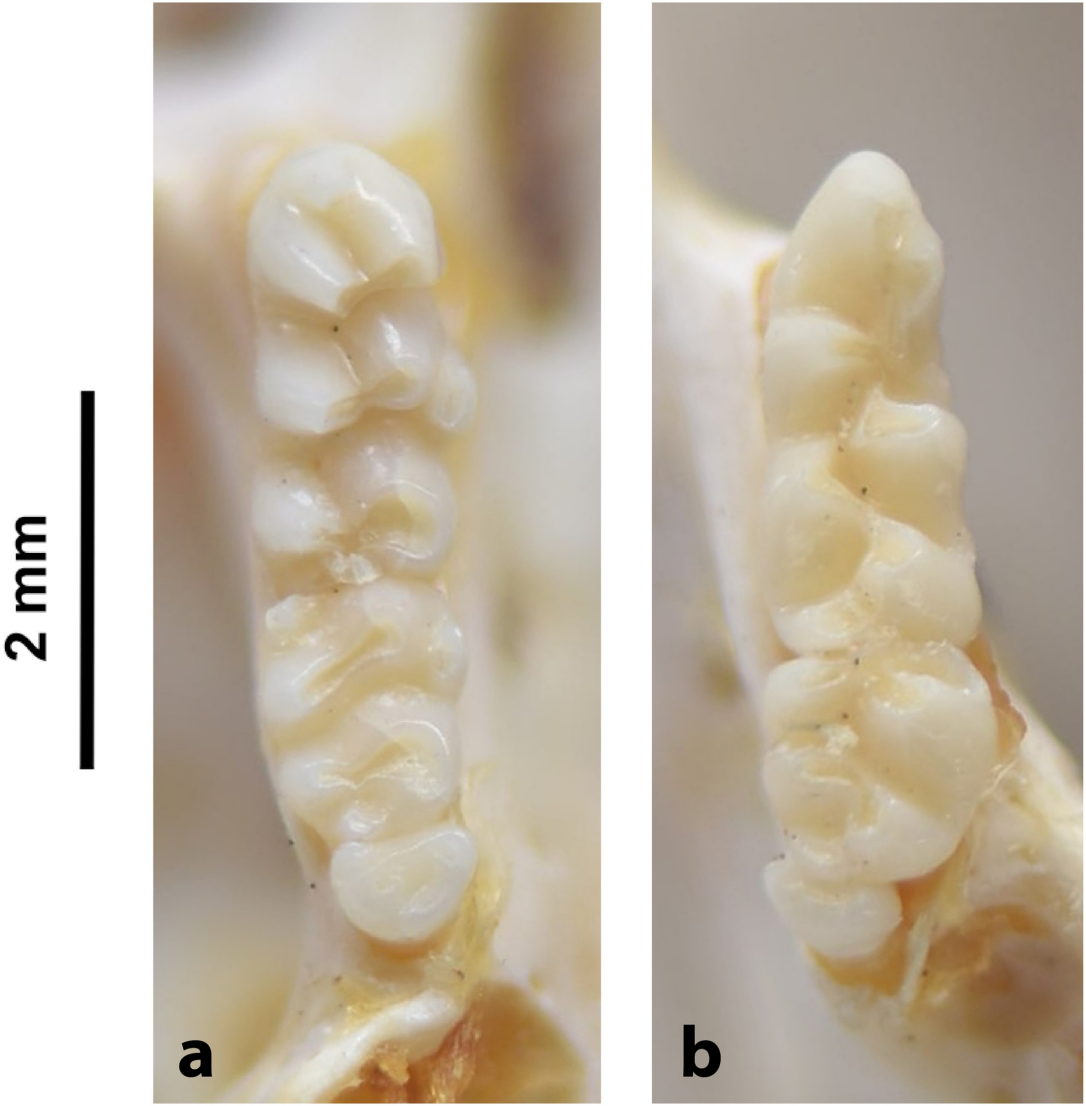
Upper (a) and lower (b) right toothrow of *M*. *typica* (DNMNH-15184).

**Fig 80:**
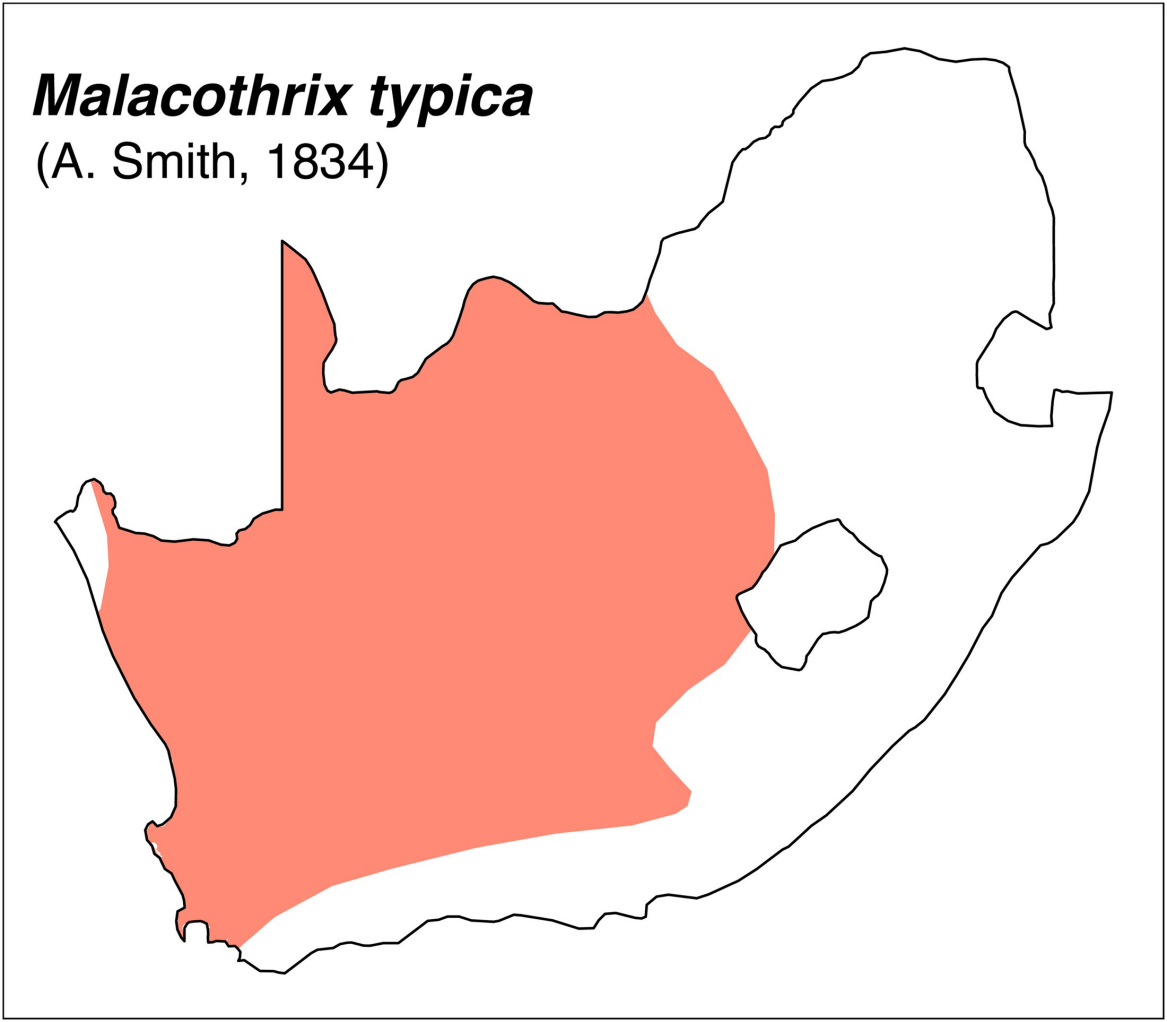
Distribution map.

Figs 78–80; Table 27

**Table 27:** Dental measurements (in mm) for *Malacothrix typica*, sexes combined.

	Mean	Min	Max	n
LLTR	3.7	3.3	4.2	9
WM_1_	1.0	0.9	1.1	9
LUTR	4.0	3.6	4.5	9
WM^1^	1.1	1.0	1.2	9

Dental formula is 1-0-0-3:1-0-0-3. Alveolar formula is 3-3-1:2-2-1 (Figs 16 and 17).

#### Upper jaw

Upper incisors are opisthodont and have one groove (as other members of Dendromurinae). Anterior palatal foramina extend beyond the first row of cusps of M^1^. Molars exhibit the typical Dendromurinae pattern, but the M^1^ is very long, occupying half of the length of the toothrow. The M^1^ has a well-marked antero-median cusp. The M^3^ is very small and corresponds to Group 1 in Fig 12. The three alveoli of the M^1^ are almost in line, with the lingual alveolus less displaced lingually than in *Dendromus* and *Steatomys*. There is a ridge-shaped masseter knob on the lower anterior of the zygomatic plate (sub-circular in *Dendromus* and *Steatomys*).

#### Lower jaw

Lower incisors are narrow and ungrooved. The molars display round, well separated bunodont cusps, with the typical Dendromurinae cusp pattern. The M_1_ is narrow and very long, occupying more than half of the length of the molar toothrow; it displays seven highly alternated cusps separated by a longitudinal crest. The M_2_ has an antero-external cusp. The M_3_ is tiny and has a single cusp. The ventral edge of the mandible body between the ramus and the angular process is markedly curved. The posterior curvature between angular and coronoid process is well pronounced. As in other Dendromurinae, muscle attachment on the mandible is situated rather far from the mental foramen.

#### Systematic notes and South African fossil record

This genus is monotypic:

- *Malacothrix typica* (A. Smith, 1834)

An additional fossil species has been described in South Africa:

- *†Malacothrix makapani* De Graaff, 1961

The oldest remains of *Malacothrix* are from Makapansgat around 3.3 MYA [48]. It has been recorded in several Quaternary fossil deposits in South Africa [50].

Genus ***Steatomys*** Peters 1846 (Fat Mice)

**Fig 81:**
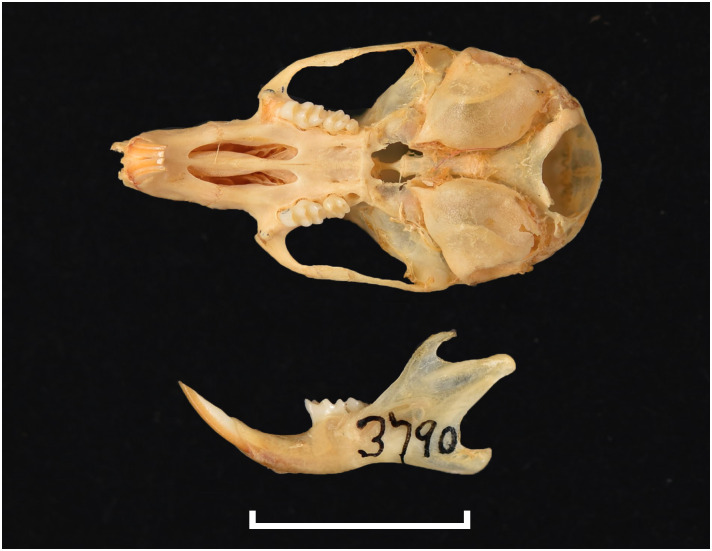
Cranium of *Steatomys krebsii* (skull DNMNH-3792, mandible skull DNMNH-3790), with scale bar of 1 cm.

**Fig 82:**
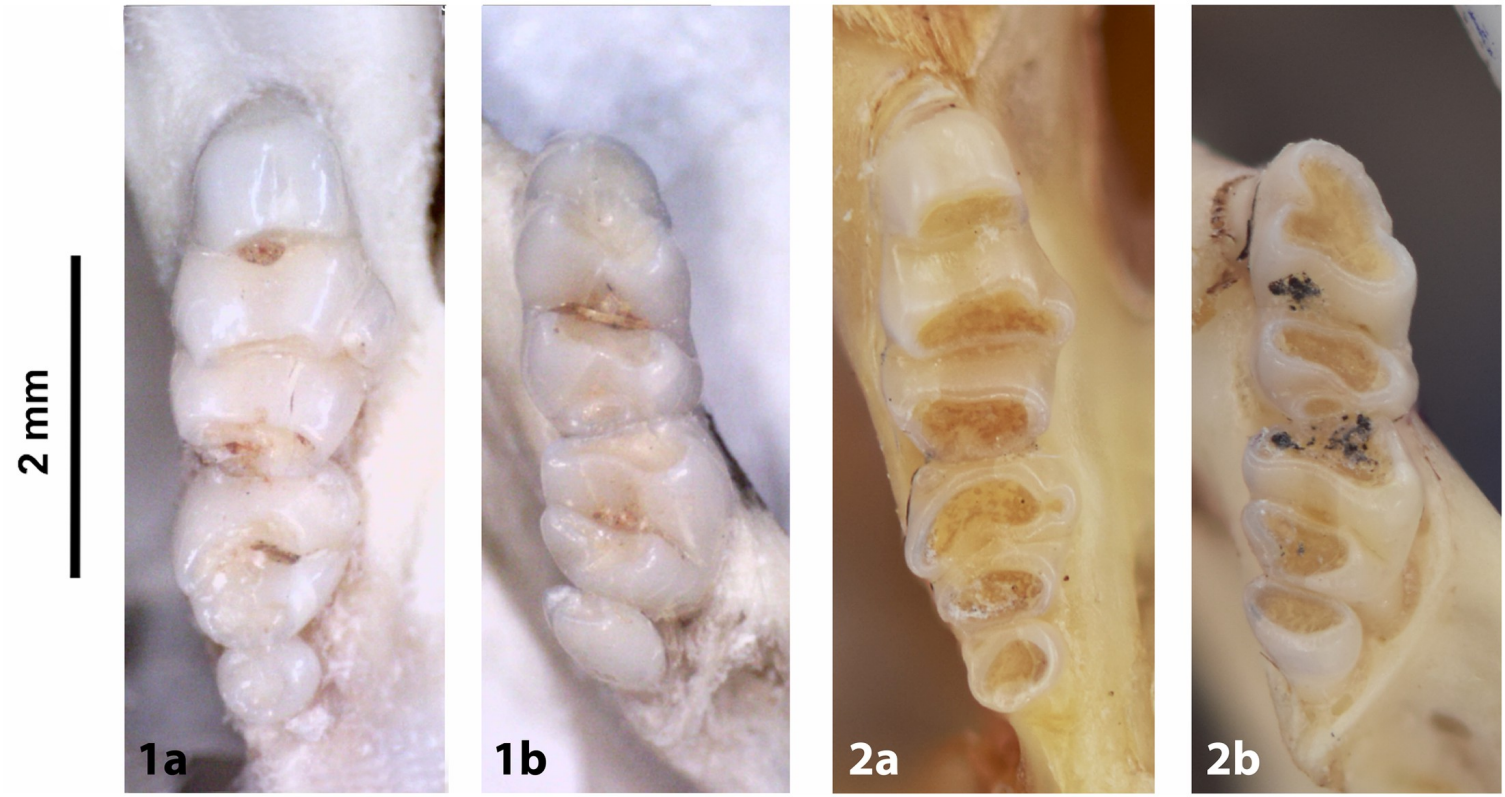
Cheekteeth of *Steatomys*. **1)** Upper (a) and lower (b) right toothrow of *S*. *pratensis* (RMCA-96-037-M-5108); **2)** Upper (a) and lower (b) right toothrow of *Steatomys* sp. (DNMNH-3458).

**Fig 83:**
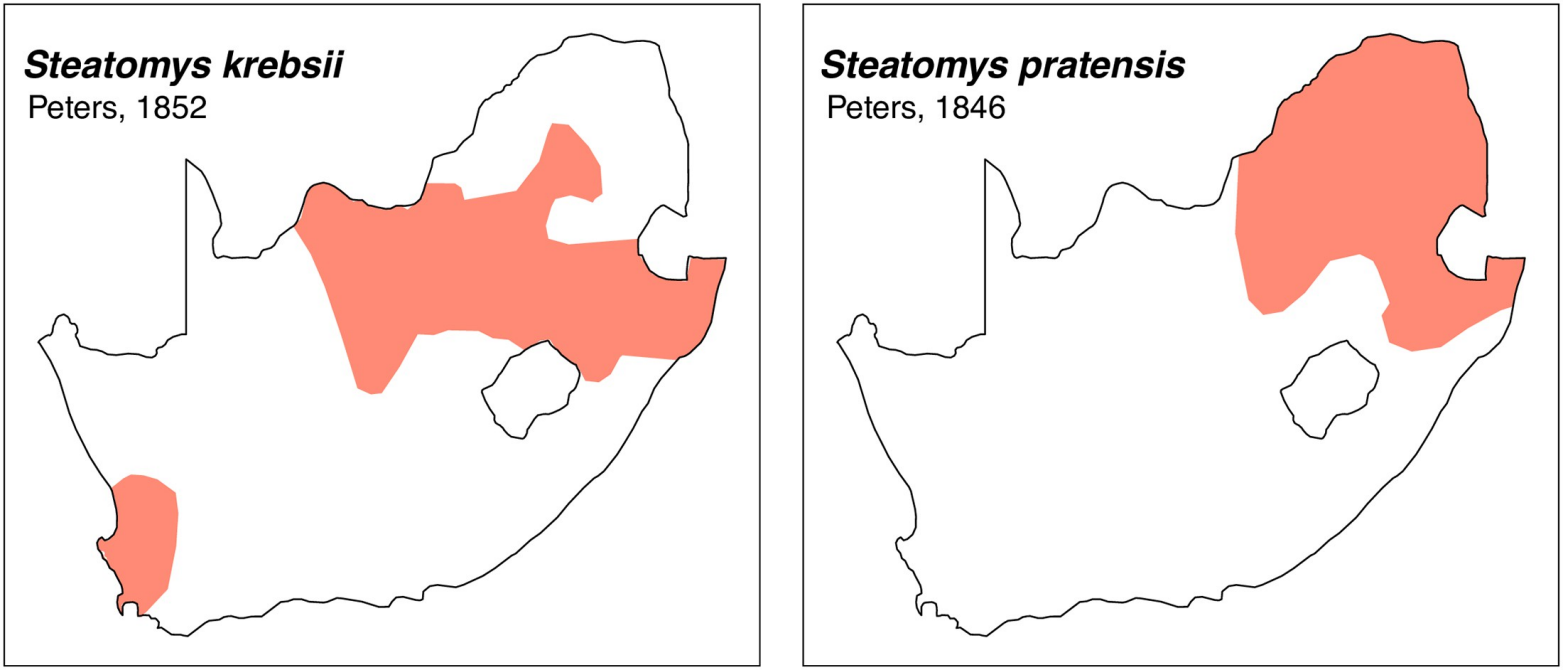
Distribution maps.

Figs 81–83; Table 28

**Table 28:** Dental measurements (in mm) for *Steatomys* from South Africa, sexes and species combined.

	Mean	Min	Max	n
LLTR	3.4	3.1	3.7	30
WM_1_	1.1	0.9	1.2	30
LUTR	3.8	3.3	4.2	30
WM^1^	1.3	1.1	4.1	30

Dental formula is 1-0-0-3:1-0-0-3. Alveolar formula is 3-3-1:2-2-1 (Figs 16 and 17).

#### Upper jaw

Upper incisors are opisthodont and have one pronounced groove, located closer to the outer margin than the inner. Anterior palatal foramina are long and extend beyond the first row of cusps of M^1^. Molars have a typical Dendromurinae cusp pattern (with t1 and t7 missing in M^1^ and M^2^) but cusps are rounder than in *Dendromus* and *Malacothrix*. The M^1^ is the longest of the toothrow and has three lobes: the first lobe has two cusps that are connected and project posteriorly; the second lobe has three cusps with a t4 closely connected to the t5 but located slightly behind; the third lobe has two cusps and a posterior cingulum, which is connected to the t8 and delimits a fovea. The M^2^ has two lobes and a small t3. The M^3^ is small (although generally not as reduced as in *Dendromus*) and corresponds to Group 1 in Fig 12.

#### Lower jaw

Lower incisors are ungrooved. Like *Dendromus*, the M_1_ has a median prelobe connected to the first oblique row of cusps and a median posterior cingulum below the second oblique row. The M_2_ has two lobes with an anterolabial cingulum and a small to medium posterior cingulum. The M_3_ is small and consists of a single cusp. As in other Dendromurinae, muscle attachment on the mandible is situated rather far from the mental foramen.

#### Systematic notes and South African fossil record

Two species are recognised in South Africa:

- *Steatomys krebsii* Peters, 1852
- *Steatomys pratensis* Peters, 1846

In South Africa, the oldest known fossils are from Makapansgat around 3.3 MYA [48]. Remains of this genus have been recorded in several Quaternary fossil deposits [50].

Subfamily **MYSTROMYINAE** Vorontsov, 1966

Genus ***Mystromys*** Wagner, 1841 (African White-tailed Rat)

**Fig 84:**
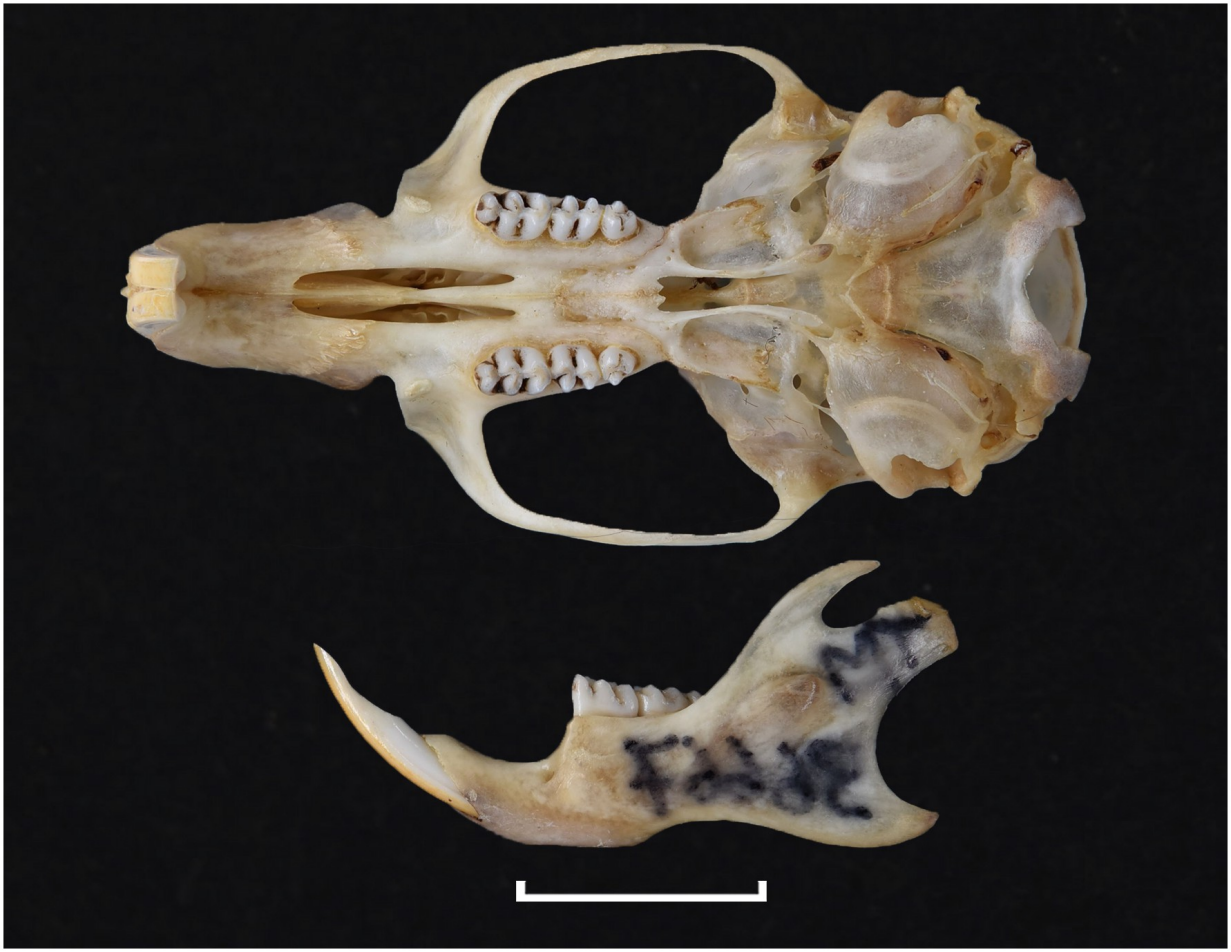
Cranium of *Mystromys albicaudatus* (skull DNMNH-5986, mandible DNMNH-8813), with scale bar of 1 cm.

**Fig 85:**
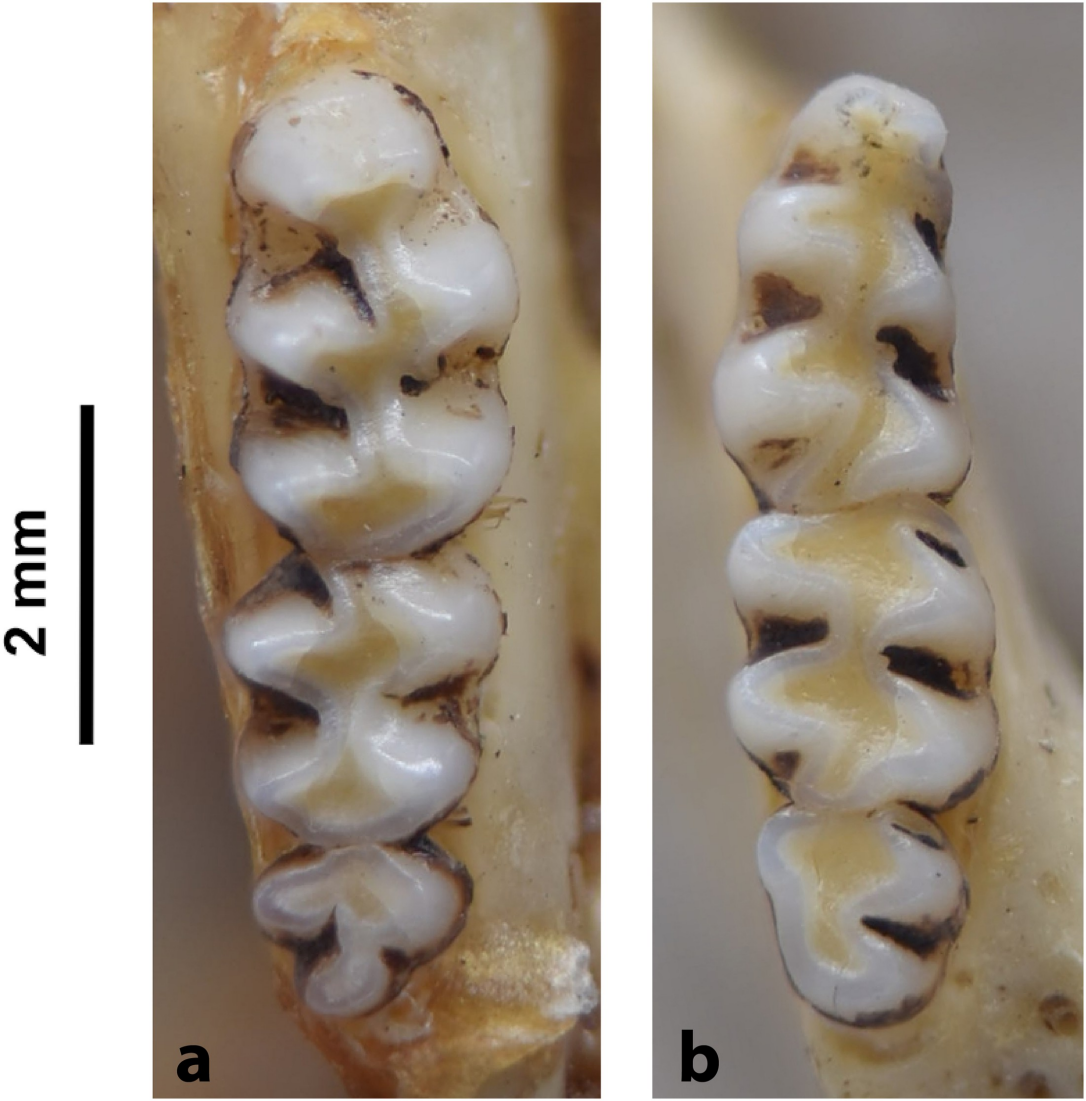
Upper (a) and lower (b) right toothrow of *M*. *albicaudatus* (DNMNH-3666).

**Fig 86:**
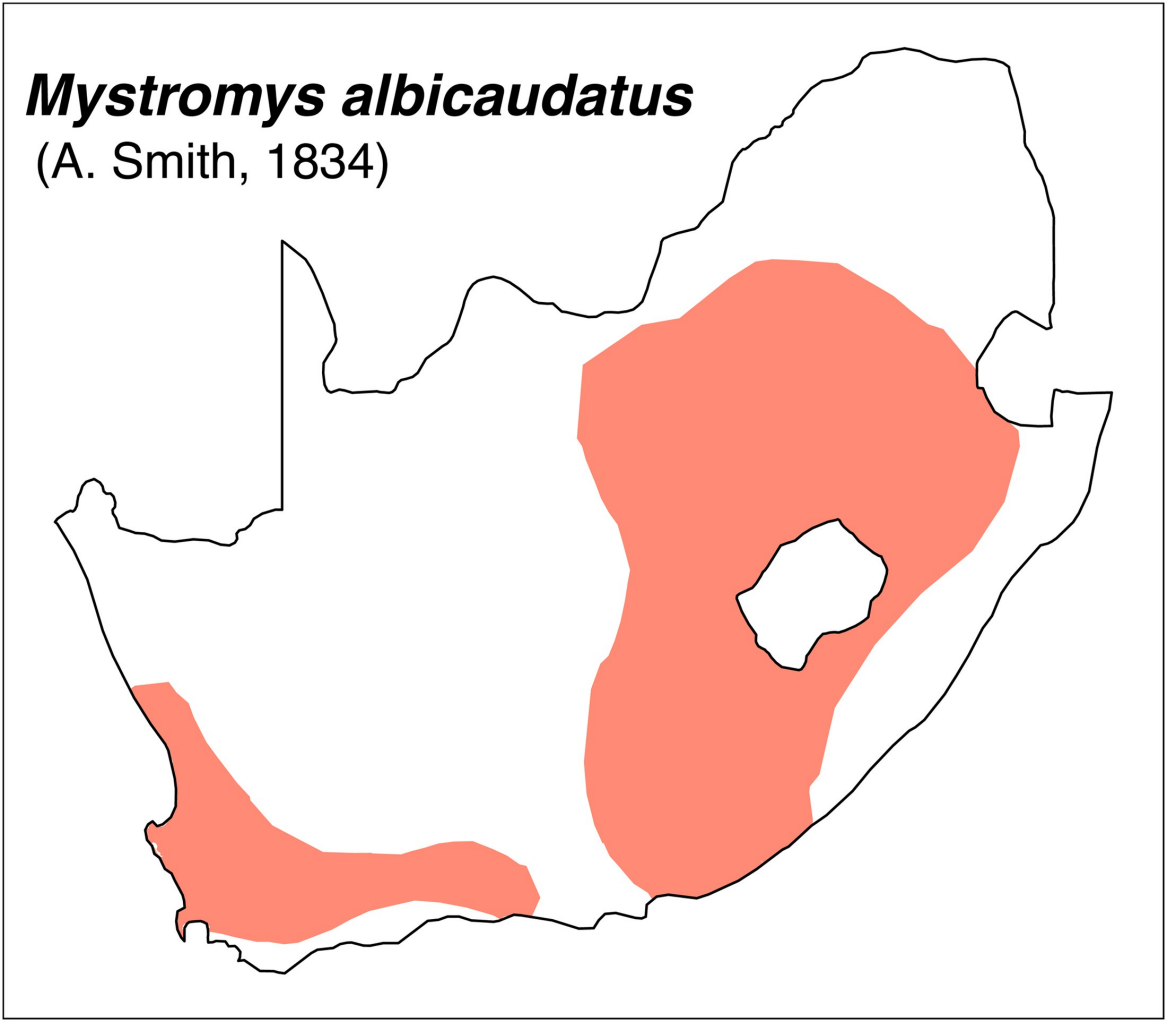
Distribution map.

Figs 84–86; Table 29

**Table 29:** Dental measurements (in mm) for *Mystromys albicaudatus*, sexes combined.

	Mean	Min	Max	n
LLTR	6.1	5.4	6.8	41
WM_1_	1.7	1.4	1.9	40
LUTR	6.0	5.5	6.8	41
WM^1^	1.8	1.6	2.0	41

Dental formula is 1-0-0-3:1-0-0-3. Alveolar formula is 3-3-2:2-2-2 (Figs 16 and 17).

#### Upper jaw

Upper incisors are orthodont and display typical striations on the anterior enamel surface that form a slightly raised band. The palatal foramina are long and extend past the M^1^. The cheekteeth have well separated opposed cusps that are connected by a longitudinal crest, resulting in a characteristic zigzag pattern. M^1^ has three lobes with two lingual and two labial folds; the first lobe has one to two fused cusps, the second and third lobe have two alternated cusps. M^2^ has two lobes each with two cusps. M^3^ is small and has two lobes, the last one consisting of a single cusp.

#### Lower jaw

Lower incisors display characteristic raised enamel bands. Cusps of lower molars have alternate cusps connected by a longitudinal crest, which results in a distinctive zigzag pattern. Molar M_1_ has three lingual and two labial folds. Molar M_2_ has two lingual and two labial folds. Molar M_3_ has three cusps with a small antero-external cingulum.

#### Systematic notes and South African fossil record

This genus is monotypic today:

- *Mystromys albicaudatus* (A. Smith, 1834)

Additional species have been described in South African fossil deposits:

- *†Mystromys hausleitneri* Broom, 1937 from various Pleistocene sites, which is often regarded as a chronospecies that closely resembles the present-day form, albeit slightly smaller [4, 47, 74].
- *†Mystromys pocockei* Denys, 1991 from the Early Pliocene site of Langebaanweg

Fossils of this genus are known from many Pleistocene sites in South Africa [50]. In the karst deposits of the Sterkfontein Valley from the Gauteng Province, it is often one the most abundant taxa from the micromammal assemblages.

† Genus ***Proodontomys*** Pocock, 1987

**Fig 87:**
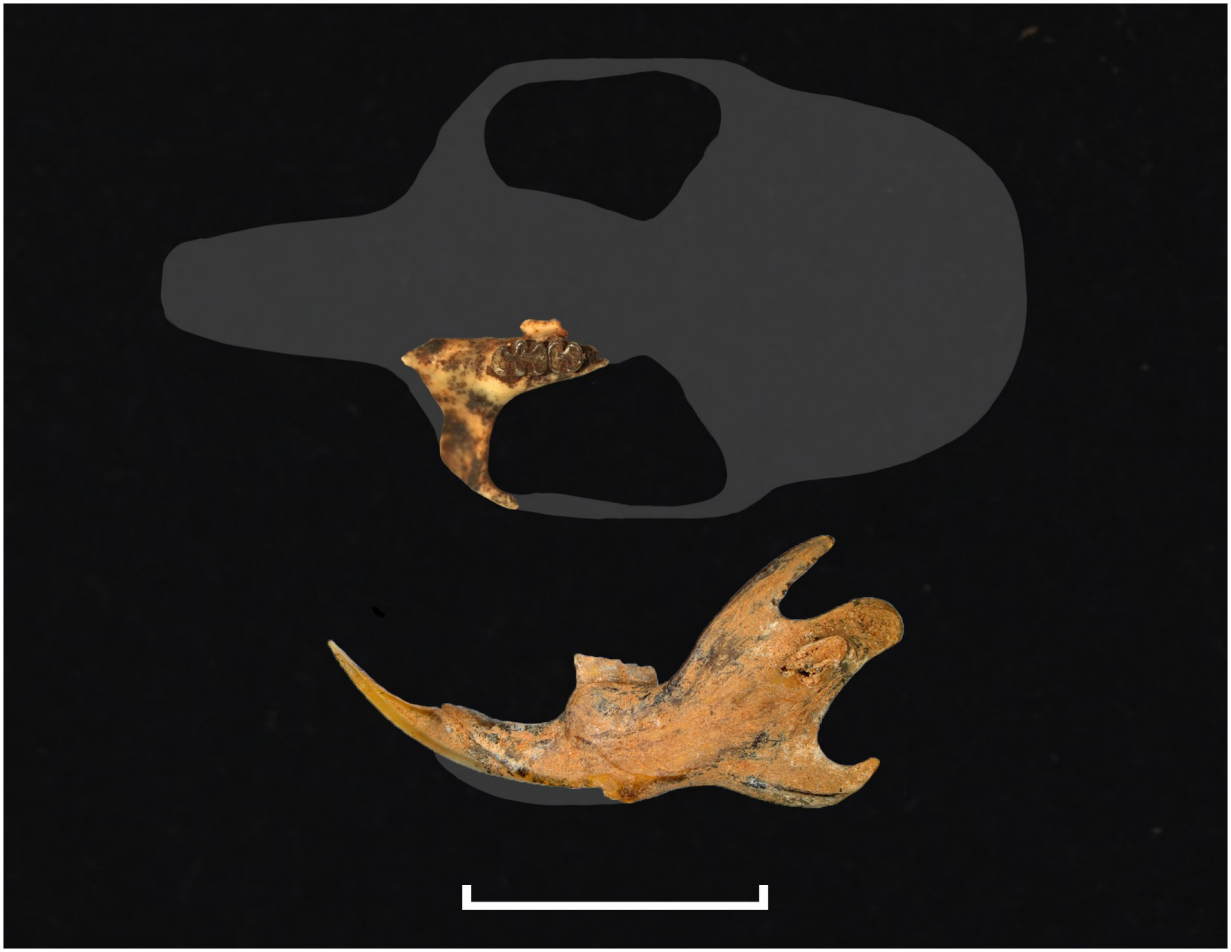
Cranium of *Proodontomys cookei* (ESI T.N. Pocock microfaunal fossil collection), with scale bar of 1 cm.

**Fig 88:**
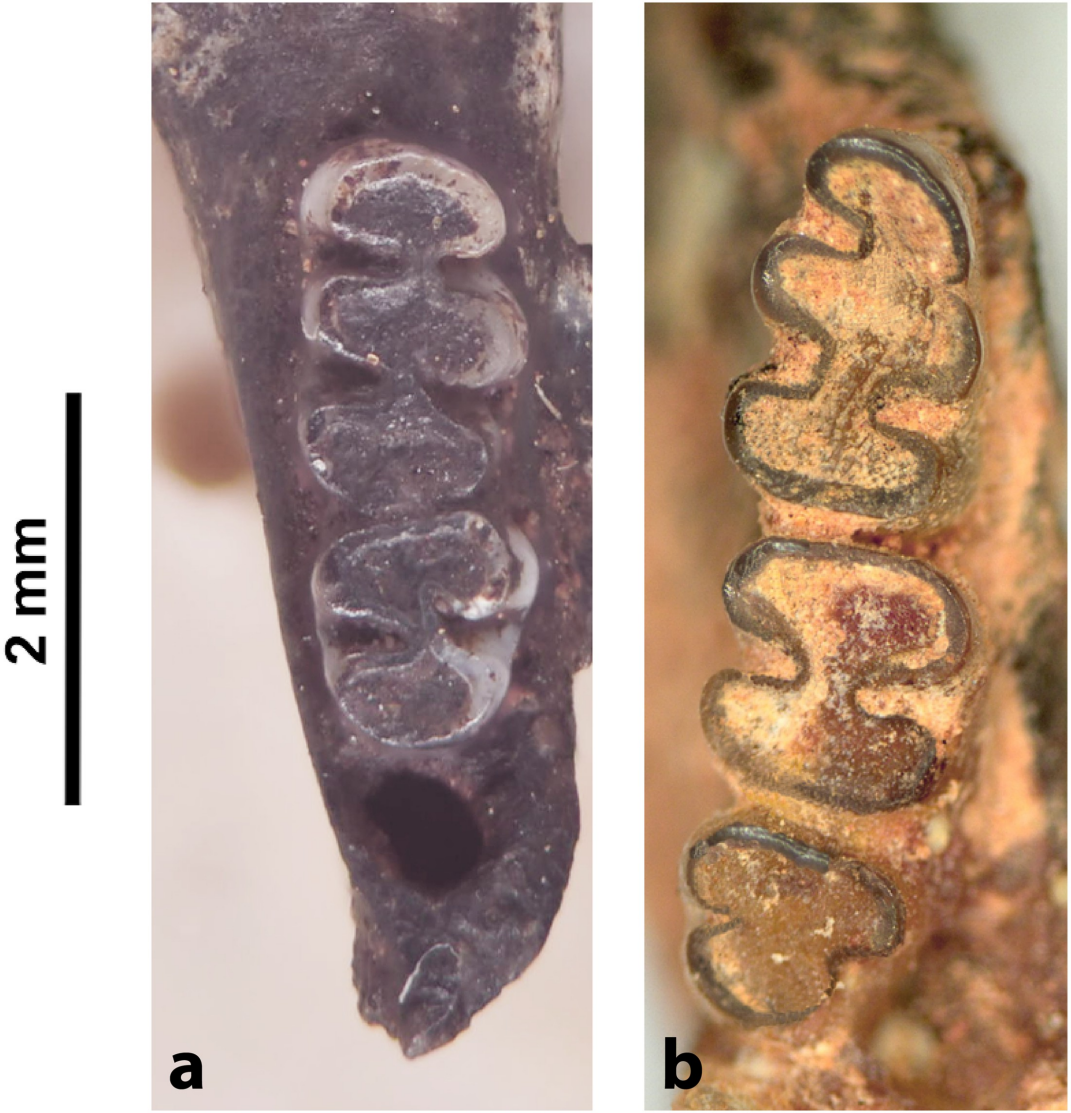
Cheekteeth of *Proodontomys*. Upper (a) and lower (b) right toothrow of *P*. *cookei* (upper ESI, Cooper’s D fossil collection CD1980; lower ESI T.N. Pocock microfaunal fossil collection, EXQRM47). The molar M^3^ is missing.

Figs 87 and 88; Table 30

**Table 30:** Dental measurements (in mm) for *Proodontomys cookei*, sexes combined.

	Mean	Min	Max	n
LLTR	4.2	3.6	4.6	9
WM_1_	1.1	1.0	1.2	35
LUTR	4.3	4.0	4.5	7
WM^1^	1.3	1.2	1.4	2

Dental formula is 1-0-0-3:1-0-0-3. Alveolar formula is 3-3-1:2-2-1/2 (Figs 16 and 17).

#### Upper jaw

Upper incisors are highly proodont and ungrooved; they do not display the typical ridge seen in *Mystromys*. Anterior palatal foramina are long and penetrate between the second rows of cusps in M^1^. Molars show a tendency to hypsodonty, with weak cusps that quickly wear down to a plain occlusal surface. As in *Mystromys*, the cheekteeth lack lateral cusps, but they differ by having opposite, rather than alternate, molar cusps, resulting in a semi-lophodont dentition. The M^1^ has three lobes, the M^2^ has two lobes, and the M^3^ has two poorly differentiated lobes that tend to fuse with wear to form a simple cylindrical tooth.

#### Lower jaw

Lower incisors are ungrooved and do not display the typical ridge present in *Mystromys*. As in upper molars, molars have week cusps that flatten in early wear into a plain, semi-lophodont occlusal surface. The molar M_1_ shows a trilophodont pattern, and both the M_2_ and M_3_ are bilophodont. The coronoid process of the mandible is very large.

#### Systematic notes and South African fossil record

This genus got extinct around 1 MYA, and a single species has been described in South Africa fossil deposits:

- †*Proodontomys cookei* Pocock, 1987

The oldest remains of *Proodontomys* come from the locality of Limeworks Makapansgat around 3.3 MYA [48].

Subfamily **PETROMYSCINAE** Roberts, 1951

Genus ***Petromyscus*** Thomas, 1926 (Pygmy Rock Mice)

**Fig 89:**
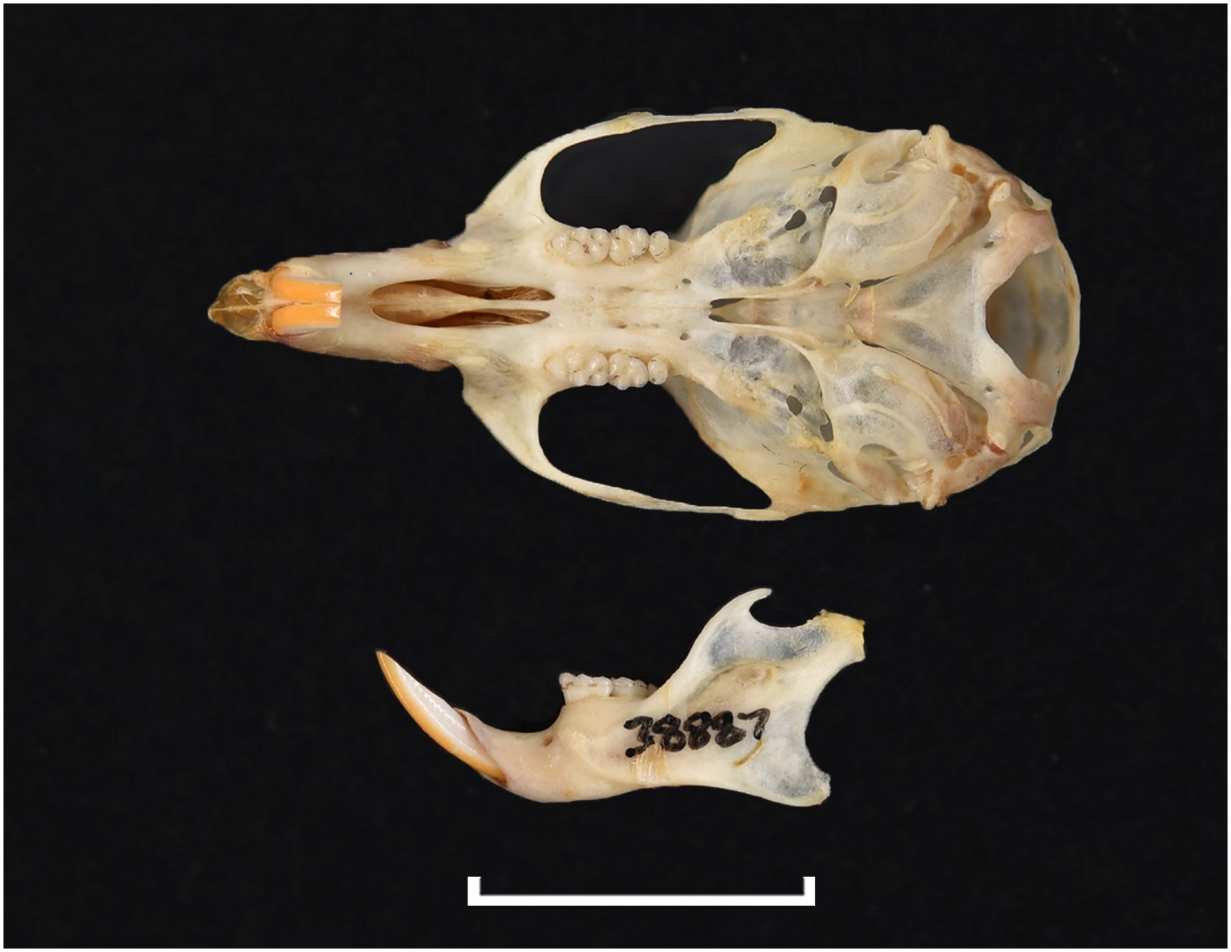
Cranium of *Petromyscus collinus* (DNMNH-38887), with scale bar of 1 cm.

**Fig 90:**
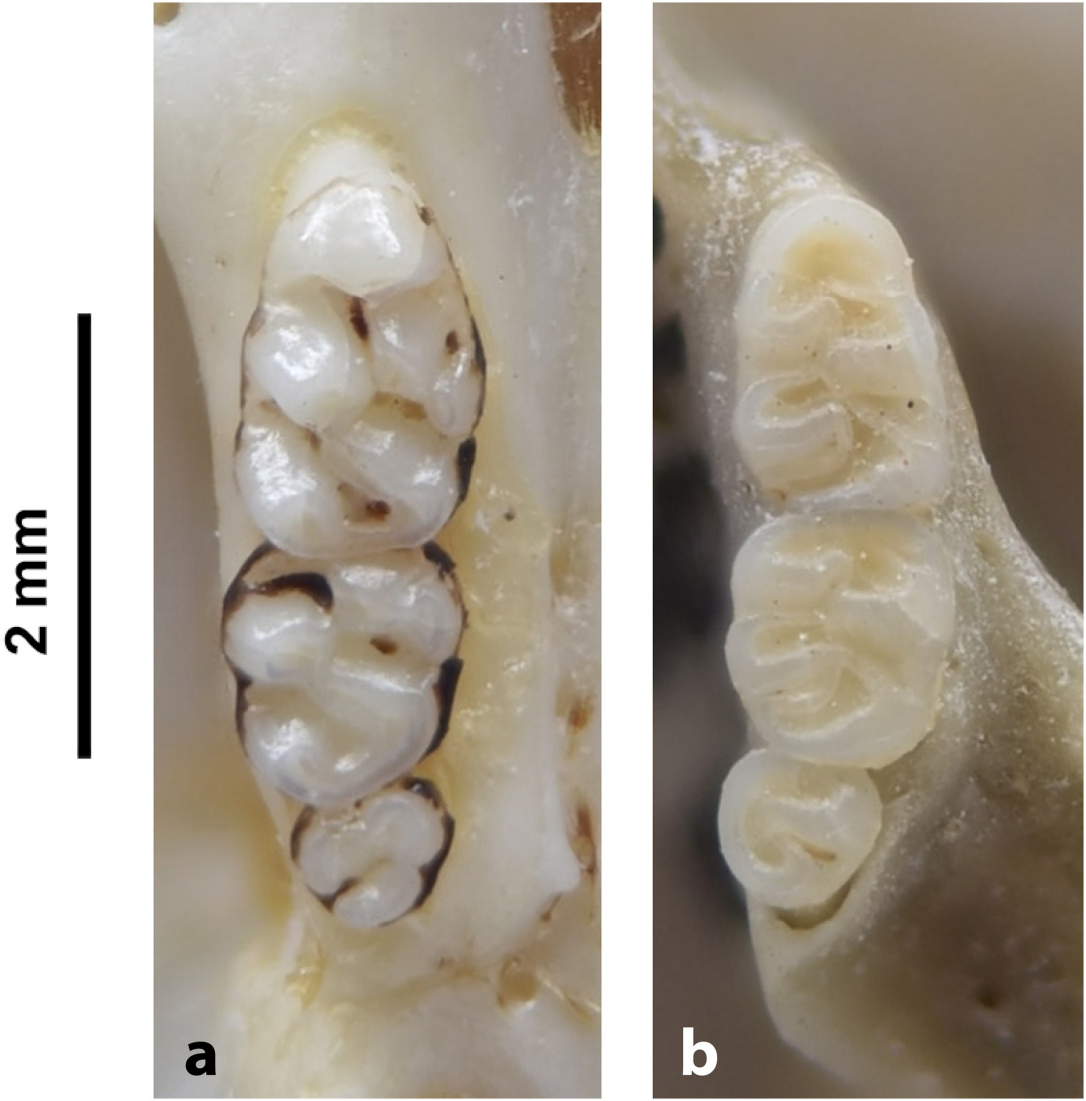
Upper (a) and lower (b) right toothrow of *P*. *shortridgei* (upper DNMNH-23111; lower DNMNH-29253).

**Fig 91:**
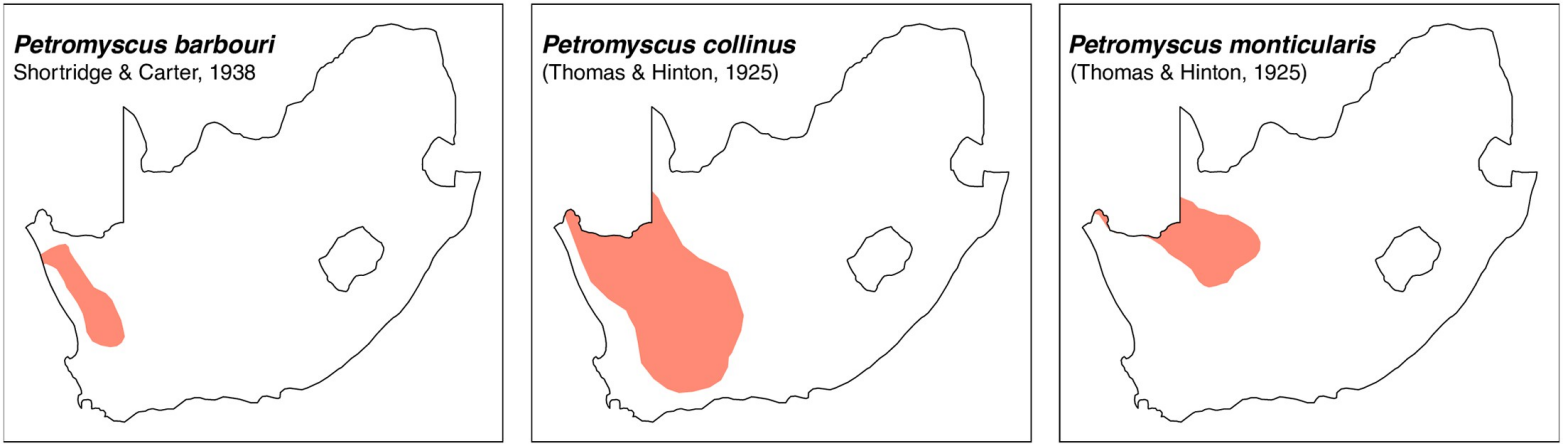
Distribution map.

Figs 89–91; Table 31

**Table 31:** Dental measurements (in mm) for *Petromyscus* from South Africa, sexes and species combined.

	Mean	Min	Max	n
LLTR	3.3	2.9	3.7	13
WM_1_	1.0	0.8	1.0	13
LUTR	3.5	3.3	3.9	10
WM^1^	1.1	1.0	1.2	12

Dental formula is 1-0-0-3-:1-0-0-3. Alveolar formula is: 3-3-1:2-2-1 (Figs 16 and 17).

#### Upper jaw

Upper incisors are opisthodont and ungrooved. Cheekteeth are relatively small. Anterior palatal foramina reach the margin or the first lobe of the M^1^. Molars display round cusps connected by a median longitudinal crest, resulting in a zigzag pattern reminiscent of *Mystromys*. The M^1^ has three lobes: the first has one or two fused cusps, the second has three cusps including a t4 attached to the t5 (which distinguishes this genus from the members of *Mystromyinae*), the third has two cusps. The M^2^ has two lobes as well as an antero-external cusp (t3) but no t4. The M^3^ is small, having a tiny t3 (t1 is absent) and two rows of cusps connected lingually. It corresponds to Group 1 in Fig 12.

#### Lower jaw

Lower incisors are ungrooved. The molar M_1_ has three lobes, which are connected in their median part by a longitudinal crest. Molar M_3_ is small and display a tiny antero-external cingulum.

#### Systematic notes and South African fossil record

Three species are presently recognized in South Africa:

- *Petromyscus barbouri* Shortridge & Carter, 1938
- *Petromyscus collinus* (Thomas & Hinton, 1925)
- *Petromyscus monticularis* (Thomas & Hinton, 1925)

Remains of *Petromyscus* are scanty in the Quaternary fossil record, and the oldest fossils in South Africa are known from Holocene sites [50].

### Bathyergidae

Mole-rats of the family Bathyergidae (Tables 32 and 33) are fossorial (adapted to digging or burrowing) rodents and exhibit numerous morphological adaptations for an underground life. Some four genera occur in South Africa. They live in tunnel systems and are almost never seen above the ground’s surface [21]. They mostly become vulnerable during the mound formation, when predators are able to locate them with accuracy. Only juveniles of *Bathyergus* and *Georychus* are small enough to be captured by owls such as *Tyto alba* (Barn owl), and *Bubo africanus* (Spotted eagle-owl), which are the main accumulators of Quaternary fossil micromammal deposits in South Africa. As a result, it is common to find mainly young individuals among fossil assemblages accumulated by these predators, with specimens showing only two or three completely erupted cheekteeth *versus* the four cheekteeth found in adult specimens. Members of Bathyergidae display distinctly proodont upper incisors. The lower jaw is hystricomorph in character, showing a characteristic flared and inflected angular process on the mandible. In South African mole-rats, the dental formula is always 1-0-1-3:1-0-1-3 and the anterior palatal foramina are small, ending well before the M^1^.

**Table 32:** Key to the bathyergid genera: Upper jaw.

1	molars with folds of enamel	*Georychus*
	molars without folds of enamel	2
2	all molars are approximately the same size; incisors grooved	*Bathyergus*
	M^3^ is smaller; incisors are ungrooved	*Cryptomys/Fukomys*

**Table 33:** Key to the bathyergid genera: Lower jaw.

1	molars with folds of enamel	*Georychus*
	molars without folds of enamel	2
2	LLTR > 8 mmm	*Bathyergus*
	LLTR < 7 mm	*Cryptomys/Fukomys*

Family **BATHYERGIDAE** Waterhouse, 1841

Genus ***Bathyergus*** Illiger, 1811 (Dune Mole-rats)

**Fig 92:**
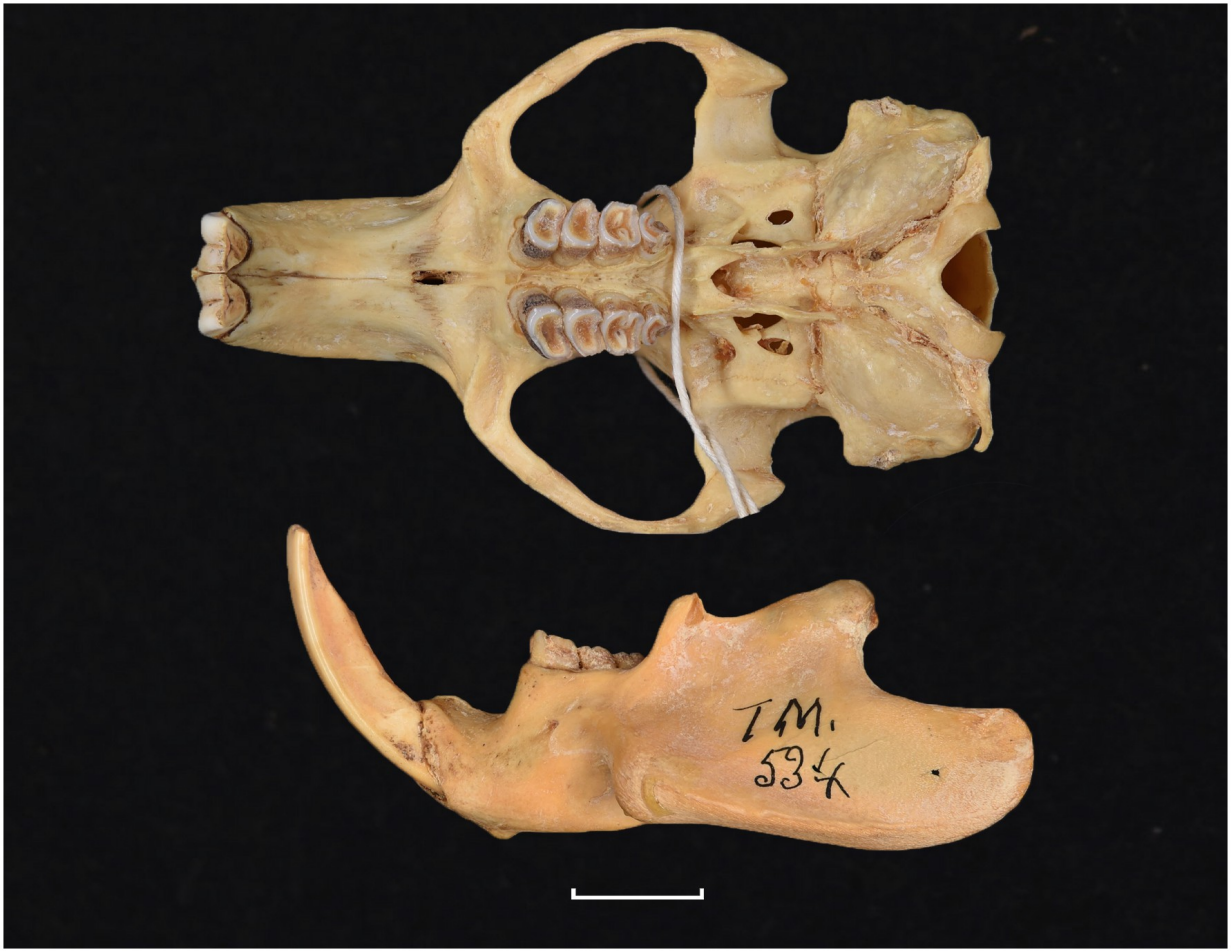
Cranium of *Bathyergus suillus* (skull DNMNH 523; mandible DNMNH-534), with scale bar of 1 cm.

**Fig 93:**
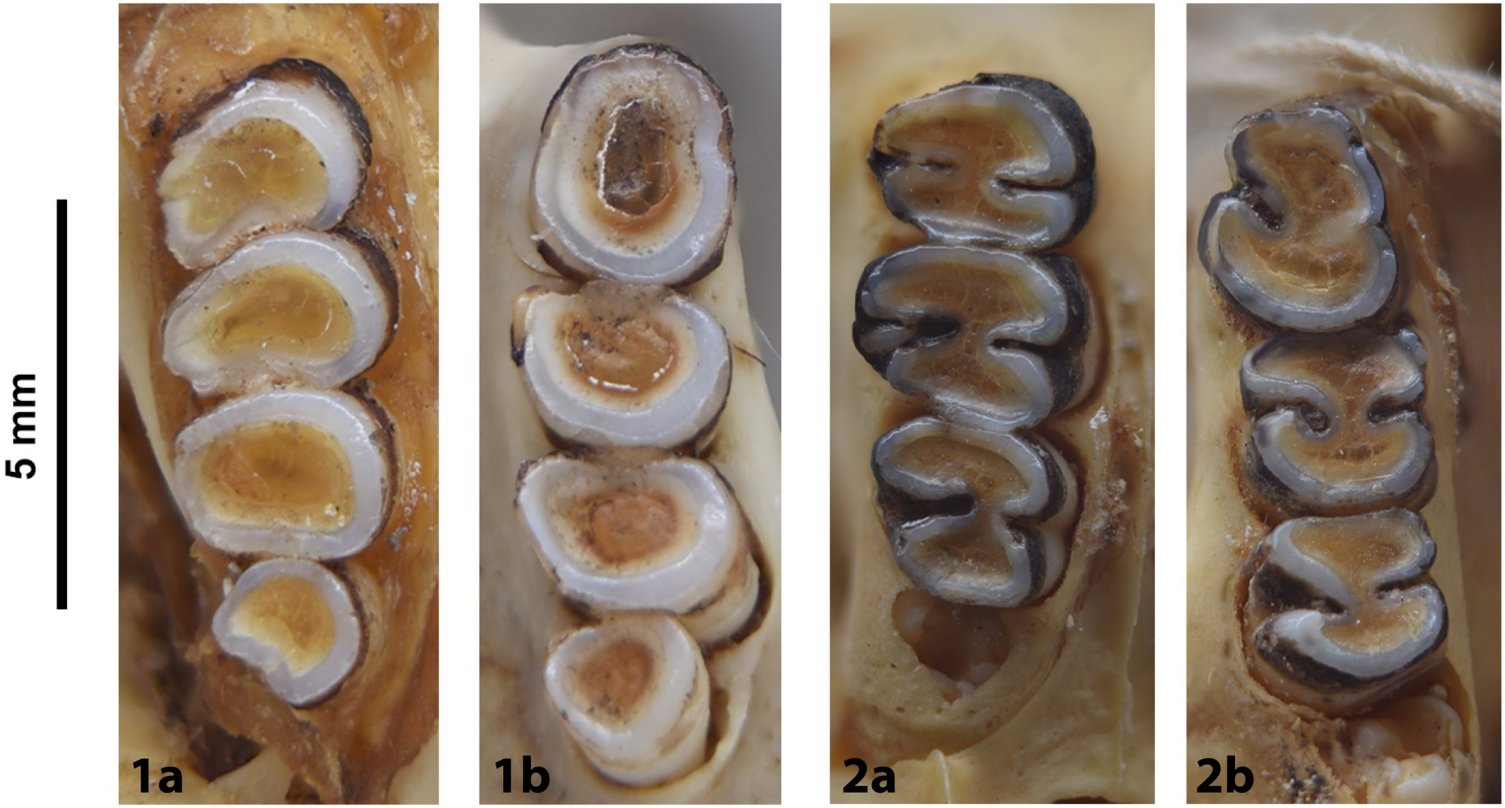
Cheekteeth of *Bathyergus*. **1)** Upper (a) and lower (b) right toothrow of an adult *B*. *janetta* (upper DNMNH-543; lower DNMNH-39304); **2)** Upper (a) and lower (b) right toothrow of a juvenile *B*. *suillus* (DNMNH-2162), with molars M^3^/M_3_ not erupted yet.

**Fig 94:**
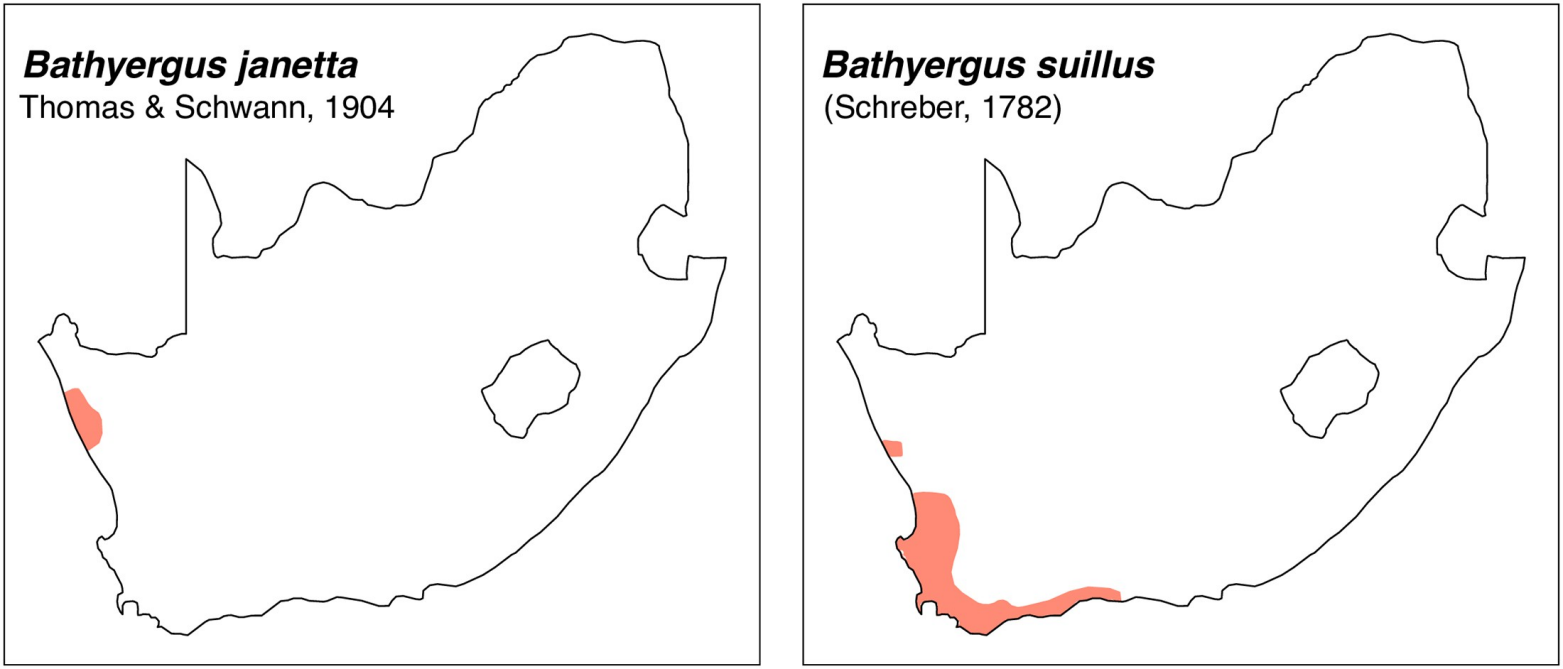
Distribution maps.

Figs 92–94; Table 34

**Table 34:** Dental measurements (in mm) for *Bathyergus* from South Africa, sexes and species combined.

	Mean	Min	Max	n
LLTR	11.0	8.8	13.5	19
WM_1_	3.5	2.4	4.7	20
LUTR	10.5	8.1	12.8	19
WM^1^	3.7	2.7	4.6	17

Dental formula is 1-0-1-3:1-0-1-3. Alveolar formula varies with age of specimen.

#### Upper jaw

Upper Incisors are proodont, robust and heavily grooved; they are less protruding than in other genera and are rooted above the anterior cheekteeth. Anterior palatal foramina are short, ending before molar M^1^. Cheeekteeth are hypsodont and simple (rounded and uncusped), but young animals display re-entrant folds.

#### Lower jaw

Lower incisors are plain. Lower cheekteeth are similar to upper cheekteeth. The mandible is hystricognathous and stickily built, with the angular portion extending well posteriorly. The mental foramen is positioned posteriorly to the diastema, aligned with the posterior border of the M_1_.

#### Systematic notes and South African fossil record

Two species of *Bathyergus* occur in South Africa:

- *Bathyergus janetta* Thomas & Schwann, 1904
- *Bathyergus suillus* (Schreber, 1782)

An additional fossil species has been described:

- †*Bathyergus hendeyi* Denys, 1998 from the Early Pliocene of Langebaanweg

Genus ***Cryptomys*** Gray, 1864 (Mole-rats)

**Fig 95:**
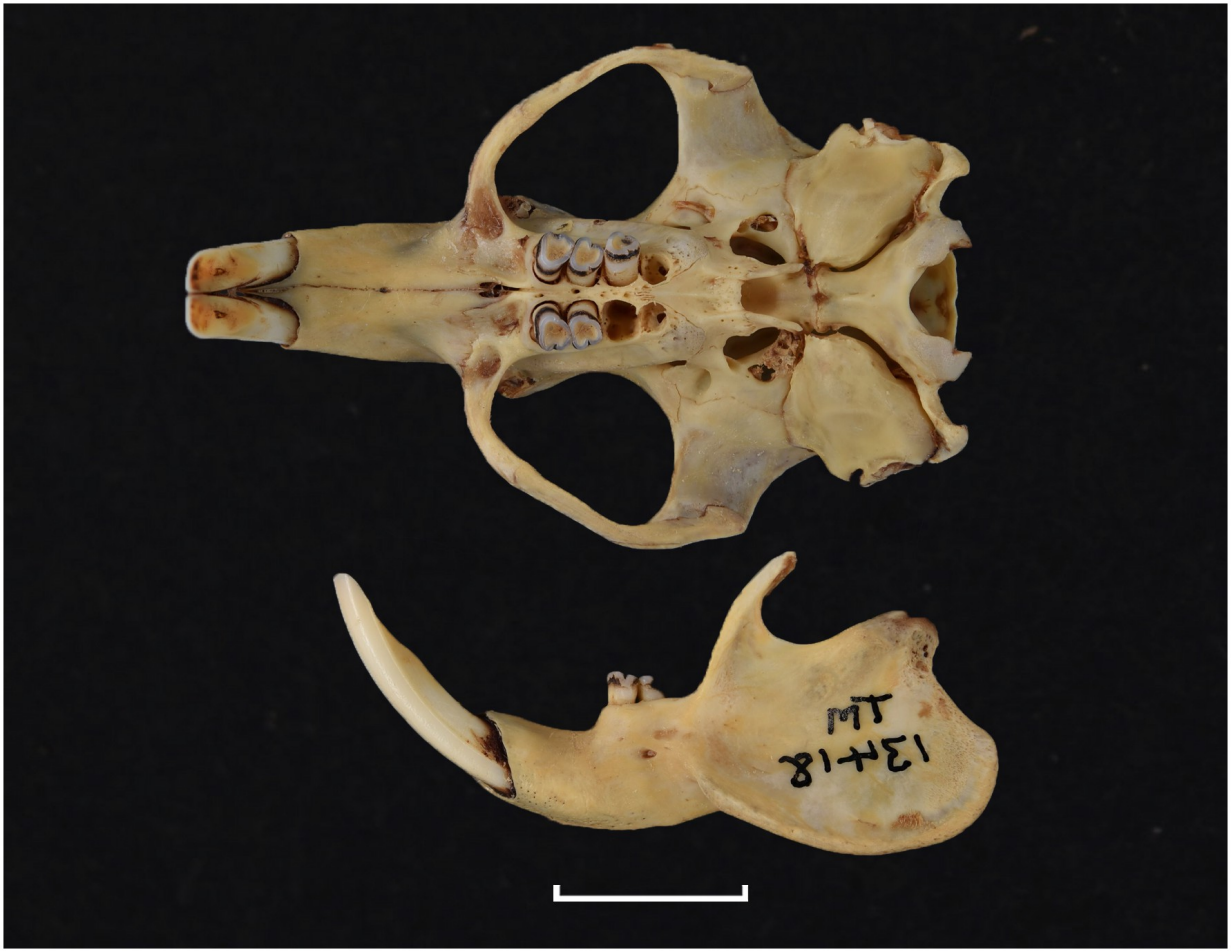
Cranium of *Cryptomys hottentotus* (DNMNH-13418), with scale bar of 1 cm. The molars M^3^ are missing.

**Fig 96:**
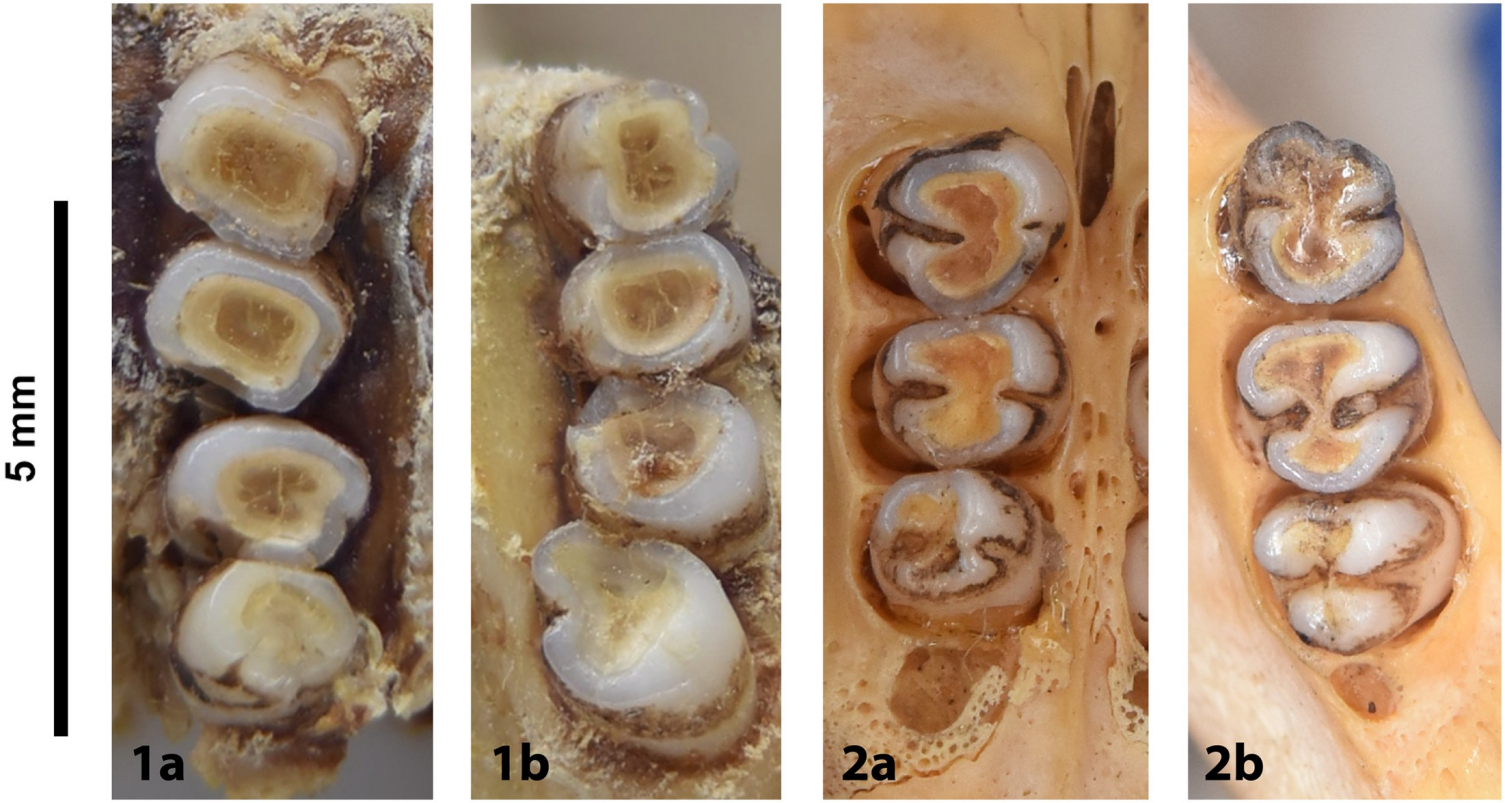
Cheekteeth of *Cryptomys*. **1)** Upper (a) and lower (b) right toothrow of an adult *C*. *hottentotus* (DNMNH-13033); **2)** Upper (a) and lower (b) right toothrow of a juvenile *C*. *hottentotus* (DNMNH-3418), with molars M^3^/M_3_ not erupted yet.

**Fig 97:**
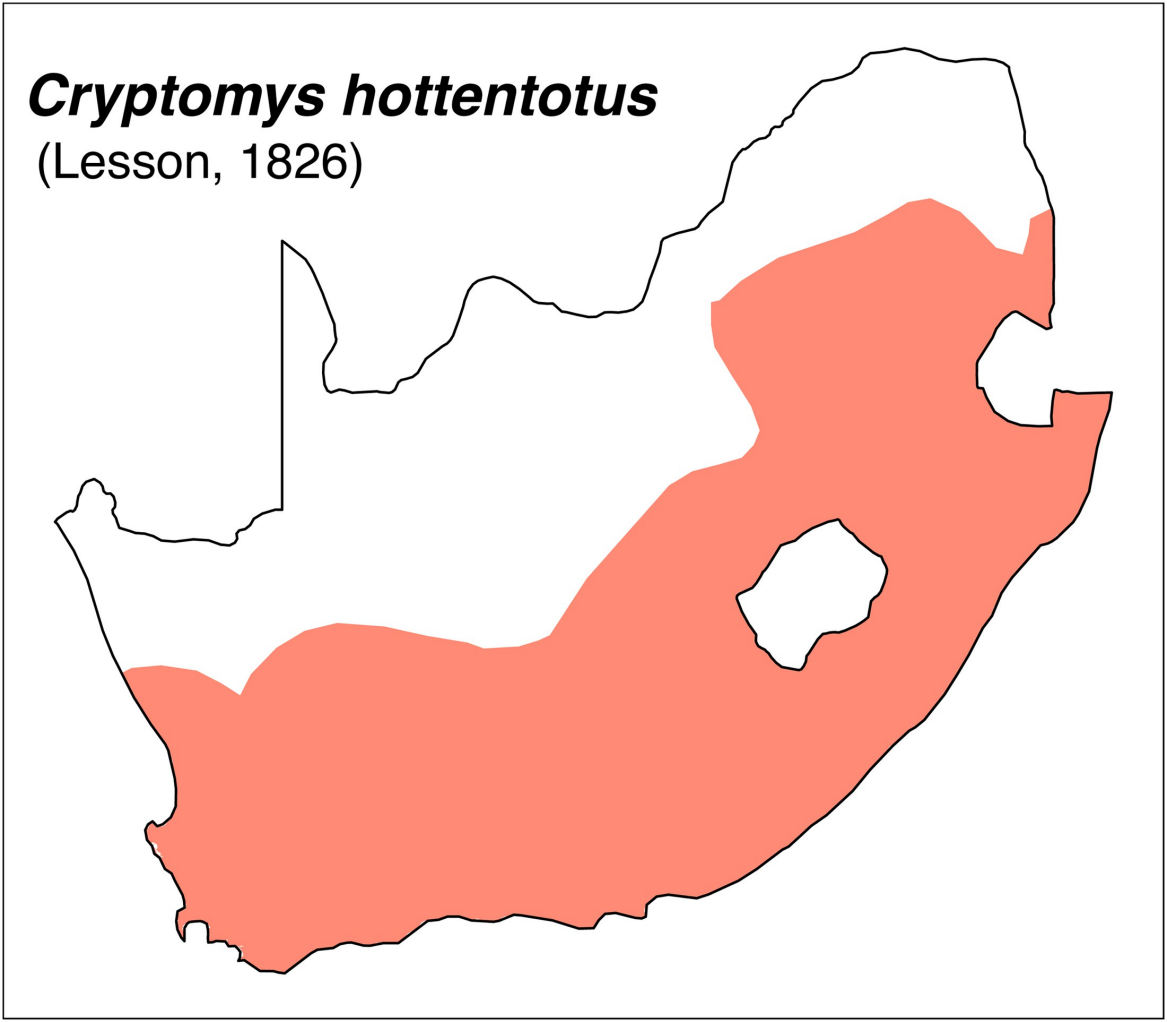
Distribution map.

Figs 95–97; Table 35

**Table 35:** Dental measurements (in mm) for *Cryptomys hottentotus*, sexes combined.

	Mean	Min	Max	n
LLTR	6.1	5.1	6.8	11
WM_1_	1.9	1.7	2.2	11
LUTR	6.3	5.2	6.6	11
WM^1^	2.1	1.9	2.3	11

Dental formula is 1-0-1-3:1-0-1-3. Alveolar formula varies with age of specimen.

#### Upper jaw

*Cryptomys* and *Fukomys* appear to be indistinguishable on morphological grounds. Upper incisors are robust, ungrooved and proodont. Anterior palatal foramina are very short, ending before molar M^1^. Cheekteeth are simple, rounded and uncusped, but young animal display re-entrant folds. The skull is the smallest found in the Bathyergidae.

#### Lower jaw

Lower incisors are smooth on their anterior surfaces. Their width is less than 2 mm, even for older specimens (in *Bathyergus*, the width of the incisor is superior to 2 mm before the M_3_ is erupted). Lower cheekteeth are similar to upper cheekteeth: simple, rounded and uncusped, although young animals display re-entrant folds. The coronoid process of the hystricognathous mandible is high and projects backwards. The mental foramen is positioned posteriorly to the diastema, aligned with the posterior border of the M_1_.

#### Systematic notes and South African fossil record

The species delimitation of *Cryptomys* has changed multiple times over last decades [75]. This genus formerly encompassed all the species currently placed within *Fukomys*. A single species is currently recognised in South Africa:

- *Cryptomys hottentotus* (Lesson, 1826)

Additional fossil species have been described in South Africa:

- *†Cryptomys broomi* Denys, 1998 from Langebaanweg
- †*Cryptomys robertsi* Broom, 1937

Fossils of *Cryptomys* are known from the Early Pliocene and have been identified in many Quaternary deposits. However, most of this material was identified before the recognition of *Fukomys* as a distinct genus. It is therefore likely that some of these remains may rather be attributed to this latter genus.

Genus ***Fukomys*** Kock et al., 2006 (Mole-rats)

**Fig 98:**
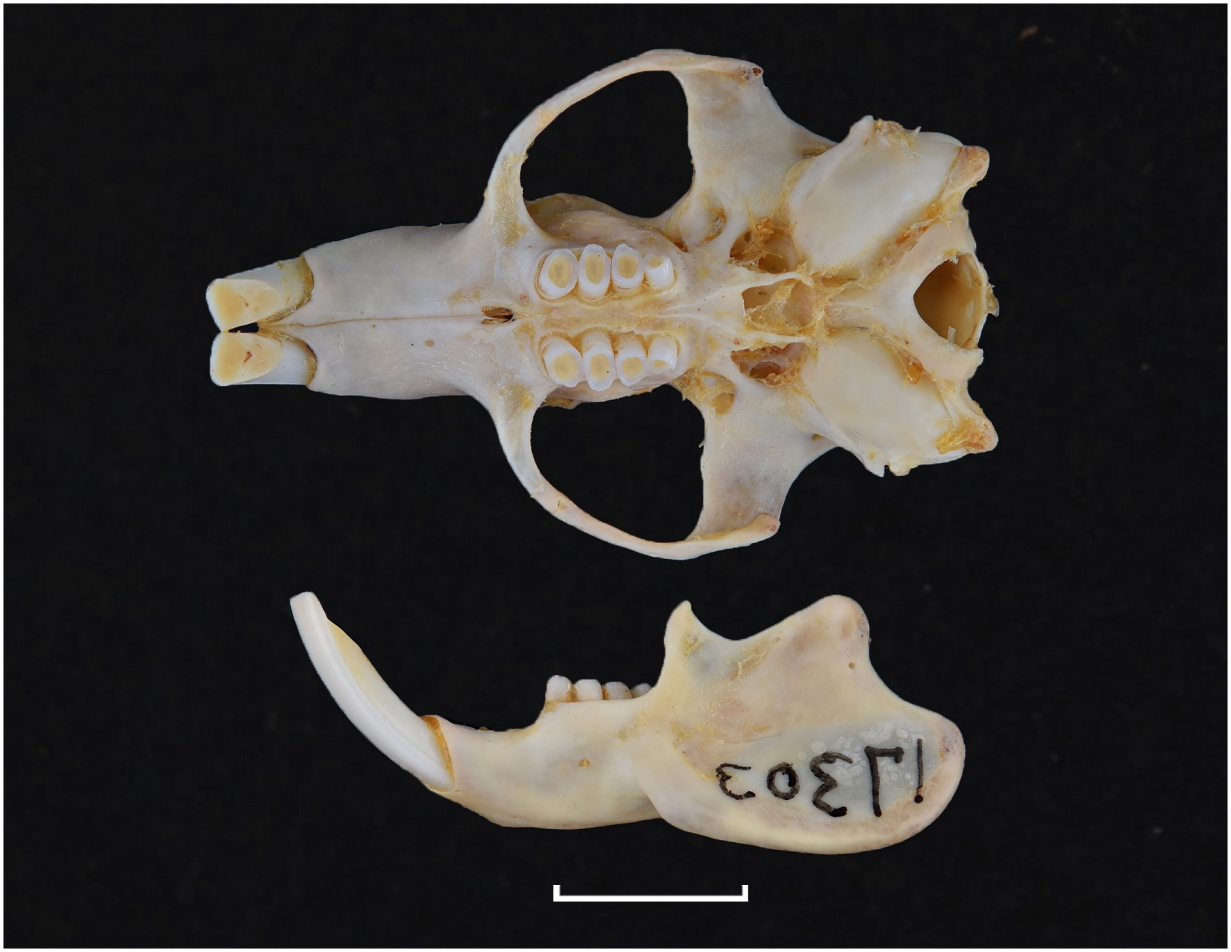
Cranium of *Fukomys damarensis* (DNMNH-17303), with scale bar of 1 cm.

**Fig 99:**
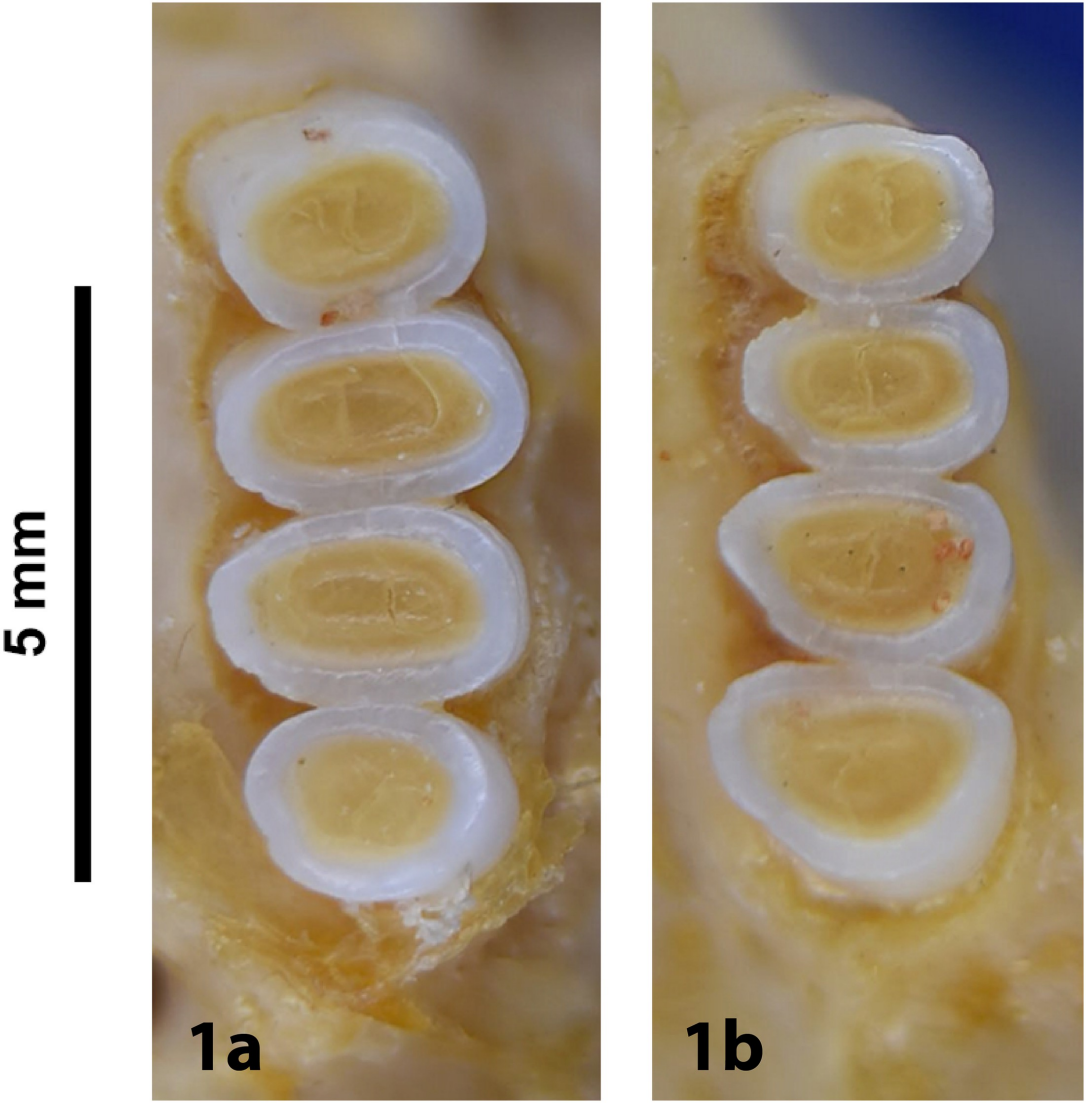
Upper (a) and lower (b) right toothrow of an adult *F*. *damarensis* (DNMNH-17304).

**Fig 100:**
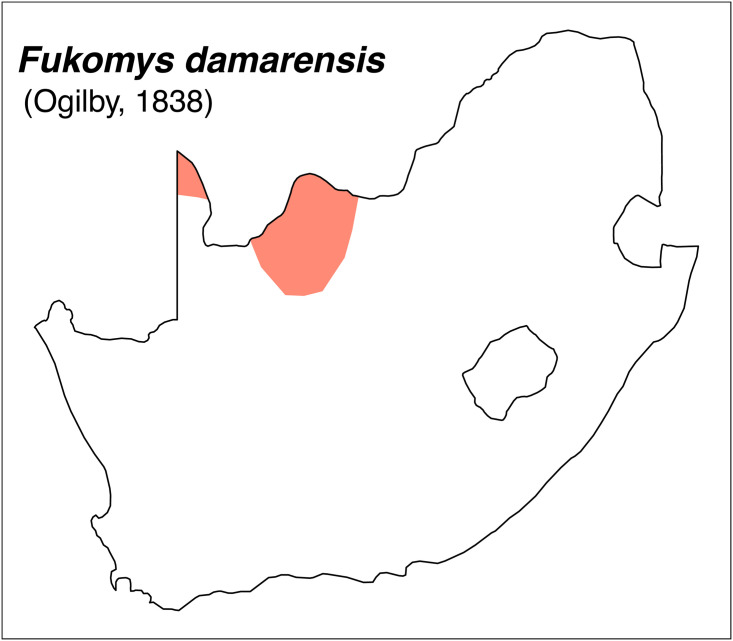
Distribution map.

Figs 98–100; Table 36

**Table 36:** Dental measurements (in mm) for *Fukomys damarensis*, sexes combined.

	Mean	Min	Max	n
LLTR	6.4	6.2	6.9	12
WM_1_	2.1	2.0	2.3	12
LUTR	6.8	6.6	7.7	11
WM^1^	2.4	2.3	2.7	12

Dental formula is 1-0-1-3:1-0-1-3. Alveolar formula varies with age of specimen.

#### Upper jaw

*Cryptomys* and *Fukomys* appear to be indistinguishable on morphological grounds. See *Cryptomys* for anatomical description.

#### Lower jaw

See *Cryptomys*.

#### Systematic notes and South African fossil record

Species of this genus were previously included in *Cryptomys* until molecular analyses revealed the existence of two well separated clades within this genus [75]. A single species is currently recognized in South Africa:

- *Fukomys damarensis* (Ogilby, 1838)

No fossil remains of *Fukomys* have been identified in South Africa, probably due to the fact that *F*. *damarensis* has only recently been erected as a separate species from *C*. *hottentotus*.

Genus ***Georychus*** Illiger, 1811 (Cape Mole-rats)

**Fig 101:**
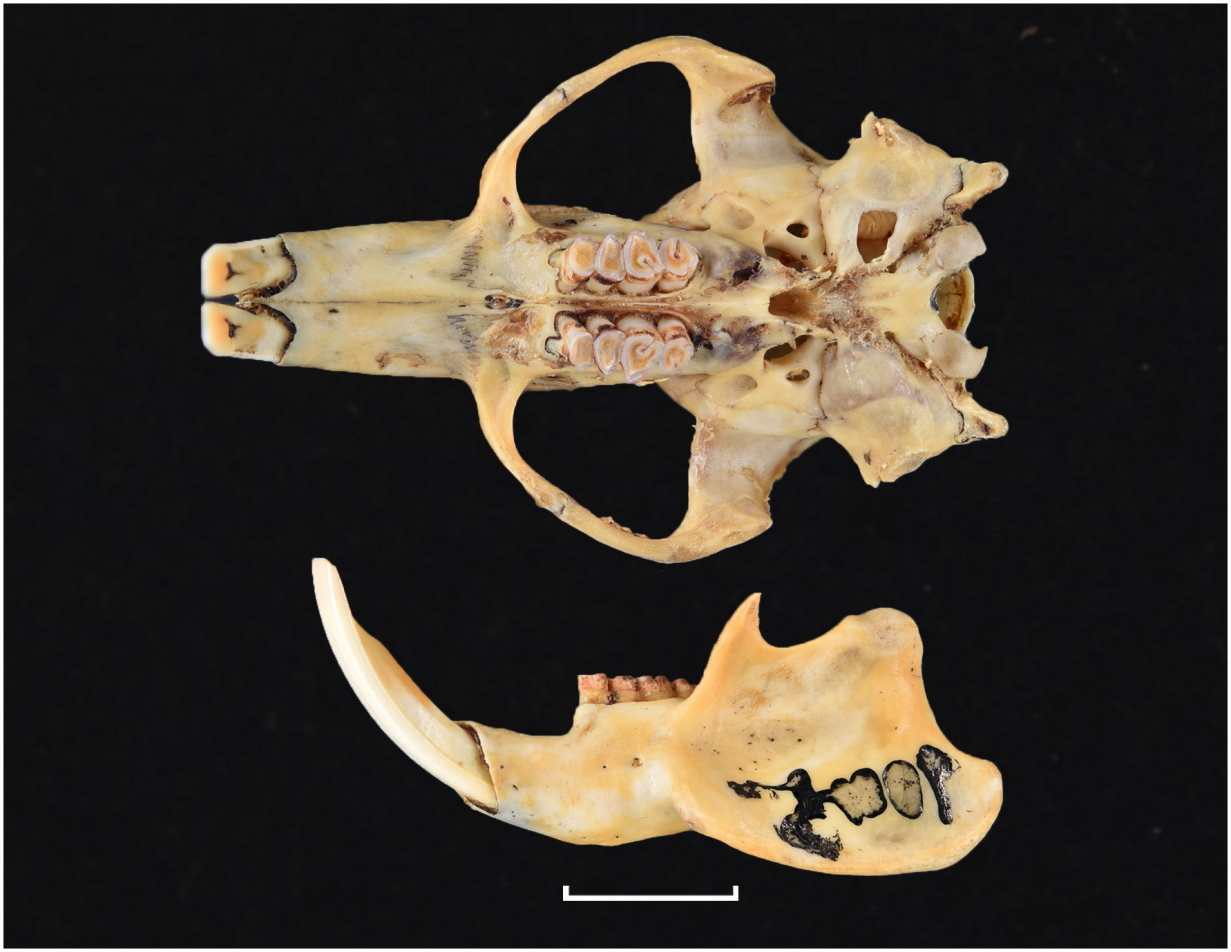
Cranium of *Georychus capensis* (DNMNH-9145), with scale bar of 1 cm.

**Fig 102:**
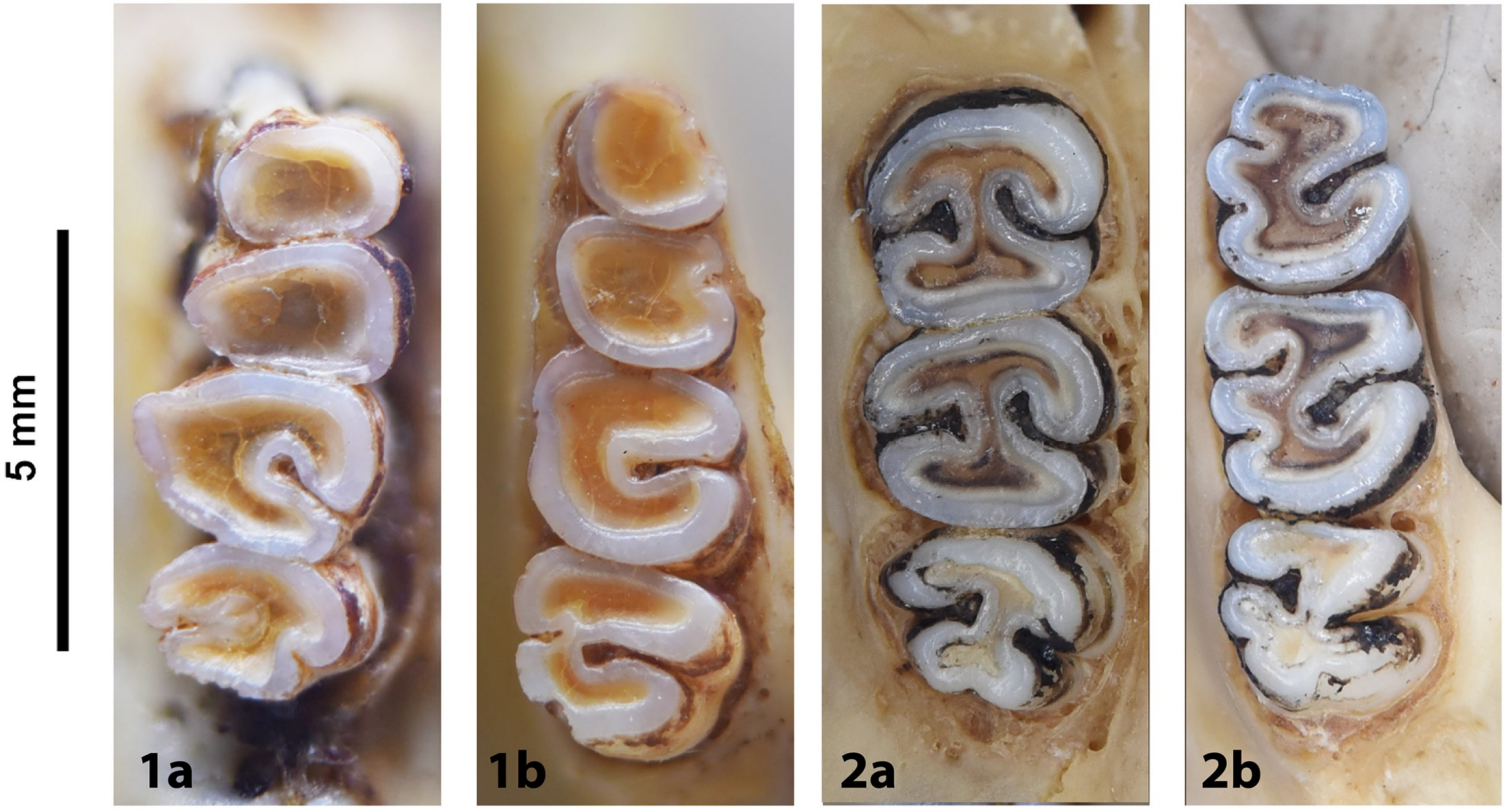
Cheekteeth of *Georychus*. 1) Upper (a) and lower (b) right toothrow of an adult *G*. *capensis* (DNNH-9145); 2) Upper (a) and lower (b) right toothrow of a juvenile *G*. *capensis* (DNMNH-4159), with molars M^3^/M_3_ not erupted yet.

**Fig 103:**
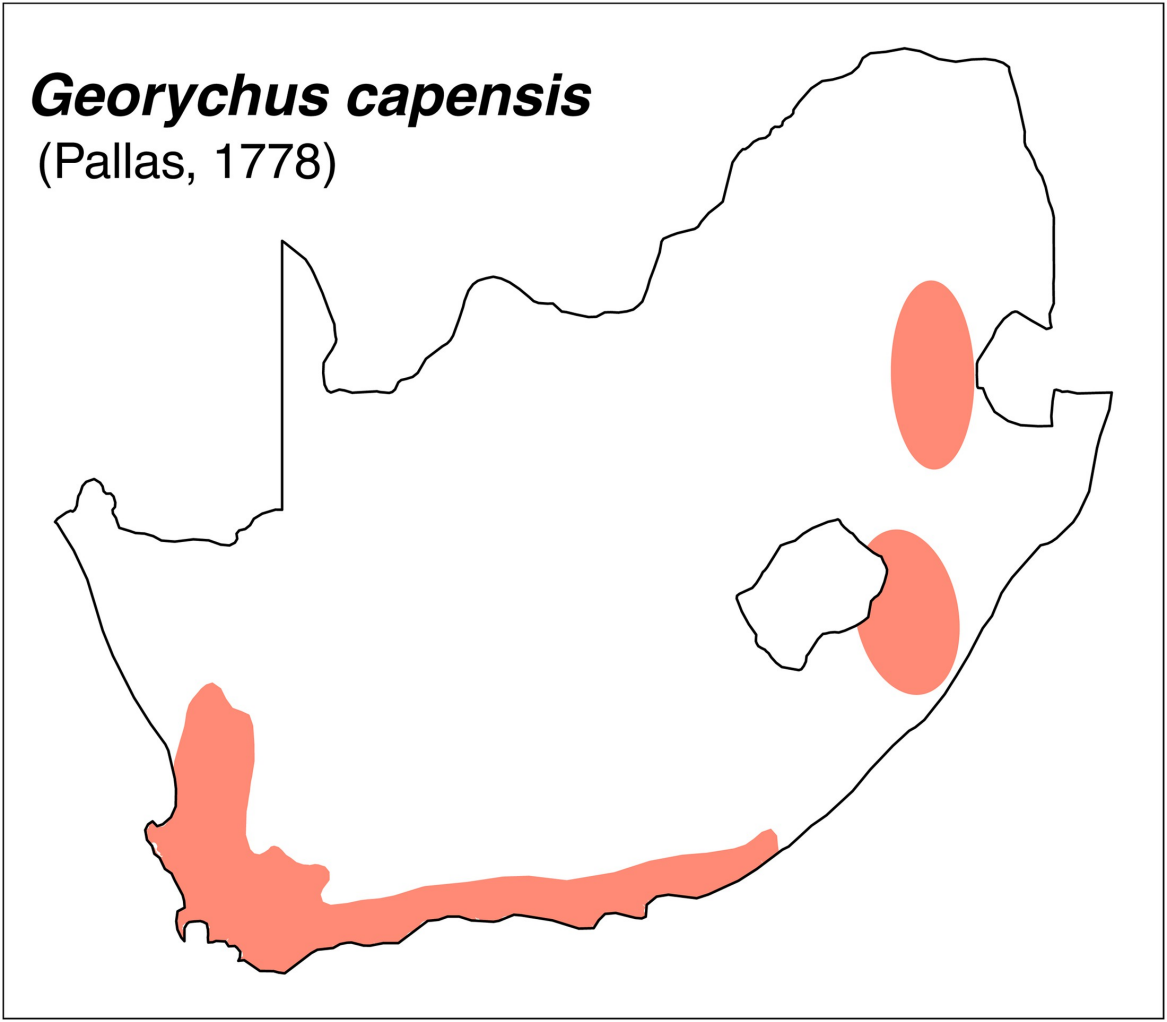
Distribution map.

Figs 101–103; Table 37

**Table 37:** Dental measurements (in mm) for *Georychus capensis*, sexes combined.

	Mean	Min	Max	n
LLTR	7.9	7.1	8.6	10
WM_1_	2.4	2.0	2.9	10
LUTR	7.7	6.7	8.3	12
WM^1^	2.7	2.3	3.1	12

Dental formula is 1-0-1-3:1-0-1-3. Alveolar formula varies with specimen age.

#### Upper jaw

Incisors are robust, ungrooved and proodont. Anterior palatal foramina are very short, ending before molar M^1^. Molars have single inner and outer folds of enamel jutting into the dentine. Folds on M^2^ persist with age, on M^3^ also to a lesser extent, and folds on P^4^ and M^1^ tend to disappear when teeth are worn. The skull is of intermediate size, being larger than *Cryptomys/Fukomys* but smaller than *Bathyergus*; it is robust and has a narrow rostrum.

#### Lower jaw

Lower incisors are smooth. Their width is more than 2.3 mm, even for young specimens. Lower molars have one outer fold that persists with age and one inner fold that tends to disappear with wear. The mental foramen is positioned posteriorly to the diastema, aligned with the posterior border of the M_1_.

#### Systematic notes and South African fossil record

This genus is monotypic:

- *Georychus capensis* (Pallas, 1778)

Fossil data suggest that this genus is present since the Late Pliocene in South Africa [50, 58].

### Pedetidae

The family Pedetidae contains only the genus *Pedetes*. It is a very large rodent, surpassed in weight only by *Hystrix* and large specimens of *Thryonomys*. Due to its size and high weight, eagle owls (*Bubo africanus*, *Bubo capensis* and *Bubo lacteus*) are the only nocturnal raptors capable of predating adult individuals [21, 76]. The nomenclature of the teeth of fossil and modern representatives of Pedetidae was detailed in Pickford & Mein [77].

Family **PEDETIDAE** Gray, 1825

Genus ***Pedetes*** Fitzinger, 1867 (Springhares)

**Fig 104:**
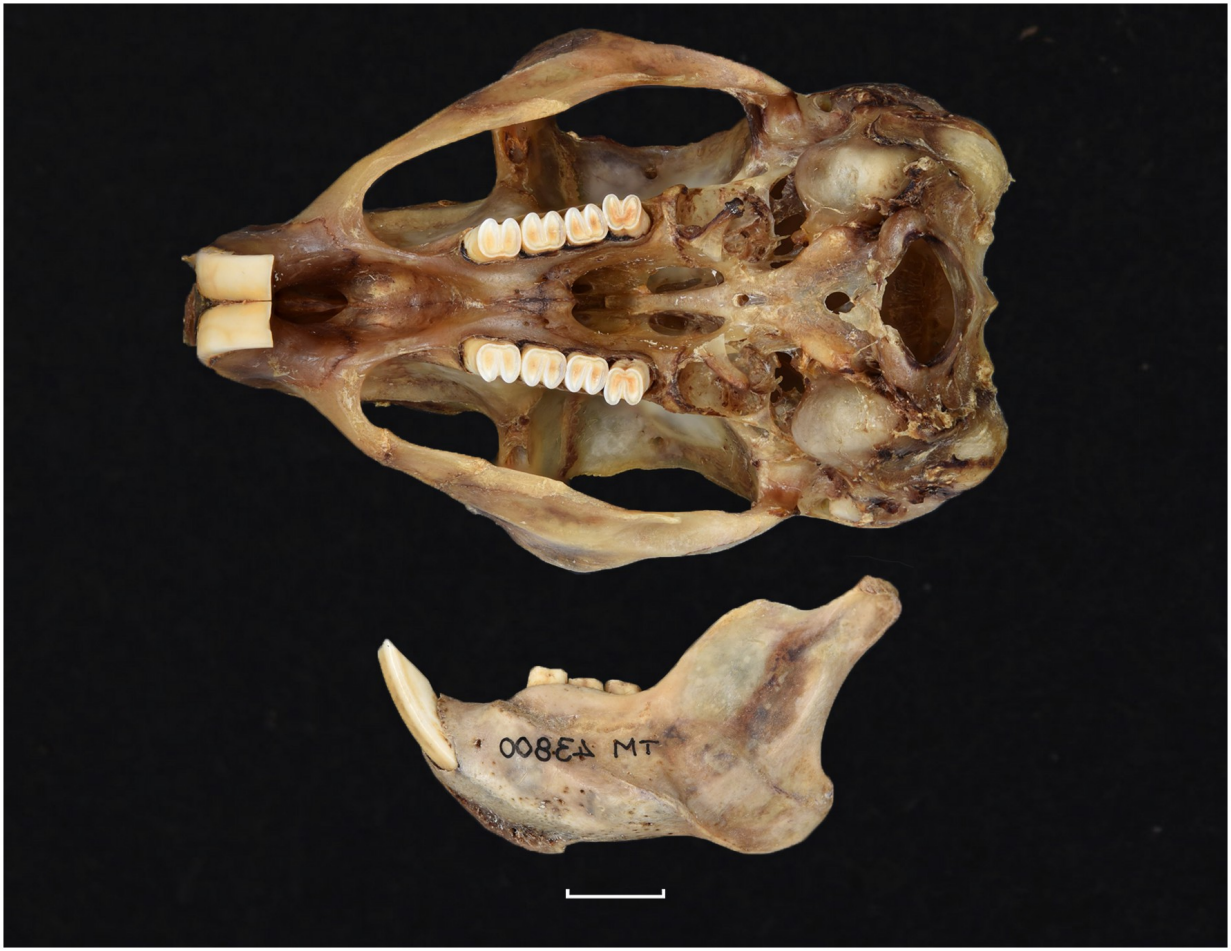
Cranium of *Pedetes capensis* (skull DNMNH-44480, mandible DNMNH-43800), with scale bar of 1 cm.

**Fig 105:**
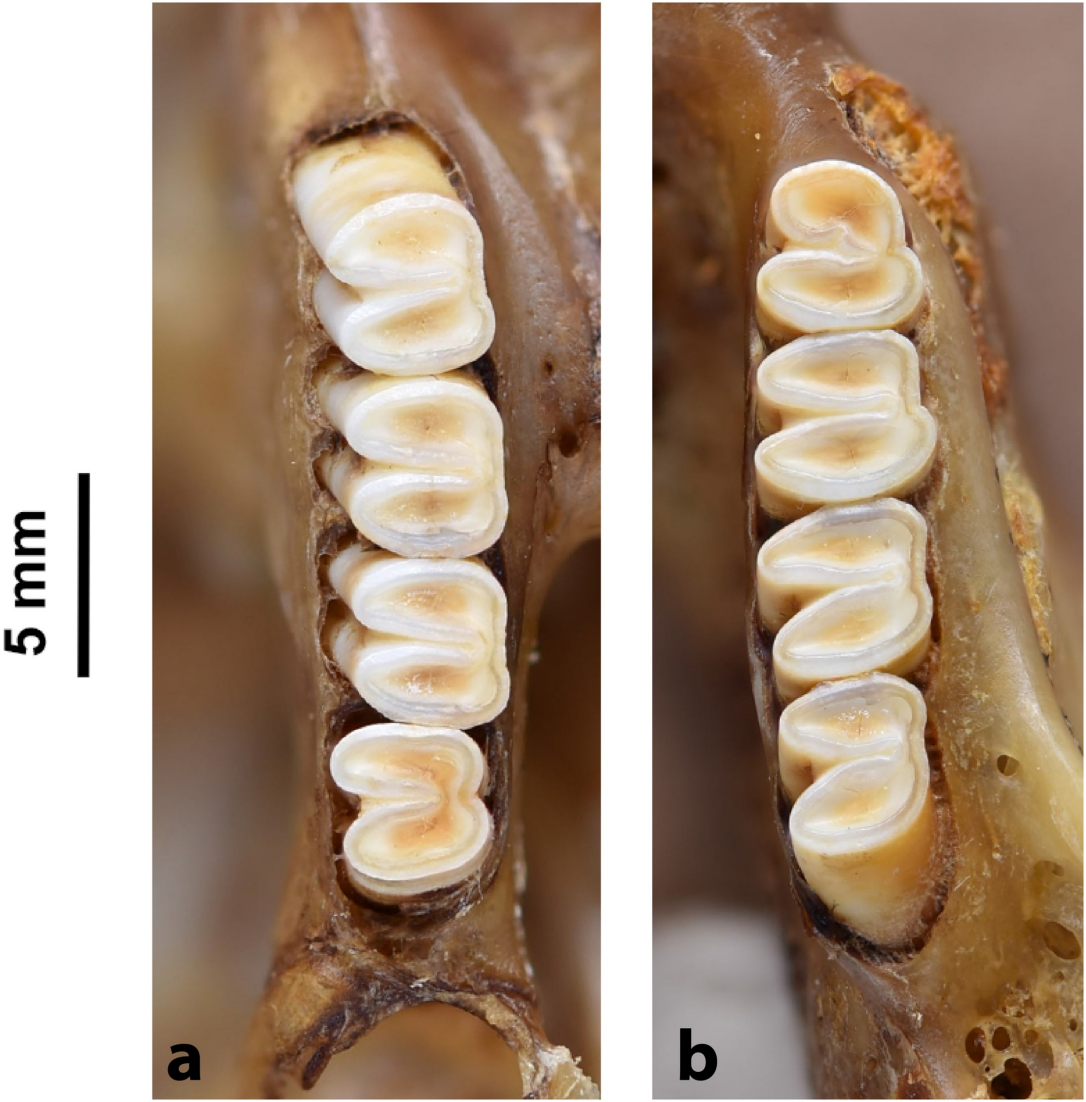
Upper (a) and lower (b) right toothrow of *P*. *capensis* (DNMNH-44480).

**Fig 106:**
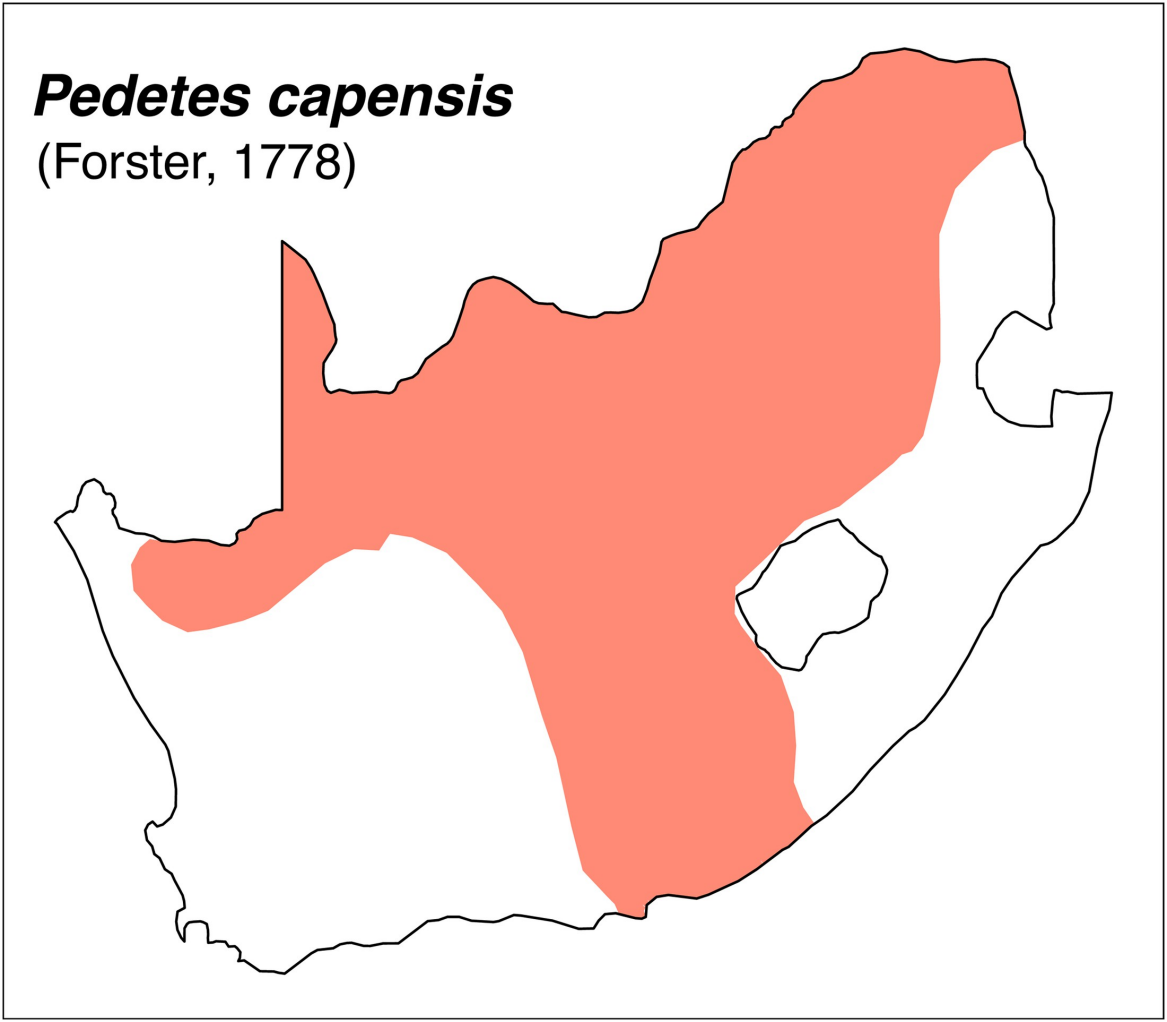
Distribution map.

Figs 104–106; Table 38

**Table 38:** Dental measurements (in mm) for *Pedetes capensis*, sexes combined.

	Mean	Min	Max	n
LLTR	18.3	16.2	19.9	16
WM_1_	4.4	3.5	5.5	16
LUTR	18.2	16.1	19.8	11
WM^1^	3.9	3.4	4.2	14

Dental formula is 1-0-1-3:1-0-1-3.

#### Upper jaw

The skull is large and robust. Upper incisors are opisthodont, thick and ungrooved. The anterior palatal foramina are short. The cheekteeth are all roughly the same size. They are hypsodont and open-rooted. The cheekteeth are comprised of two lophs, which are separated by a median transverse valley and uniting in the lingual side.

#### Lower jaw

The mandible is sciurognath. The condylar process reaches beyond the level of the angular process, which is poorly defined. The coronoid process consists of a thin ridge. Lower incisors are thick and ungrooved. Lower cheekteeth are bilophed like in upper tooth row but are uniting in the labial side.

#### Systematic notes and South African fossil record

A single species of *Pedetes* occurs in South Africa:

- *Pedetes capensis* (Forster, 1778)

Two additional fossil species have been listed:

- *†Pedetes gracilis* Broom, 1934 from the Pliocene locality of Taung
- †*Pedetes hagenstadti* Dreyer and Lyle, 1931 from the Middle Pleistocene locality of Florisbad

### Hystricidae

In South Africa, only the genus *Hystrix* occurs. It contains one modern species, *H*. *africaeaustralis*, and one fossil species, *H*. *makapanensis*. *H*. *africaeaustralis* is the largest African rodent and has long spines (quills) covering its back and flanks. Large carnivores are the most common predator. The nomenclature of the teeth of fossil and modern representatives of African *Hystrix* is detailed in Azzarà et al. [78].

Family **HYSTRICIDAE** G. Fischer, 1817

Genus ***Hystrix*** Linnaeus, 1758 (Crested Porcupines)

**Fig 107:**
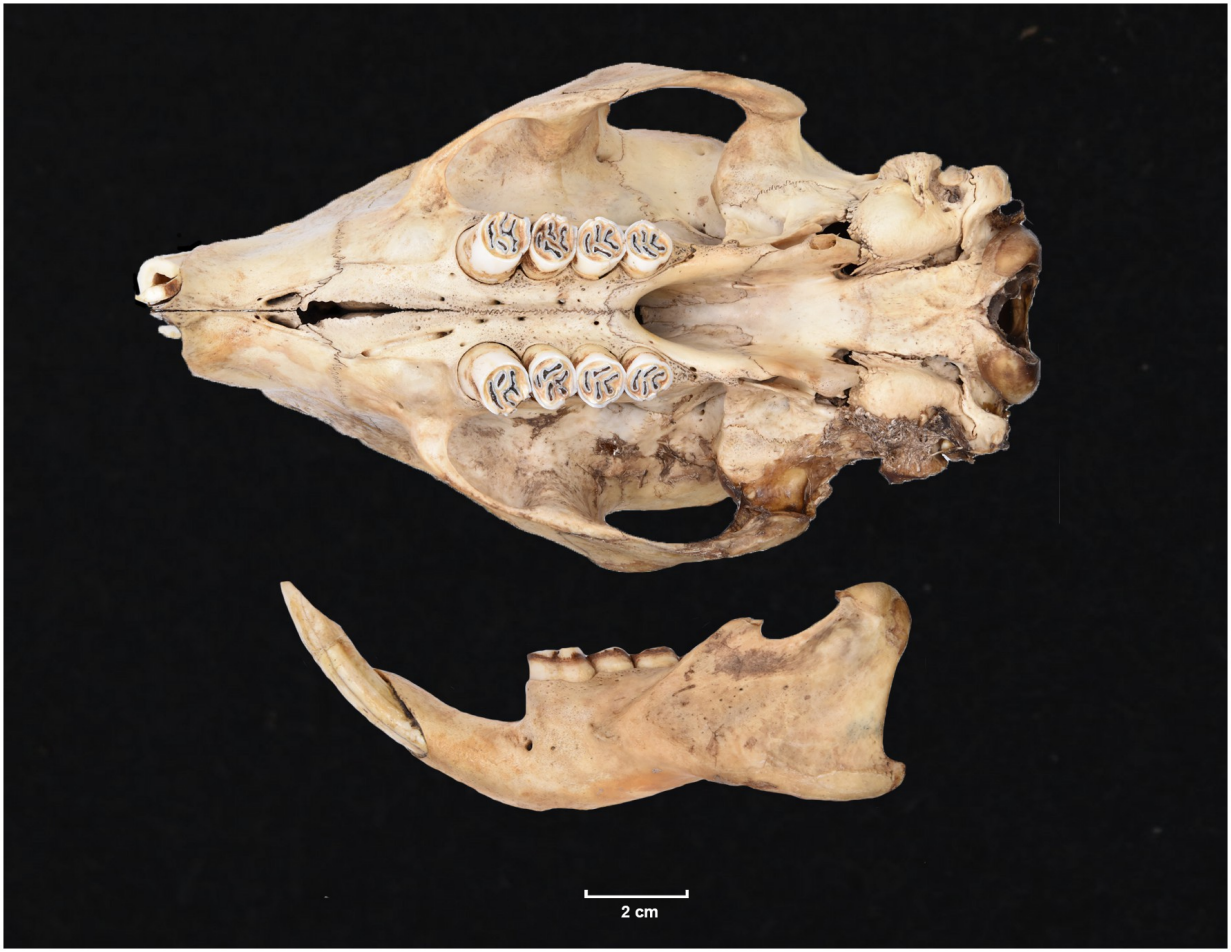
Cranium of *Hystrix africaeaustralis* (ESI BPI-4-881), with scale bar of 2 cm.

**Fig 108:**
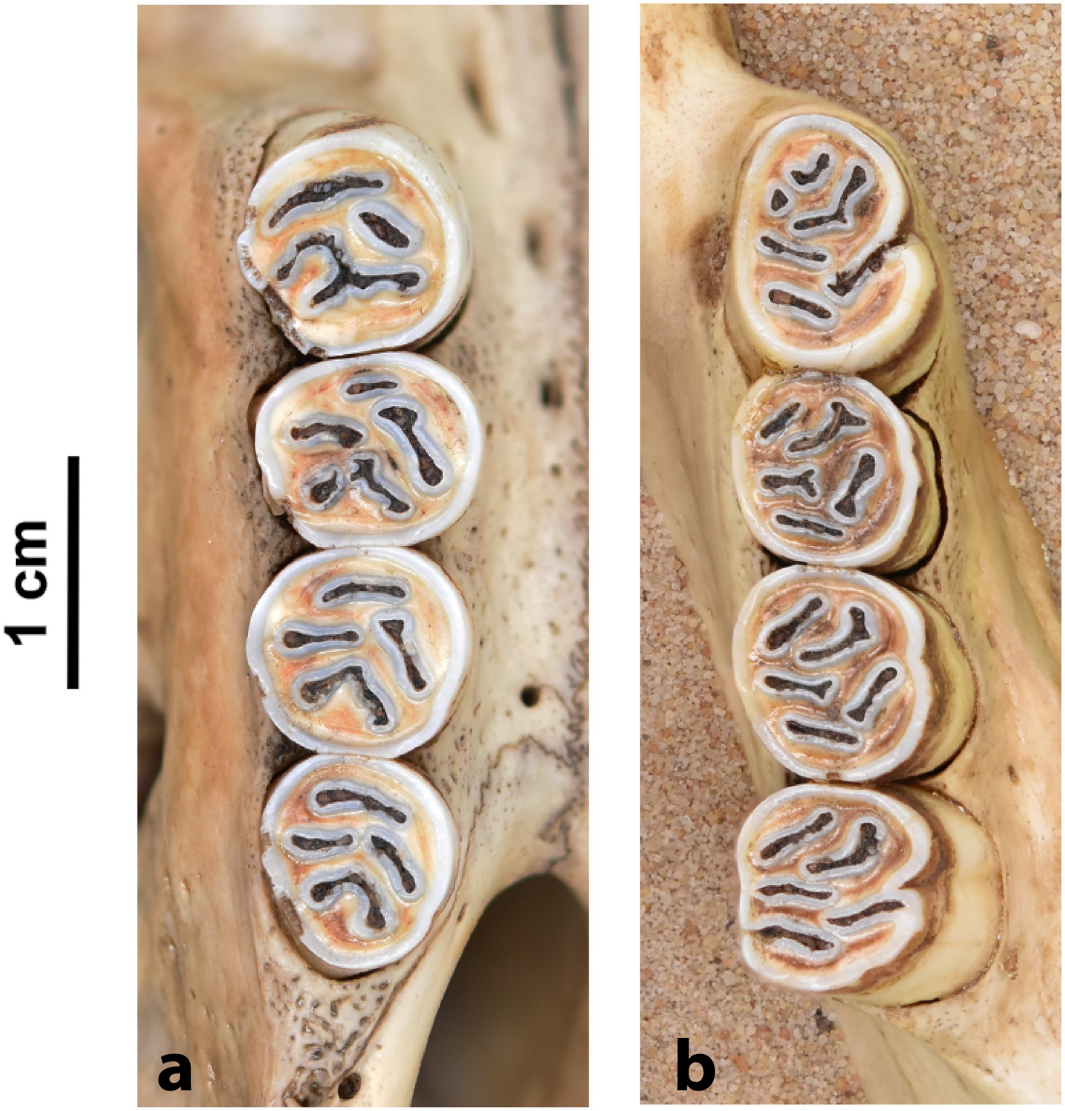
Upper (a) and lower (b) right toothrow of *H*. *africaeaustralis* (ESI BPI-4-881).

**Fig 109:**
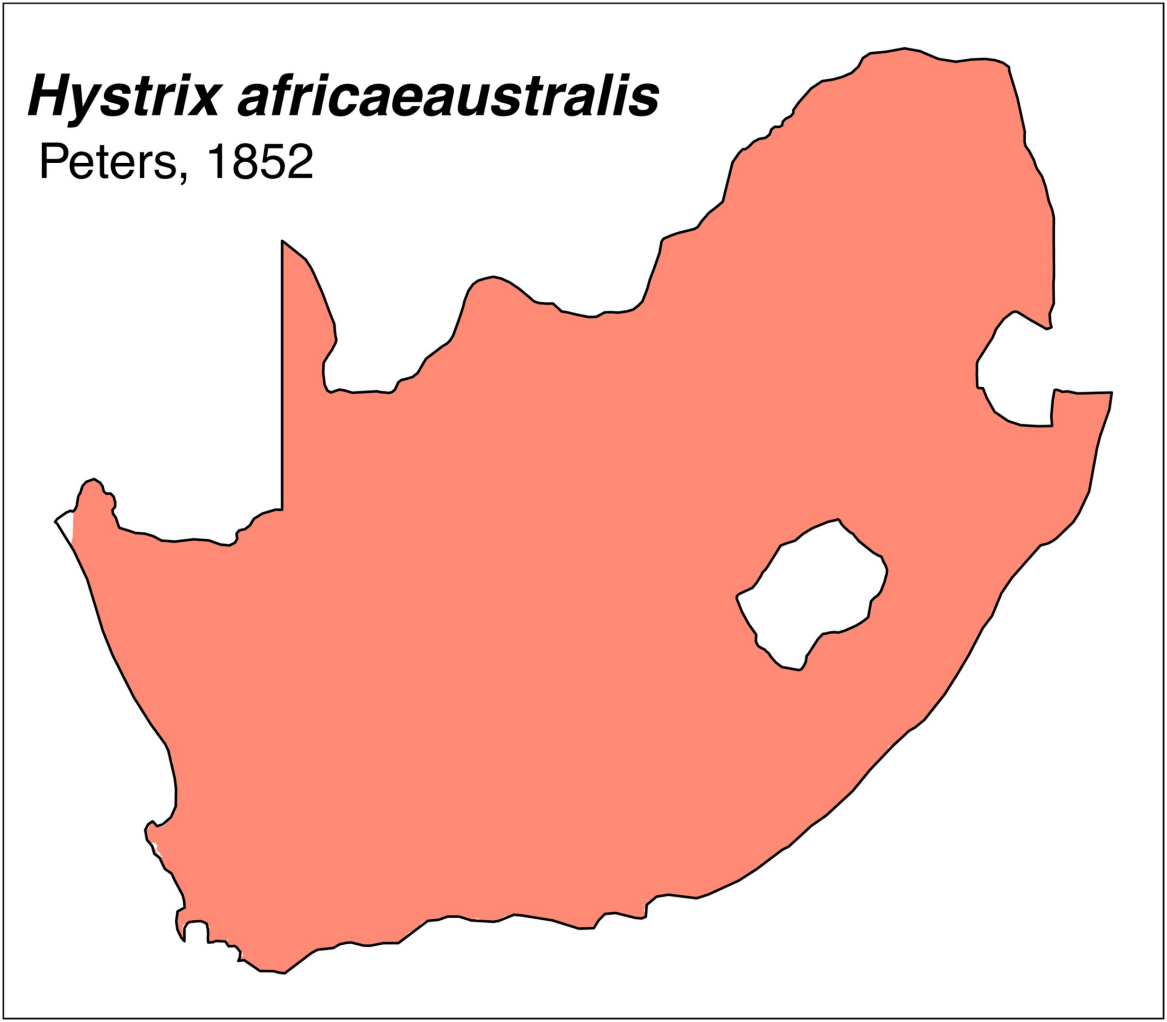
Distribution map.

Figs 107–109; Table 39

**Table 39:** Dental measurements (in mm) for *Hystrix africaeaustralis*, sexes combined.

	Mean	Min	Max	n
LLTR	36.9	33.8	39.2	7
WM_1_	8.2	7.7	8.7	7
LUTR	36.1	31.8	39.9	8
WM^1^	9.0	6.9	10.1	8

Dental formula is 1-0-1-3:1-0-1-3.

#### Upper jaw

Upper incisors are large, proodont and smooth. The anterior palatal foramina are short and located far forwards from the cheekteeth. Molars are large and tend towards hypsodonty. They display cusps related by lophs when unworn, but the crowns rapidly reduce in height with age, and wear of the tooth and cusps get individualized into small enamel islands. The teeth all have only one fold (also called *flexus*) on the lingual side and up to three folds on the labial side (this arrangement is revers in the lower teeth).

#### Lower jaw

Lower incisors are large and ungrooved. Lower teeth display the same characteristic occlusal pattern with crests of enamel, but there is one labial fold and up to three lingual folds. The mandible is hystricognath.

#### Systematic notes and South African fossil record

The following species occurs in South Africa:

- *Hystrix africaeaustralis* Peters, 1852

An additional species is listed in the South African fossil record:

- *†Hystrix makapanensis* Greenwood, 1958 found in the Pliocene Limeworks of Makapansgat and several Early Pleistocene deposits from the Sterkfontontein Valley in Gauteng Province [78].

Remains of *Hystrix* are found in many fossil deposits through the Quaternary.

### Thryonomyidae

The family Thryonomyidae includes the single genus, *Thryonomys*. It is the third largest rodent genus in South Africa, after *Hystrix* and *Pedetes*. Like the latter, eagle owls are the only nocturnal raptors susceptible of predating adults [21] but mesocarnivores can prey upon this genus.

Family **THRYONOMYIDAE** Pocock, 1922

Genus ***Thryonomys*** Fitzinger, 1867 (Cane Rats)

**Fig 110:**
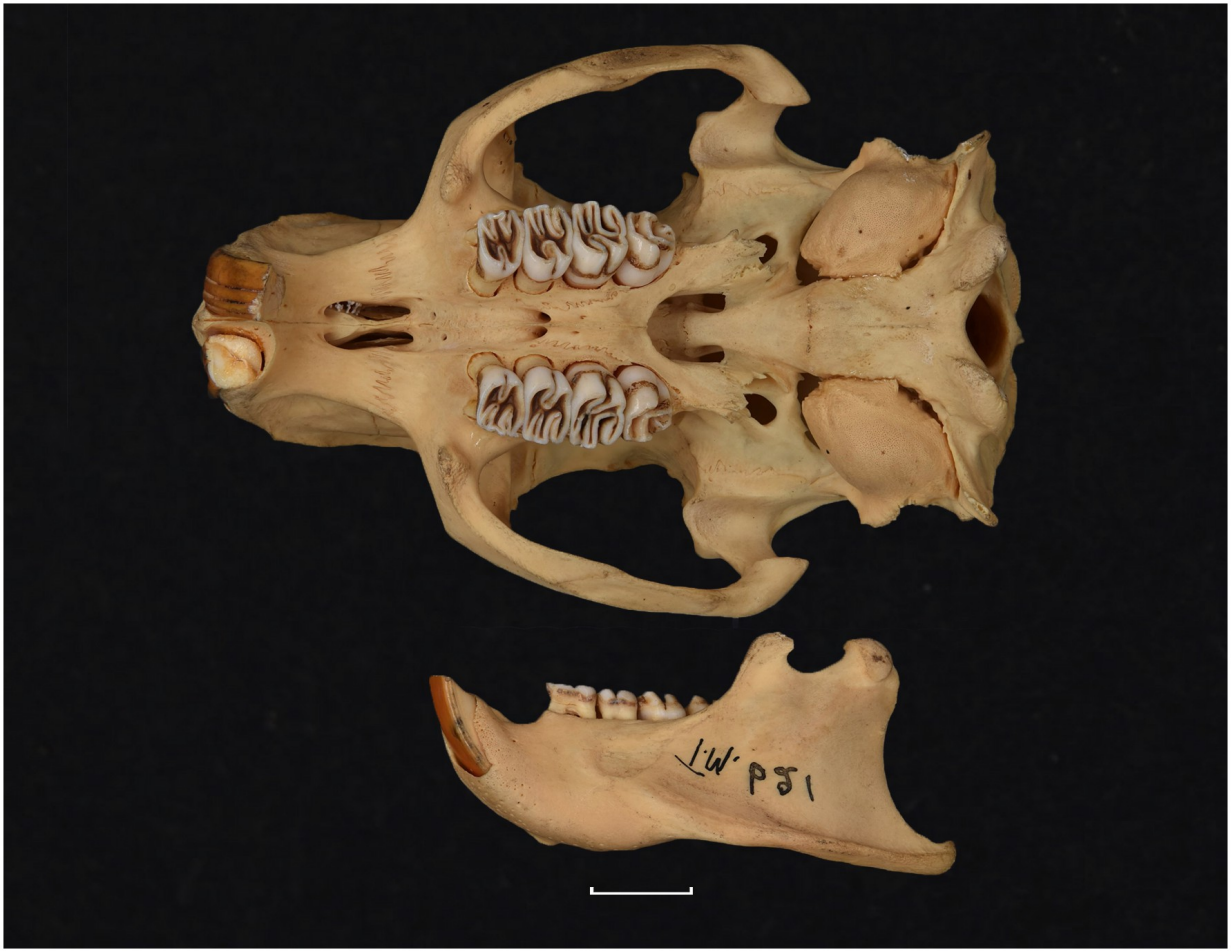
Cranium of *Thryonomys swinderianus* (DNMNH-621), with scale bar of 1 cm.

**Fig 111:**
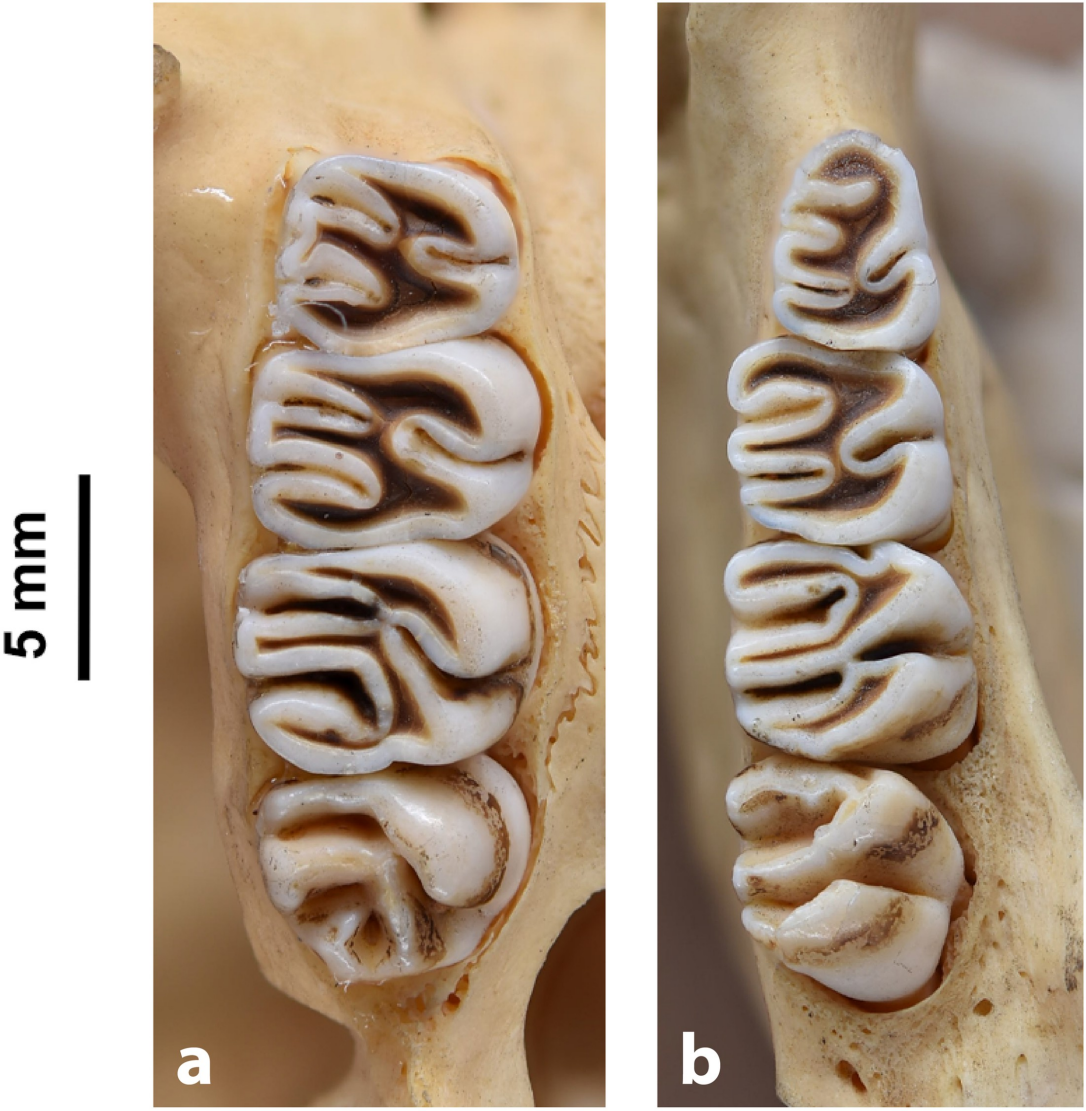
Upper (a) and lower (b) right toothrow *T*. *swinderianus* (DNMNH-621).

**Fig 112:**
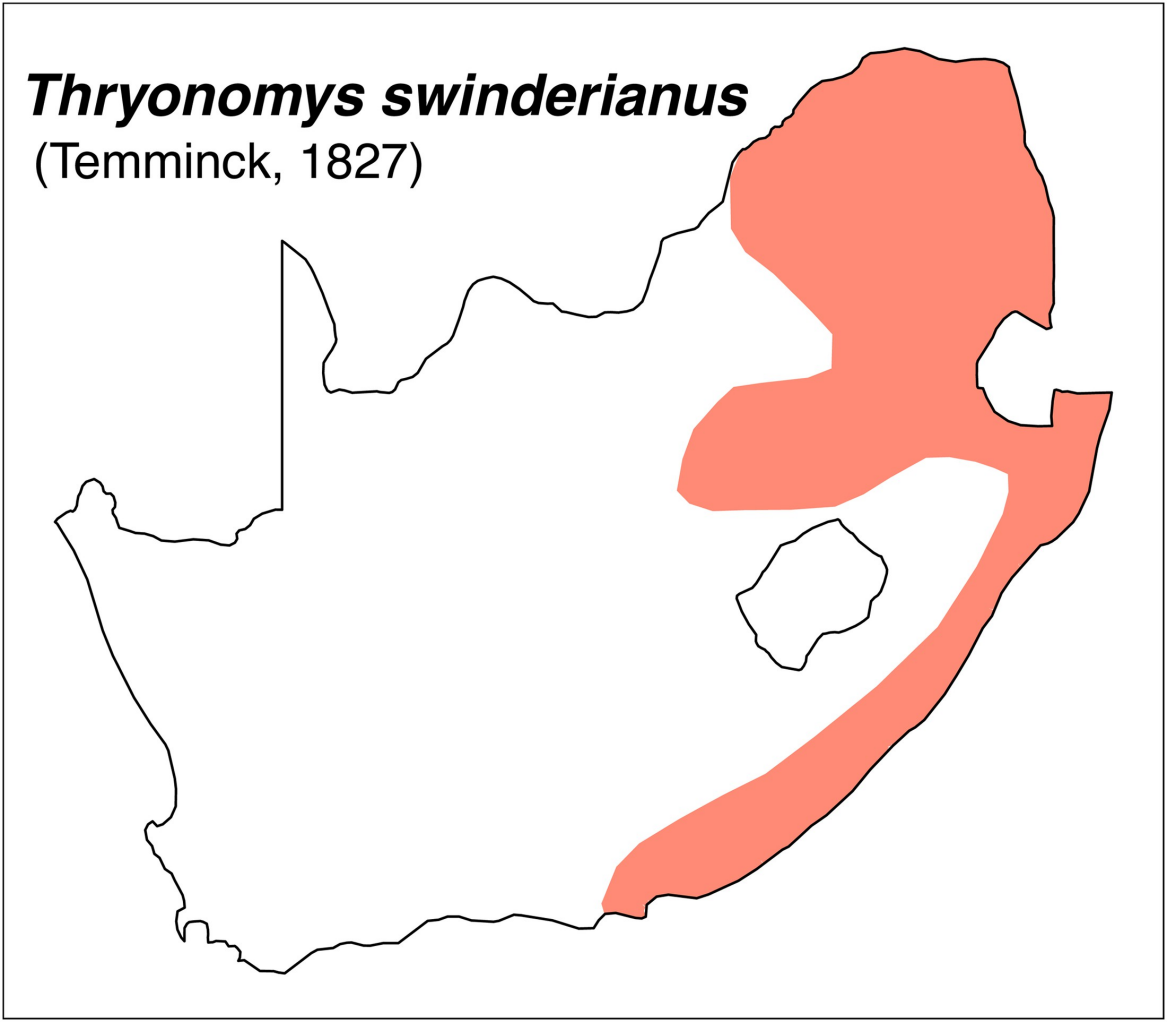
Distribution map.

Figs 110–112; Table 40

**Table 40:** Dental measurements (in mm) for *Thryonomys swinderianus*, sexes combined.

	Mean	Min	Max	n
LLTR	22.0	17.8	23.7	14
WM_1_	5.7	4.4	8.1	26
LUTR	18.8	15.1	20.6	15
WM^1^	7.0	5.3	9.3	15

Dental formula is 1-0-1-3:1-0-1-3.

#### Upper jaw

Upper incisors are broad, opisthodont, and have three grooves on the inner side. The skull is stickily built with a short rostrum. The anterior palatal foramina reach the M^1^. The cheekteeth have three to four characteristic enamel infoldings, which are single in the lingual side and double in the labial side. The premolar P^4^ has four lophs, while the molars M^1^-M^3^ have only three. The M^2^ is often the largest tooth in the row, and the M^3^ erupts late in the life on the individual.

#### Lower jaw

Lower incisors are ungrooved. The cheekteeth have two outer and one inner enamel infoldings (it is reversed in the maxilla). As for the lower teeth, there are four lophs in P^4^ and three in the molars M^1^-M^3^. The mandible is robust, and the well-developed angular process projects far backwards, and is hystricognathous.

#### Systematic notes and South African fossil record

In South Africa, a single species occurs:

- *Thryonomys swinderianus* (Thomas, 1894).

Its remains are scanty in the Quaternary fossil record, and it is found for the first time during the Late Pleistocene from Umhlatuzana [79] and Sibudu [80] caves.

### Petromuridae

The family Petromuridae contains only a single genus, *Petromus*. These medium-sized rodents are squirrel-like in appearance, and their present-day distribution is restricted to the west coast of Southern Africa. They are associated with rocky habitats, occupying rock crevices in boulders, canyons and mountain slopes [21]. Little is known about its predators, but remains were identified within pellet material produced by *Bubo africanus* [81].

Family **PETROMURIDAE** Tullberg, 1899

Genus ***Petromus*** A. Smith, 1831 (Nokis or Dassie Rats)

**Fig 113:**
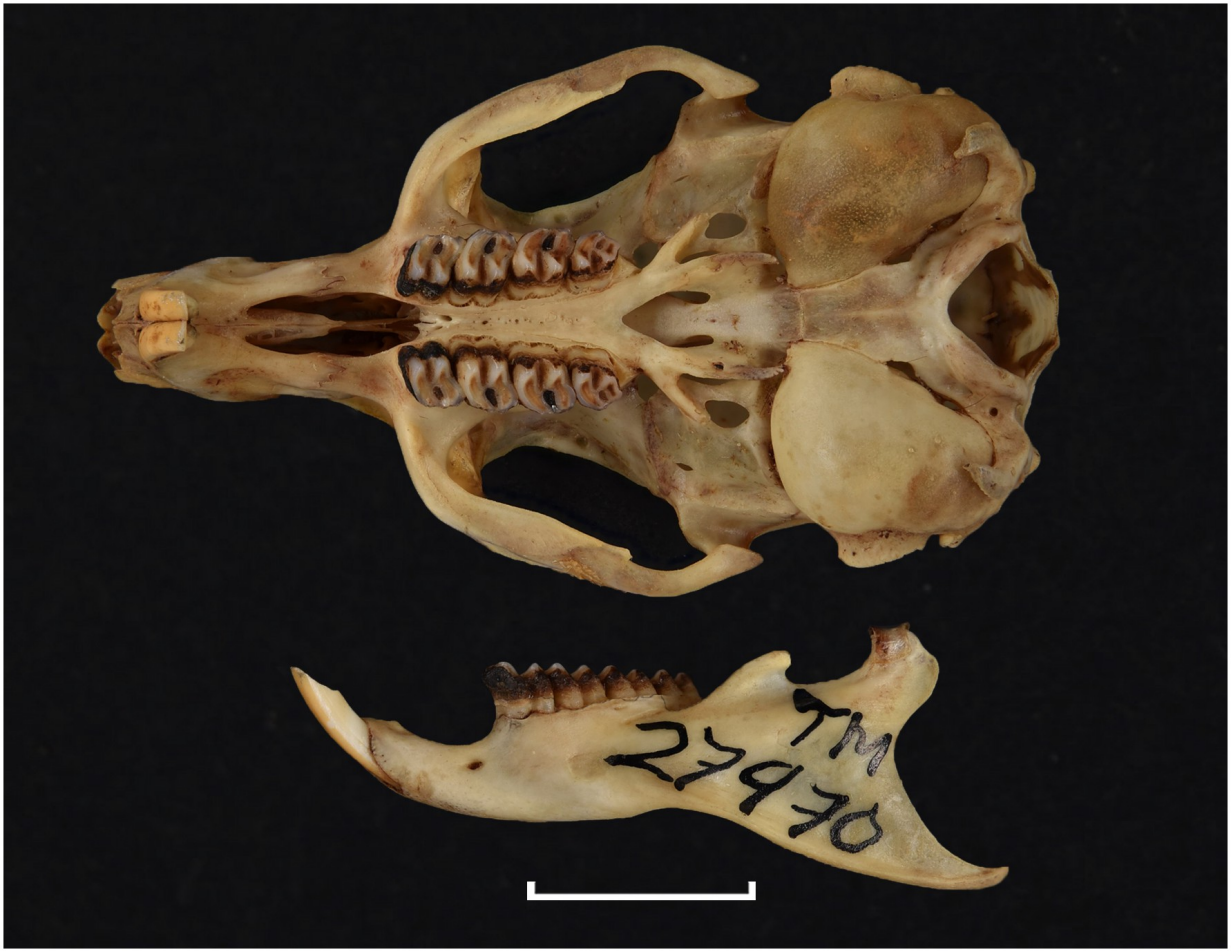
Cranium of *Petromus typicus* (DNMNH-27970), with scale bar of 1 cm.

**Fig 114:**
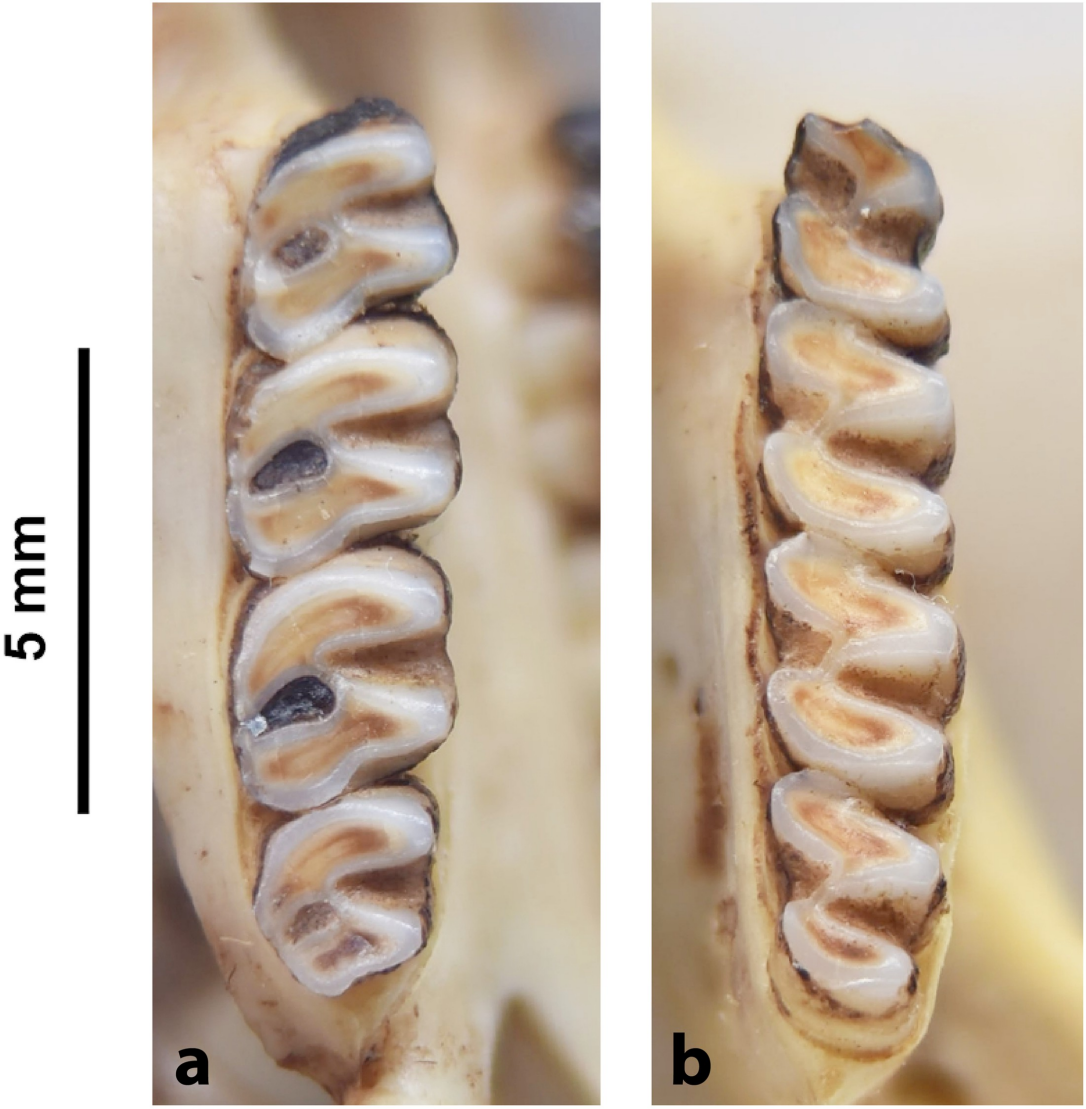
Upper (a) and lower (b) right toothrow of *P*. *typicus* (DNMNH-27970).

**Fig 115:**
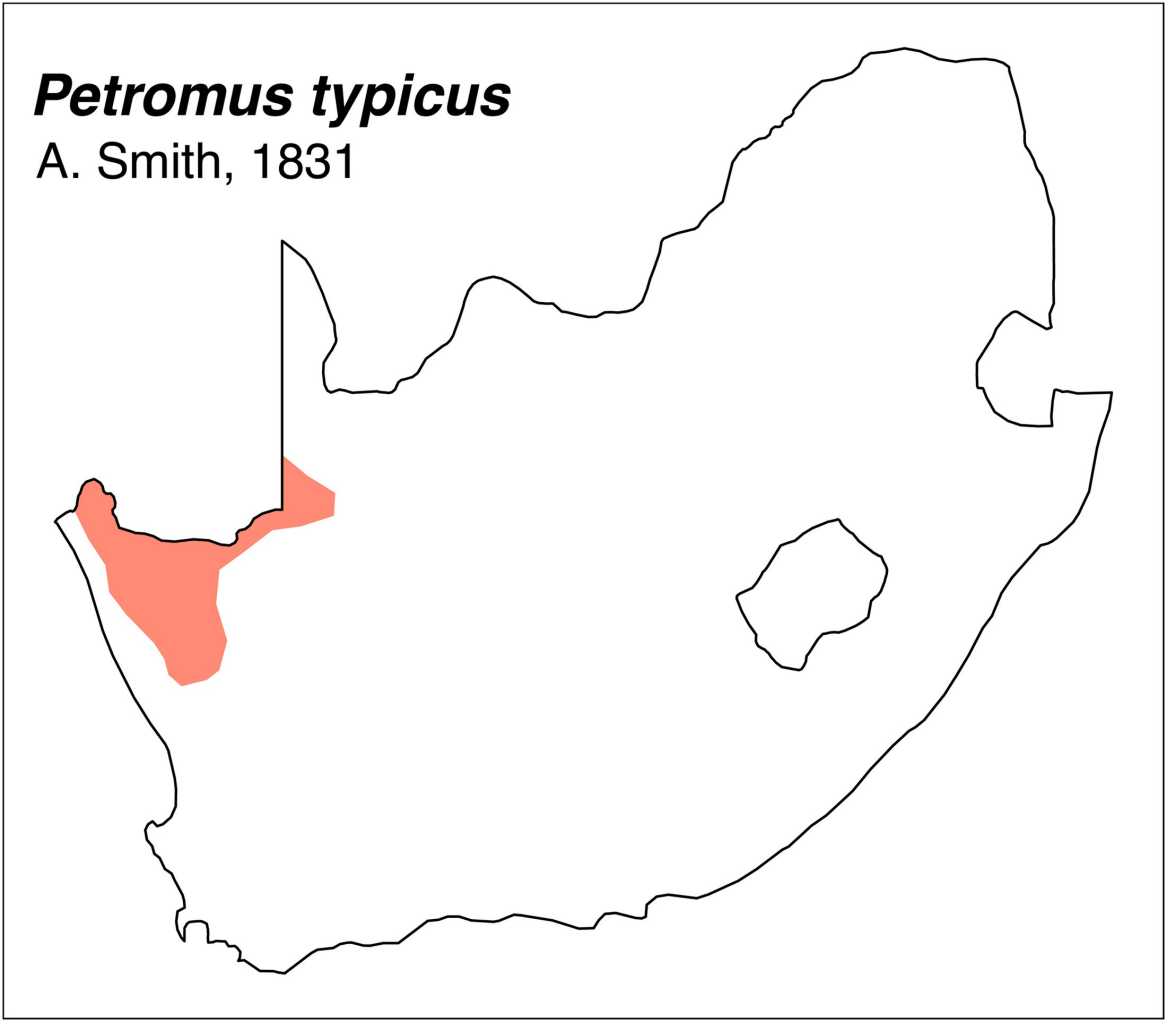
Distribution map.

Figs 113–115; Table 41

**Table 41:** Dental measurements (in mm) for *Petromus typicus*, sexes combined.

	Mean	Min	Max	n
LLTR	10.5	9.5	11.0	7
WM_1_	2.4	2.2	2.7	7
LUTR	9.9	9.5	10.8	7
WM^1^	2.8	2.6	3.2	7

Dental formula is 1-0-1-3:1-0-1-3.

#### Upper jaw

The skull is dorsoventrally flat with inflated bulla. Upper incisors are plain and opisthodont. The palate is narrow, about equal to the width of the P^4^ in its anterior part. The anterior palatal foramina are long and reach the P^4^. The four cheekteeth have roughly the same size. They are hypsodont with deep lingual enamel infoldings.

#### Lower jaw

Lower incisors are ungrooved. There are four cheekteeth, which are hypsodont with deep buccal enamel infoldings. The mandible is very long and vertically compressed with a sharp angular process, showing hystricognathy.

#### Systematic notes and South African fossil record

The genus is monotypic:

- *Petromus typicus* A. Smith, 1831

Two additional fossil species have been described in the South African fossil record:

- *†Petromus antiquus* Sénégas, 2004 from the Early Pliocene site of Waypoint 160
- *†Petromus minor* Broom, 1939 from the Late Pliocene site of Taung

### Gliridae

Only one genus of Gliridae occur in Southern Africa, the African dormouse *Graphiurus*. Species of *Graphiurus* are small-sized, squirrel-like rodents with good climbing abilities. They are predominantly arboreal, or are associated with boulders and rocky outcrops [22]. They are preyed upon by various predators, including owls.

Family **GLIRIDAE** Thomas, 1897

Subfamily **GRAPHIURINAE** Winge, 1887

Genus ***Graphiurus*** Smuts, 1832 (Dormice)

**Fig 116:**
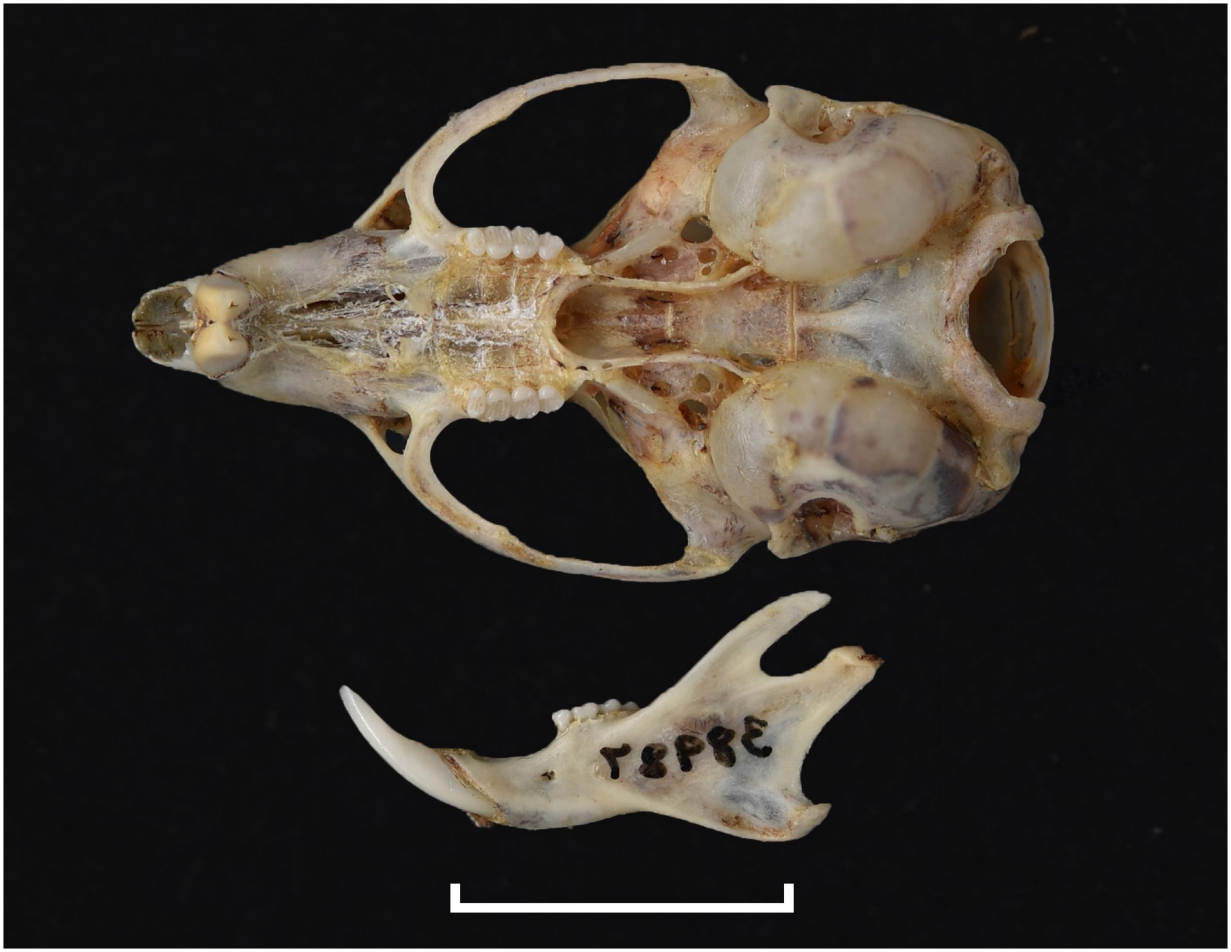
Cranium of *Graphiurus murinus* (DNMNH-23386), with scale bar of 1 cm.

**Fig 117:**
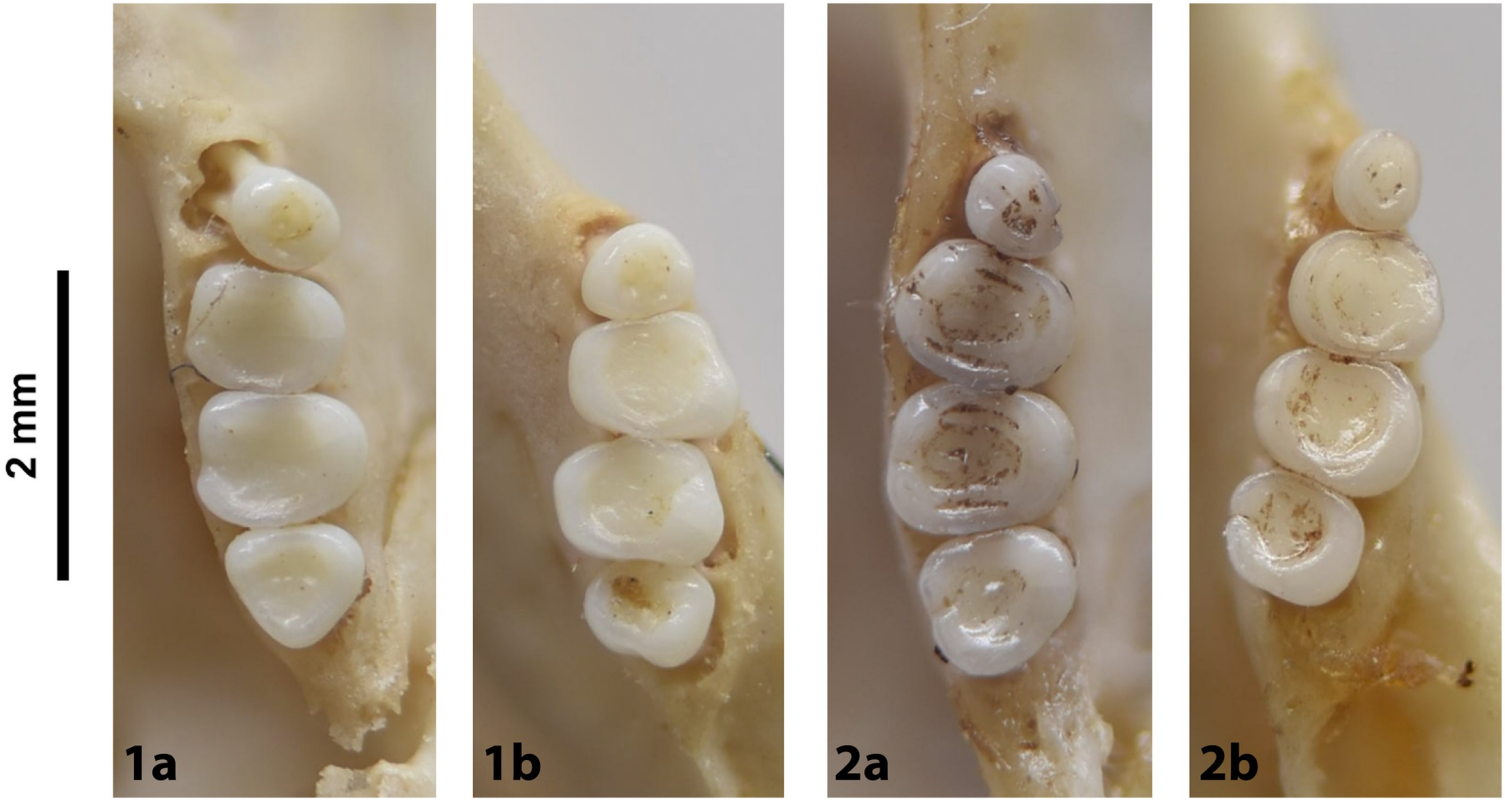
Cheekteeth of *Graphiurus*. 1) Upper (a) and lower (b) right toothrow of *G*. *platyops* (DNMNH-4360); 2) Upper (a) and lower (b) right toothrow of *G*. *ocularis* (DNMNH-27470).

**Fig 118:**
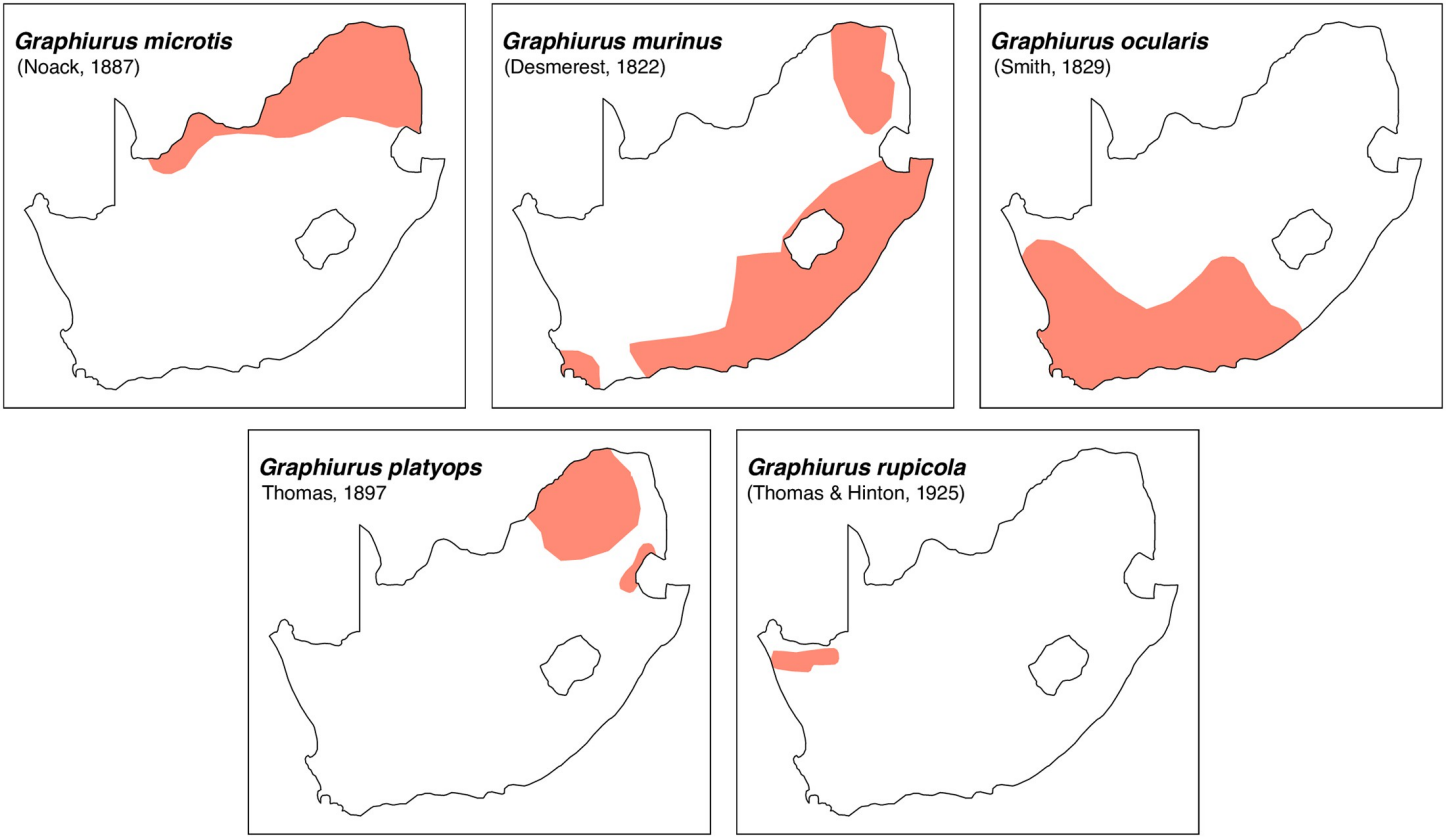
Distribution maps.

Figs 116–118; Table 42

**Table 42:** Dental measurements (in mm) for *Graphiurus* from South Africa, sexes and species combined.

	Mean	Min	Max	n
LLTR	3.0	2.7	3.3	18
WM_1_	1.0	0.7	1.1	21
LUTR	3.2	2.7	3.6	17
WM^1^	1.1	0.9	1.2	18

Dental formula is 1-0-1-3:1-0-1-3.

#### Upper jaw

Incisors are ungrooved, and orthodont to slightly opisthodont. Palatal foramina are small and reach far before the toothrow. The palate is wide, about equal to the length of the upper toothrow. The premolar P^4^ is roughly the same size or a bit smaller than M^3^, except in *G*. *ocularis* where P^4^ is much reduced. The occlusal surface of the teeth displays faint transverse ridges.

#### Lower jaw

Incisors are ungrooved. Lower cheekteeth also display faint transverse ridges. The mandible is elongated with a long coronoid process and a tilted angular process. The mental foramen is well marked.

#### Systematic notes and South African fossil record

Five species of *Graphiurus* are currently described in South Africa:

- *Graphiurus microtis* (Noack, 1887)
- *Graphiurus murinus* (Desmerest, 1822)
- *Graphiurus ocularis* (Smith, 1829)
- *Graphiurus platyops* Thomas, 1897
- *Graphiurus rupicola* (Thomas & Hinton, 1925)

The oldest remains of *Graphiurus* in South Africa are found in several Early Pleistocene deposits from the Sterkfontein Valley, in Gauteng Province [5, 47, 48, 74, 82].

### Sciuridae

**Fig 119:**
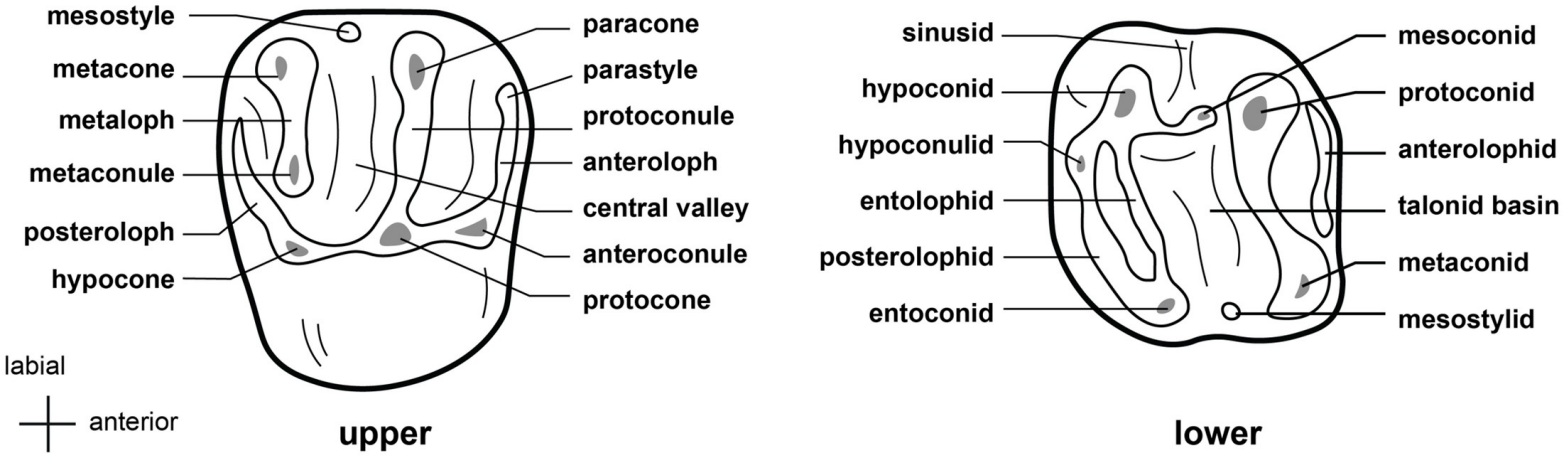
Sciurid right upper and left lower molars with nomenclature of the cusps, adapted from Cuenca-Bescós (1988) [86] and Viriot et al., (2011) [87].

Some two genera of Sciuridae (Tables 43 and 44) occur in RSA: *Paraxerus* and *Geosciurus* (which was included until recently in the genus *Xerus*). Species of the genus *Paraxerus* are small to medium-sized tree squirrels, which have predominantly diurnal and arboreal habits. They typically nest in tree holes and occupy forests and woodland areas. They are preyed upon by owls [24]. *Geosciurus* is a larger ground-dwelling squirrel, being terrestrial and resting in burrows, piles of rocks and termite mounds [21]. Members of *Geosciurus* are too big to be hunted by most nocturnal raptors, but Spotted eagle owl (*Bubo africanus*) and giant eagle owl (*Bubo lacteus*) have been reported preying upon them [83, 84]. The nomenclature of the teeth of fossil and modern representatives of African sciurids is provided in Denys et al. [85], along with a list of distinctive dental and skulls characters presented below (see Fig 119).

**Table 43:** Key to the sciurid genera: Upper jaw.

1	four cheekteeth; LUTR > 10 mm; in M^1^ and M^2^, metaloph is short and connected to the posteroloph; palatine bone extends well behind the molars	*Geosciurus*
	five cheekteeth; LUTR < 10 mm; in M^1^ and M^2^, metaloph is long and connected to the protocone; palatine bone extends only the posterior edge of the M^3^	*Paraxerus*

**Table 44:** Key to the sciurid genera: Lower jaw.

1	LLTR > 10 mm; in M_1_ and M_2_, protoconid and metaconid fused by posterior edge of cusps	*Geosciurus*
	LLTR < 9 mm; in M_1_ and M_2_, protoconid and metaconid fused by anterior edge of cusps	*Paraxerus*

Family **SCIURIDAE** Fischer de Waldheim, 1817

Genus ***Geosciurus*** Smith, 1834 (Ground squirrels)

**Fig 120:**
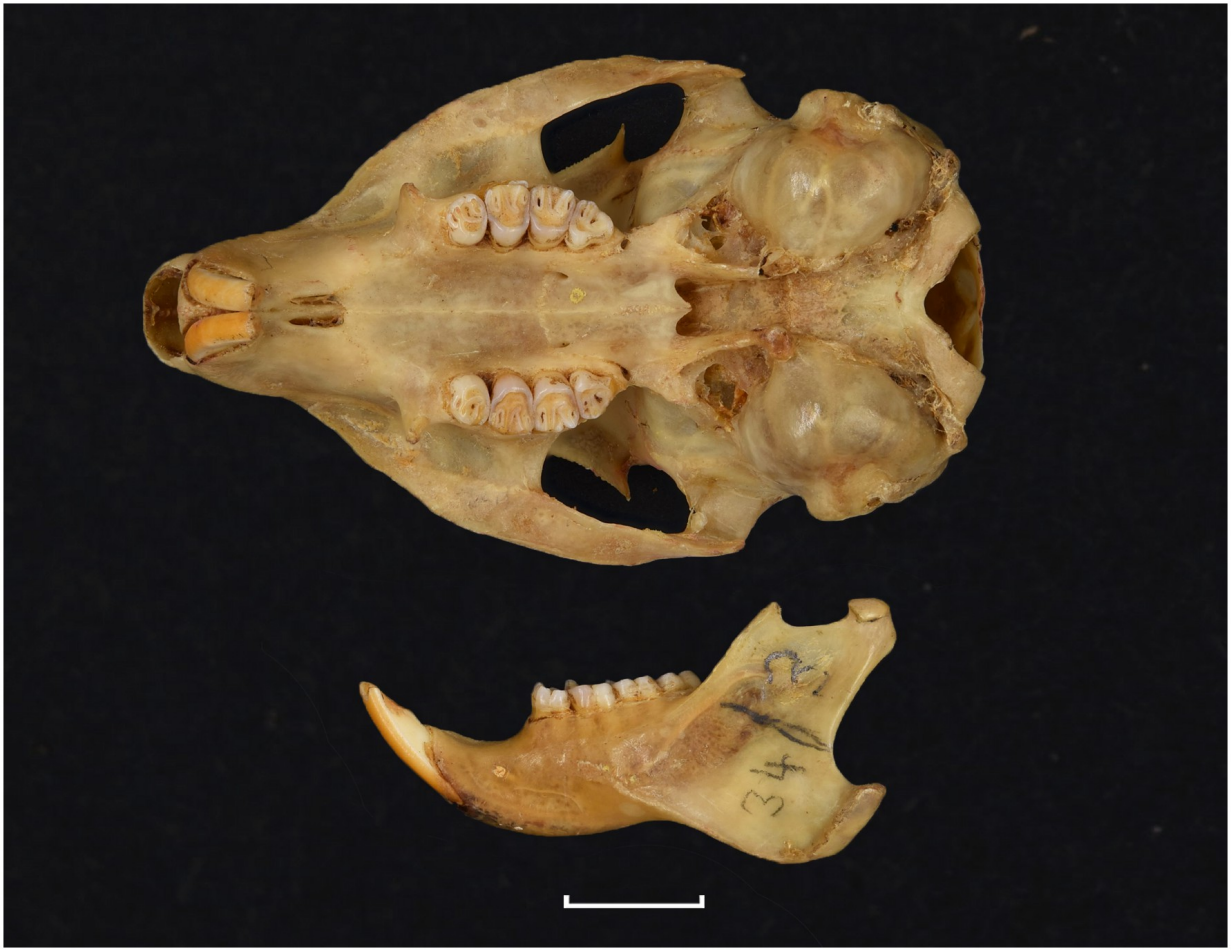
Cranium of *Geosciurus princeps* (DNMNH-8344), with scale bar of 1 cm.

**Fig 121:**
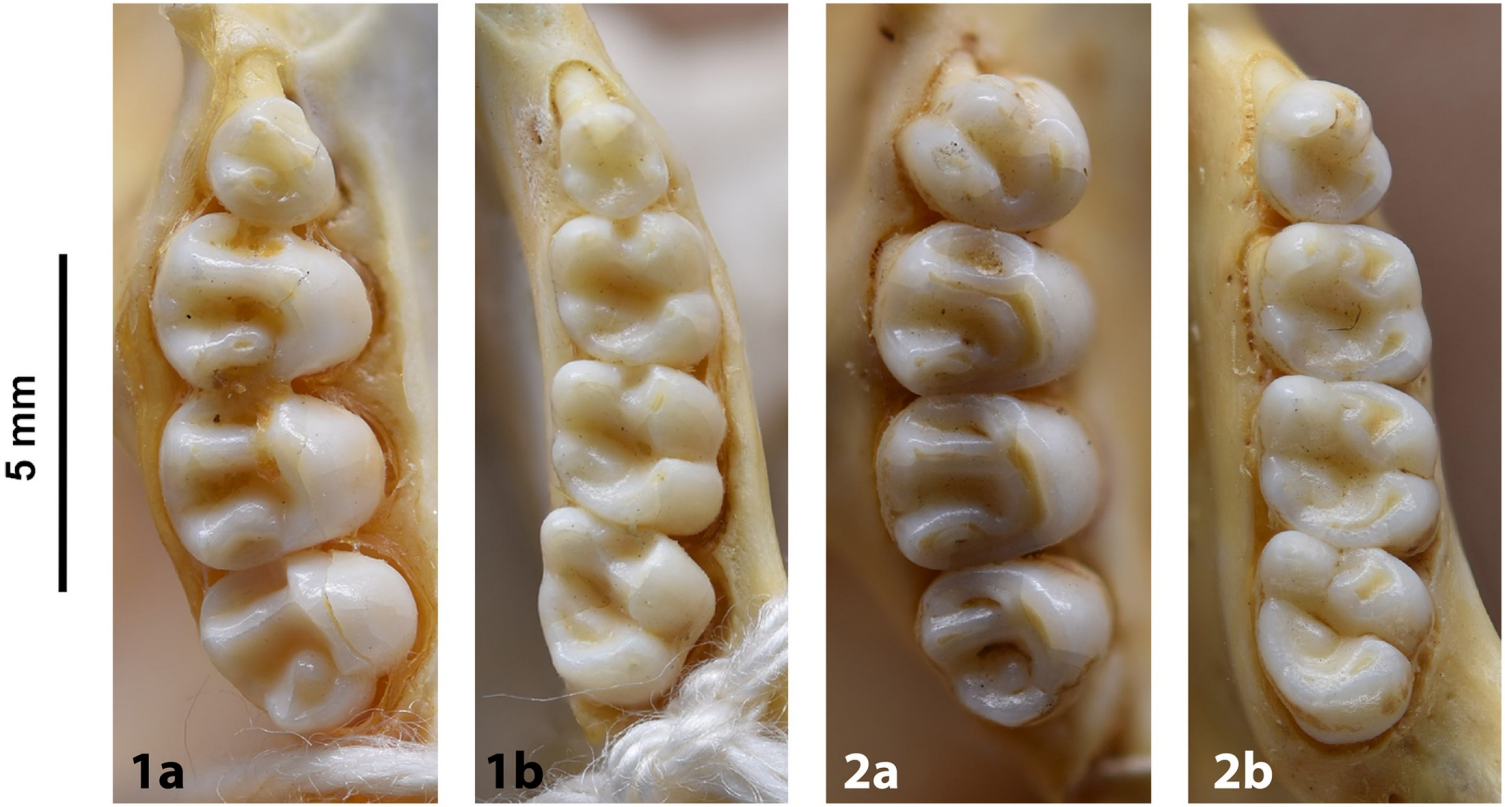
Craniodental anatomy of *Geosciurus*. 1) Upper (a) and lower (b) right toothrow of *G*. *inauris* (DNMNH-15143); 2) Upper (a) and lower (b) right toothrow of *G*. *princeps* (DNMNH-6327).

**Fig 122:**
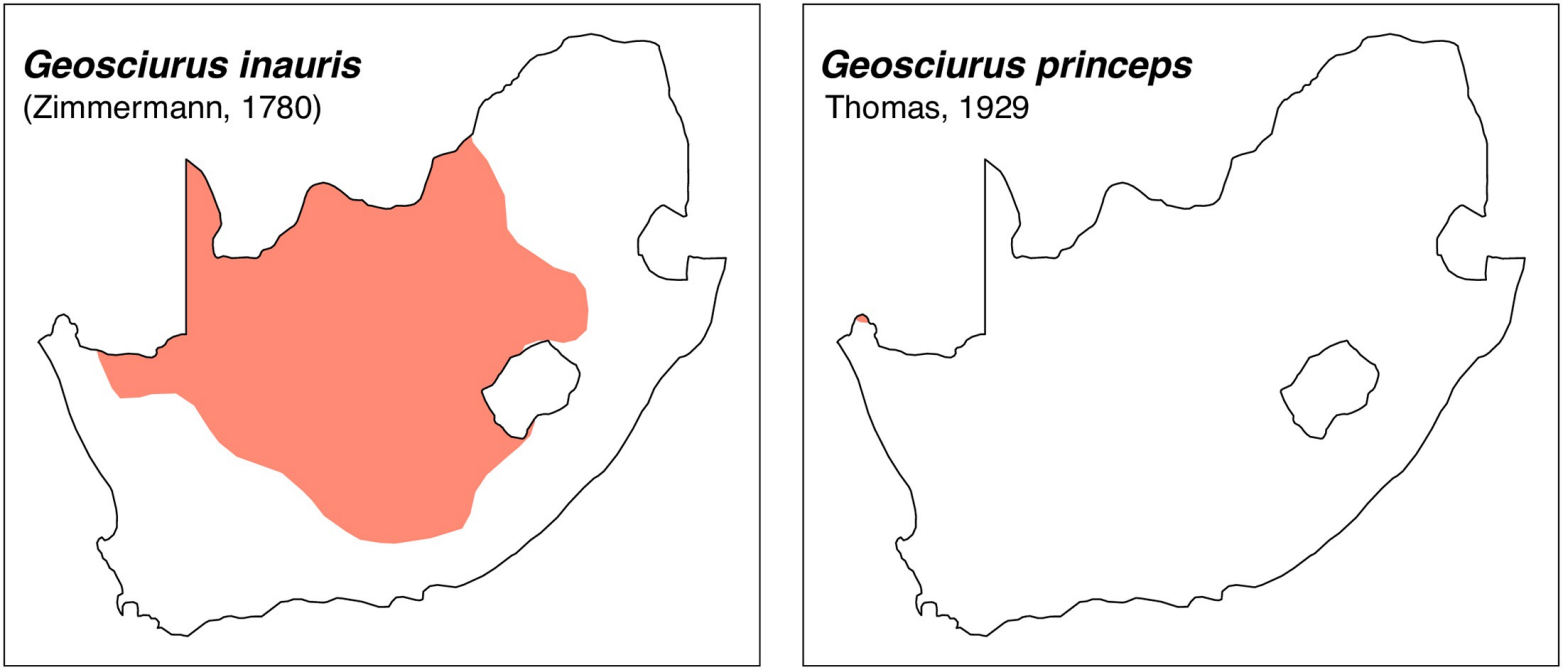
Distribution maps.

Figs 120–122; Table 45

**Table 45:** Dental measurements (in mm) for *Geosciurus* from South Africa, sexes and species combined.

	Mean	Min	Max	n
LLTR	12.1	11.3	13.3	32
WM_1_	3.3	3.0	3.9	32
LUTR	11.0	10.2	11.9	32
WM^1^	4.1	3.1	4.7	32

Dental formula is 1-0-1-3-:1-0-1-3.

#### Upper jaw

Upper incisors are ungrooved and opisthodont. The palatine bone extends well behind the molars. There is a well-developed masseter knob. A single premolar (P^4^) is present in the upper jaw (there are two premolars in *Paraxerus*). Molars have three transverse lophs (anteroloph, metaloph, posteroloph) relating bunodont cusps on the occlusal surface. They display a big protocone on the whole lingual part of the molar, while the hypocone is hardly visible. In M^1^ and M^2^, the metaloph is short and is connected to the posteroloph. The presence of a mesostyle varies among specimens.

#### Lower jaw

Lower incisors are ungrooved. Molars have well individualized bunodont cusps (a prominent hypoconid and protoconid, a metaconid and a crestiform entoconid) connected by transverse ridges (protolophid, entolophid, posterolophid) that develop with wear. The molars have a prominent hypoconid In M_1_ and M_2_, the cusps protoconid and metaconid are fused by their distal side (anterior side in *Paraxerus*). The mandible is sciurognath, with its angular part stockily built.

#### Systematic notes and South African fossil record

This genus was previously included within *Xerus*, until recent phylogenetic reconstructions led to the recognition of the genus *Geosciurus* for specimens from Southern Africa [88]. Two species are currently recognised:

- *Geosciurus inauris* (Zimmermann, 1780)
- *Geosciurus princeps* Thomas, 1929

Remains of this genus are scanty in the Quaternary fossil record. The oldest record of *Geosciurus* is from the Middle Pleistocene locality of Florisbad [89, 90].

Genus ***Paraxerus*** Forsyth Major, 1893 (Bush Squirrels)

**Fig 123:**
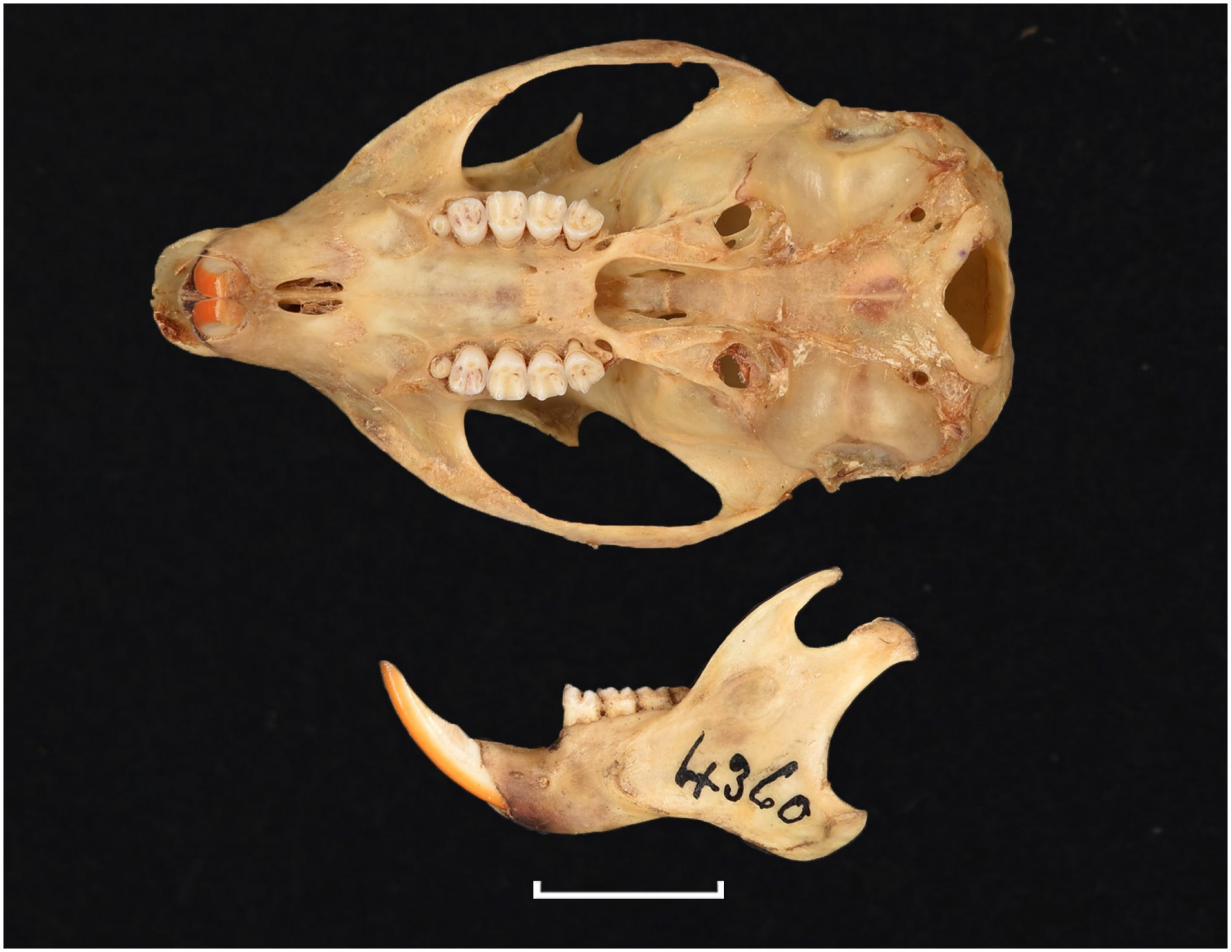
Cranium of *Paraxerus cepapi* (skull DNMNH-4363, mandible DNMNH-6322), with scale bar of 1 cm.

**Fig 124:**
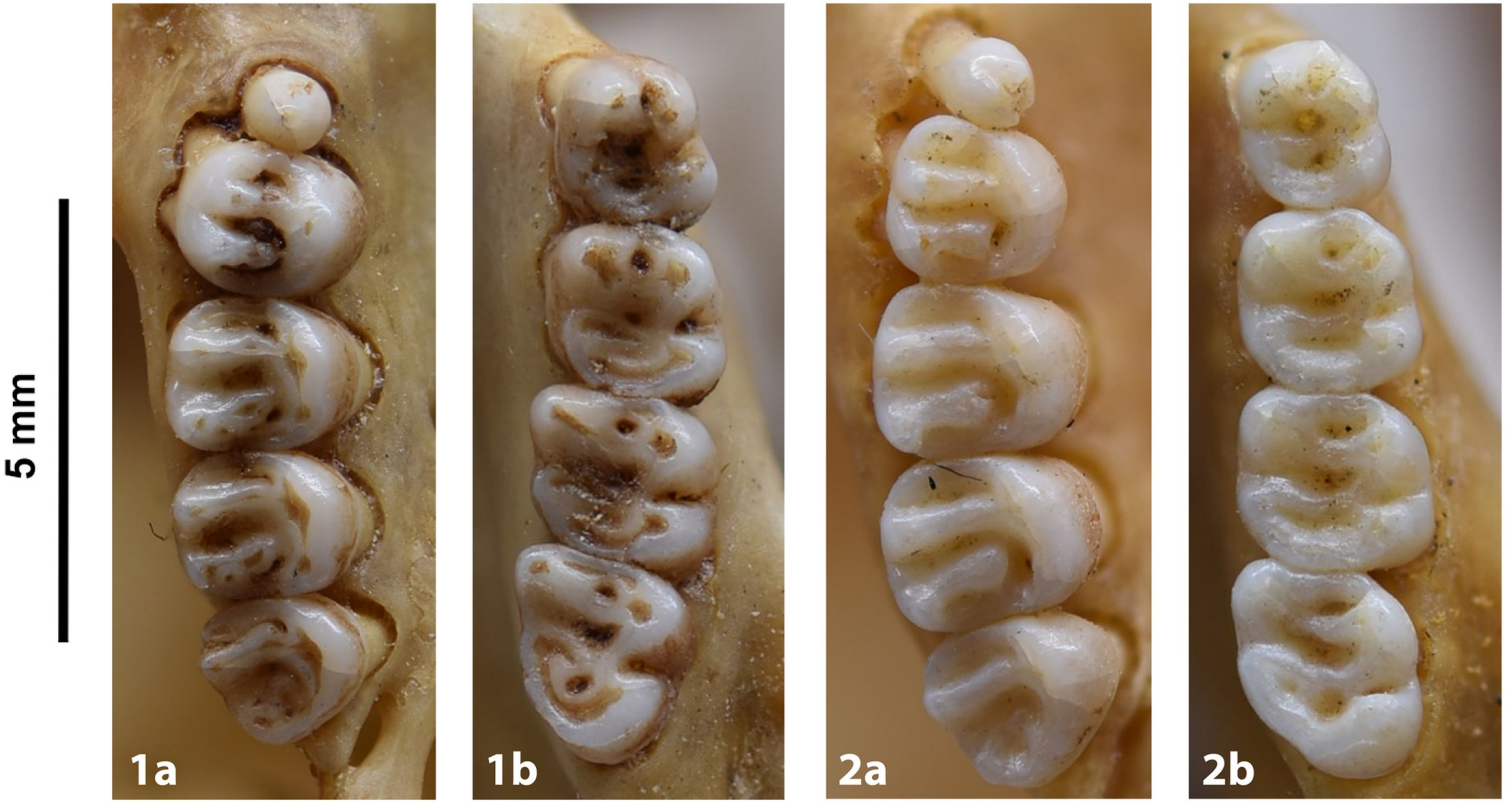
Cheekteeth of *Paraxerus*. **1)** Upper (a) and lower (b) right toothrow of *P*. *cepapi* (DNMNH-4360); **2)** Upper (a) and lower (b) right toothrow of *P*. *palliatus* (DNMNH-6214).

**Fig 125:**
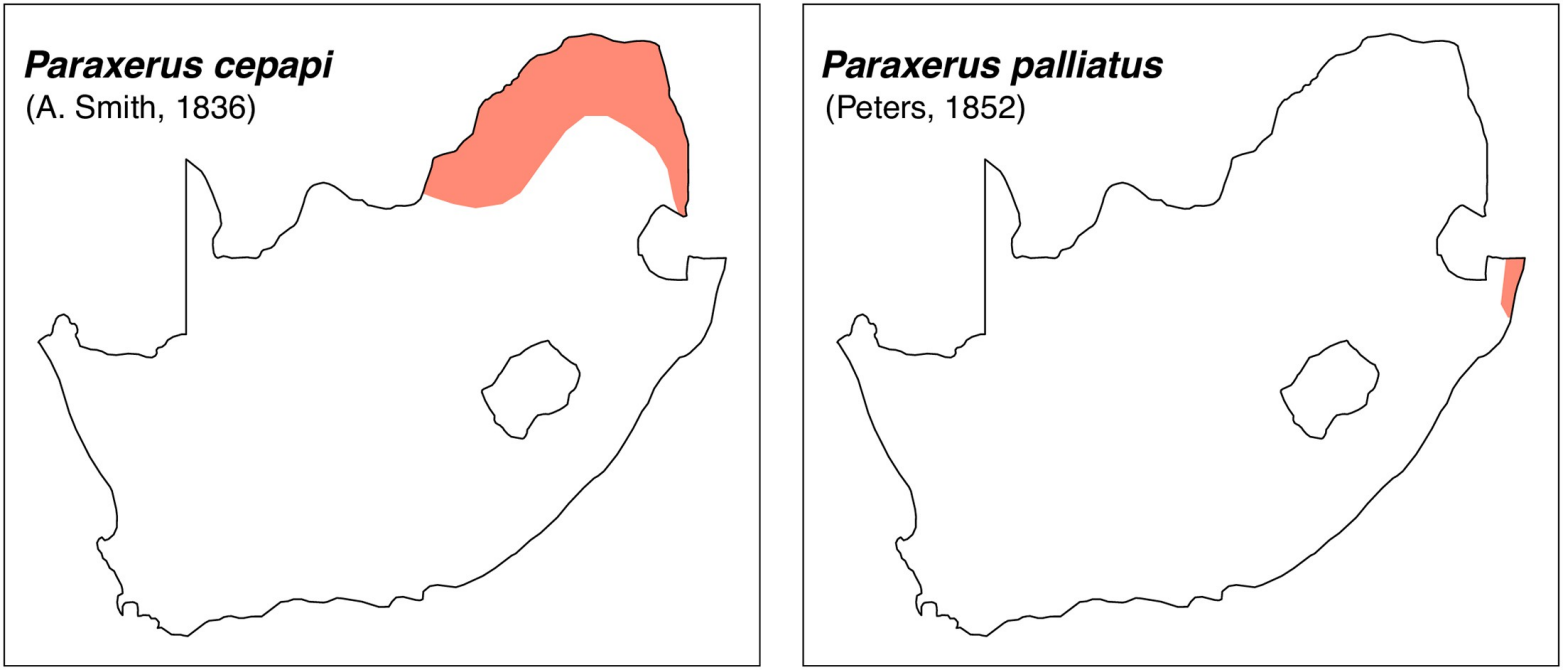
Distribution maps.

Figs 123–125; Table 46

**Table 46:** Dental measurements (in mm) for *Paraxerus* from South Africa, sexes and species combined.

	Mean	Min	Max	n
LLTR	8.4	7.6	9.3	30
WM_1_	2.2	1.9	2.4	30
LUTR	8.4	7.2	9.7	28
WM^1^	2.6	2.3	3.0	29

Dental formula is 1-0-2-3:1-0-1-3.

#### Upper jaw

Upper incisors are orthodont and ungrooved. The palatine bone extends only the posterior edge of the M^3^. The anterior palatal foramina are short and located far forward from the teeth. There are five upper cheekteeth, with the presence of a minute P^3^. Molars are of approximately the same size and display transverse lophs (anteroloph, protoloph, metaloph, posteroloph) relating bunodont cusps on the occlusal surface. M^1^ and M^2^ both have three transverse valleys, M^3^ has two. In M^1^-M^2^, the metaloph is long and connected to the protocoone, and the mesostyle is absent. In the M^3^ the hypocone and protocone are connected.

#### Lower jaw

Lower incisors are ungrooved. Molars are ridged more or less transversely and display four main cusps which are not always visible with wear: a big protoconid fused to a metaconid by the anterior side (posterior in *Geosciurus*), a hypoconid and a transverse entoconid. The M_3_ is the largest tooth of the molar row. The mandible is sciurognath; it has a high coronoid process, well segregated from the condylar process.

#### Systematic notes and South African fossil record

Two species are currently recognized in South Africa:

- *Paraxerus cepapi* (A. Smith, 1836)
- *Paraxerus palliates* (Peters, 1852)

Fossils of this genus are rare in the Quaternary fossil record, with a single Pleistocene record from Border Cave [91] and a few Holocene records [50].

## 6. Discussion and conclusion

Recent advances in rodent taxonomy at the subfamily, genus, and species level, together with the need for more detailed description of their dental anatomy, has encouraged us to provide an updated version of identification keys previously published by Coetzee [23] and De Graaff [24], based on standard, and new, morphological characters. This contribution has endeavored to assemble the latest information regarding rodent systematics, taxonomy, and palaeontology. We aim to support and facilitate the identification of rodent specimens from Quaternary and modern coprocenoses by providing a simple identification tool based on a traditional dichotomous system and scaled photographs of the skull and dentition.

We have attempted to compile the most reliable characters based on collection specimens identified on cytogenetic or molecular grounds mainly. Some published questionable characters were discarded (for instance, the presence of an accessory anterior median cusp on M^1^ was previously used to distinguish *Thallomys* from *Grammomys*) and characters likely to display variability have received less emphasis in this work than in previous identification keys (such as the presence of accessory cusplets or posterior cingulum). Instead, we have retained mainly those features that we were able to observe consistently in the collection specimens ourselves.

Regrettably, however, isolated teeth and fragmented specimens from owl pellet or fossil material may remain unidentifiable, even at the genus level. Most of the criteria used in our key involve having the complete dental row preserved. In a similar way, this key does not include genera found in the savannahs and deserts of Botswana, Zambia, Namibia and Zimbabwe that were possibly found further south or west during the Pleistocene period. This remark concerns, for instance, the genus *Pelomys*, which was identified by some authors among the fossil remains from Makapansgat, Border Cave, and Sterkfontein [47, 70, 92].

Accurate taxonomic identification is essential for a wide range of scientific applications, from ecology, conservation biology and pest management, to archaeozoology and palaeoenvironmental reconstruction. The species level generally constitutes the fundamental unit of investigation. For well-preserved modern specimens, one may have little difficulty in identifying features characteristic of a species using criteria available in literature coupled with biogeographic data. Works of this kind are to be found in general monographs or more specialised journal articles, and we have elaborated on the factors that led us to restrict our key to genus level. Problems arise with broken specimens from modern and palaeo coprocenoses, which lack diagnostic criteria. Moreover, several species cannot be distinguished on the basis of craniodental anatomy unless time-intensive techniques such as geometric morphometrics are employed, and this may not always be feasible. Finally, when it comes to fossils, the use of distribution data is extremely risky, and may explain the disagreement that exists between some researchers on the status of different fossil species, especially those described during the last century (this is the case, for example, of the large number of species described by Broom in the 1930s and 1940s).

Ironically, the huge progress made in systematics and species identification through the use of numerical taxonomy and molecular taxonomy has eroded our confidence in identifying species based on bone and teeth remains. However, the discovery of new fossil rodents in South Africa is continuing at a rapid pace, and taxonomic identification of craniodental material remains the foundation of palaeontological and archaeological research. Identification skills rely mainly on experience and repeated observations of key features. In this respect, this key should be a valuable resource for both professionals and amateurs alike. It is particularly beneficial regarding the numerous genera which have similar morphological features, and which are challenging to differentiate. Nevertheless, we must emphasize that the identification key is intended to complement the use of official natural history collections in order to facilitate accurate identification.

## Supporting information

S1 Fig Comparative osteological plates. Upper jaws.
(PDF)

S2 Fig Comparative osteological plates. Lower jaws.
(PDF)

S1 Checklist References of specimens used for osteological plates. (DOCX)

S1 Table Material examined for measurements. (XLSX)
