# Supplementary material for: Quaternary rodents of South Africa: A companion guide for cranio-dental identification
Source: PLoS One. 2023 Nov 28;18(11):e0289812. doi: 10.1371/journal.pone.0289812 (PMC10684104; doi:10.1371/journal.pone.0289812)
Supplement: S1 Fig — Upper jaws. (PDF) [file pone.0289812.s001.pdf]

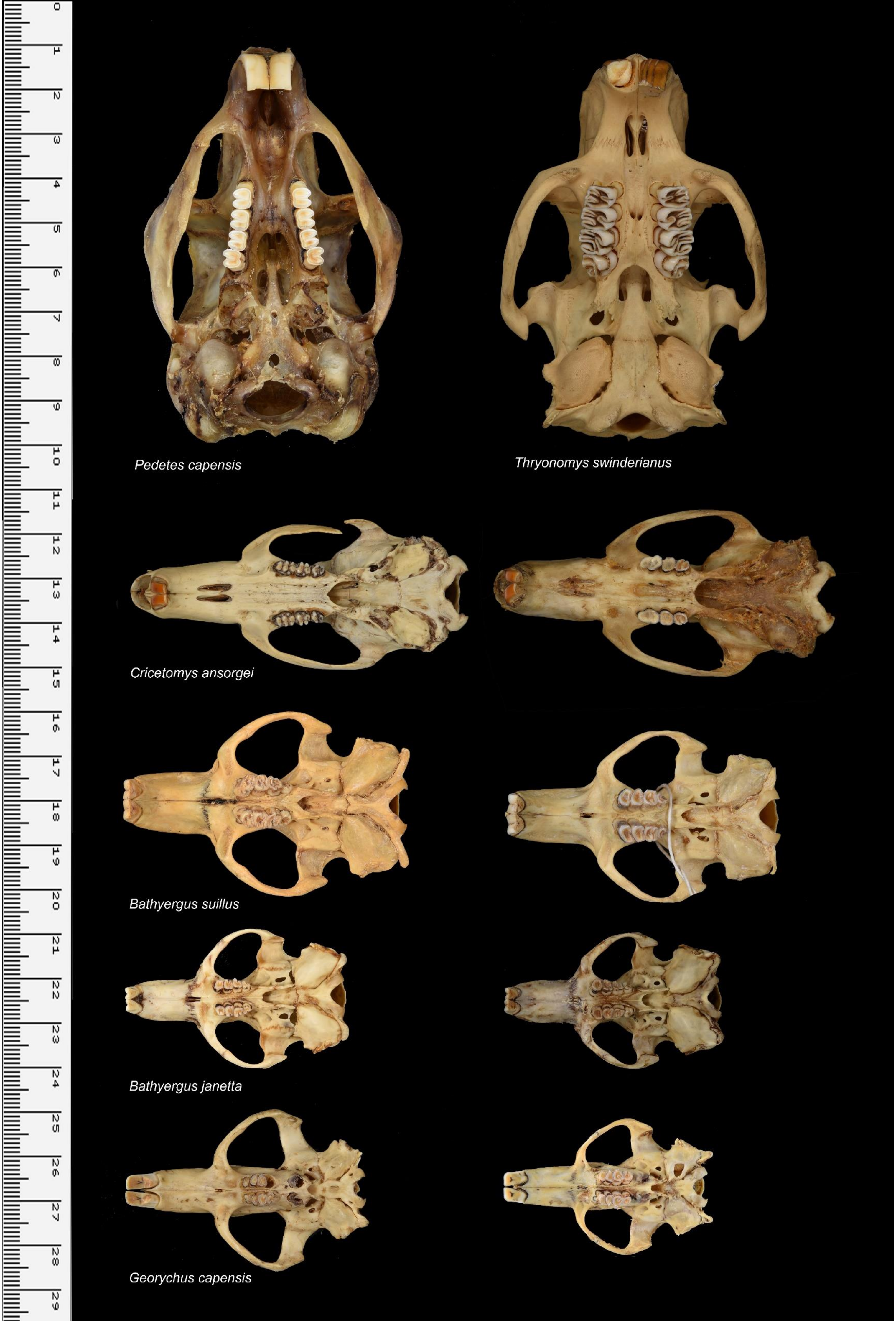
Pedetes capensis
Thryonomys swinderianus
Cricetomys ansorgei
Bathyergus suillus
Bathyergus janetta
Georychus capensis

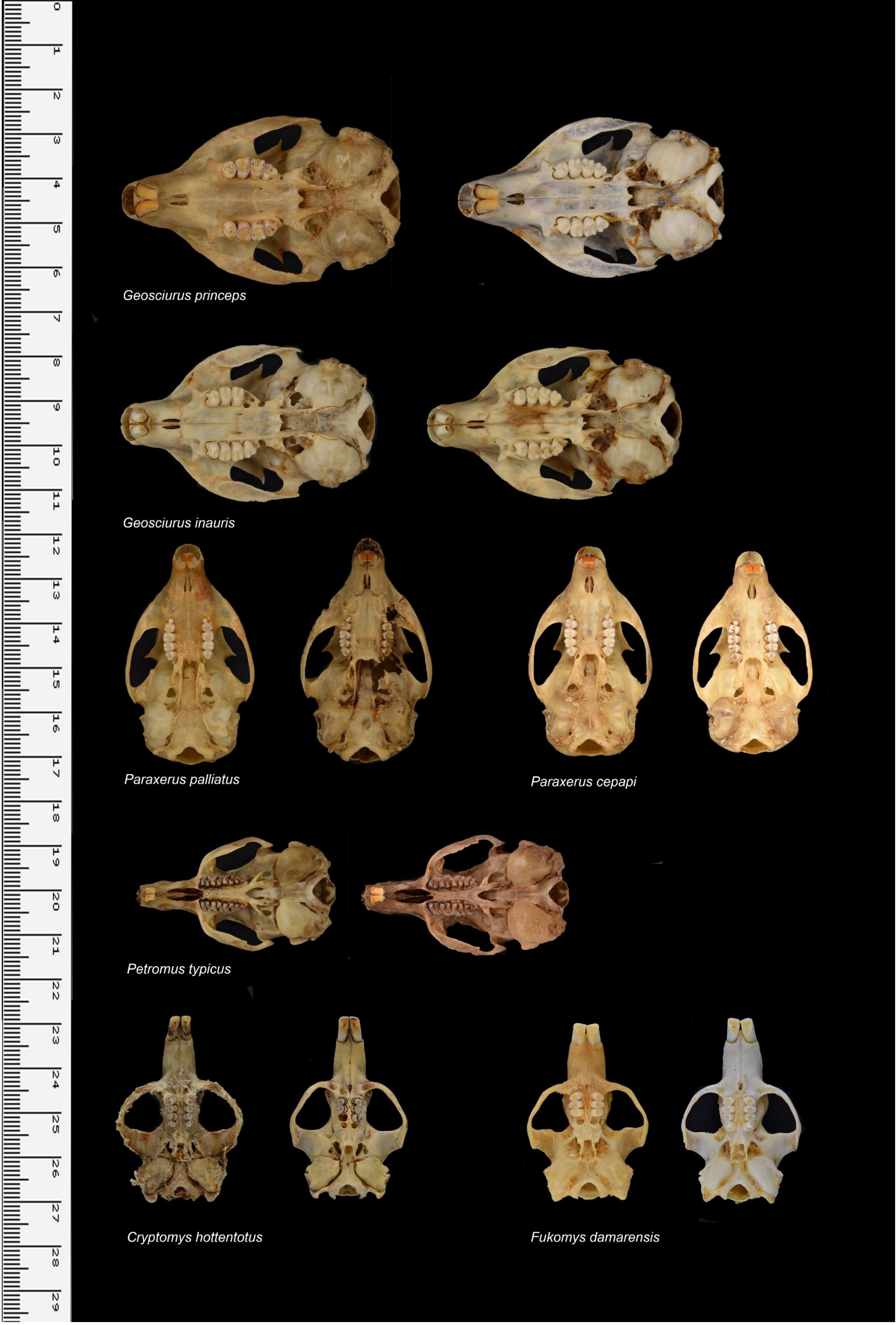
Geosciurus princeps
Geosciurus inauris
Paraxerus palliatus
Paraxerus cepapi
Petromus typicus
Cryptomys hottentotus
Fukomys damarensis

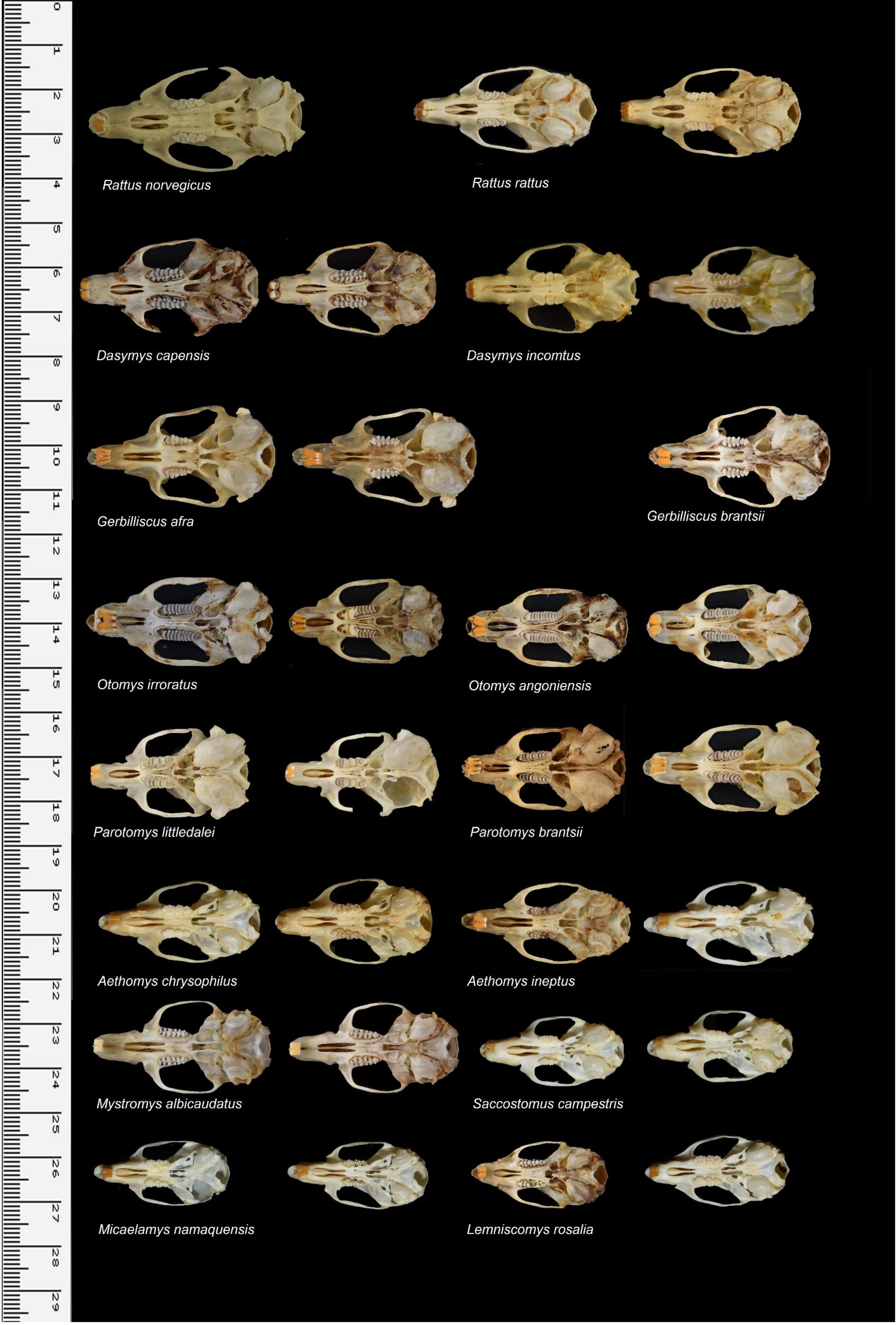
Rattus norvegicus
Rattus rattus
Dasymys capensis
Dasymys incomtus
Gerbilliscus afra
Gerbilliscus brantsii
Otomys irroratus
Otomys angoniensis
Parotomys littledalei
Parotomys brantsii
Aethomys chrysophilus
Aethomys ineptus
Mystromys albicaudatus
Saccostomus campestris
Micaelamys namaquensis
Lemniscomys rosalia

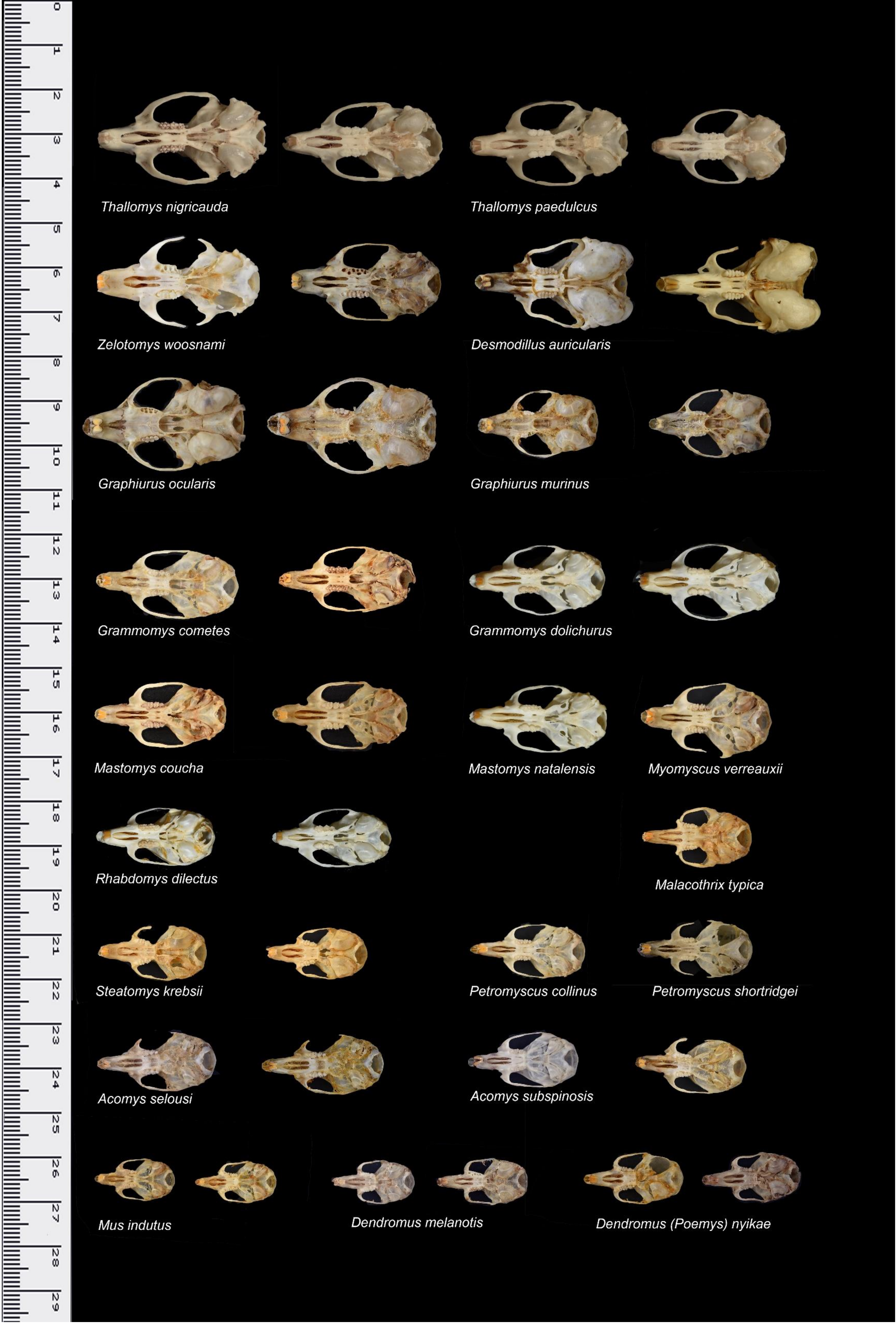
Thallomys nigricauda
Thallomys paedulcus
Zelotomys woosnami
Desmodillus auricularis
Graphiurus ocularis
Graphiurus murinus
Grammomys cometes
Grammomys dolichurus
Mastomys coucha
Mastomys natalensis
Myomyscus verreauxii
Rhabdomys dilectus
Malacothrix typica
Steatomys krebsii
Petromyscus collinus
Petromyscus shortridgei
Acomys selousi
Acomys subspinosis
Mus indutus
Dendromus melanotis
Dendromus (Poemys) nyikae
