# Supplementary material for: Quaternary rodents of South Africa: A companion guide for cranio-dental identification
Source: PLoS One. 2023 Nov 28;18(11):e0289812. doi: 10.1371/journal.pone.0289812 (PMC10684104; doi:10.1371/journal.pone.0289812)
Supplement: S2 Fig — Lower jaws. (PDF) [file pone.0289812.s002.pdf]

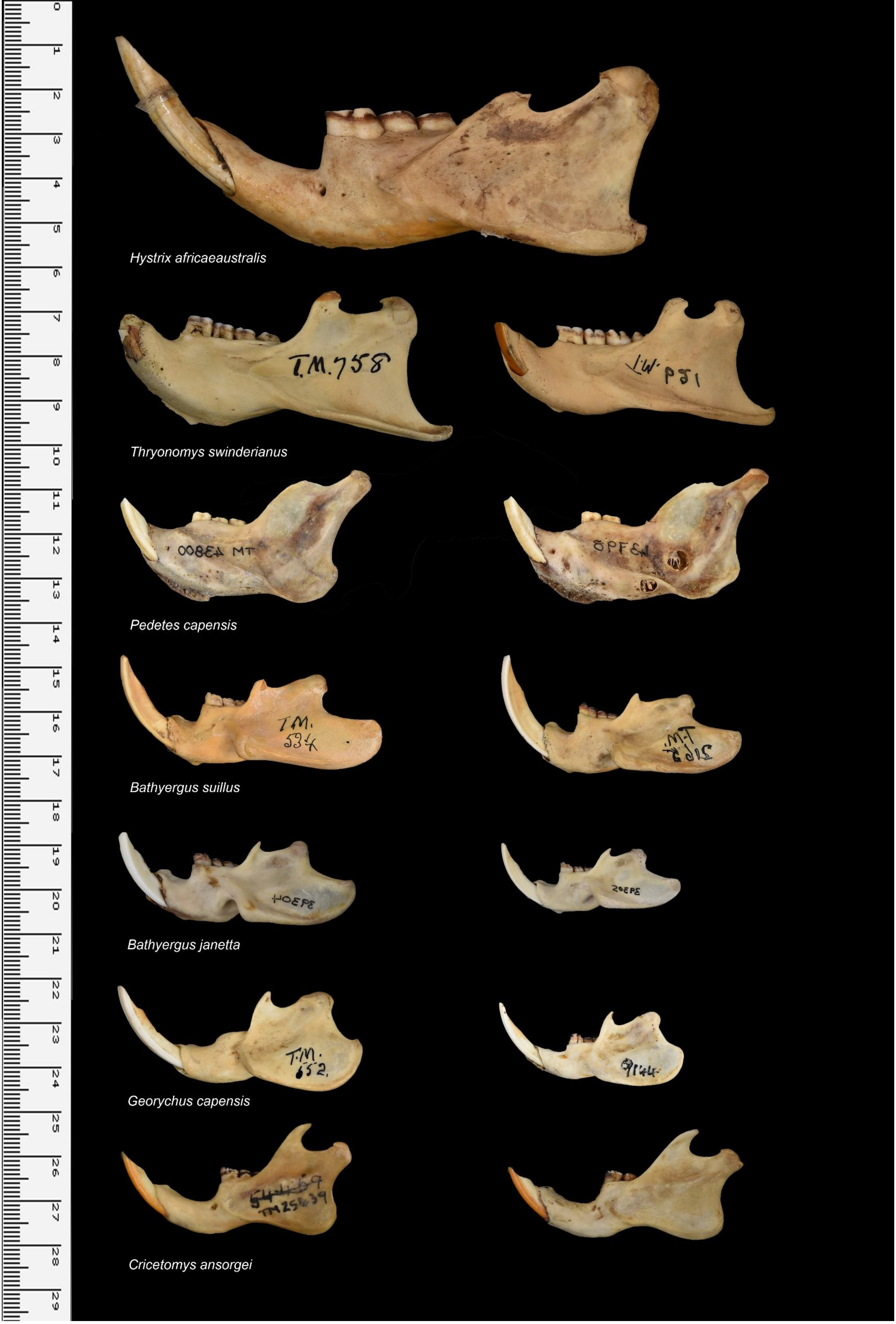
Hystrix africaeaustralis
Thryonomys swinderianus
Pedetes capensis
Bathyergus suillus
Bathyergus janetta
Georychus capensis
Cricetomys ansorgei

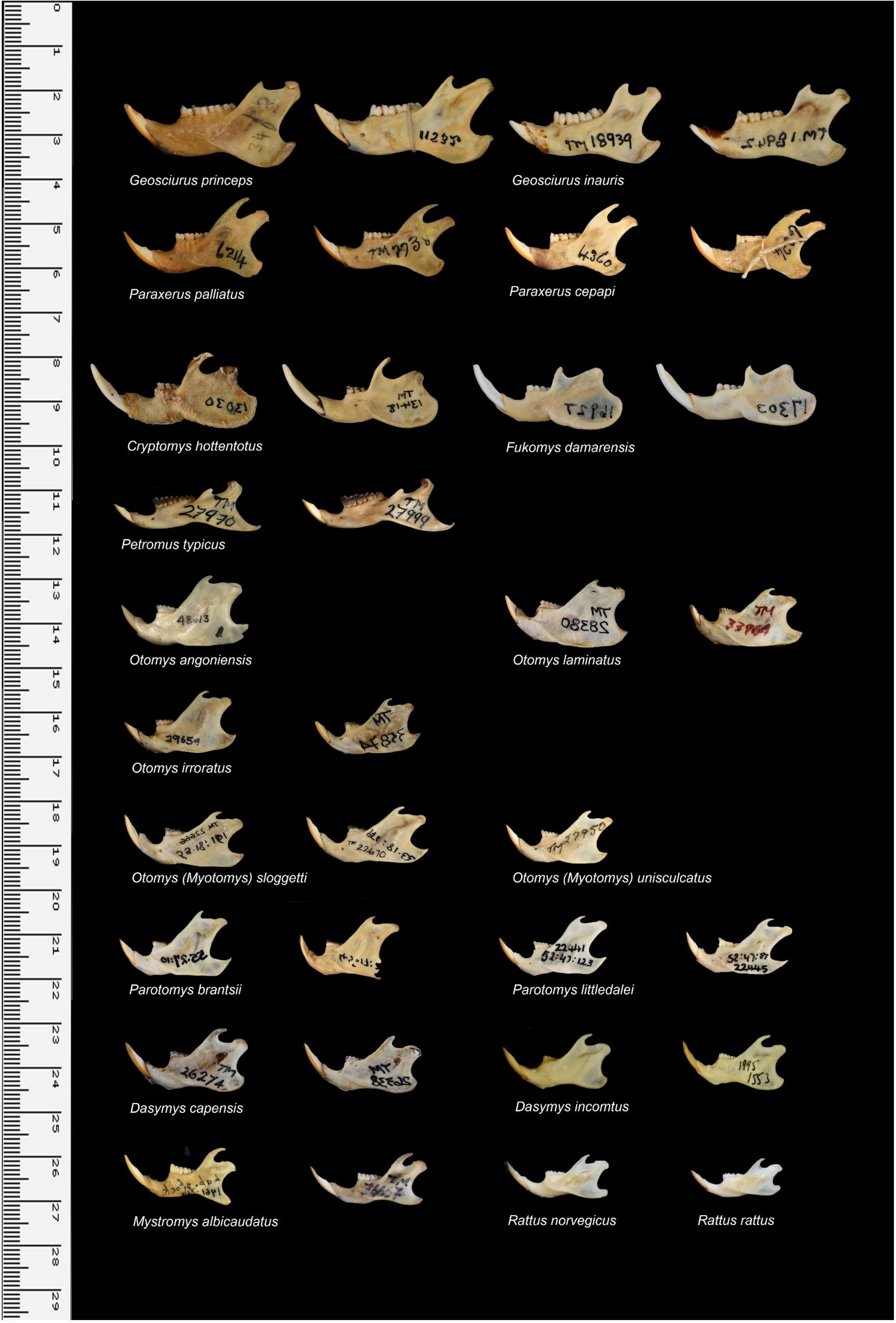
Geosciurus princeps
Geosciurus inauris
Paraxerus palliatus
Paraxerus cepapi
Cryptomys hottentotus
Fukomys damarensis
Petromus typicus
Otomys angoniensis
Otomys laminatus
Otomys irroratus
Otomys (Myotomys) sloggetti
Otomys (Myotomys) unisculcatus
Parotomys brantsii
Parotomys littledalei
Dasymys capensis
Dasymys incomtus
Mystromys albicaudatus
Rattus norvegicus
Rattus rattus

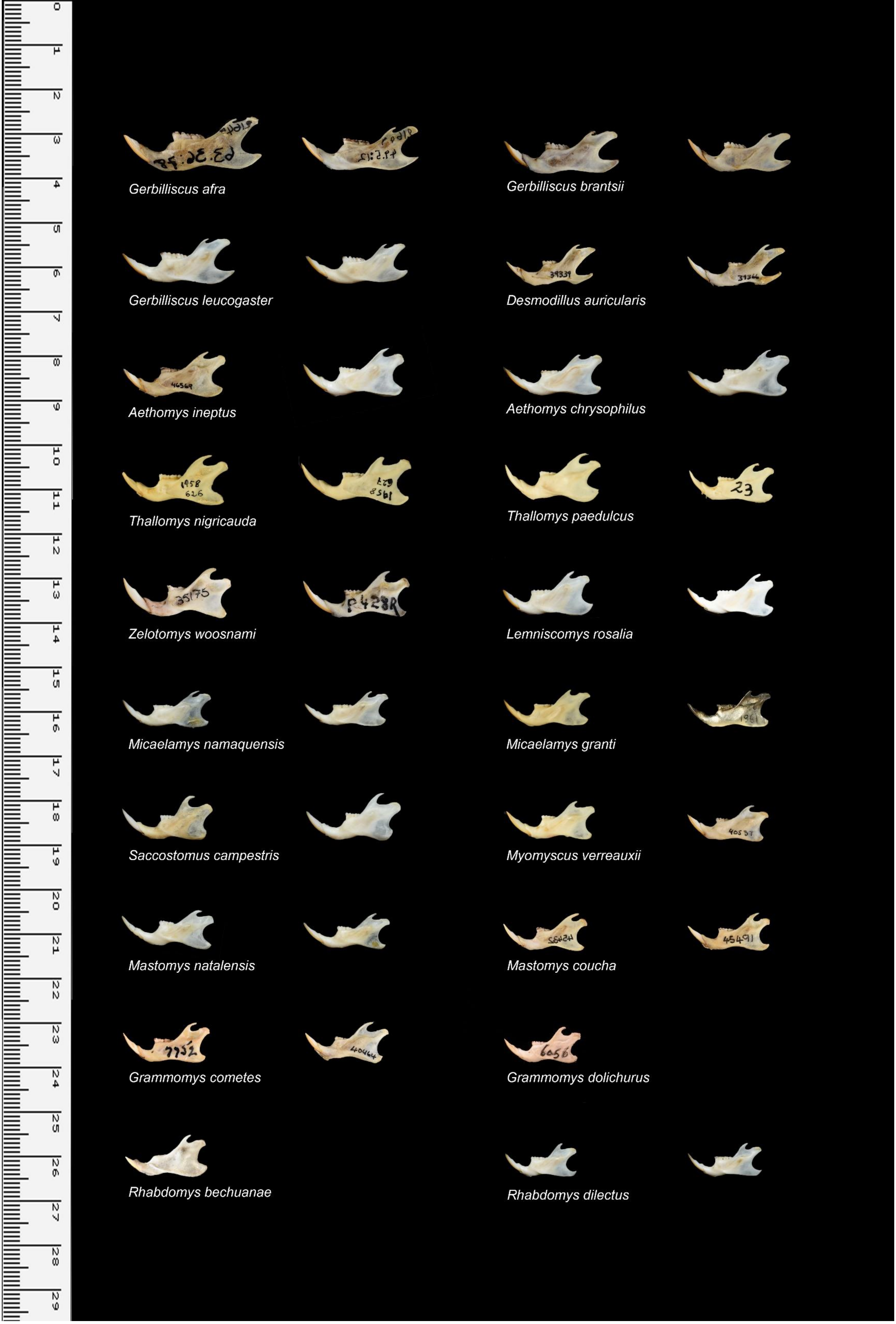
Gerbilliscus afra
Gerbilliscus brantsii
Gerbilliscus leucogaster
Desmodillus auricularis
Aethomys ineptus
Aethomys chrysophilus
Thallomys nigricauda
Thallomys paedulcus
Zelotomys woosnami
Lemniscomys rosalia
Micaelamys namaquensis
Micaelamys granti
Saccostomus campestris
Myomyscus verreauxii
Mastomys natalensis
Mastomys coucha
Grammomys cometes
Grammomys dolichurus
Rhabdomys bechuanae
Rhabdomys dilectus

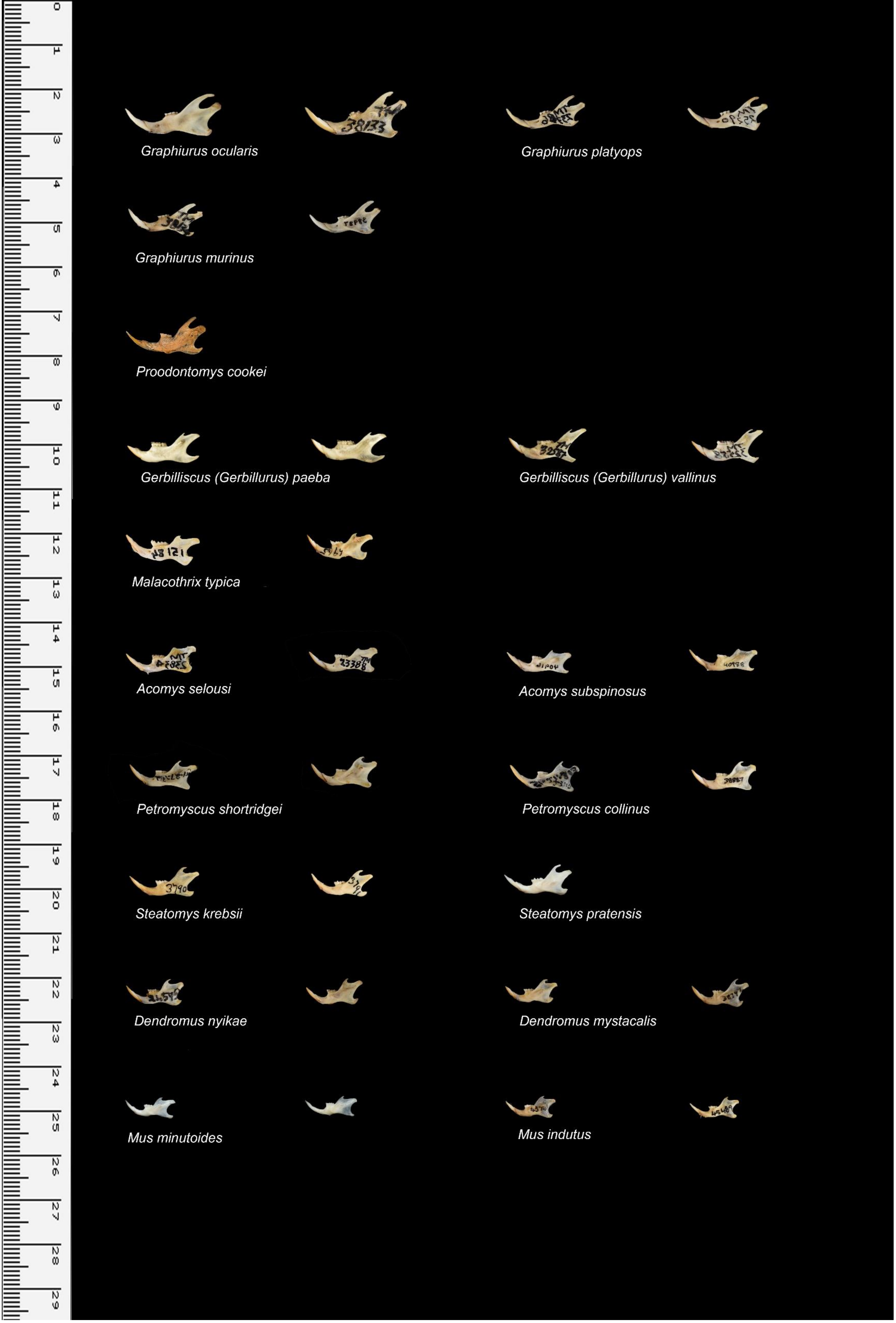
Graphiurus ocularis
Graphiurus platyops
Graphiurus murinus
Proodontomys cookei
Gerbilliscus (Gerbillurus) paeba
Gerbilliscus (Gerbillurus) vallinus
Malacothrix typica
Acomys selousi
Acomys subspinosus
Petromyscus shortridgei
Petromyscus collinus
Steatomys krebsii
Steatomys pratensis
Dendromus nyikae
Dendromus mystacalis
Mus minutoides
Mus indutus
