# Supplementary material for: Quaternary rodents of South Africa: A companion guide for cranio-dental identification
Source: PLoS One. 2023 Nov 28;18(11):e0289812. doi: 10.1371/journal.pone.0289812 (PMC10684104; doi:10.1371/journal.pone.0289812)
Supplement: S1 Checklist — (DOCX) [file pone.0289812.s003.docx]

**Supplementary III. List of specimens displayed in the anatomical plates**

*Rodents of South Africa: a companion identification guide for Quaternary archaeologists*

Linchamps P., Avery D. M., Cornette R., Denys C., Matthews T., Stoetzel E.

**A. Upper jaws (Supplementary I)**

Read from left to right, from top to bottom:

**Plate 1**

*Pedetes capensis*: DNMNH-44480

*Thryonomys swinderianus*: DNMNH-621

*Cricetomys ansorgei*: DNMNH-13954, DNMNH-30736

*Bathyergus suillus*: DNMNH-534, DNMNH-523

*Bathyergus janetta*: DNMNH-39307, DNMNH-39332

*Georychus capensis*: DNMNH-552, DNMNH-9145

**Plate 2**

*Geosciurus princeps*: DNMNH-8344, DNMNH-38442

*Geosciurus inauris*: DNMNH-18941, DNMNH-18942

*Paraxerus palliatus*: DNMNH-506, DNMNH-6214

*Paraxerus cepapi*: DNMNH-4363, DNMNH-6322

*Petromus typicus*: DNMNH-27970, DNMNH-27999

*Fukomys damarensis*: DNMN-17303, DNMN-16297

*Cryptomys hottentotus*: DNMNH13030, DNMNH13418

**Plate 3**

*Rattus norvegicus*: MNHN-ZM-MO-2019-1884

*Rattus rattus*: MNHN-ZM-MO-1994-784, MNHN-ZM-MO-1994-789

*Dasymys capensis*: DNMNH-26274, DNMNH-26338

*Dasymys incomtus*: MNHN-ZM-MO-1953-852, RMCA-78.039-M-0257

*Gerbilliscus afra*: DNMNH-21644, DNMNH-21640

*Gerbilliscus brantsii*: DNMNH-27751

*Otomys irroratus*: DNMNH-39455, DNMNH-35874

*Otomys angoniensis*: DNMNH-27526, DNMNH-24791

*Parotomys littledalei*: DNNNH-22441, DNNNH-22446

*Parotomys brantsii*: DNNNH-22614, DNNNH-22620

*Aethomys chrysophilus*: IVB-M8x0024, IVB-MOZ054

*Aethomys ineptus*: DNMNH-4659, IVB-MOS001

*Mystromys albicaudatus*: DNMNH-36667, DNMNH-36665

*Saccostomus campestris*: IVB-MOS14

*Micaelamys namaquensis*: IVB-MOZ002, IVB-RS1210

*Lemniscomys rosalia*: DNMNH-29961, IVB-RS1611

**Plate 4**

*Thallomys nigricauda:* MNHN-ZM-MO-1962-1123, MNHN-ZM-MO-1958-628

*Thallomys paedulcus*: MNHN-ZM-MO-1990-333, MNHN-ZM-MO-1977-23

*Desmodillus auricularis*: DNMNH-39339, MNHN-ZM-MO-1983-814

*Graphiurus ocularis*: DNMNH-27470, DNMNH-38133

*Graphiurus murinus*: DNMNH-38170, DNMNH-38987

*Grammomys cometes*: DNMNH-7752, DNMNH-40464

*Grammomys dolichurus*: IVB-RS0756, IVB-RS0825

*Mastomys coucha*: DNMNH-45432, DNMNH-45491

*Mastomys natalensis*: IVB- RS1103

*Myomyscus verreauxii*: DNMNH-40537

*Rhabdomys dilectus*: IVB-KE257, IVB-T8x331

*Malacothrix typica*: DNMNH-4965

*Steatomys krebsii*: DNMNH-3790, DNMNH-3792

*Petromyscus collinus*: DNMNH-23110

*Petromyscus shortridgei*: DNMNH-23111

*Acomys selousi*: DNMNH-23834, DNMNH-23612

*Acomys subspinosus*: DNMNH-40916, DNMNH-40988

*Mus indutus*: DNMNH-45741, DNMNH-46896

*Dendromus melanotis*: DNMNH-25617, DNMNH-30114

*Dendromus nyikae*: DNMNH-24595, DNMNH-34625

**B. Lower jaws (Supplementary II)**

Read from left to right, from top to bottom:

**Plate 1**

*Hystrix africaeaustralis*: ESI-BP-4-881

*Thryonomys swinderianus*: DNMNH-621, DNMNH-7433

*Pedetes capensis*: DNMNH-4800, DNMNH-43798

*Bathyergus suillus*: DNMNH-534, DNMNH-2165

*Bathyergus janetta*: DNMNH-39304, DNMNH-3905

*Georychus capensis*: DNMNH-552, DNMNH-9144

*Cricetomys ansorgei*: DNMNH-30736, DNMNH-25639

**Plate 2**

*Geosciurus princeps*: DNMNH-8344, DNMNH-11532

*Geosciurus inauris*: DNMNH-18939, DNMNH-18942

*Paraxerus palliatus*: DNMNH-6214, DNMNH-7738

*Cryptomys hottentotus*: DNMNH-13030, DNMNH-13418

*Fukomys damarensis*: DNMNH-16927, DNMNH-17303

*Rattus norvegicus*: MNHN-2019-1884

*Rattus rattus*: MNHN-1994-784, MNHN-1994-789

*Petromus typicus*: DNMNH-27970, DNMNH-27999

*Otomys laminatus*: DNMNH-28380, DNMNH-33964

*Otomys irroratus:* DNMNH-29659, DNMNH-35874

*Otomys angoniensis*: DNMNH-24708

*Otomys sloggetti*: DNMNH-22666, DNMNH-22670

*Otomys unisulcatus*: DNMNH-27950

*Parotomys brantsii*: DNMNH-22620, DNMNH-22614

*Parotomys littledalei*: DNMNH-22441, DNMNH-22445

*Dasymys capensis*: DNMNH-26274, DNMNH-52338

*Dasymys incomtus*: MNHN-ZM-MO-1995-1553, MNHN-ZM-MO-1953-852

*Mystromys albicaudatus*: MNHN-ZM-MO-1961-714, DNMNH-76667

**Plate 3**

*Gerbilliscus afra*: DNMNH-21640, DNMNH-21644

*Gerbilliscus brantsii*: DNMNH-27751, DNMNH-27756

*Gerbilliscus leucogaster*: IVB-JS105, IVB-JS159

*Desmodillus auricularis*: DNMNH-39339, DNMNH-39366

*Aethomys ineptus*: DNMNH-4659, IVB-MOS01

*Aethomys chrysophilus*: IVB-JS028, IVB-JS099

*Zelotomys woosnami*: DNMNH-37175, DNMNH-37801

*Lemniscomys rosalia*: IVB-RS1611, IVB-MOZ004

*Micaelamys namaquensis*: IVB-MOZ005, IVB-RS1206

*Micaelamys granti*: MNHN-ZM-MO-1964-289, MNHN-ZM-MO-1964-290

*Saccostomus campestris*: IVB-M8x0147, IVB-MOS14

*Myomyscus verreauxii*: MNHN-ZM-1964-287, DNMNH-40537

*Mastomys natalensis*: IVB-RS1365, IVB-RS1287

*Mastomys coucha*: DNMNH-45432, DNMNH-45491

*Grammomys cometes:* DNMNH-7752, DNMNH-40464

*Grammomys dolichurus:* RMCA-96.037-M-6370

*Rhabdomys bechuanae:* MNHN-ZM-2020-575 (KAL-2242)

*Rhabdomys dilectus:*IVB-T8x334, IVB-T8x3301

**Plate 4**

*Graphiurus ocularis*: DNMNH-27470, DNMNH-38133

*Graphiurus platyops:* DNMNH-23386, DNMNH-25290

*Graphiurus murinus:* DNMNH-38134, DNMNH-38987

*Proodontomys cookei:* ESI T.N. Pocock microfaunal fossil collection Kromdraai KA56 EXQRM57

*Gerbilliscus (Gerbillurus) paeba:* MNHN-ZM-2020-575 (KAL-2242 & KAL-2255)

*Gerbilliscus (Gerbillurus) vallinus:* DNMNH-32587, DNMNH-32596

*Malacothrix typica:* DNMNH-15184, DNMNH-4965

*Acomys selousi:* DNMNH-23834

*Acomys subspinosus:* DNMNH-40916, DNMNH-40988

*Petromyscus shortridgei:* DNMNH-23111, DNMNH-29251

*Petromyscus collinus:* DNMNH-23110, DNMNH-38887

*Steatomys krebsii:* DNMNH-3791, DNMNH-3790

*Steatomys pratensis:* IVB-VM274

*Dendromus nyikae:* DNMNH-24595, DNMNH-34695

*Dendromus mystacalis:* DNMNH-24766

*Mus minutoides:* IVB-JS052, IVB-RS1588

*Mus indutus:* DNMNH-45741, DNMNH-45489
